# Large range sizes link fast life histories with high species richness across wet tropical tree floras

**DOI:** 10.1038/s41598-024-84367-3

**Published:** 2025-02-08

**Authors:** Timothy R. Baker, Stephen Adu-Bredu, Kofi Affum-Baffoe, Shin-ichiro Aiba, Perpetra Akite, Miguel Alexiades, Everton Almeida, Edmar Almeida de Oliveira, Esteban Alvarez Davila, Christian Amani, Ana Andrade, Luiz Aragao, Alejandro Araujo-Murakami, Eric Arets, Luzmila Arroyo, Peter Ashton, Suspense A. Averti Ifo, Gerardo A. C. Aymard, Michel Baisie, William Balee, Michael Balinga, Lindsay F. Banin, Olaf Banki, Christopher Baraloto, Jorcely Barroso, Jean-Francois Bastin, Hans Beeckman, Serge Begne, Natacha Nssi Bengone, Nicholas Berry, Wemo Betian, Vincent Bezard, Lilian Blanc, Pascal Boeckx, Damien Bonal, Frans Bongers, Francis Q. Brearley, Roel Brienen, Foster Brown, Musalmah Bt. Nasaradin, Benoit Burban, David F. R. P. Burslem, Plinio Camargo, Jose Luis Camargo, Wendeson Castro, Carlos Ceron, Victor Chama Moscoso, Colin Chapman, Jerome Chave, Eric Chezeaux, Murray Collins, James Comiskey, David Coomes, Fernando Cornejo Valverde, Flavia R. C. Costa, Aida Cuni-Sanchez, Lola da Costa, Douglas C. Daly, Martin Dančák, Armandu Daniels, Greta Dargie, Stuart Davies, Charles De Canniere, Thales de Haulleville, Jhon del Aguila Pasquel, Geraldine Derroire, Kyle G. Dexter, Anthony Di Fiore, Marie-Noel K. Djuikouo, Jean-Louis Doucet, Vincent Droissart, Gerald Eilu, Thaise Emillio, Julien Engel, Bocko Yannick Enock, Fidele Evouna Ondo, Corneille Ewango, Sophie Fauset, Ted R. Feldpausch, Muhammad Fitriadi, Gerardo Flores Llampazo, Ernest G. Foli, Gabriella Fredriksson, David R. Galbraith, Martin Gilpin, Emanuel Gloor, Christelle Gonmadje, Rene Guillen Villaroel, Jefferson Hall, Keith C. Hamer, Alan Hamilton, Olivier Hardy, Terese Hart, Radim Hédl, Rafael Herrera, Niro Higuchi, Claude Marcel Hladik, Eurídice Honorio Coronado, Isau Huamantupa-Chuquimaco, Walter Huaraca Huasco, Wannes Hubau, Muhammad Idhamsyah, Sascha A. Ismail, Kath Jeffery, Eliana Jimenez, Tommaso Jucker, Elizabeth Kearsley, Lip Khoon Kho, Timothy Killeen, Kanehiro Kitayama, William Laurance, Susan Laurance, Miguel Leal, Simon L. Lewis, Stanislav Lhota, Jeremy Lindsell, Gabriela Lopez-Gonzalez, Jon Lovett, Richard Lowe, William E. Magnusson, Jean-Remy Makana, Yadvinder Malhi, Beatriz Marimon, Ben Hur Marimon Junior, Andrew Marshall, Colin Maycock, Faustin Mbayu, Casimiro Mendoza, Irina Mendoza Polo, Faizah Metali, Vianet Mihindou, Abel Monteagudo-Mendoza, Sam Moore, Patrick Mucunguzi, Jacques Mukinzi, Pantaleo Munishi, Laszlo Nagy, Petrus Naisso, David Neill, Adriano Nogueira Lima, Percy Nunez Vargas, Lucas Ojo, Walter Palacios, Nadir Pallqui Camacho, Alexander Parada Gutierrez, Julie Peacock, Kelvin S.-H. Peh, Antonio Pena Cruz, Colin Pendry, Toby Pennington, Maria Cristina Penuela-Mora, Pascal Petronelli, Oliver L. Phillips, Georgia Pickavance, G. John Pipoly, Nigel Pitman, Axel Dalberg Poulsen, Ghillean T. Prance, Adriana Prieto, Richard B. Primack, Lan Qie, Simon A. Queenborough, Terry Sunderland, Carlos Quesada, Freddy Ramirez Arevalo, Hirma Ramirez-Angulo, Jan Reitsma, Maxime Réjou-Méchain, Anand Roopsind, Francesco Rovero, Ervan Rutishauser, Kamariah Abu Salim, Rafael Salomao, Ismayadi Samsoedin, Muhd Shahruney Saparudin, Juliana Schietti, Ricardo A. Segovia, Julio Serrano, Rafizah Serudia, Douglas Sheil, Natalino Silva, Javier Silva Espejo, Marcos Silveira, Murielle Simo-Droissart, James Singh, Bonaventure Sonké, Thaise Emilio Lopes De Sousa, Juliana Stropp, Rahayu Sukri, Terry Sunderland, Martin Svátek, Michael Swaine, Hermann Taedoumg, Joey Talbot, Sylvester Tan, James Taplin, David Taylor, Hans ter Steege, John Terborgh, Armando Torres-Lezama, John Tshibamba Mukendi, Darlington Tuagben, Peter van de Meer, Geertje van der Heijden, Peter van der Hout, Mark van Nieuwstadt, Bert van Ulft, Rodolfo Vasquez Martinez, Ronald Vernimmen, Barbara Vinceti, Simone Vieira, Ima Celia Guimaries Vieira, Emilio Vilanova Torre, Jason Vleminckx, Lee White, Simon Willcock, Mathew Williams, John T. Woods, Tze Leong Yao, Ishak Yassir, Roderick Zagt, Lise Zemagho

**Affiliations:** 1 School of Geography, University of Leeds, Woodhouse Lane, Leeds, LS2 9JT UK; 2 Forestry Research Institute of Ghana (FORIG), Kumasi, Ghana; 3 Mensuration Unit, Forestry Commission of Ghana, Kumasi, Ghana; 4 Faculty of Environmental Earth Science, Hokkaido University, Sapporo, 060-0810 Japan; 5 College of Natural Sciences, Makerere University, Kampala, Uganda; 6 New York Botanical Garden, Bronx, NY 10458 USA; 7 Instituto de Biodiversidade e Florestas, Universidade Federal do Oeste do Pará, Belem, Brazil; 8 Universidade do Estado de Mato Grosso, Nova Xavantina, Brazil; 9 Fundación Con Vida, Medellín, Colombia; 10 Center for International Forestry Research, Goma, Democratic Republic of Congo; 11 Université Officielle de Bukavu, Bukavu, Democratic Republic of Congo; 12 Projeto Dinamica Biologica de Fragmentos Florestais, Instituto Nacional de Pesquisas da Amazonia, Manaus, Brazil; 13 Geography, University of Exeter, Exeter, EX4 4RJ UK; 14 National Institute for Space Research (INPE), Sao Jose dos Campos, SP Brazil; 15 Museo de Historia Natural Noel Kempff Mercado, Universidad Autónoma Gabriel Rene Moreno, Santa Cruz, Bolivia; 16 Wageningen Environmental Research, Wageningen University and Research, Wageningen, The Netherlands; 17 Department of Organismic and Evolutionary Biology, Harvard University, Cambridge, MA 02138 USA; 18 Université Marien N’Gouabi, Brazzaville, Republic of Congo; 19 UNELLEZ-Guanare, Programa de Ciencias del Agro y El Mar, Herbario Universitario (PORT), Guanare, Venezuela; 20 CIRAD, Forêts et Sociétés, 34398 Montpellier, France; 21 Tulane University, New Orleans, USA; 22 Tetra Tech ARD, Accra, Ghana; 23 UK Centre of Ecology and Hydrology, Edinburgh, UK; 24 Utrecht University, Utrecht, The Netherlands; 25 Naturalis Biodiversity Center, Leiden, The Netherlands; 26 International Center for Tropical Botany (ICTB), The Kampong of the National Tropical Botanical Garden, Miami, FL 33133 USA; 27 Department of Biological Sciences, Florida International University, Miami, FL 33199 USA; 28 Universidade Federal do Acre, Centro Multidiciplinar, Cruzeiro do Sul, Brazil; 29 TERRA Teaching and Research Centre, Gembloux Agro Bio-Tech, Université de Liège, Liège, Belgium; 30 Laboratory for Wood Biology and Xylarium, Royal Museum for Central Africa, Brussels, Belgium; 31 Plant Systematic and Ecology Laboratory, Department of Biology, Higher Teachers Training College, University of Yaounde I, Soa, Cameroon; 32 Ministry of Forests, Seas, Environment and Climate, Libreville, Gabon; 33 The Landscapes and Livelihoods Group, Edinburgh, UK; 34 CNRS, Forêts et Sociétés, 34398 Montpellier, France; 35 ONF, Forêts et Sociétés, 34398 Montpellier, France; 36 CIRAD, Forêts et Sociétés, 34398 Montpellier, France; 37 Department of Green Chemistry and Technology, Isotope Bioscience Laboratory-ISOFYS, Ghent University, Ghent, Belgium; 38 INRAE, UMR SILVA, Champenoux, France; 39 Forest Ecology and Forest Management Group, Wageningen University, Wageningen, The Netherlands; 40 Department of Natural Sciences, Manchester Metropolitan University, Manchester, M1 5GD UK; 41 Woods Hole Research Center, Falmouth, USA; 42 Forest Research Institute of Malaysia, Kuala Lumpur, Malaysia; 43 INRAE, UMR Ecofog, Kourou, French Guiana; 44 School of Biological Sciences, University of Aberdeen, Aberdeen, AB24 3FX UK; 45 Centro de Energia Nuclear Na Agricultura, Universidade de São Paulo, São Paulo, Brazil; 46 Botany and Plant Ecology Laboratory, Federal University of Acre, Rio Branco, Brazil; 47 Herbario Alfredo Paredes (QAP), Universidad Central del Ecuador, Quito, Ecuador; 48 Universidad Nacional de San Antonio Abad del Cusco, Cusco, Peru; 49 Biology Department, Vancouver Island University, Nanaimo, BC V9R 5S5 Canada; 50 Laboratoire EDB, CNRS, IRD, Université Paul Sabatier, 31062 Toulouse, France; 51 Rougier-Gabon, Libreville, Gabon; 52 School of GeoSciences, University of Edinburgh, Edinburgh, EH8 9XP UK; 53 Space Intelligence Ltd, Edinburgh, EH3 2ES UK; 54 National Park Service, Washington, DC USA; 55 Smithsonian Institution, Washington, DC USA; 56 Department of Plant Sciences, University of Cambridge, Cambridge, CB2 3EA UK; 57 Andes to Amazon Biodiversity Program, Lima, Peru; 58 INPA-Brazilian National Institute of Amazonian Research, Manaus, Brazil; 59 Department of International Environmental and Development Studies (NORAGRIC), Norwegian University of Life Sciences, Ås, Norway; 60 Geography, University of York, York, UK; 61 Universidade Federal do Para, Belém, Brazil; 62 Department of Ecology and Environmental Sciences, Faculty of Science, Palacky University, Olomouc, Czech Republic; 63 Forestry Development Authority of the Government of Liberia (FDA), Monrovia, Liberia; 64 ForestGEO, Smithsonian Tropical Research Institute, Washington, DC 20013-7012 USA; 65 Agroecology Lab, Université Libre de Bruxelles, Brussells, Belgium; 66 Instituto de Investigaciones de La Amazonia Peruana, Iquitos, Peru; 67 Universidad Nacional de la Amazonía Peruana, Iquitos, Peru; 68 Cirad, UMR EcoFoG (AgroParistech, CNRS, INRAE, Université des Antilles, Université de La Guyane), Kourou, French Guiana; 69 Royal Botanic Garden Edinburgh, Edinburgh, EH3 5NZ UK; 70 Department of Life Sciences and Systems Biology, University of Turin, Turin, Italy; 71 Department of Anthropology and Primate Molecular Ecology and Evolution Laboratory, The University of Texas at Austin, Austin, USA; 72 Tiputini Biodiversity Station, Universidad San Francisco de Quito, Quito, Ecuador; 73 Department of Plant Science, Faculty of Science, University of Buea, Buea, Cameroon; 74 TERRA Teaching and Research Centre, University of Liege, Liege, Belgium; 75 AMAP, Univ. Montpellier, IRD, CNRS, CIRAD, INRAE, Montpellier, France; 76 College of Agricture and Environmental Studies, Makerere University, Kampala, Uganda; 77 São Paulo State University (UNESP), Institute of Biosciences, Campus Rio Claro, Center for Research On Biodiversity Dynamics and Climate Change (CBioClima), São Paulo, Brazil; 78 Agence Nationale des Parcs Nationaux Gabon, Libreville, Gabon; 79 Wildlife Conservation Society-DR Congo, Brazzaville, Republic of the Congo; 80 School of Geography, Earth and Environmental Sciences, University of Plymouth, Devon, PL4 8AA UK; 81 Sungai Wain Protection Forest, Balikpapan City, Indonesia; 82 Sunbear Conservation Centre, Samboja, Indonesia; 83 Institute for Biodiversity and Ecosystem Dynamics, University of Amsterdam, Amsterdam, The Netherlands; 84 National Herbarium, Yaounde, Cameroon; 85 Smithsonian Tropical Research Institute, Smithsonian Institution Forest Global Earth Observatory (ForestGEO), Panama City, Panama; 86 School of Biology, University of Leeds, Leeds, LS2 9JT UK; 87 Kunming Institute of Botany, Chinese Academy of Sciences, Beijing, China; 88 Evolutionary Biology and Ecology, Universite Libre de Bruxelles (ULB), Brussels, Belgium; 89 Lukuru Wildlife Research Foundation, Circleville, USA; 90 Division of Vertebrate Zoology, Yale Peabody Museum of Natural History, New Haven, USA; 91 Institute of Botany, Czech Academy of Sciences, Prague, Czechia; 92 Department of Botany, Faculty of Science, Palacky University in Olomouc, Czechia; 93 Instituto Venezolano de Investigaciones Científicas (IVIC), Caracas, Venezuela; 94 Instituto Nacional de Pesquisas da Amazónia-Coordenaçao de Pesquisas em Silvicultura Tropical, Manaus, Brazil; 95 Departement Hommes Natures Societes, Museum National d’Histoire Naturelle, 75005 Paris, France; 96 Royal Botanic Gardens Kew, London, UK; 97 Herbario Alwyn Gentry (HAG), Universidad Nacional Amazónica de Madre de Dios (UNAMAD), Puerto Maldonado, Peru; 98 Department of Forest and Water Management, Laboratory of Wood Technology, Ghent University, Ghent, Belgium; 99 Balitek-KSDA, Samboja, Indonesia; 100 Swiss Academy of Sciences (SCNAT), Bern, Switzerland; 101 CENAREST and ANPN and Stirling University, Stirling, UK; 102 Universidad Nacional de Colombia, Bogotá, Colombia; 103 School of Biological Sciences, University of Bristol, Bristol, BS8 1TQ UK; 104 CAVElab-Computational and Applied Vegetation Ecology, Ghent University, 9000 Gent, Belgium; 105 Ministry of Energy and Environmental Sustainability Sarawak, Kuching, Malaysia; 106 World Wide Fund for Nature, Gland, Switzerland; 107 Graduate School of Agriculture, Kyoto University, Kyoto, Japan; 108 Centre for Tropical Environmental and Sustainability Science (TESS), College of Science and Engineering, James Cook University, Cairns, QLD 4878 Australia; 109 Wildlife Conservation Society, New York, USA; 110 Department of Geography, University College London, London, WC1E 6BT UK; 111 Czech University of Life Sciences Prague, Prague, Czechia; 112 A Rocha International, Cambridge, UK; 113 The Royal Society for the Protection of Birds, Centre for Conservation Science, Sandy, UK; 114 Royal Botanic Gardens, Kew, Richmond, London, TW9 3AE UK; 115 Environmental Change Institute, School of Geography and the Environment, University of Oxford, Oxford, UK; 116 Universiti Malaysia Sabah, Kota Kinabalu, Malaysia; 117 Université de Kisangani Faculté des Sciences Agronomiques République Démocratique du Congo, Kisangani, Democratic Republic of the Congo; 118 Forest Management in Bolivia, La Paz, Bolivia; 119 Jardin Botanico de Medellin, Grupo de Investigacion en Servicios Ecosistemicos y Cambio Climatico, Medellín, Colombia; 120 Environmental and Life Sciences, Faculty of Science, Universiti Brunei Darussalam, Gadong, Brunei; 121 World Wide Fund for Nature, Gland, Switzerland; 122 Wildlife Conservation Society, Salonga National Park, Ikali, Democratic Republic of the Congo; 123 Sokoine University of Agriculture, Morogoro, Tanzania; 124 University of Campinas, Campinas, Brazil; 125 Universidad Estatal Amazónica, Facultad de Ingeniería Ambiental, Pastaza, Ecuador; 126 University of Abeokuta, Adzho, Nigeria; 127 Universidad Tecnica del Norte, Herbario Nacional del Ecuador, Quito, Ecuador; 128 Biological Sciences, University of Southampton, Southampton, SO17 1BJ UK; 129 Jardin Botanico de Missouri, Oxapampa, Peru; 130 Universidad Regional Amazonica Ikiam, Tena, Ecuador; 131 CIRAD, UMR Ecologie des Foréts de Guyane, Kourou, French Guiana; 132 Broward County Parks and Recreation, FAU, Nova SE Univ, Fort Lauderdale, USA; 133 Nova Southeastern University, Fort Lauderdale, USA; 134 Science and Education, The Field Museum, Chicago, USA; 135 Instituto de Ciencias Naturales, Universidad Nacional de Colombia, Bogotá, Colombia; 136 Department of Biology, Boston University, Boston, MA 02215 USA; 137 School of Life and Environmental Sciences, University of Lincoln, Lincoln, LN6 7TS UK; 138 Escuela de Ciencias Biológicas, Pontificia Universidad Católica del Ecuador, Quito, Ecuador; 139 University of British Columbia, Vancouver, Canada; 140 Universidad de los Andes, Merida, Venezuela; 141 Bureau Waardenburg BV, Culemborg, The Netherlands; 142 Iwokrama International Centre for Rainforest Conservation and Development, Georgetown, Guyana; 143 Department of Biology, University of Florence, Florence, Italy; 144 MUSE - Science Museum, Trento, Italy; 145 CarboFor, São Paulo, Brazil; 146 Museu Paraense Emílio Goeldi, Belen, Brazil; 147 Forest Research and Development Agency (FORDA), Cornwall, UK; 148 Institute of Ecology and Biodiversity (IEB), Santiago, Chile; 149 Servicio Florestal Brasileiro, Brasília, Brazil; 150 Departamento de Biología, Universidad de La Serena, La Serena, Chile; 151 Museu Universitario, Universidade Federal do Acre, Rio Branco, Brazil; 152 AMAP Lab, IRD, CIRAD, CNRS, INRA, Université de Montpellier, Montpellier, France; 153 Plant Systematic and Ecology Laboratory, Department of Biology, Higher Teacher Training College, University of Yaoundé I, Yaoundé, Cameroon; 154 Guyana Forestry Commission, Georgetown, Guyana; 155 Joint Research Centre of the European Commission, Brussels, Belgium; 156 Mendel University in Brno, Faculty of Forestry and Wood Technology, Brno, Czech Republic; 157 University of Yaounde, Soa, Cameroon; 158 Biodiversity International, Rome, Italy; 159 Sarawak Forestry Corporation, Kuching, Malaysia; 160 Harvard University, Cambridge, USA; 161 Forum for the Future, London, UK; 162 UK Research and Innovation, Swindon, UK; 163 Department of Geography, National University of Singapore, Singapore, Singapore; 164 Florida Museum of Natural History and Department of Biology, University of Florida - Gainesville, Florida, 32611 USA; 165 Forestry Development Authority of the Government of Liberia (FDA), Kamkarn Town, Liberia; 166 Van Hall Larenstein University of Applied Sciences, Leeuwarden, The Netherlands; 167 University of Nottingham, Nottingham, UK; 168 Van der Hout Forestry Consulting, Rotterdam, The Netherlands; 169 PROMAB, Richmond, USA; 170 Data for Sustainability, Axel, 4571 AK The Netherlands; 171 Bioversity International, Rome, Italy; 172 Center for Environmental Studies and Research, University of Campinas, Campinas, Brazil; 173 Service Evolution Biologique et Ecologie, Brussels, Belgium; 174 Florida International University, Miami, USA; 175 Institut de Recherche en Ecologie Tropicale (CENAREST) Gabon/Agence Nationale des Parcs Nationaux, Libreville, Gabon; 176 Net Zero and Resilient Farming, Rothamsted Research, Harpenden, Hertfordshire, AL5 2JQ UK; 177 School of Environmental and Natural Sciences, Bangor University, Bangor, Gwynedd LL57 2DG UK; 178 University of Liberia, Monrovia, Liberia; 179 Tropenbos International, Ede, The Netherlands

**Keywords:** Tropical forest, Diversification, Biodiversity, Trees, Evolution, Biogeography, Biodiversity, Forest ecology, Macroecology, Tropical ecology

## Abstract

Understanding how the traits of lineages are related to diversification is key for elucidating the origin of variation in species richness. Here, we test whether traits are related to species richness among lineages of trees from all major biogeographical settings of the lowland wet tropics. We explore whether variation in mortality rate, breeding system and maximum diameter are related to species richness, either directly or via associations with range size, among 463 genera that contain wet tropical forest trees. For Amazonian genera, we also explore whether traits are related to species richness via variation among genera in mean species-level range size. Lineages with higher mortality rates—faster life-history strategies—have larger ranges in all biogeographic settings and have higher mean species-level range sizes in Amazonia. These lineages also have smaller maximum diameters and, in the Americas, contain dioecious species. In turn, lineages with greater overall range size have higher species richness. Our results show that fast life-history strategies influence species richness in all biogeographic settings because lineages with these ecological strategies have greater range sizes. These links suggest that dispersal has been a key process in the evolution of the tropical forest flora.

## Introduction

Species richness varies across the branches of the tree of life depending both on the traits and biogeographical setting of each lineage^1^. Traits influence the propensity of a lineage to disperse, adapt and diverge from ancestral populations, as well as survive extinction events^1–3^. In contrast, the biogeographical setting of a lineage relates to the location and history of the landscape where the lineage is found, and determines its exposure to processes that influence speciation and extinction, such as mountain building, tectonic movements and environmental change over geological timescales^4,5^. Understanding the interplay of these extrinsic factors and traits is important for developing a comprehensive understanding of the trajectory of evolution^6^. This debate is particularly important in the context of the tree flora of the wet tropics, as up to 53,000 of the global total of ca. 73,000 tree species occur in old-growth, closed-canopy wet tropical forests^7–9^.

There are three broad biogeographical settings across wet tropical forests: the Americas, characterised by the largest area of contiguous forest, rapid mountain uplift and shifting drainage patterns during recent geological history^10^; Africa, characterised by forests that have experienced substantial fluctuations in area during glacial and interglacial cycles^11^; and SE Asia, characterised by island formation driven by fluctuations in sea level^12^. These settings are considered to have promoted high speciation rates, in the case of the Americas and SE Asia, and high extinction rates, in the case of Africa. Importantly, these settings are typically assumed to be more important than the traits of any lineage for determining patterns of diversity^4,5,13,14^. For example, the high species richness of *Hirtella* compared to other genera of Chrysobalanaceae has been argued to be a result of the colonisation of the Americas by this lineage^15^: the setting, rather than any specific traits associated with this genus, is thought to have led to high rates of diversification in this group. However, the traits that a lineage possesses can also affect diversification rates. For example, faster demographic rates are linked to high species richness among a range of Amazonian tree lineages^16^. Here, we therefore test how traits are associated with species richness among genera of the tree flora across the three major biogeographical settings of the lowland wet tropics.

Traits influence diversification by affecting the wide range of processes that underlie speciation and extinction^17,18^. For example, traits affect the degree to which lineages form isolated populations, which is commonly an initial step towards speciation^18–20^. Traits also influence subsequent stages of speciation, associated with genetic divergence, the emergence of ecological and reproductive isolation, the ability of new species to persist and the interactions among these processes^18–21^. Traits may also affect extinction rates by conferring resistance to a specific environmental change that causes populations to decline^17^ or by influencing the ability of a lineage to disperse and migrate^22^. A common feature of many of these mechanisms of speciation and extinction is that they are spatial processes. As a result, lineage range size is positively correlated with species richness among plant lineages^23,24^ because larger ranges increase speciation rates by peripatric and parapatric processes (i.e. allopatric speciation involving geographic isolation^25^ and selection over environmental gradients^26^), and decrease extinction rates^17^. It is therefore important to understand if traits promote higher species richness of lineages because they are associated with large range sizes, or because traits may influence other processes linked to high speciation rates and low extinction rates, such as the rates of ecological differentiation and genetic divergence^18^.

In tropical wet forests, previous work on understanding variation in species richness among lineages has either focussed on individual groups^15^, considered a single region^16^ or excluded traits^27^. Here, we therefore compare the relationships among traits and species richness of 463 genera across wet forests in the Americas, Africa and SE Asia. We explore the role of three key traits, (1) stem-level mortality rate of genera, as a proxy of generation time, which we expect to correlate positively with species richness, as a ‘live fast, die young’ strategy may allow faster rates of adaptation to novel environmental conditions^16^; (2) maximum tree size, as a proxy of dispersal ability, which we expect to be negatively correlated with species richness, as limited dispersal may enhance reproductive isolation^28^; and (3) dioecy, which we expect to be negatively associated with species richness, as it may reduce the ability of isolated populations to establish^29^. We estimated these traits using demographic and structural data from a pan-tropical network of permanent forest plots^30,31^, range size estimates based on botanical records^32^, and information on breeding systems from floras (SI Table S5). We used generalised least squares (GLS) to explore the associations among traits, range size and species richness, and piecewise structural equation models (pSEM)^33^ to assess whether traits influence species richness directly, or indirectly via variation in range size.

We performed an additional analysis for genera from the Americas, which also incorporated variation in genus population size^34^, dispersal mode^35^ and mean species range size within genera^36^, based on published datasets that are only available for this continent. We expected genus population size to be positively correlated with species richness, given sampling considerations, and dispersal by wind to be associated with lower species richness, as higher dispersal distances may preclude effective reproductive isolation. Finally, we predicted that smaller species-level range sizes would be associated with greater species richness within a genus, as small ranges and high diversification rates are a feature of lineages in endemic hotspots associated with for example, mountain building and island formation, in the tropics^37^.

In all analyses, we accounted for the phylogenetic relationships among genera in these analyses using a pantropical, DNA-based, genus-level phylogeny, developed from a published genus-level phylogeny for American trees^38,39^.

## Results

**Fig. 1:**
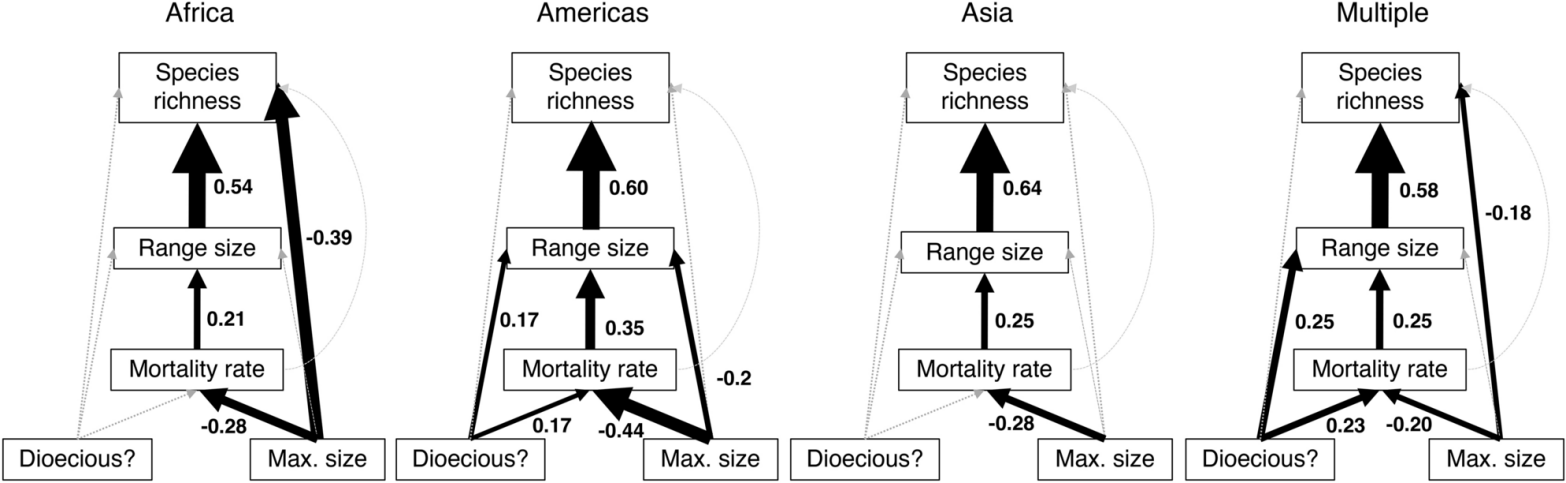
Structural equation models from pSEM analysis showing the relationships between traits, range size and species richness for 463 genera of tropical trees, that occur in the Americas, Africa, Asia or on multiple continents. Standardised effect sizes shown for significant relationships; arrow width is proportional to the standardised effect size. Non-significant relationships are shown with grey dotted lines.

**Fig. 2:**
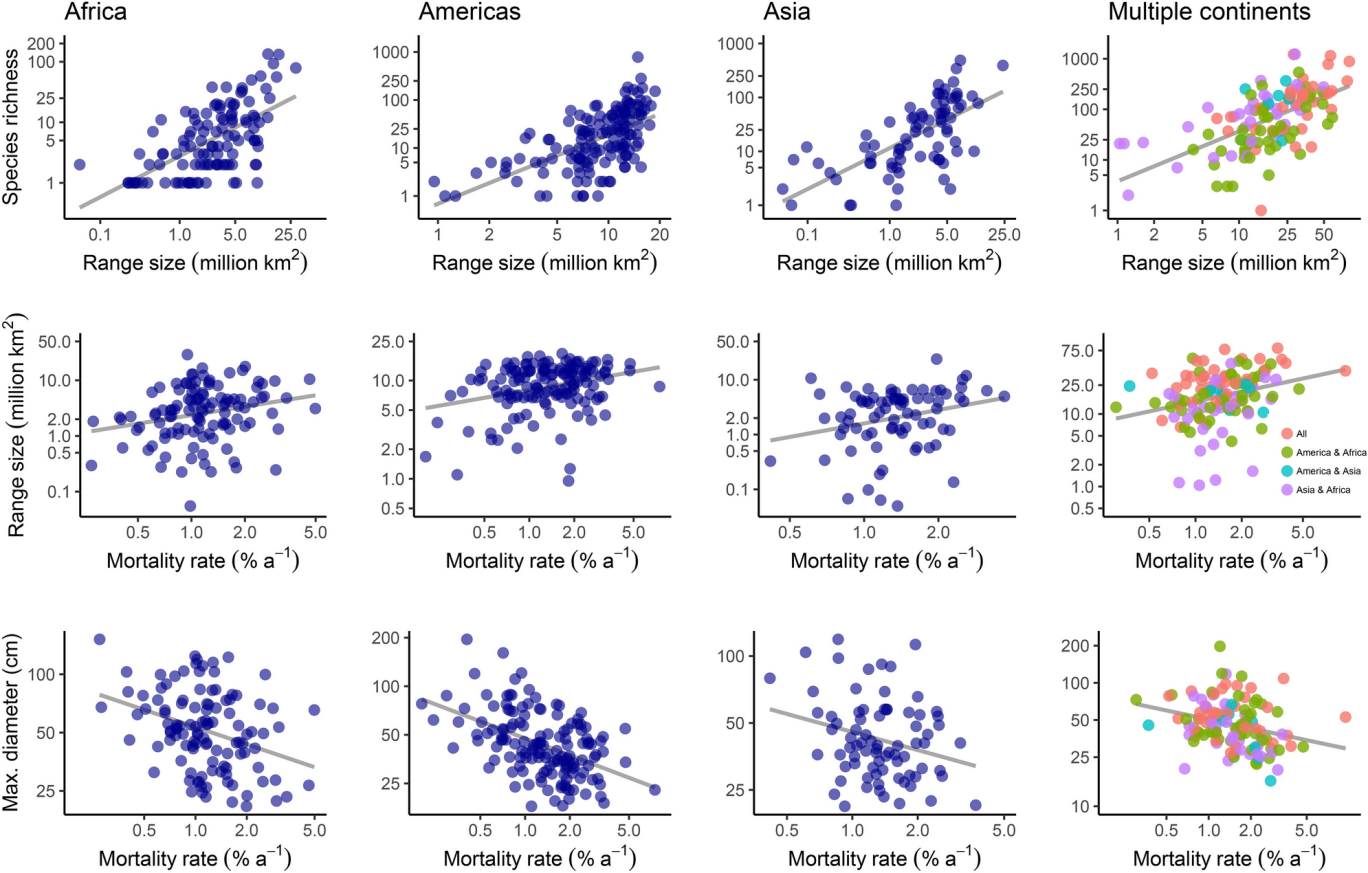
Relationship between (**a**) range size and species richness, (**b**) mortality rate and range size and (**c**) maximum diameter and mortality rate for 463 genera of tropical trees. Genera are grouped by their distribution in American, African or Asian tropical forests or presence in multiple continents. Regression lines show GLS relationships from pSEM models shown in this figure; all relationships are significant and account for the phylogenetic relationships among lineages. Note that y-axes are scaled differently to optimise display of the relationships within each biogeographic setting.

**Fig. 3:**
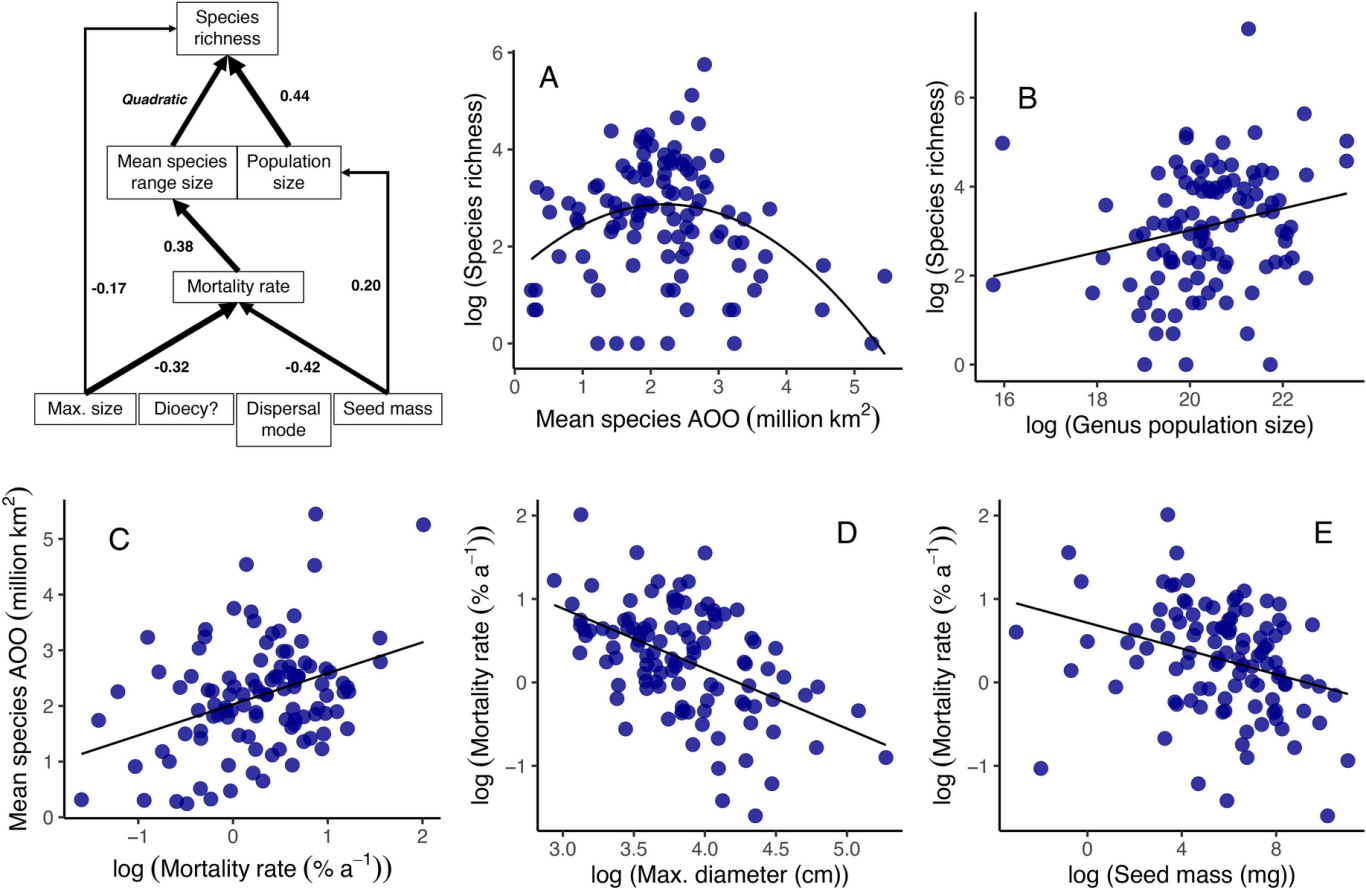
Structural equation models and key univariate relationships from pSEM analysis between traits, mean species range size and species richness for 105 genera of Amazonian trees. Standardised effect sizes are shown for significant relationships and arrow width is proportional to the standardised effect size, apart from for significant quadratic relationship between mean species range size where arrow width where this is not possible to calculate. Boxes that are contiguous have significant correlated errors. Non-significant relationships are omitted for clarity. Univariate relationships between species richness and (**A**) mean species range size (AOO), and (**B**) genus population size, (**C**) mortality rate and mean species range size, (**D**) maximum diameter and (**E**) seed mass.

**Fig. 4:**
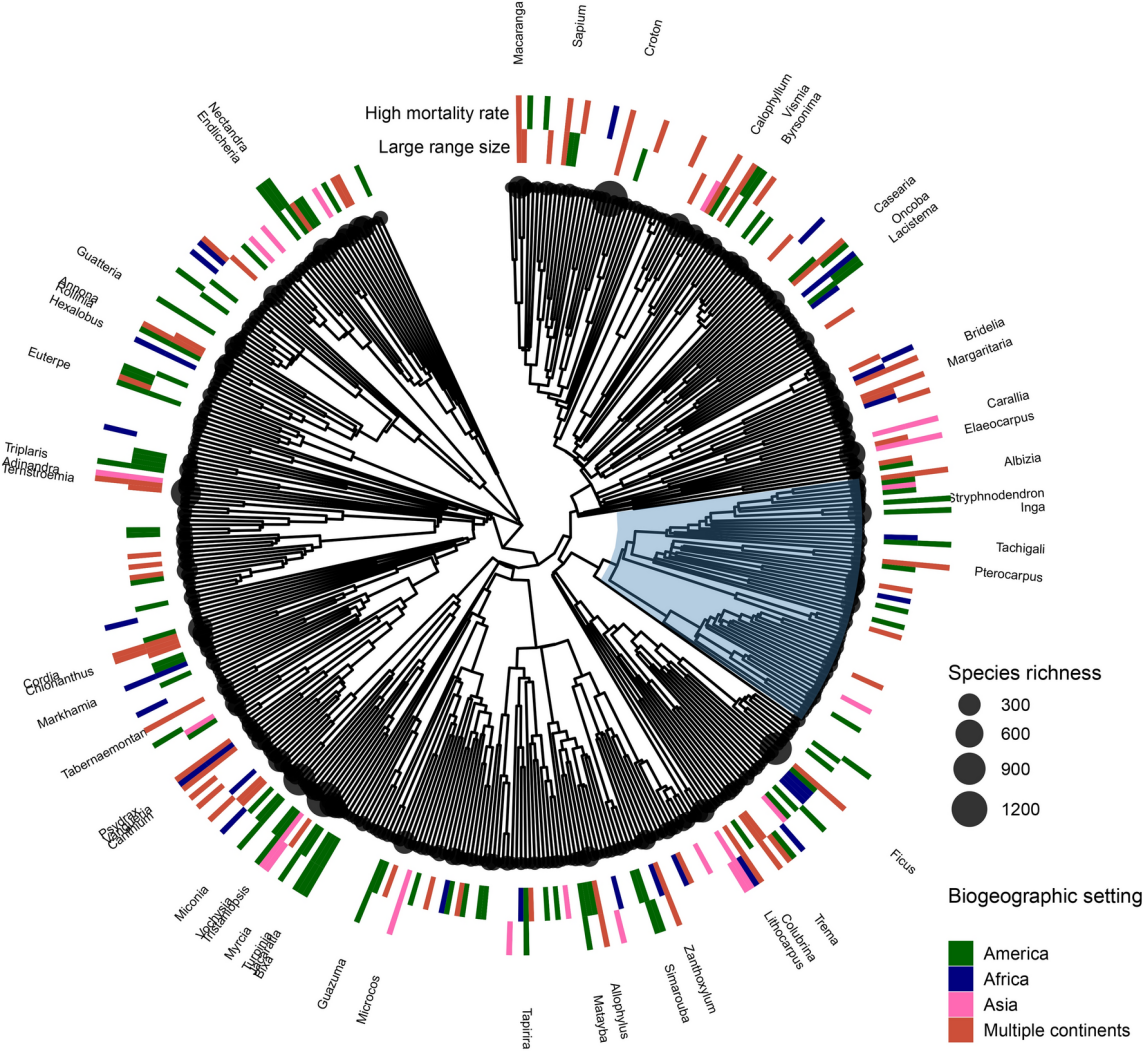
Phylogenetic relationships among species richness, high mortality rates and large range size for 463 genera of tropical trees. Tip circle size is proportional to the species richness of each genus. Coloured bars indicate genera with high mortality rates and/or large range sizes. High mortality rates are classified as > 2% a^−1^; large range size classified as > 10 M km^2^ (America), > 8 M km^2^ (Africa), > 5 M km^2^ (Asia) and > 20 M km^2^ (multiple continents). Bar colour indicates the biogeographic setting of each genus. Named genera are those that share both high mortality rates and large range sizes; their distributed pattern illustrates that the association between large range size and high mortality rates is found across the phylogeny and is not restricted to certain clades. The blue segment on the phylogeny highlights the legumes, including the Dialioideae and Detarioideae subfamiles which are the only major clades on the phylogeny that do not demonstrate this association.

Variation in mortality rates was positively correlated with lineage range size using GLS analysis and in every biogeographic setting using pSEM (Figs. 1 and 2; Table S1). This relationship was also significant for a subset of genera that solely comprise lowland, tropical wet forest trees (Fig. S1) and, among Amazonian genera, mortality rates were positively correlated with mean species range size (Fig. 3). These relationships were driven by lineages from across the phylogeny that share high mortality rates and large range sizes (Fig. 4; e.g. Americas, *Inga,* mortality rate 2.8% a^−1^, range size 15.5 million km^2^; Africa, *Uapaca*, 1.6% a^−1^, range size 13.6 million km^2^; Asia, *Elaeocarpus*, 2.5% a^−1^, range size 7.2 million km^2^). The only major clades that do not contain genera with these linked characteristics are the Dialioideae and Detarioideae subfamilies of legumes (Fig. 4). Overall, these results indicate that fast demography is associated with greater range sizes across the phylogeny and biogeographical settings of the wet tropical tree flora (Fig. 2).

In turn, lineage range size was strongly associated with variation in species richness: genera with large range sizes have greater species richness (Figs. 1 and 2, Table S1). The link between large range size and high species richness was found in all biogeographical settings and across the phylogeny (e.g. Americas, *Inga* (15.5 million km^2^; 281 species), *Protium* (18.8 million km^2^; 152 species); Africa, *Uapaca* (13.6 million km^2^; 25 species), *Cola* (12.9 million km^2^, 134 species); Asia, *Aglaia* (6.6 million km^2^; 119 species), *Elaeocarpus* (7.2 million km^2^, 488 species). In contrast to these linear relationships between genus range size and species richness, mean species-level range size within genera had a unimodal relationship with species richness in Amazonia: richness peaked at intermediate values and declined in lineages with the greatest mean species-level range size (Fig. 3A). This quadratic relationship between mean species range size and species richness in Amazonia was independent of a significant positive correlation between population size and species richness (Table S2, Fig. 3B).

Variation in a range of other traits were linked to variation in mortality rates among genera (Fig. 1). There was a consistent negative relationship in all biogeographic settings between mortality rate and maximum size: across the phylogeny, genera with smaller maximum size have high mortality rates (Fig. 2 and Fig. S2). For Amazonian trees, low seed mass was also associated with high mortality rates (Fig. 3). Finally, the presence of dioecy was also associated with high mortality rates for genera that occur on multiple continents and those in Amazonian forests (Figs. S3 and S4). Overall, these relationships indicate that there are linked suites of traits related to stature, dispersal and breeding system that are all associated with fast life-history strategies, larger range sizes of lineages and ultimately, greater species richness (Fig. 1).

In some biogeographical settings, there were direct relationships between traits and species richness that were independent of variation in range size (Fig. 1). For example, species richness is higher in genera with low stature from African forests and those on multiple continents (Fig. S3), but there is no direct association between stature and species richness in Asian forests (Fig. 1 and Fig. S3).

## Discussion

This study demonstrates that fast life-history strategies are associated with greater range sizes of lineages of tropical trees in all biogeographical settings, as well as larger mean species-level range sizes amongst Amazonian trees (Figs. 2 and 3). Fast life-history strategies are associated with high dispersal rates, such as low seed mass (Fig. 3), and greater dispersal ability is known to be linked with greater range sizes across a wide variety of groups^22^. However, links between life-history strategies and range size are poorly documented among plants, particularly within the tropics^22^. Fast life-history strategies are associated with large range sizes amongst temperate and boreal tree species^22,40^ and for tropical trees, there is an association between smaller range sizes and slow life-history strategies among 35 species in Costa Rica^41^, as well as between smaller range sizes and greater fruit mass for palms^42^. However, our study is the first to identify a link between fast life-history strategies and large range sizes across the entire tropical tree flora.

In turn, this study shows that the link between demographic rates and species richness is mediated by lineage range size (Fig. 1). The link between demographic rates and species richness is consistent with findings for birds and mammals^43^, a study of North American trees and shrubs^44^, and previous findings for Amazonian trees, where higher mortality rates were associated with higher species richness among 51 genera^16^. However, the results here are novel in two respects. First, this study identifies that range size mediates the link between demographic rates and species richness (Fig. 1). Second, we show that the relationship is universal across all three major biogeographical settings of the tropics. Genera with higher mortality rates are associated with greater species richness whether they have evolved and colonised landscapes influenced by geological events that are characterised by high rates of speciation, such as mountain- or island-building in Amazonia or SE Asia, or high rates of extinction, such as the prolonged drier periods that have characterised the African tropics^14^; the relationship is independent of geological history.

One process that may underpin these relationships may be more rapid colonisation of new landscapes by genera with higher mortality rates, as lineages with fast life-history strategies reproduce more frequently and have traits such as low seed mass that may allow longer-distance seed dispersal^45^. For Amazonian forests, higher mortality rates are linked to low seed mass among genera (Fig. 3) and at a species-level, to low seed volume^46^. Faster rates of colonisation and achieving greater range sizes may promote higher speciation rates and lower extinction rates^47^. For example, larger range sizes are more likely to intersect with a geological event that divides a population, leading to speciation via vicariance, and small populations at the edges of large ranges are more likely to be isolated for sufficient time for peripatric speciation to occur^25,26,28^. Such processes have been invoked to explain patterns of species richness and diversity among islands in SE Asia^48^ and have been suggested as a reason why closely related species within genera do not tend to occur together in Amazonia^28,49^. However, the unimodal relationship between mean species-level range size and species-richness for genera of Amazonian trees (Fig. 3), suggests that high dispersal ability may not be the only process that underpins these patterns. This decline in the species richness of lineages with high mean species-level range sizes is consistent with the idea that speciation rates decline with very high dispersal rates among populations because it becomes harder for ecological and genetic isolation to emerge^18^.

A second process that therefore may also be important is that higher mortality rates and therefore shorter generation times may be linked with higher rates of genetic change^50^ that allow faster adaptation to novel environmental conditions. For example, higher rates of genetic change in genera with faster life-history strategies may have promoted the emergence of adaptations to novel environmental conditions, such as new soil types^51^, over geological timescales. This mechanism is also consistent with predictions from eco-evolutionary theory and experimental studies of insects that indicate that rapid evolution enhances range expansion into novel habitats^52,53^.

The link between small stature and high species-richness in African, but not Amazonian or Asian lineages, may reflect an association between maximum size and resistance to a specific environmental change that causes extinction: an ability to tolerate drought^54,55^. The impact of more extended drought periods during the geological history of forests in Africa^5,56^ may have elevated extinction rates within lineages of large-stature trees, leading to few species-rich, large-stature genera today. Although speculative, this argument suggests that the effect of some traits on species richness at least, may be contingent on the biogeographic setting of the lineage and may be linked to effects on rates of extinction, rather than speciation.

The finding that dioecy is positively associated with species richness in American forests, via associations with high mortality rates and larger range sizes (Fig. 1), contradicts the classic idea that dioecy should be an evolutionary ‘dead-end’^29,57^. However, these findings are consistent with a broad array of recent work showing that dioecy is common in tropical secondary forests^58^ and is linked with large geographical range sizes of genera^59^, species richness is higher in dioecious clades once variation in branch length between non-dioecious and dioecious clades is accounted for^60^ and genetic diversity is greater and adaptation rates are faster in dioecious compared to monoecious lineages of plants^61^. The lack of significant associations between dioecy, mortality rates and range size in African and Asian forests may reflect the lower levels of sampling in these regions; significant relationships among these variables are only found in the two groups with the greatest number of genera (Fig. 1). Overall, our results suggest that dioecy in the tropical tree flora, at least for the Americas, may be related to a suite of traits, such as short generation times and high dispersal ability that more than compensate for the difficulty of achieving successful reproduction when sexes are on separate individuals. More generally, our findings demonstrate the need for precise estimates of species’ demographic rates to understand the links between breeding systems and species richness in comparative analyses.

There are uncertainties associated with the estimates of range size, taxonomy and traits that we used in this study. First, range sizes have fluctuated over the evolutionary history of the extant diversity of tropical forests, and time-integrated estimates of range size are required to estimate the land area that has been available for each genus more precisely over geological time^62^. Second, the number of herbarium records that we used to assess the extant range sizes of the constituent species of genera varies greatly among and within regions. For example, there are more records for each genus for American than for African and Asian forests (Fig. S5), whilst for genera from Africa, most botanical sampling of wet forests is restricted to the Atlantic coast^63^. Range sizes of some Asian genera are also low, even after acknowledging the island distribution of many taxa (Fig. S6). We expect that estimates of range sizes may increase with more records and coverage, and particularly as African herbaria become digitised. Although these limitations make it difficult to compare the influence of a specific biogeographic setting on species richness today in these analyses, neither increased collections nor integrating changes in biome area over geological time are likely to alter the consistent positive direction of the relationships between range size and species richness, and between mortality rates and range size (Fig. 1).

A third source of uncertainty, are the genus concepts that are used to frame these analyses. Genera are readily identified even within the most diverse tropical flora and therefore provide confidence that we can unite disparate datasets on traits, ecology and phylogenetics^64^. However, we recognise that the process of re-circumscribing and/or confirming genera as monophyletic using DNA sequence-based phylogenies is an on-going process^65,66^. We also recognise the lack of equivalence amongst genera because, even using a monophyly criterion, taxonomists may be able to circumscribe one large, or several smaller genera^67^. However, the tendency either to lump or split genera, will not affect the parameter estimates of our GLS or pSEM analyses as the phylogenetic covariance error structure weights these analyses based on the shared branch length among the tips of the phylogeny. Finally, the ecological traits we use are only proxies for the ability of populations to disperse, adapt and diverge genetically. For example, the use of average mortality rates of trees ≥ 10 cm diameter as a proxy for generation times assumes that the onset of fruit production occurs when trees reach 10 cm diameter and then remains constant until tree death, and that passage time from seed germination to 10 cm diameter is correlated with life expectancies beyond 10 cm diameter^16^. Studies of juvenile growth and reproductive phenology of tropical trees suggests that these are reasonable simplifications for the tropical tree flora but clearly obscure variation among lineages. For example, the mean minimum diameter of reproduction of 12 tree species in moist forest in Panama was 14.8 cm, but individual species varied from 6.1 to 46.7 cm^68^. However, across lineages, average lifespan beyond 10 cm diameter correlates with estimates of total generation time because adult survival is such a large and variable component of tree lifespans^16^. Finally, we note that there are likely other traits that influence diversification that are not included here, such as those associated with pollination and reproductive structures and strategies^69^. Inclusion of such traits may increase the importance of traits that influence species richness directly, through altering the likelihood and length of transition times for genetic divergence^21^.

Overall, our results indicate the importance of ecological traits for understanding variation in range size and species richness among genera of tropical trees. Further studies are required to understand the degree of overlap of species ranges within genera, in terms of both geographical and environmental space, and to explore how the signature of founder effects in the genetic structure of species populations varies among lineages with different demographic traits^70,71^. Phylogenetic analyses of species traits, distributions and demography using comprehensive species-level phylogenies of lineages that vary across the full spectrum of life-history strategies are also required to tease apart the underpinning mechanisms that have driven diversification. Obtaining suitable species-level demographic data means that we need to overcome the challenge that most taxa of tropical trees are rare, and knowledge of the characteristics of this rare majority is crucial for understanding how the high biodiversity of tropical forests evolved. Addressing this challenge involves both making a continued commitment to supporting and expanding on-the-ground monitoring and working to ensure accurate and consistent identifications are used across disciplines^64^. For tropical trees, the now extensive data across multiple environmental gradients from long-term plot networks^30,31^ provide a platform for this research and could provide the precise and comparable estimates of demographic rates that are required.

## Methods

### Plot and genus selection

This study is based on a novel compilation of long-term inventory data from 655 plots located in old-growth and secondary forests from across the tropics (Table S3; Fig. S7). These data are derived from the RAINFOR, AfriTRON, T-FORCES and CTFS-ForestGeo networks and are curated either at ForestPlots.net^30^ or by CTFS-ForestGeo^31^. Plot size varied from 0.5 to 50 ha. We selected all individual stems ≥ 10 cm diameter that had two or more measurements where the status of the tree (alive or dead) was recorded at each census. We included genera represented by ≥ 100 individual trees, as this level of sampling is required to obtain robust estimates of the rate of tree mortality^72^ and maximum tree diameter^73^. The final dataset comprised 333,540 trees, which had been monitored on average for 14.5 years (maximum 55.8 years; Fig. S8), from 463 genera from 81 plant families that comprise 28,177 species (Fig. S9). The median abundance of the genera in the dataset was 491 stems. All the selected genera contain trees, including palms, that occur in tropical lowland wet forest, but the distribution of the genera may also extend to other biomes and include other life forms such as lianas and shrubs. Analyses were also conducted for a subset of 288 genera (comprising 13,364 species) that solely comprise trees in the tropical lowland wet forest biome, and a subset of 107 genera of Amazonian trees.

### Mortality rate and maximum size

We used the plot data to estimate average mortality rates (*m*), calculated from re-measurements of long-term forest plots, as a proxy for variation in generation times^16^. Generation time is a key life-history trait that influences processes linked to speciation and extinction, such as higher rates of molecular evolution^50^. The calculation of mortality rates was based on a maximum likelihood approach based on the survivorship of all individual trees within a genus^74^. The annual probability of mortality for each tree, $i,$ was estimated using a logistic transformation that incorporated the U-shaped impact of tree diameter on mortality:

$$P\left(mortality,i\right)=\frac{1}{1+{e}^{{-k}_{i}}}$$

where

$${k}_{i}=a-{b}_{1}\left({dbh}_{i}\right){e}^{{b}_{2}{dbh}_{i}}$$

These functions were used to calculate the log-likelihood of the dataset of the status of each trees as dead or alive at the end of the period of monitoring for each individual, using Eq. (8) in^74^. For each genus, the parameter estimates that minimised this function were identified using simulated annealing. Finally, the mortality rate for a median diameter tree of each genus was used as the estimate of genus-level mortality rates.

We assume that the broad extent of the plot network with sample plots widely distributed across the wet tropics (Fig. S7), covers the range of species and environmental conditions that are characteristic of each genus, and that the temporal sampling of the plots (Fig. S8) is sufficient to capture the distribution of the return time of events that cause mortality in wet tropical forests. The mean length of monitoring exceeds the typical return time of two to seven years for El Niño/La Niña events^75^ which are a major driver of climatic variation and inter-annual variation in tree mortality in this region^76^. Mean annual mortality rates among genera ranged from 0.1 to 10% a^−1^ and had a similar distribution among biogeographical regions (Fig. S6).

We used data on the maximum diameter of trees within a lineage calculated from the forest plot data as a measure of dispersal potential, as higher stature is associated with longer seed dispersal distances^45^. Maximum size data were calculated as the 95th percentile of the tree diameter across all individuals of each genus^77,78^. Maximum diameter varied from 12 to 200 cm among genera, with similar distributions among continents (Fig. S6).

### Species richness, range size, breeding systems, dispersal syndrome and seed mass

We used the list of accepted, species-level names in the World Checklist of Vascular Plants^79^ to calculate the species richness of each genus. Species richness per genus varied from 1 to > 1000 species (Fig. S6).

We defined range size as the extent of occurrence (EOO; km^2^) for each genus^80^. EOOs were calculated using herbarium records for all species within a given genus downloaded from the Global Biodiversity Information Facility. In total, the calculations used 336,832 records; the mean number of records per genus was 727 (Fig. S5). A greater number of genera from African forests had fewer than 100 records, compared to genera from Asian and American forests (Fig. S5). Records were checked for typos and errors, and latitudinal and longitudinal range limits for each genus based on information in floras were used to remove non-native records (Table S4). EOOs were calculated using alpha hulls, by minimising the value of alpha to encompass 99% of all records, using the *rangeBuilder* package in R^81^ and clipping these distributions to current land area (Fig. S10).

The regional distribution of each genus was classified from the plot data, and categorised as: the Americas (including Amazonia, the Guiana Shield, Atlantic Forest and Central America; n = 151), Africa (including the forests of west Africa, the Congo basin and wet forests areas in eastern Africa; n = 115), Asia (including the Western Ghats in India, Malesia, NE Australia and Pacific islands; n = 78), or present on multiple continents (n = 119).

We used information on the presence of dioecy in a lineage to assess the role of breeding systems in constraining species richness. Lineages with a dioecious breeding system, where male and female flowers are on different trees, are classically considered to have lower opportunities for successful establishment of new populations^29,82^ and therefore lower species richness as they require successful dispersal of both male and female plants^57^. Breeding system was classified for each genus as ‘at least some dioecious species’ or ‘no dioecious species’, based on^83^ and floras (Table S5). The proportion of genera with at least some dioecious species (approximately 27%) was similar across all biogeographical settings (Fig. S6).

Finally, for taxa from Amazonia we also compiled data on seed mass, dispersal mode, mean species-level range size and population size. Seed mass was calculated for all species within each lineage, as this trait is associated with seed dispersal distances^45^. Seed mass for Amazonian taxa (n = 92) was calculated as average values for species within genera^84^; there was insufficient data available to analyse this trait for genera from Africa (n = 8) or Asia (n = 17). The dispersal mode of lineages was classified as wind or non-wind dispersed^35^. Mean species range size and total population size was calculated using estimates for all species within a lineage^34,36^.

### Statistical analysis

The phylogenetic relationships among genera were estimated using a DNA-based, genus-level phylogeny for the tropics, developed from a published genus-level phylogeny for American trees^38,39^. Mortality rate, maximum size, range size, species richness all showed significant phylogenetic signal (mortality rate, Pagel’s λ = 0.56, p < 0.005; range size, λ = 0.24, p < 0.05; maximum size, λ = 0.57, p < 0.005; species richness λ = 0.32, p < 0.05), demonstrating the importance of accounting for shared evolutionary branch lengths in our statistical analysis. Links between the phylogeny and trait values were visualised using the *ggtree* package^85^.

We used species richness as the response variable in our analyses of diversification, rather than estimating diversification rates for each clade as a function of time. This approach is based on previous work for a subset of the lineages analysed here where a ‘density-dependent’ trajectory of diversification provided a closer fit to the observed distribution of clade ages and species richness data for 51 lineages of Amazonian trees, compared to a model where species accumulate at a constant rate^16^. This approach is also consistent with general findings that estimates of diversification rates based on assuming a constant rate of species accumulation perform poorly when predicting changes in species richness of lineages through the fossil record, and that variation in clade age can lead to misleading interpretations of differences in diversification rates^86^. Our approach assumes that the species richness of each lineage has reached a steady-state, and explores which traits determine variation in these steady-state values. We note that clade age is not related to species richness across our data: stem ages of each lineage estimated from the phylogeny vary extremely widely with species richness for these genera (Fig. S11) and therefore the influence of traits on species richness is not confounded by differences in clade age. Overall, our approach is a simplification of the likely wide range of trajectories of change in the number of species within each lineage over geological time. However, in the absence of a detailed fossil record or complete species-level phylogenies for each lineage, this approach represents the only means to gain a macroecological perspective on the traits that influence species richness.

Generalised least squares (GLS) models with phylogenetically correlated errors were used to identify how biogeographical distributions and traits were related to variation in range size across lineages as:

$$log\left(R\right)=\left(log\left(\mu \right)*D\right)+\left(S*D\right)+\left(B*D\right)+\varepsilon$$

where *R* is range size, $\mu$ is mortality rate, *D* is the biogeographic setting of each genus, *S* is maximum size, *B* is a categorical variable denoting either the presence or absence of dioecious species within a genus, and $\varepsilon$ is distributed as ${\sigma }_{\epsilon }^{2}\mathbf{C}$ where $\mathbf{C}$ is the variance–covariance of the error term based on the shared branch lengths of the phylogeny.

Variation in species richness was analysed similarly as:

$$log\left(Species\:richness\right)=\left(log\left(R\right)*D\right)+\left(log\left(\mu \right)*D\right)+\left(S*D\right)+\left(B*D\right)+ \varepsilon$$

GLS analysis can only test for direct, independent effects of each predictor variable on the response variables. We therefore also used piece-wise structural equation modelling (pSEM) to explore the network of both direct and indirect relationships among these variables^33,87^.

We hypothesised that the species richness of genera would be influenced by the range size of a lineage, following studies that demonstrate this link for animals and plants^23,24^ and expectations from allopatric models of speciation that assume that range expansion precedes genetic divergence^21^. We allowed traits to either influence range size and/or species richness directly, as traits may influence rates of range expansion and contraction and/or other processes that underpin speciation, such as the transition time to genetic divergence following the establishment of a lineage^21^. Initial pSEM analysis indicated that there were also significant relationships among maximum size and mortality rates within these data, consistent with findings that demographic and structural traits are linked among lineages of tropical trees^88,89^. We therefore also allowed variation in maximum size and breeding system to be associated with variation in mortality rates in the final pSEM models. For each biogeographical setting, structural equation models based on these networks including only the statistically significant relationships showed a good fit to the data, indicating that significant relationships among the variables were not being excluded (Fig. 1; Americas, Fisher’s C = 6.5, ns; Africa: Fisher’s C = 4.9, ns; Asia, Fisher’s C = 5.8, ns; Multiple continents, Fisher’s C = 5.9, ns; core dataset, all biogeographical settings; pSEM, C = 14.7, ns).

For the analyses focussing on Amazonia, we extended these analyses to include total population size, mean species range size, seed mass and dispersal mode, using a similar statistical approach. For Amazonian taxa, we used GLS to test how traits were related to variation in mean species range size and how traits, mean species range size and total population size were associated with species richness. We included a quadratic term to model the relationship between mean species range size and species richness, based on preliminary exploration of the relationships within the data. Similarly, we also constructed pSEMs to understand the network of direct and indirect relationships among these variables. We explored whether traits determined variation in mean species range size, and how mean species range size is related to species richness. We included total population size as an additional predictor of species richness; correlated errors were permitted between mean species range size and population size. The structural equation model based on this network including only the statistically significant relationships showed a good fit to the data (Fisher’s C = 15.8, ns).

## Supplementary Information

Supplementary Information 1.

Supplementary Information 2.

Supplementary Information 3.

Supplementary Information 4.

Supplementary Information 5.

Supplementary Information 6.

Supplementary Information 7.

Supplementary Information 8.

Supplementary Information 9.

Supplementary Information 10.

Supplementary Information 11.

Supplementary Information 12.

## Data Availability

All metadata used in the analyses is available as supplementary information and as a Forestplots.net data package from https://doi.org/10.5521/forestplots.net/2025_1
