# Supplementary material for: Large range sizes link fast life histories with high species richness across wet tropical tree floras
Source: Sci Rep. 2025 Feb 8;15:4695. doi: 10.1038/s41598-024-84367-3 (PMC11807110; doi:10.1038/s41598-024-84367-3)
Supplement: Supplementary file 1 — Supplementary Information 1. [file 41598_2024_84367_MOESM1_ESM.pdf]

**Abarema**

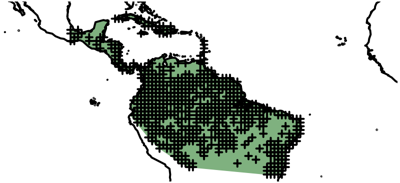

**Adelia**

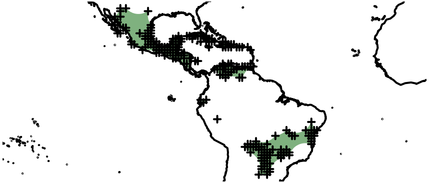

**Adinandra**

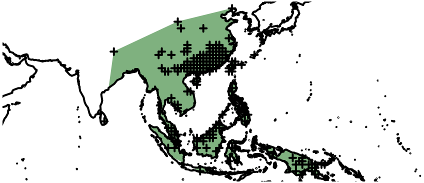

**Afrostyrax**

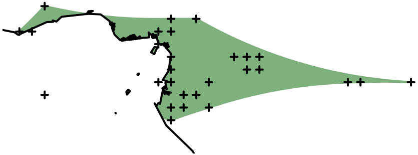

**Afzelia**

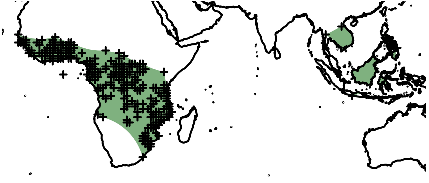

**Aglaia**

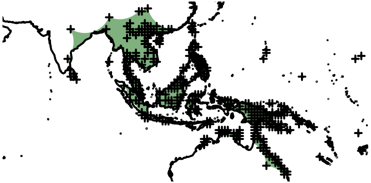

**Aidia**

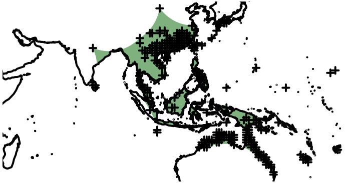

**Aidia**

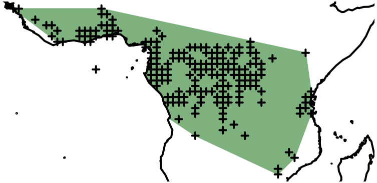

**Alangium**

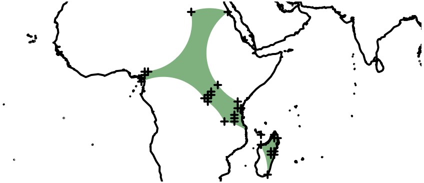

**Alangium**

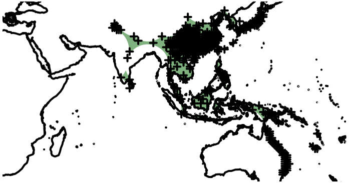

**Albizia**

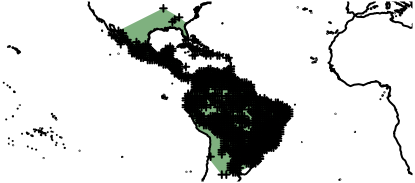

**Albizia**

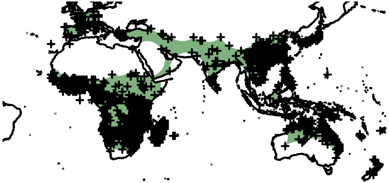

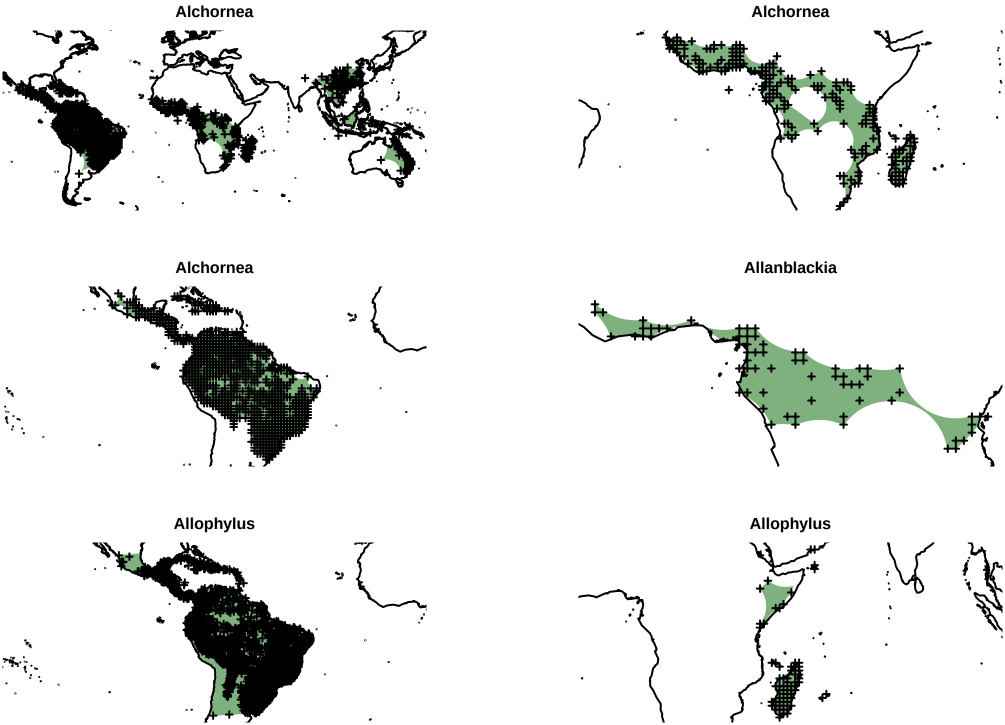
Alchornea
Alchornea
Alchornea
Allanblackia
Allophylus
Allophylus

**Allophylus**

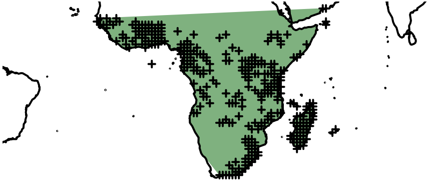

**Alphonsea**

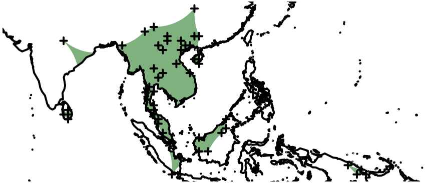

**Alseis**

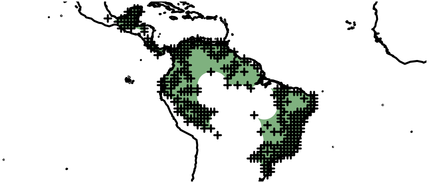

**Alseodaphne**

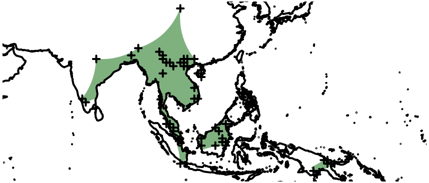

**Alstonia**

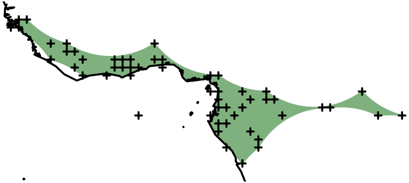

**Alstonia**

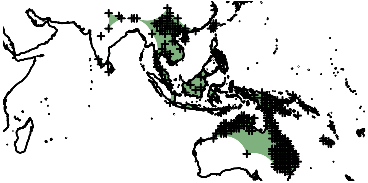

**Alstonia**

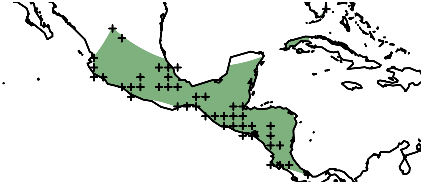

**Amaioua**

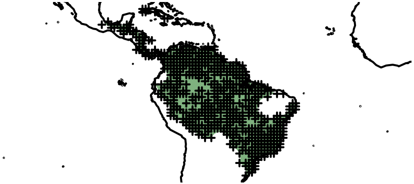

**Amanoa**

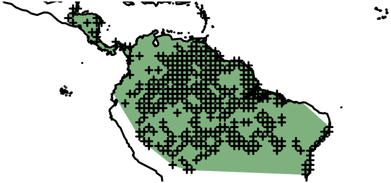

**Amanoa**

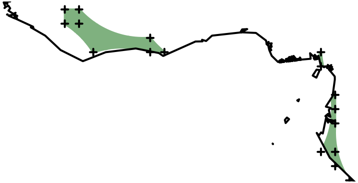

**Ambelania**

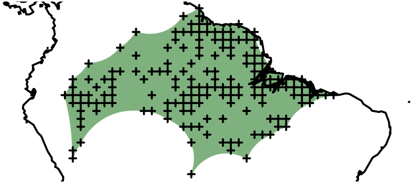

**Ampelocera**

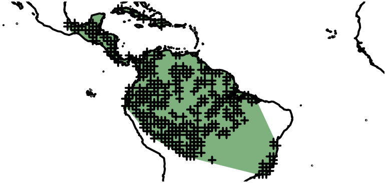

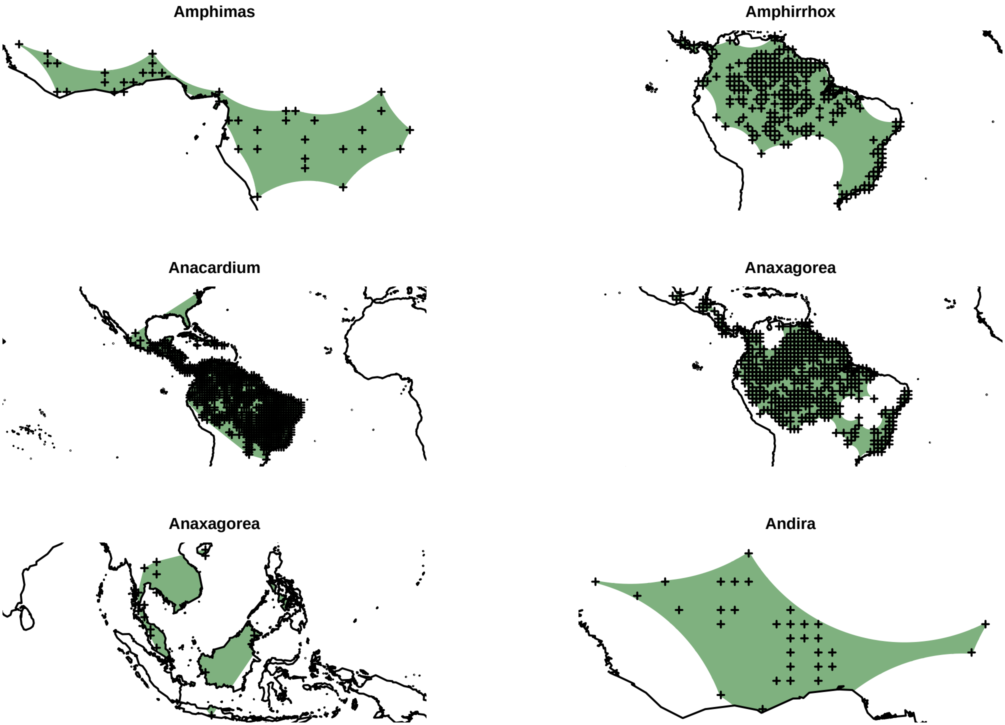
Amphimas
Amphirrhox
Anacardium
Anaxagorea
Anaxagorea
Andira

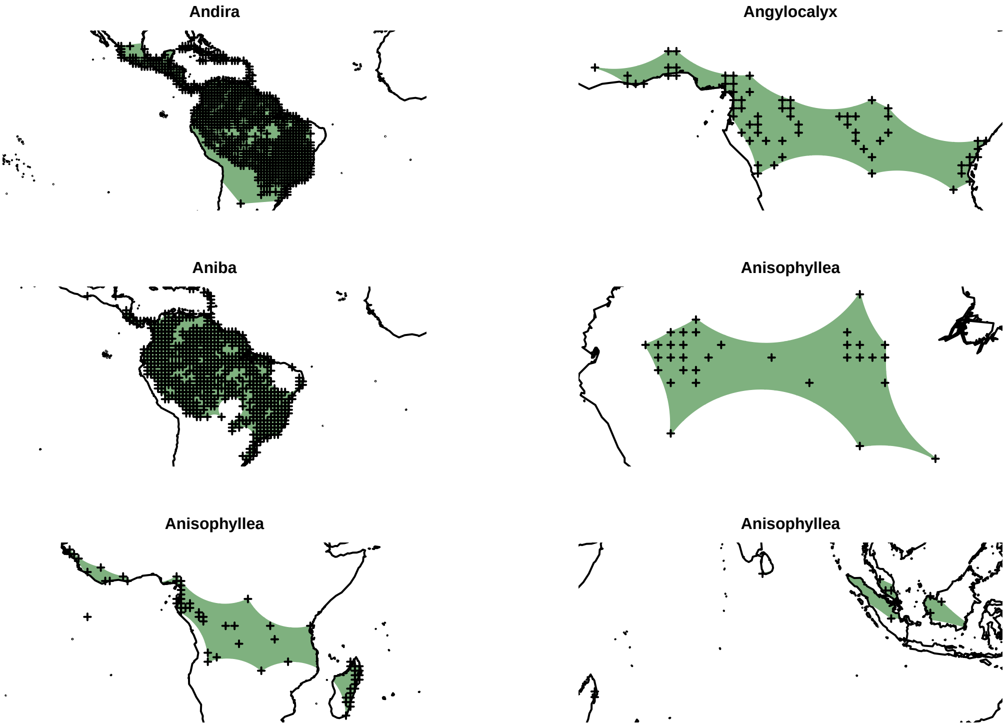
Andira
Angylocalyx
Aniba
Anisophyllea
Anisophyllea
Anisophyllea

**Anisoptera**

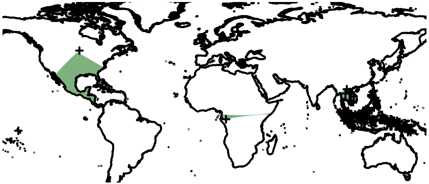

**Annickia**

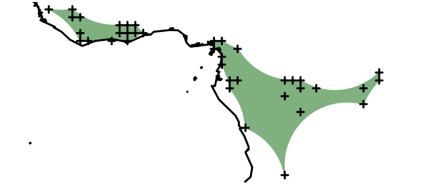

**Annona**

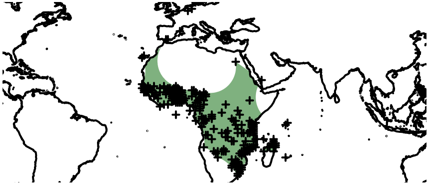

**Annona**

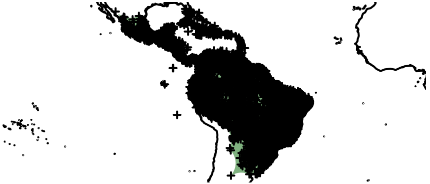

**Anonidium**

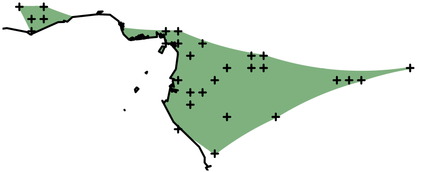

**Anopyxis**

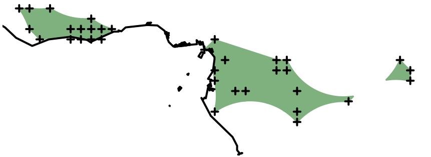

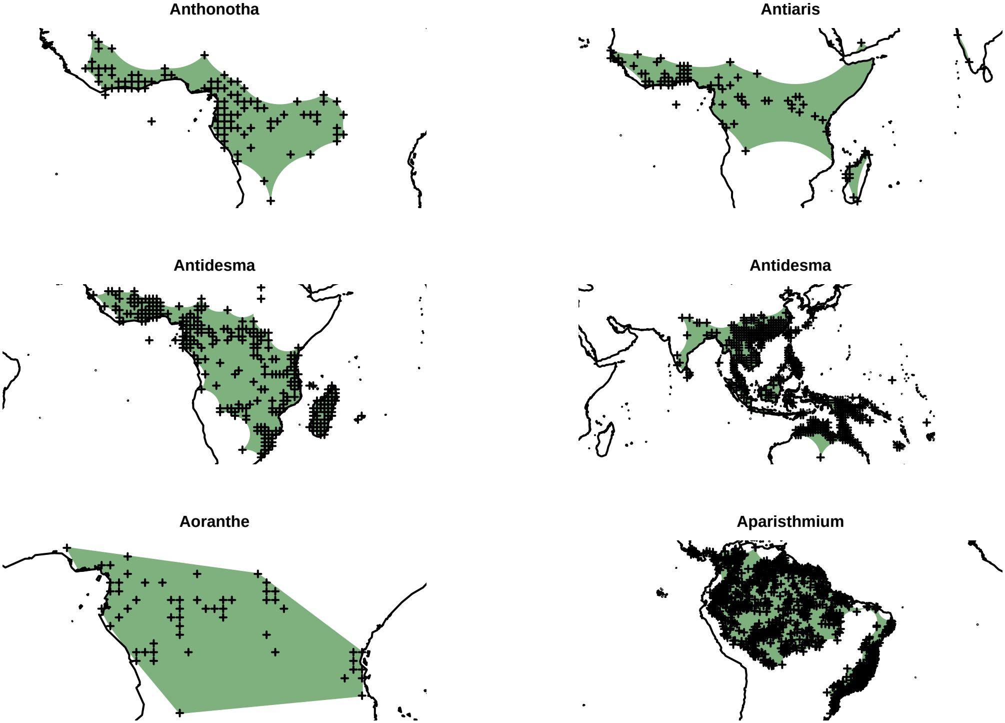
Anthonotha
Antiaris
Antidesma
Antidesma
Aoranthe
Aparisthmium

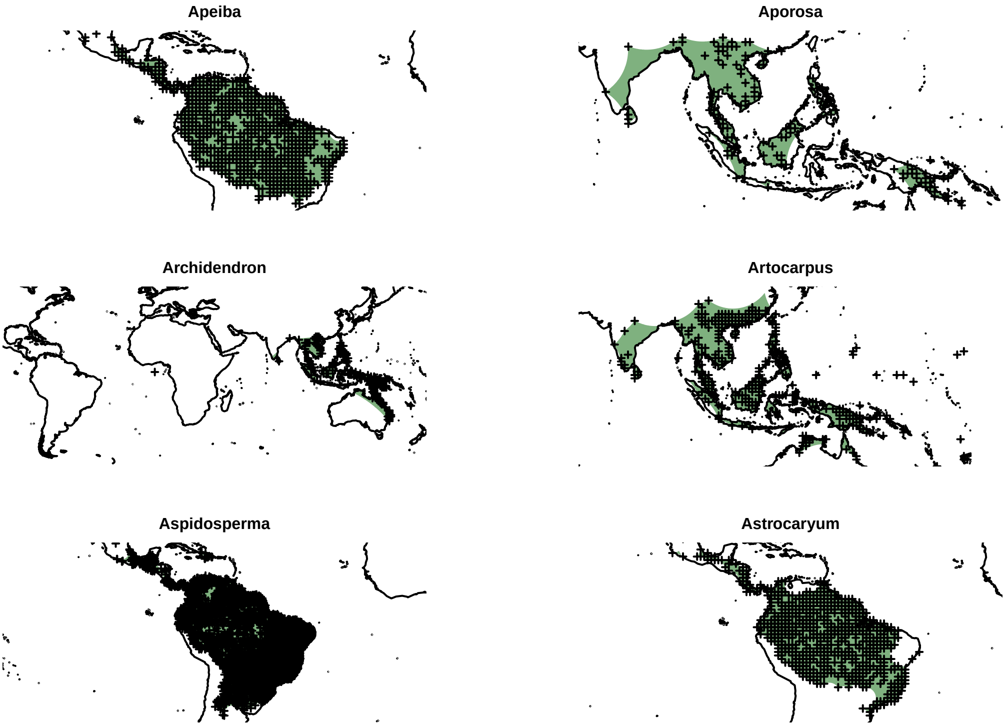
Apeiba
Aporosa
Archidendron
Artocarpus
Aspidosperma
Astrocaryum
