# Supplementary material for: Large range sizes link fast life histories with high species richness across wet tropical tree floras
Source: Sci Rep. 2025 Feb 8;15:4695. doi: 10.1038/s41598-024-84367-3 (PMC11807110; doi:10.1038/s41598-024-84367-3)
Supplement: Supplementary file 2 — Supplementary Information 2. [file 41598_2024_84367_MOESM2_ESM.pdf]

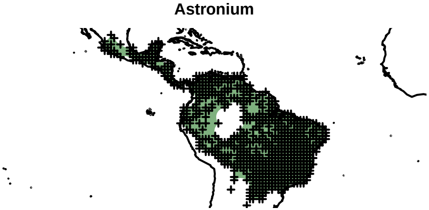
Astronium

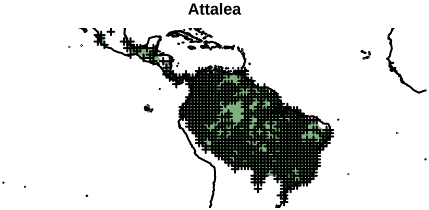
Attalea

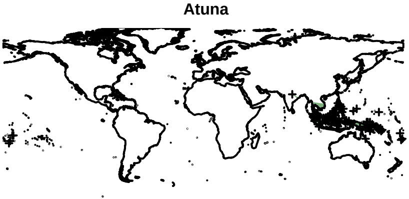
Atuna

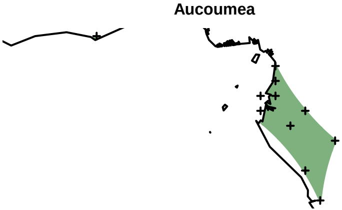
Aucoumea

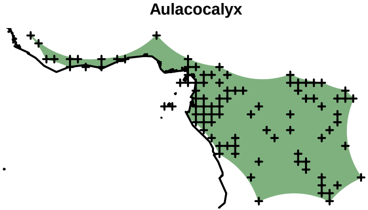
Aulacocalyx

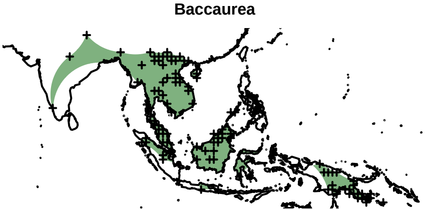
Baccaurea

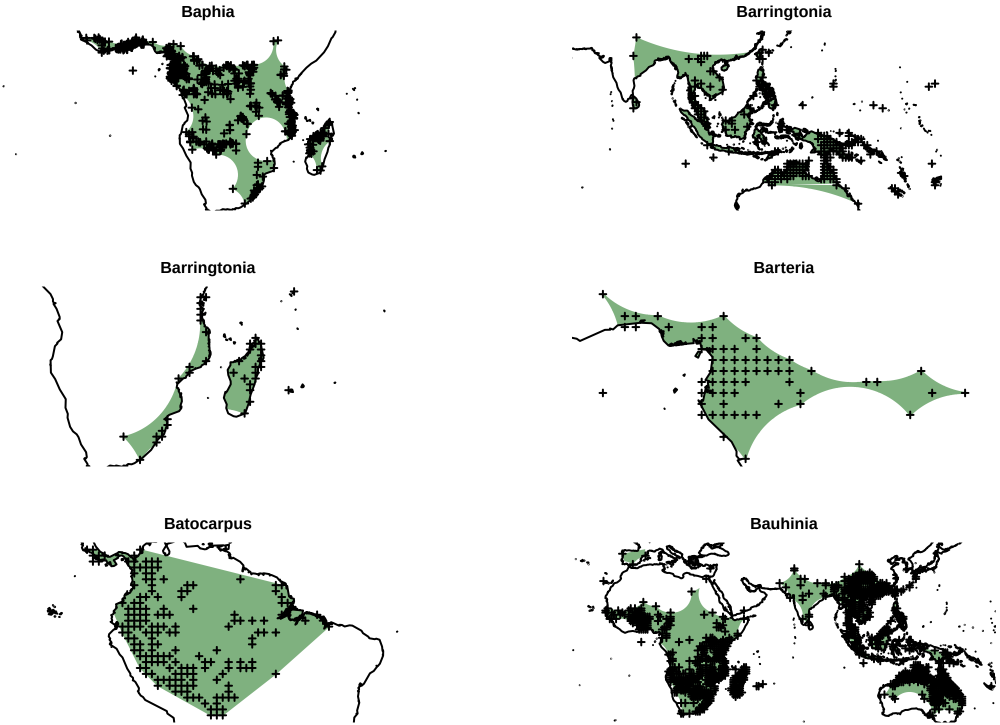
Baphia
Barringtonia
Barringtonia
Barteria
Batocarpus
Bauhinia

Bauhinia

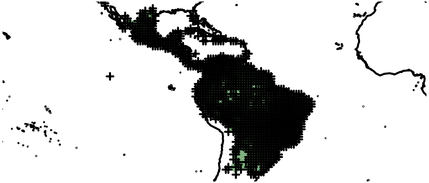

Beilschmiedia

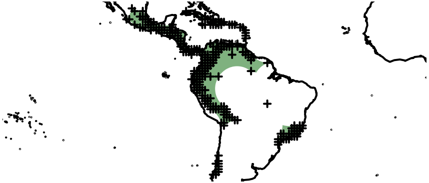

Beilschmiedia

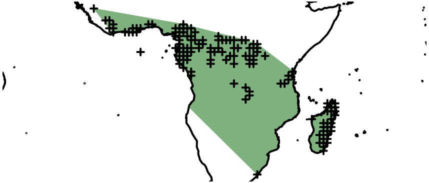

Beilschmiedia

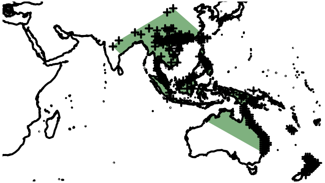

Berlinia

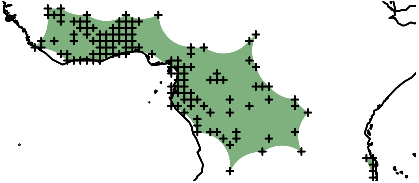

Bhesa

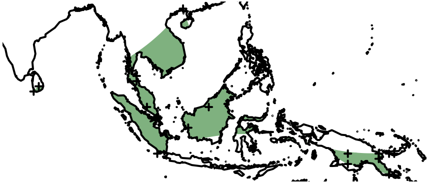

Bixa

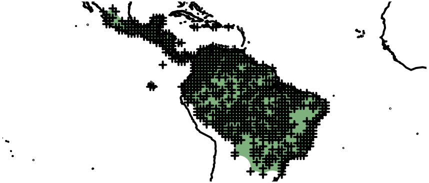

Blighia

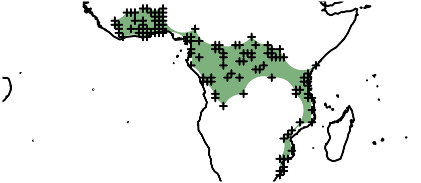

Blumeodendron

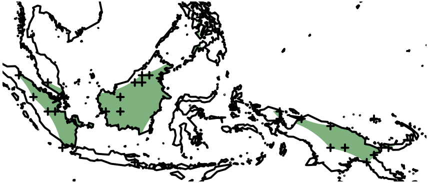

Bocageopsis

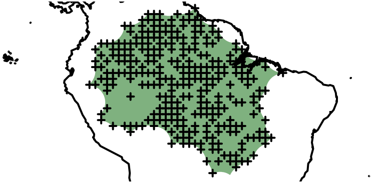

Bocoa

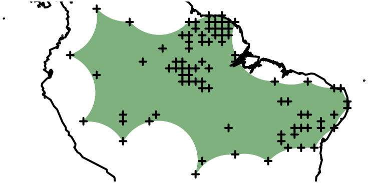

Brachystegia

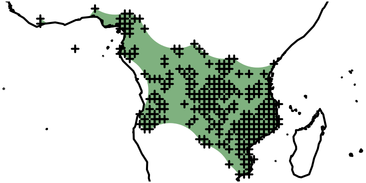

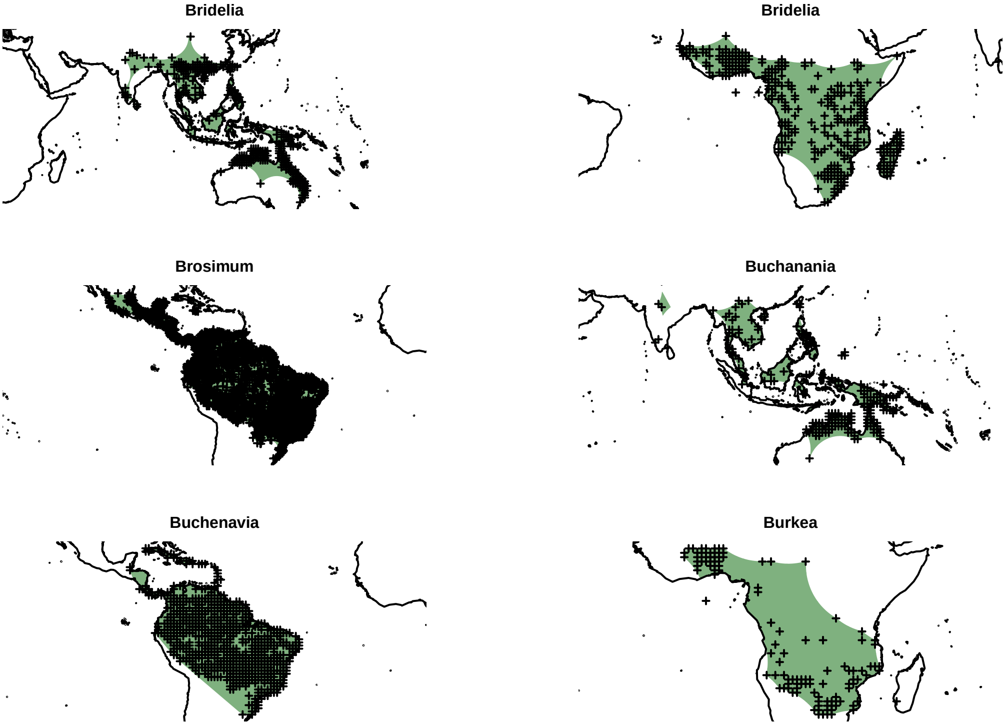
Bridelia
Bridelia
Brosimum
Buchanania
Buchenavia
Burkea

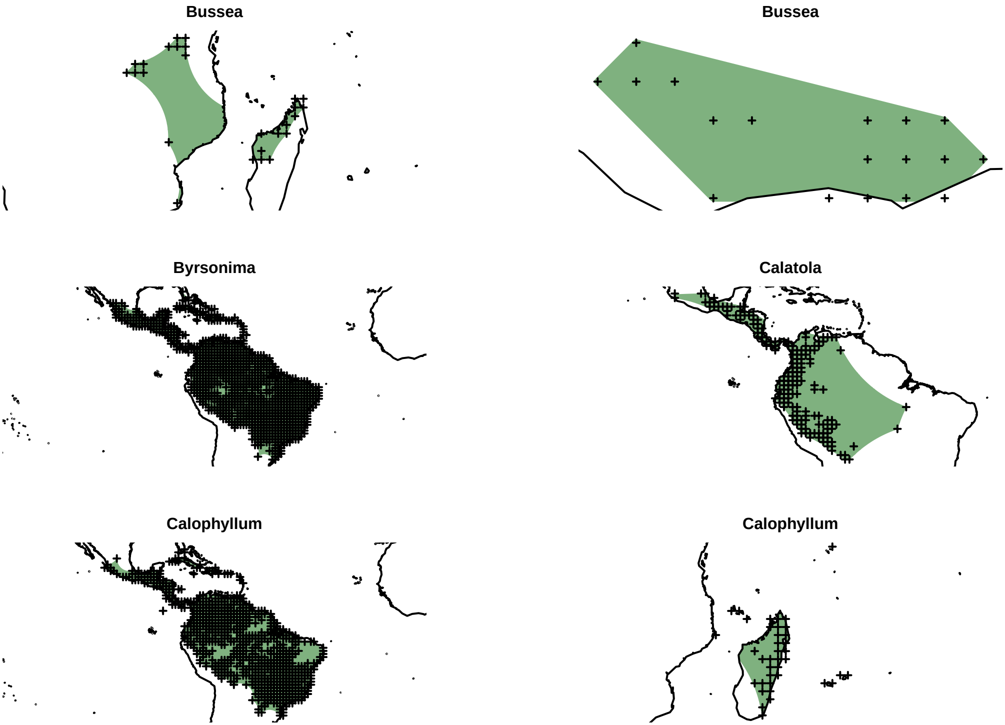
Bussea
Bussea
Byrsonima
Calatola
Calophyllum
Calophyllum

Calophyllum

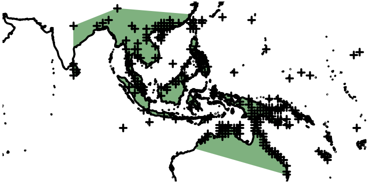

Calpocalyx

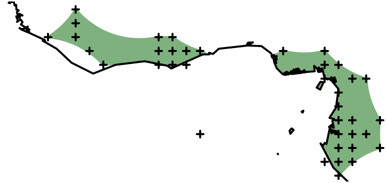

Calyptranthes

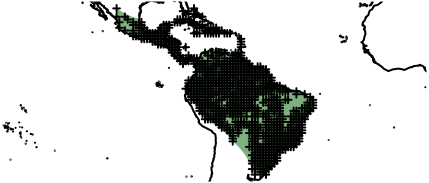

Canarium

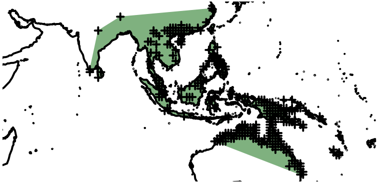

Canarium

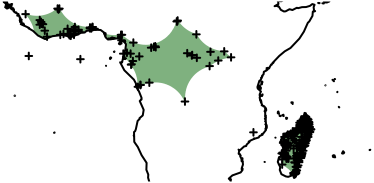

Canthium

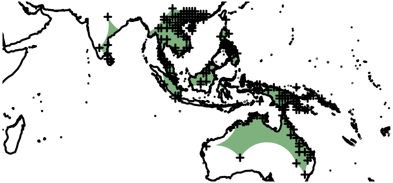

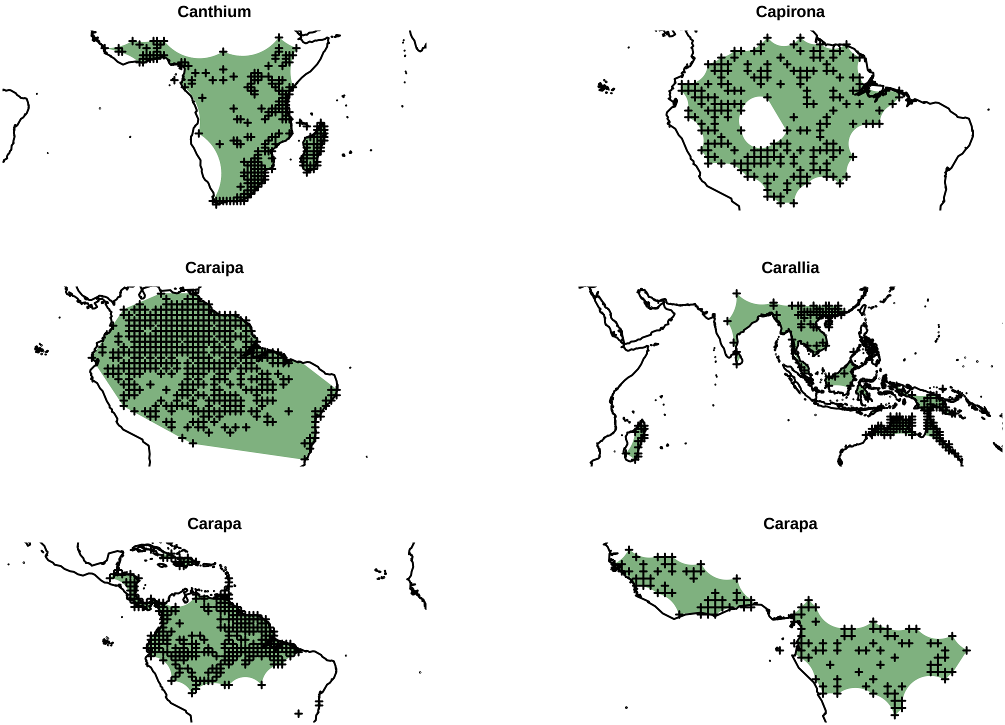
Canthium
Capirona
Caraipa
Carallia
Carapa
Carapa

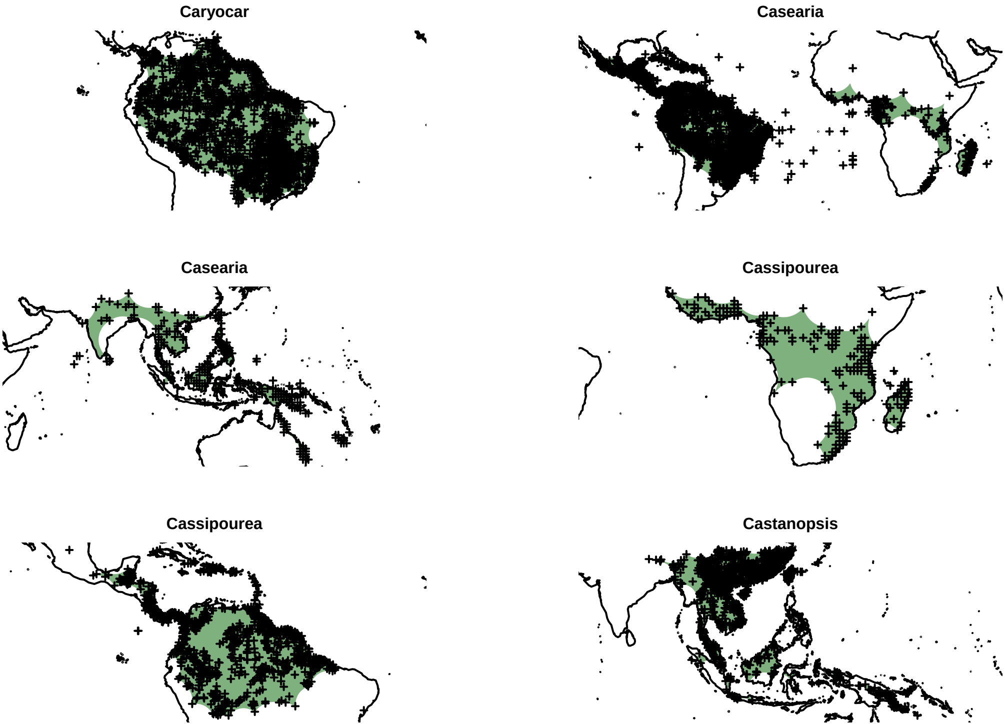
Caryocar
Casearia
Casearia
Cassipourea
Cassipourea
Castanopsis

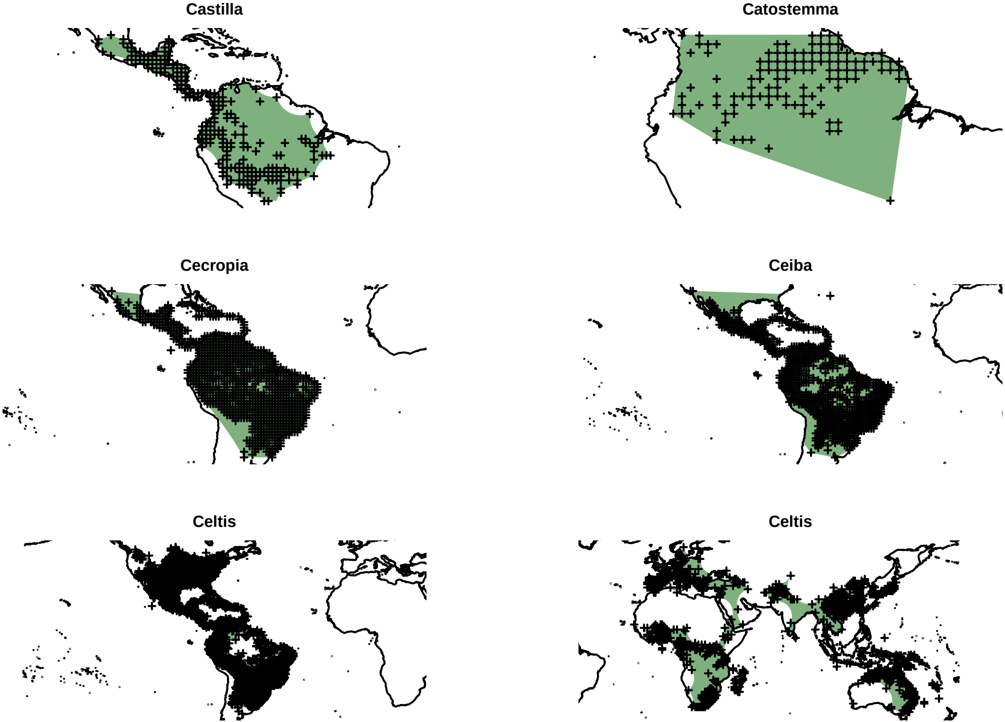
Castilla
Catostemma
Cecropia
Ceiba
Celtis
Celtis
