# Supplementary material for: Large range sizes link fast life histories with high species richness across wet tropical tree floras
Source: Sci Rep. 2025 Feb 8;15:4695. doi: 10.1038/s41598-024-84367-3 (PMC11807110; doi:10.1038/s41598-024-84367-3)
Supplement: Supplementary file 3 — Supplementary Information 3. [file 41598_2024_84367_MOESM3_ESM.pdf]

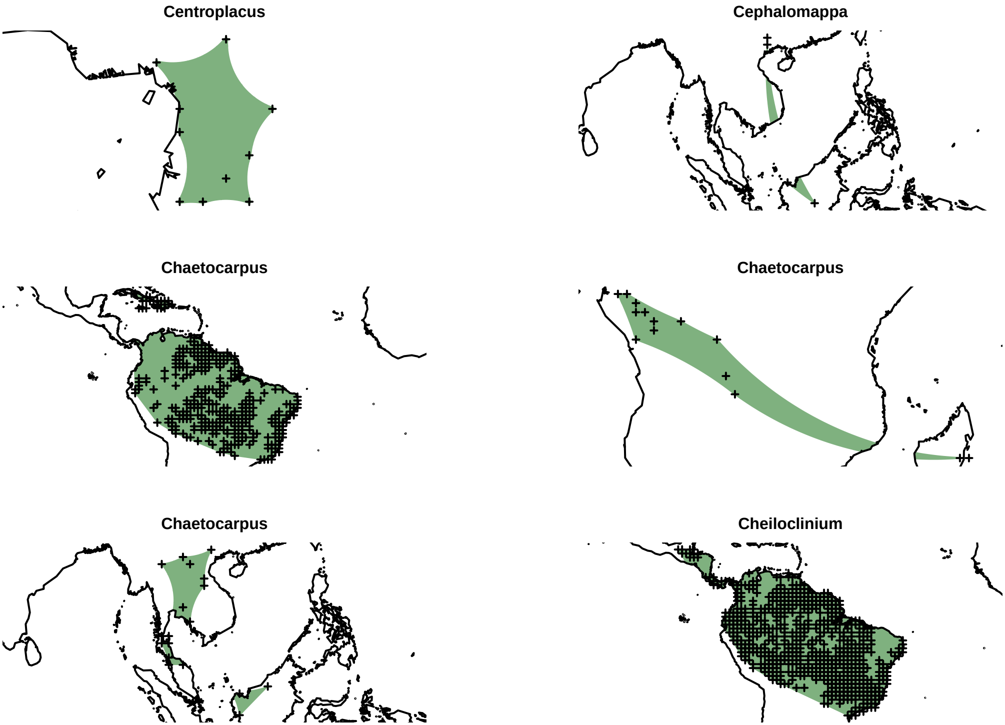

Centroplacus
Cephalomappa
Chaetocarpus
Chaetocarpus
Chaetocarpus
Cheiloclinium

Chimarrhis

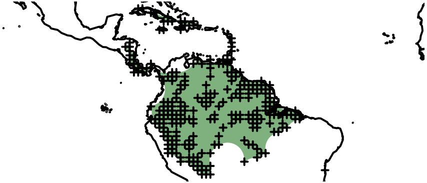

Chionanthus

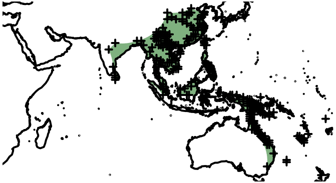

Chionanthus

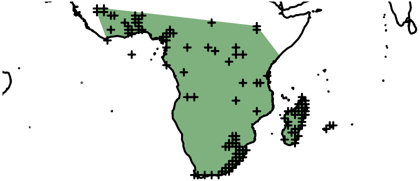

Chionanthus

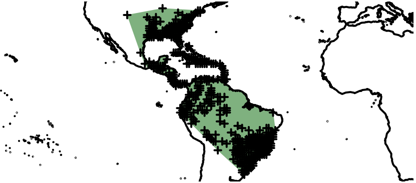

Chisocheton

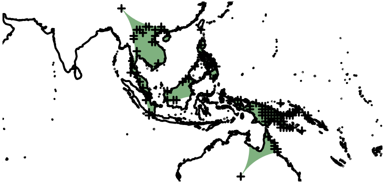

Chlorocardium

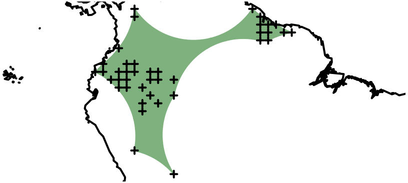

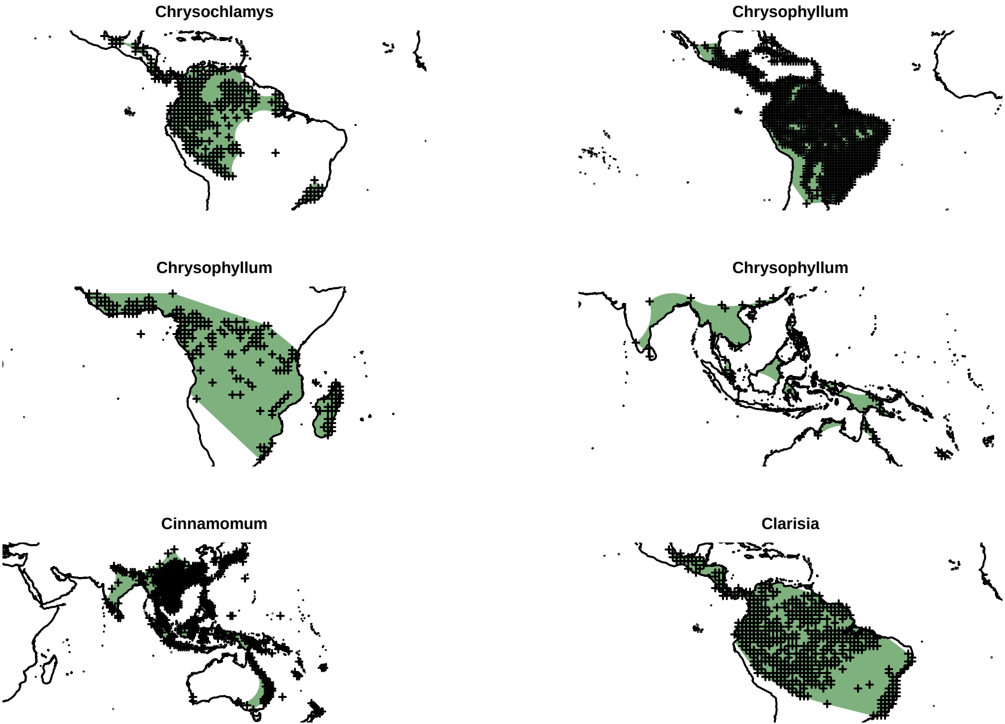
Chrysochlamys
Chrysophyllum
Chrysophyllum
Chrysophyllum
Cinnamomum
Clarisia

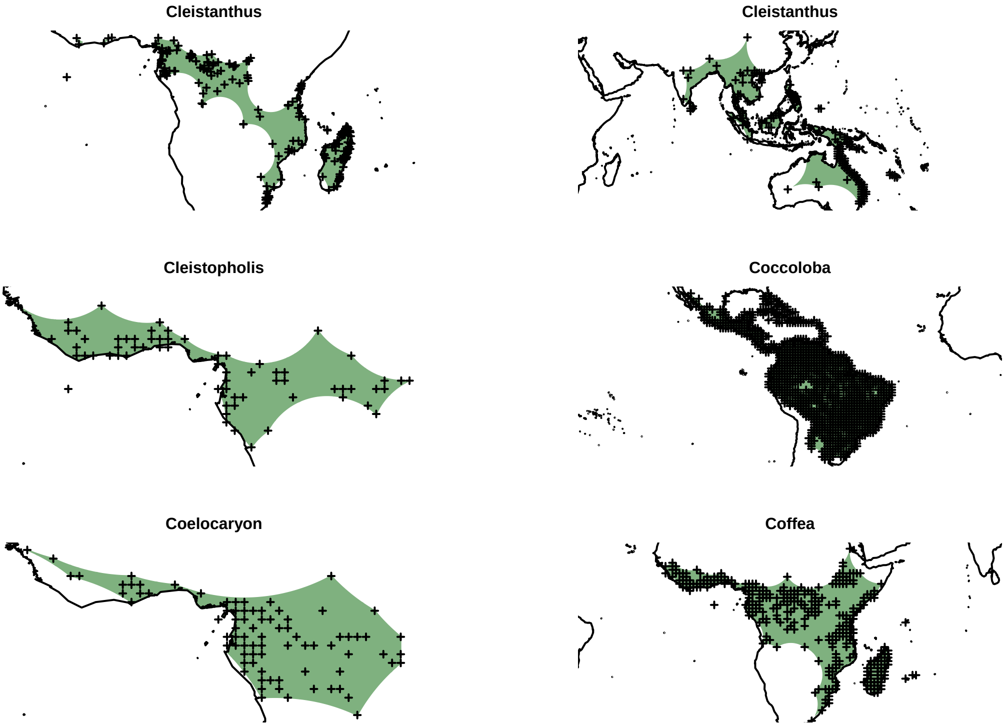
Cleistanthus
Cleistanthus
Cleistopholis
Coccoloba
Coelocaryon
Coffea

Coffea

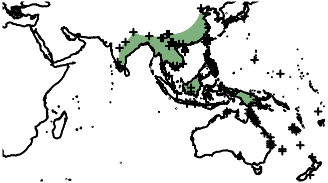

Cola

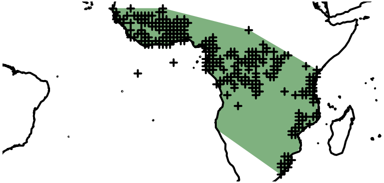

Colubrina

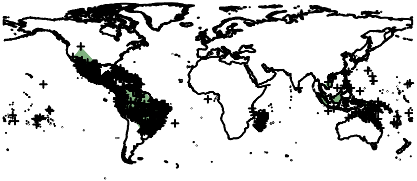

Combretum

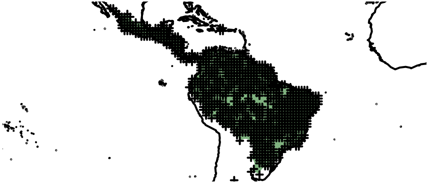

Combretum

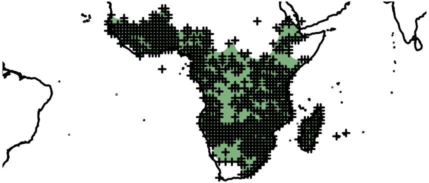

Combretum

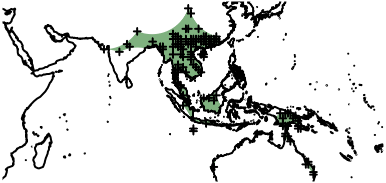

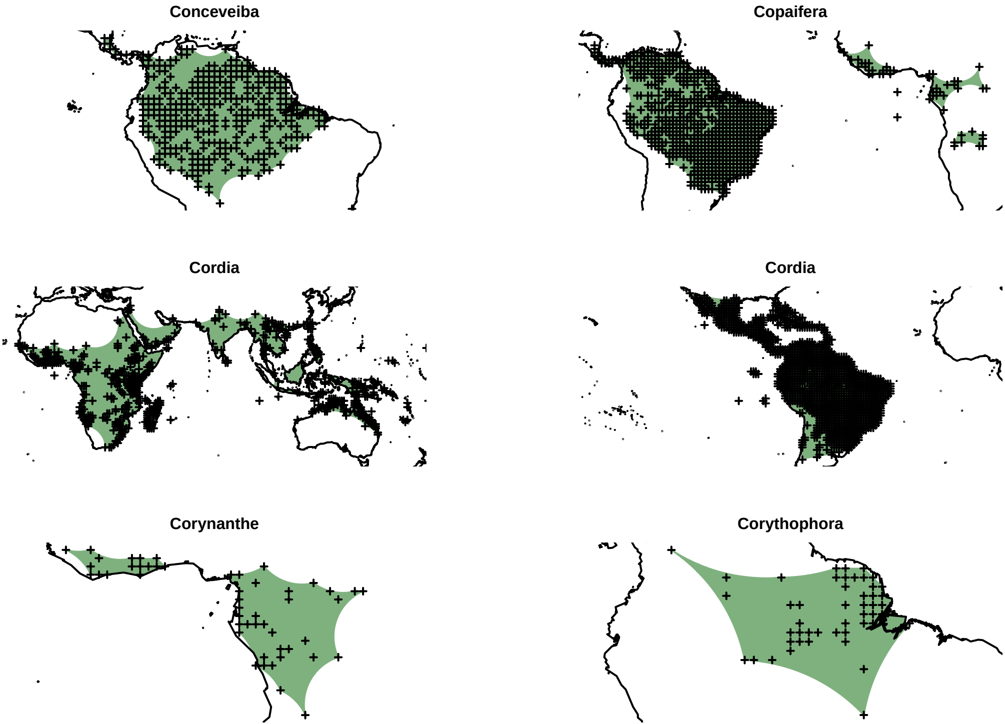
Conceveiba
Copaifera
Cordia
Cordia
Corynanthe
Corythophora

Couepia

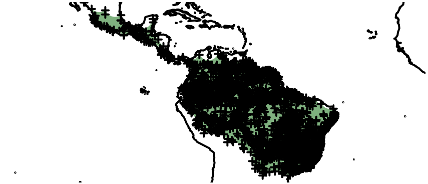

Coula

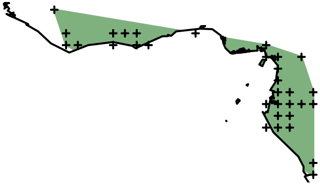

Couratari

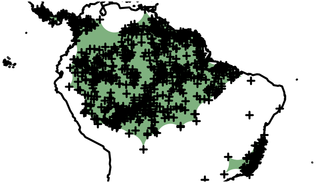

Cratoxylum

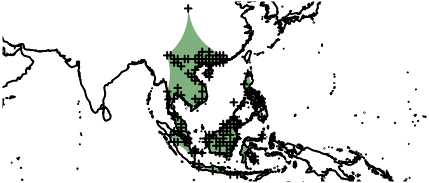

Crossopteryx

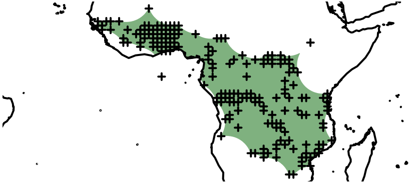

Croton

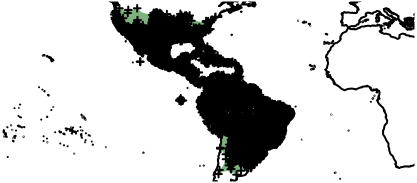

Croton

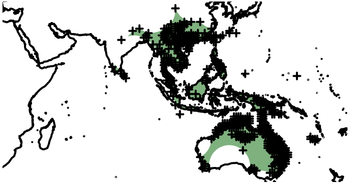

Croton

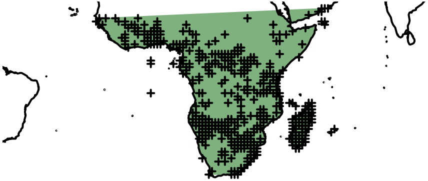

Crudia

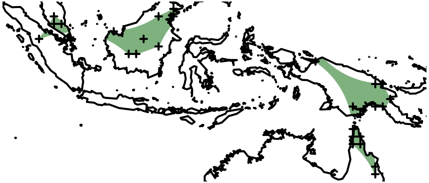

Crudia

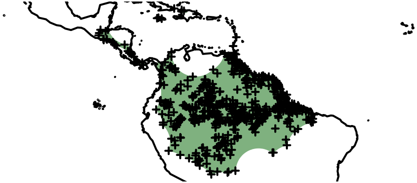

Crudia

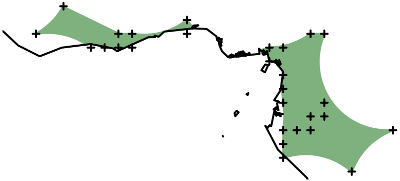

Cryptocarya

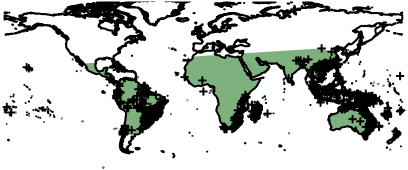

Ctenolophon

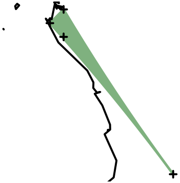

Ctenolophon

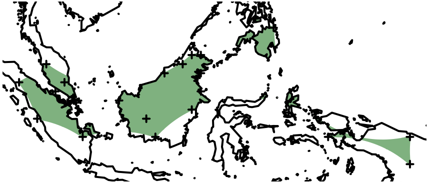

Cupania

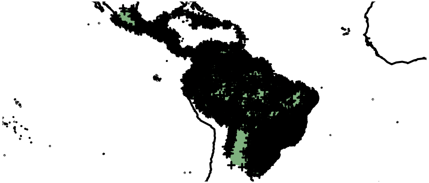

Cyathocalyx

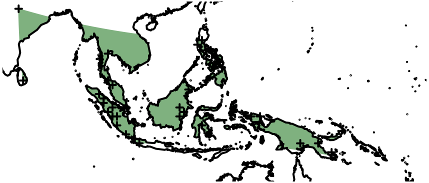

Cylicodiscus

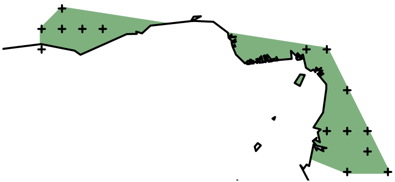

Cynometra

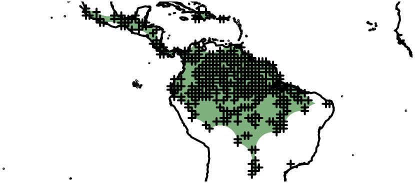

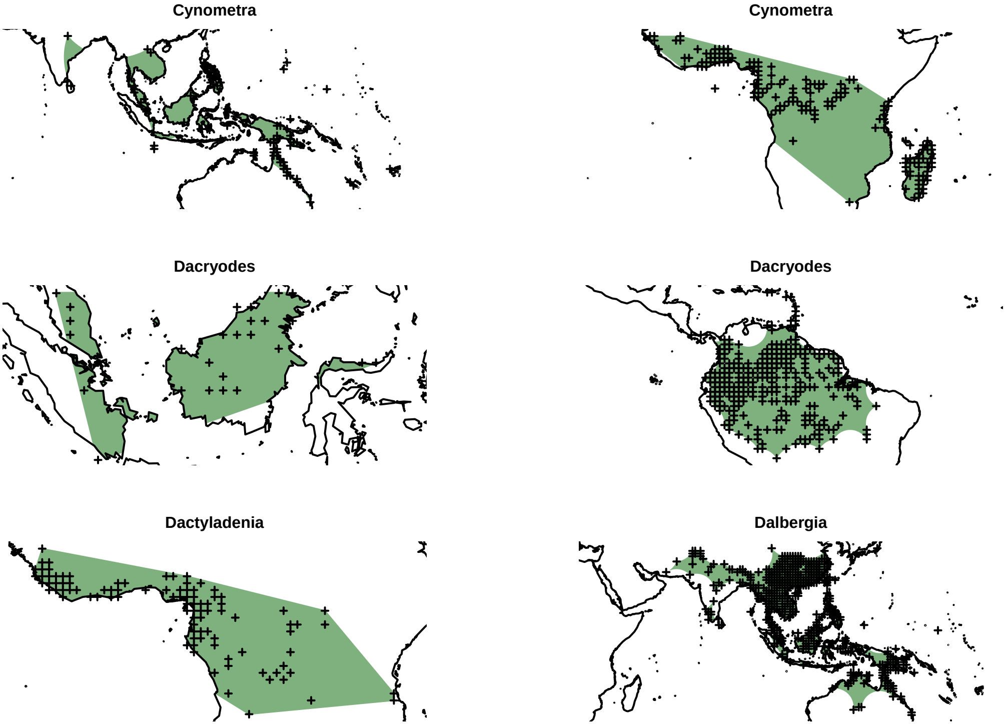
Cynometra
Cynometra
Dacryodes
Dacryodes
Dactyladenia
Dalbergia
