# Supplementary material for: Large range sizes link fast life histories with high species richness across wet tropical tree floras
Source: Sci Rep. 2025 Feb 8;15:4695. doi: 10.1038/s41598-024-84367-3 (PMC11807110; doi:10.1038/s41598-024-84367-3)
Supplement: Supplementary file 4 — Supplementary Information 4. [file 41598_2024_84367_MOESM4_ESM.pdf]

Dalbergia

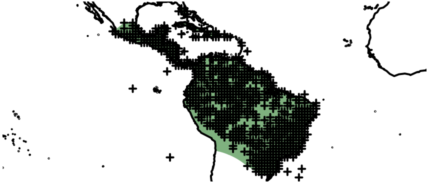

Dalbergia

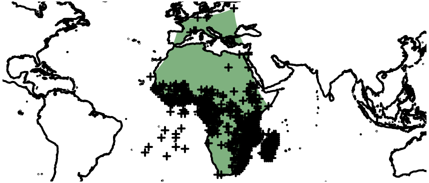

Daniellia

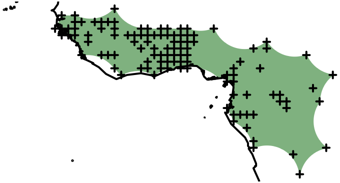

Dasylepis

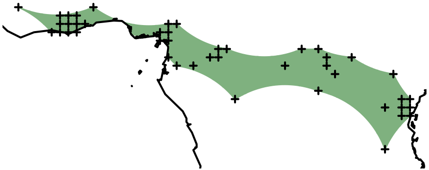

Dehaasia

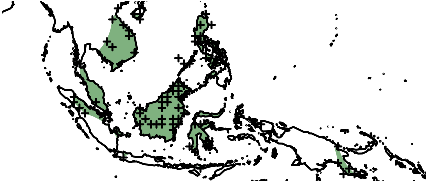

Dendrobangia

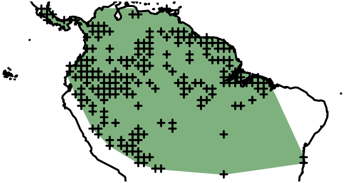

Dendropanax

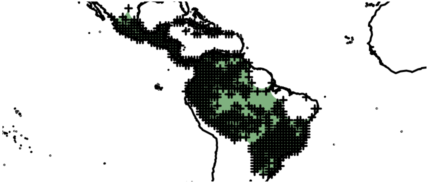

Dendropanax

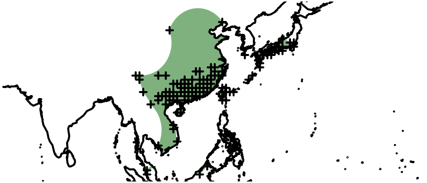

Desbordesia

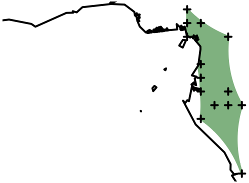

Desplatsia

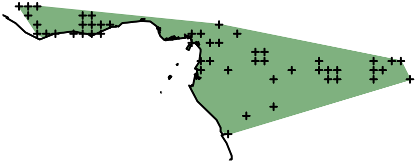

Dialium

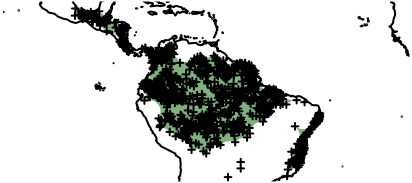

Dialium

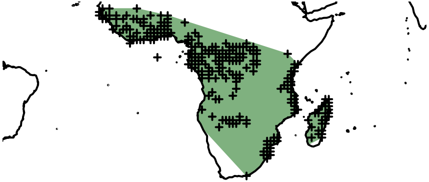

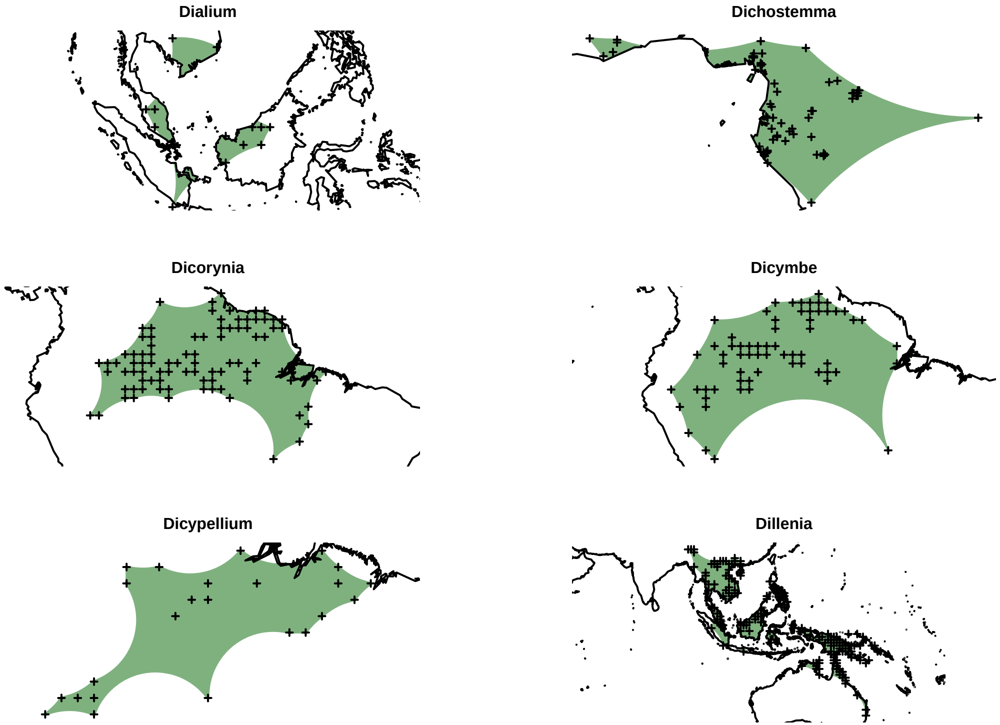
Dialium
Dichostemma
Dicorynia
Dicymbe
Dicypellium
Dillenia

Diogoa

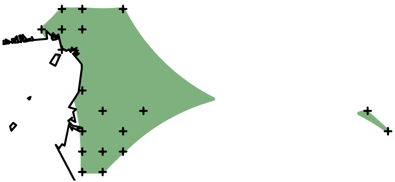

Diospyros

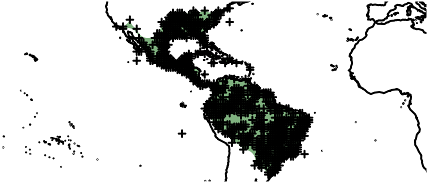

Diospyros

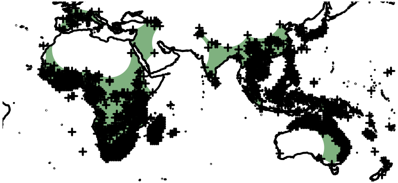

Diplorhynchus

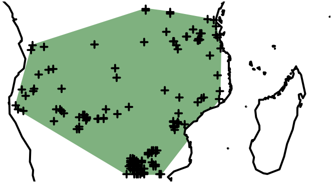

Diplospora

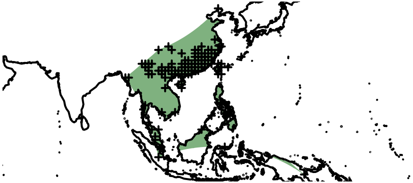

Diplotropis

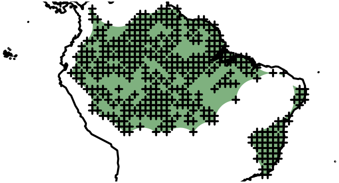

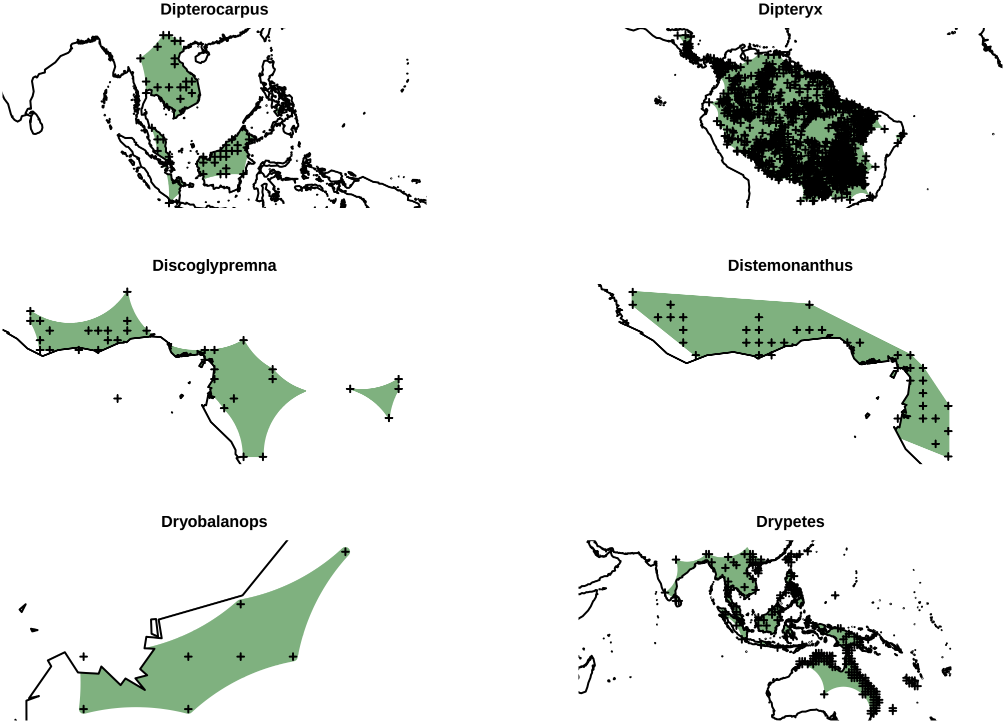
Dipterocarpus
Dipteryx
Discoglypremna
Distemonanthus
Dryobalanops
Drypetes

Drypetes

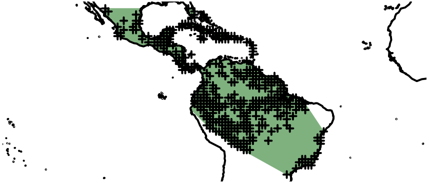

Drypetes

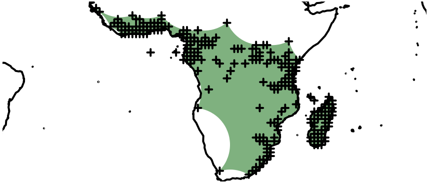

Duguetia

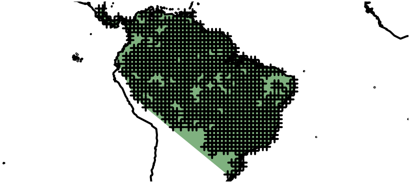

Duguetia

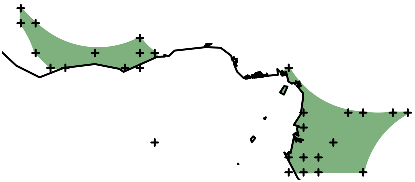

Durio

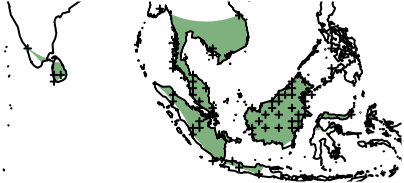

Duroia

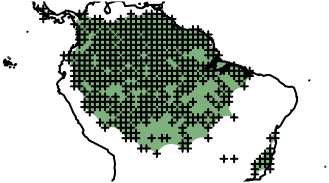

Dyera

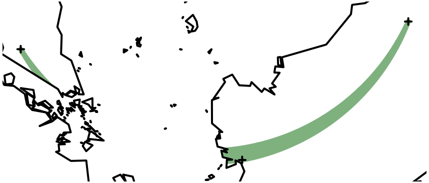

Dysoxylum

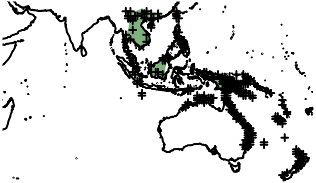

Ecclinusa

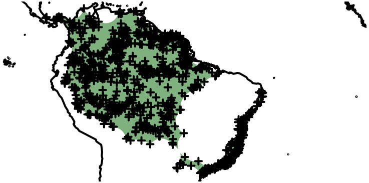

Elaeocarpus

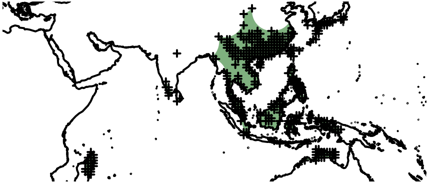

Elaeocarpus

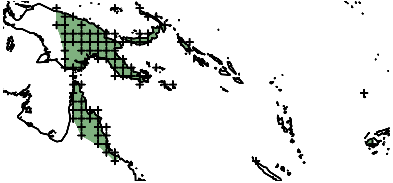

Elateriospermum

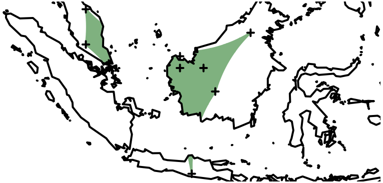

Endlicheria

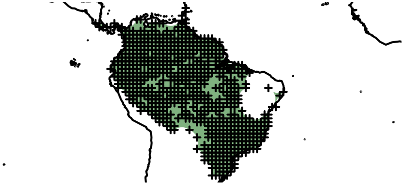

Endopleura

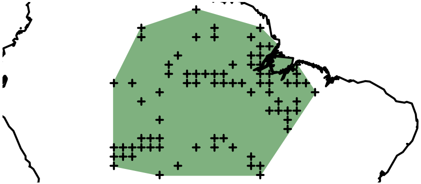

Englerophytum

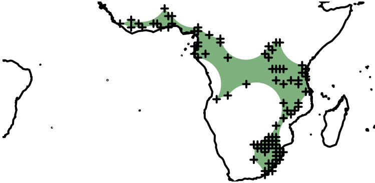

Enicosanthum

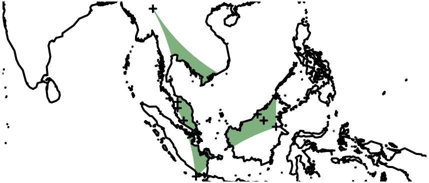

Entandrophragma

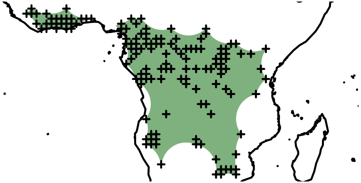

Eperua

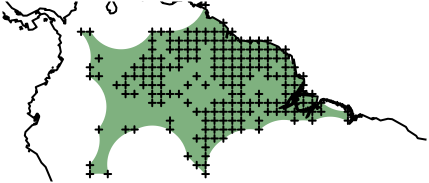

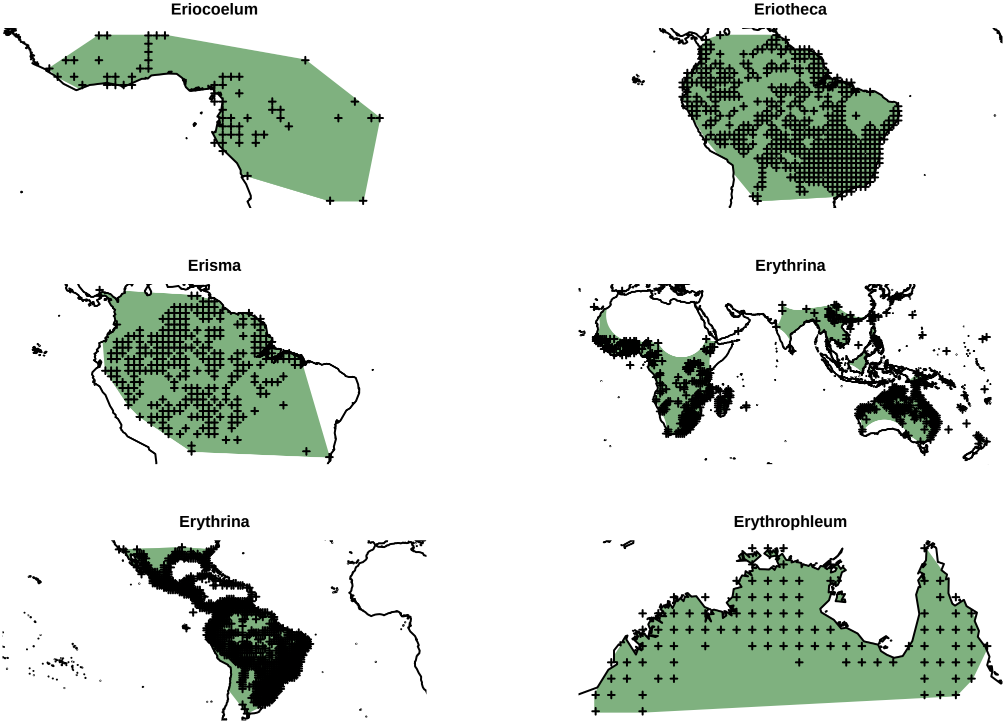
Eriocoelum
Eriotheca
Erisma
Erythrina
Erythrina
Erythrophleum

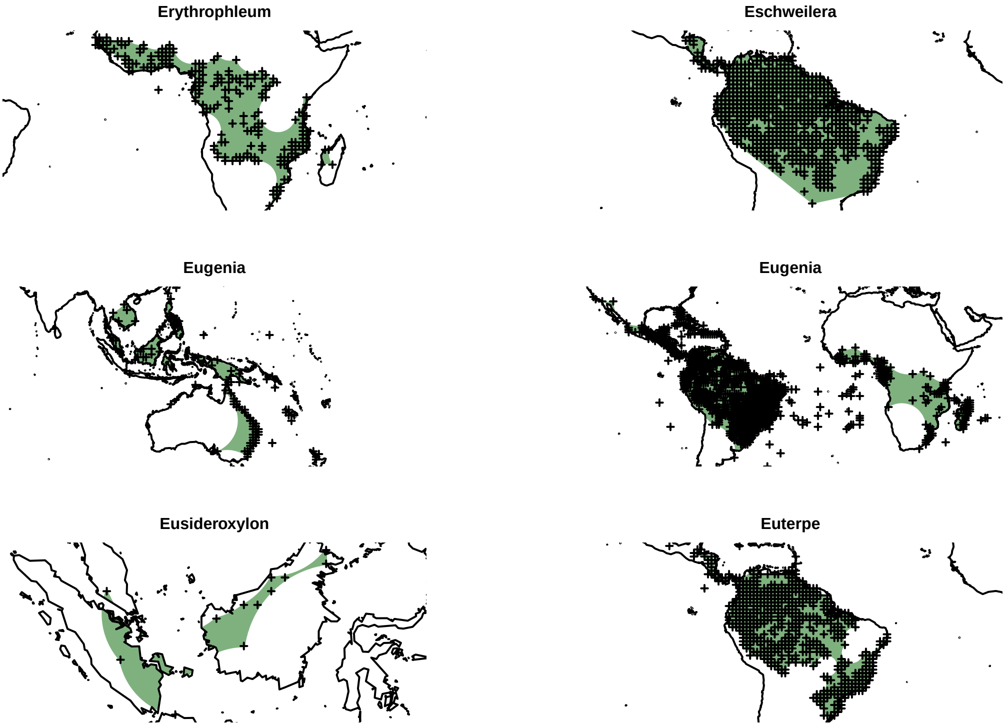
Erythrophleum
Eschweilera
Eugenia
Eugenia
Eusideroxylon
Euterpe
