# Supplementary material for: Large range sizes link fast life histories with high species richness across wet tropical tree floras
Source: Sci Rep. 2025 Feb 8;15:4695. doi: 10.1038/s41598-024-84367-3 (PMC11807110; doi:10.1038/s41598-024-84367-3)
Supplement: Supplementary file 5 — Supplementary Information 5. [file 41598_2024_84367_MOESM5_ESM.pdf]

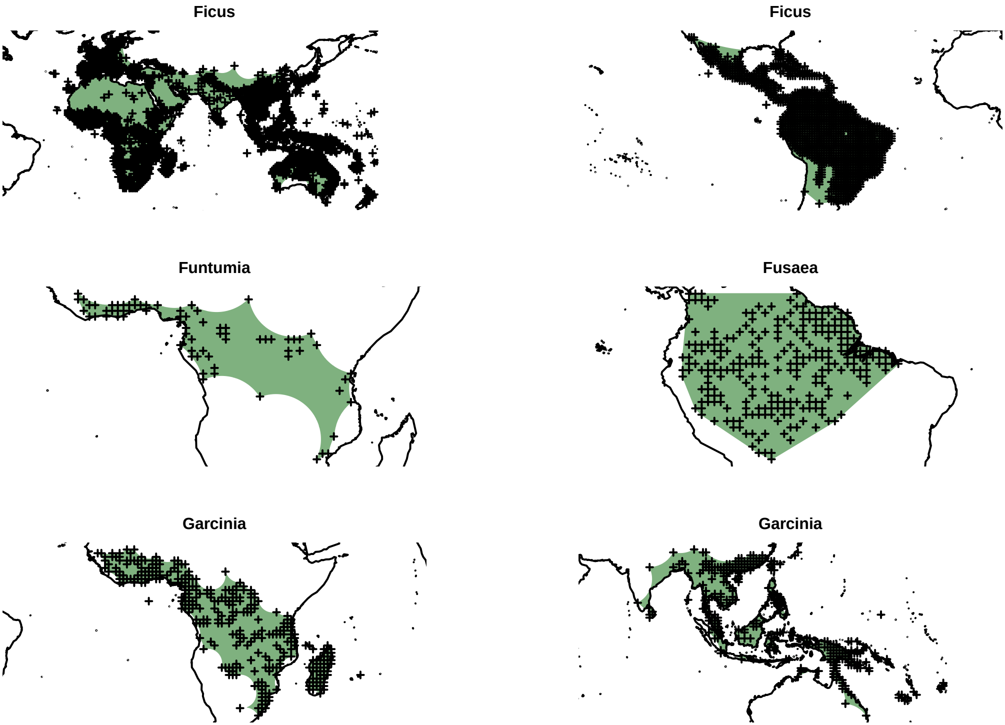
Ficus
Ficus
Funtumia
Fusaea
Garcinia
Garcinia

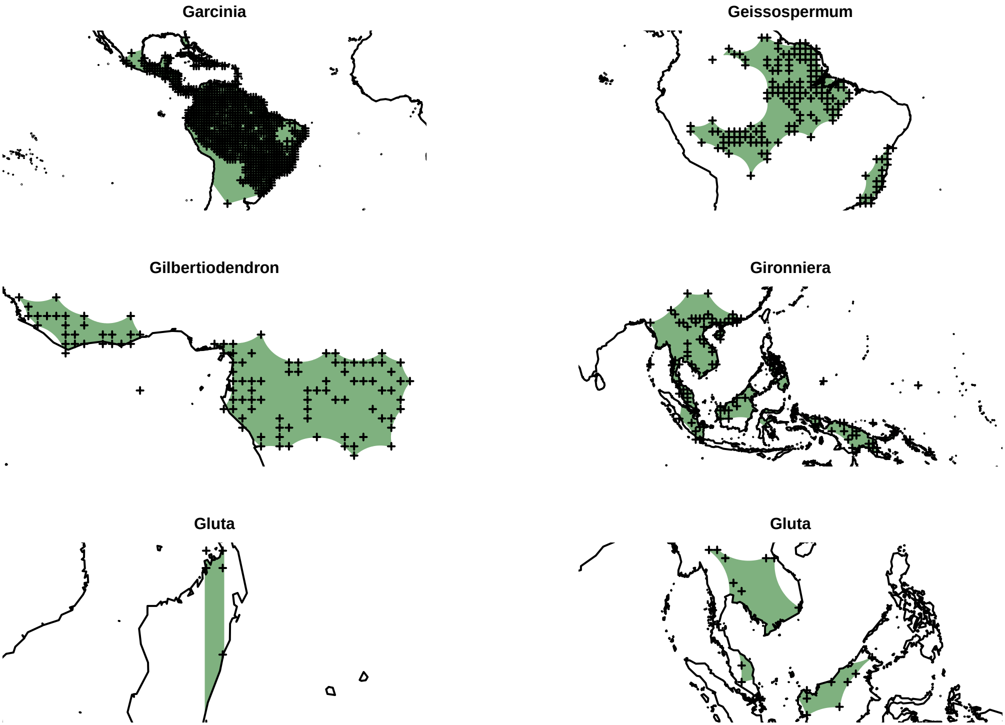
Garcinia
Geissospermum
Gilbertiodendron
Gironniera
Gluta
Gluta

Gonocaryum

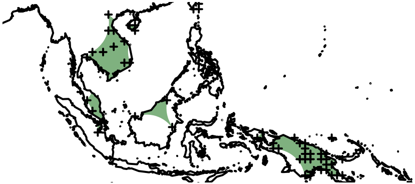

Gonystylus

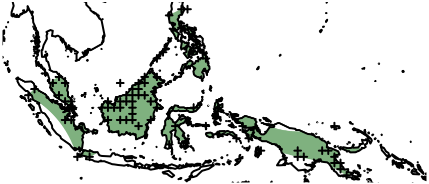

Goupia

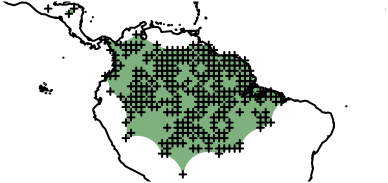

Greenwayodendron

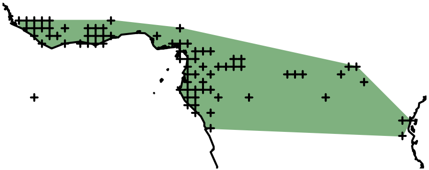

Grewia

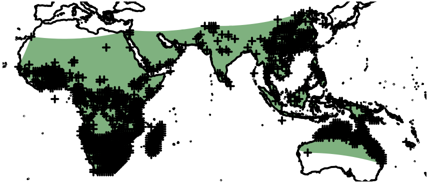

Grossera

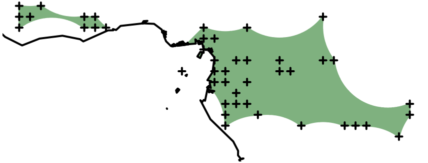

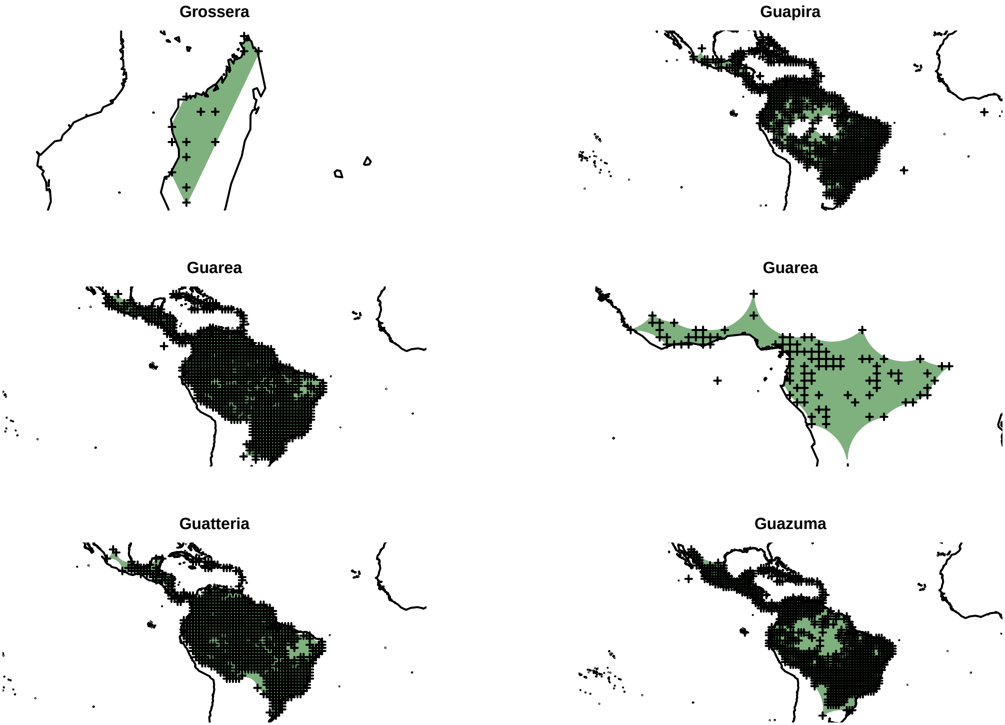
Grossera
Guapira
Guarea
Guarea
Guatteria
Guazuma

Guettarda

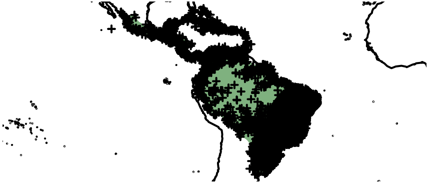

Gustavia

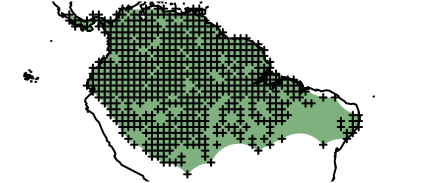

Gymnacranthera

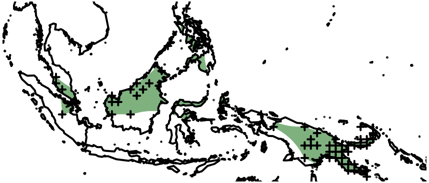

Hasseltia

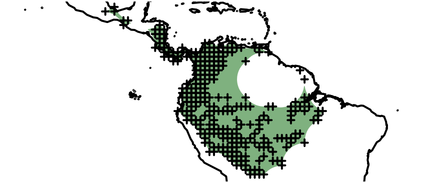

Heisteria

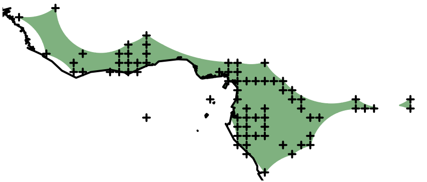

Heisteria

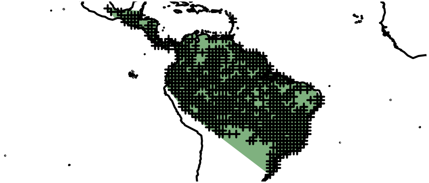

Helicostylis

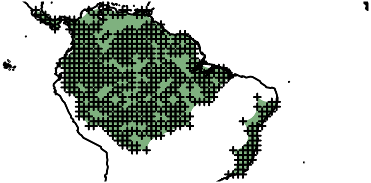

Heritiera

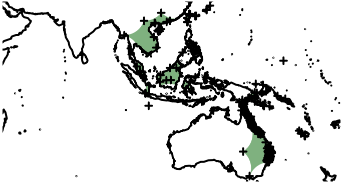

Heritiera

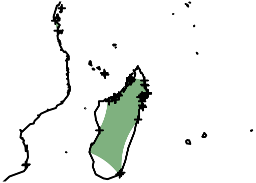

Hevea

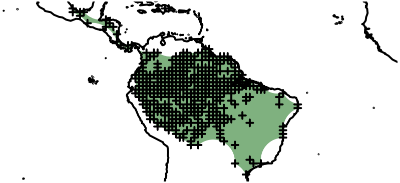

Hexalobus

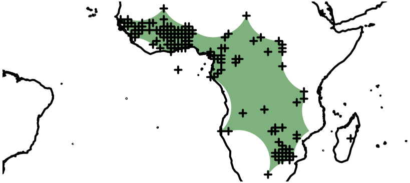

Hirtella

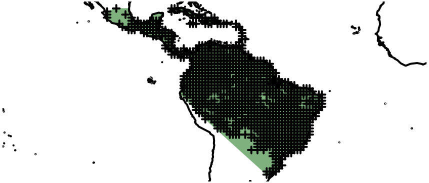

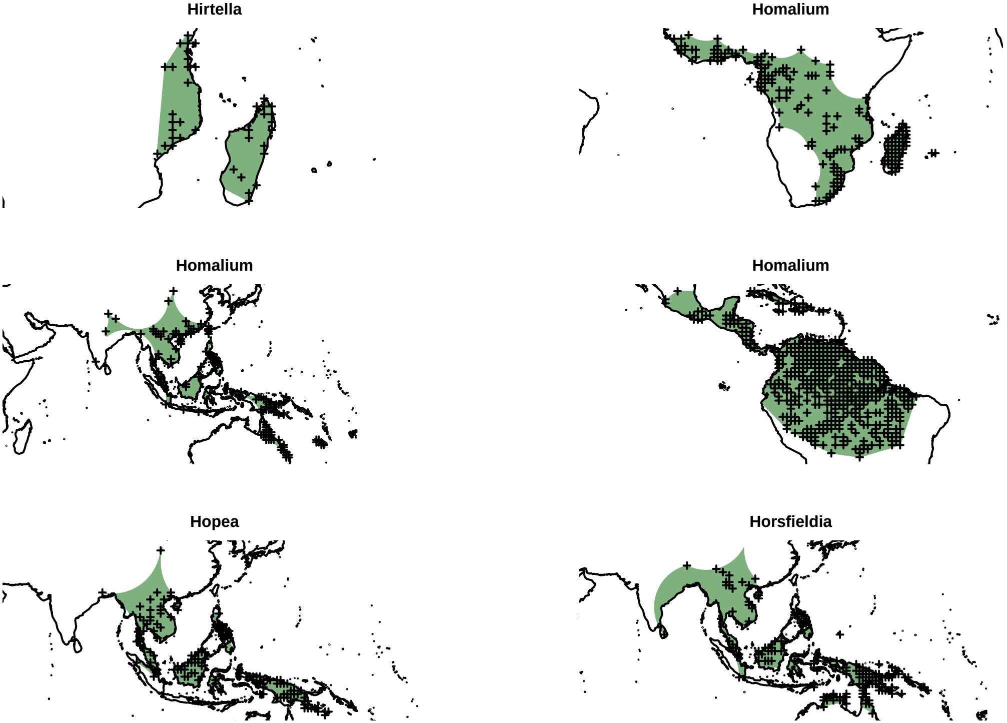
Hirtella
Homalium
Homalium
Homalium
Hopea
Horsfieldia

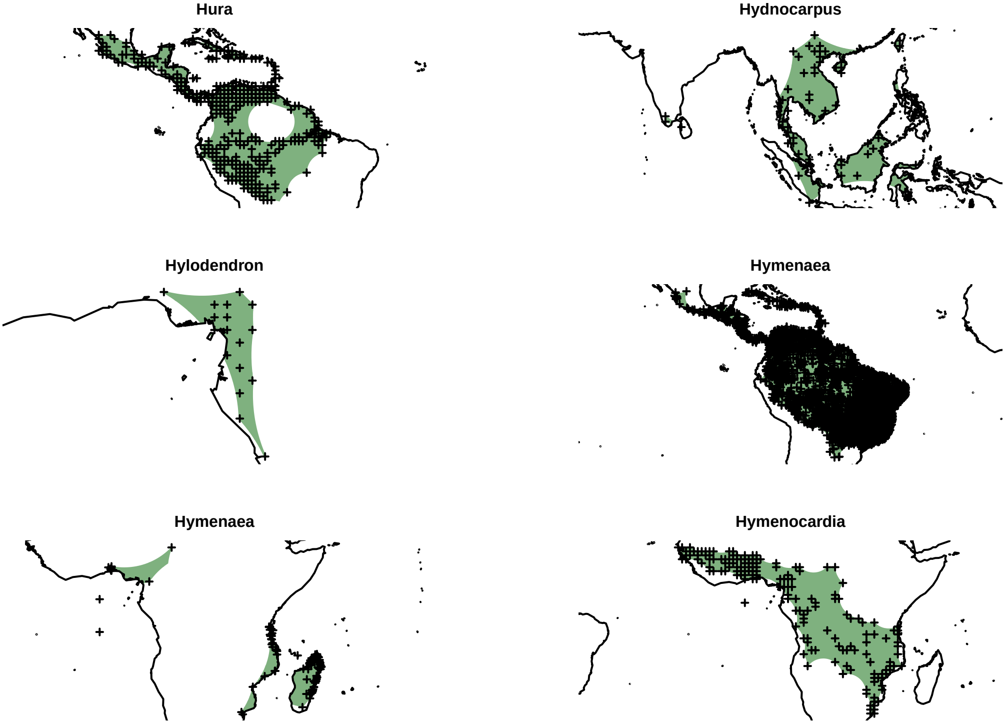

Hura
Hydnocarpus
Hylodendron
Hymenaea
Hymenaea
Hymenocardia

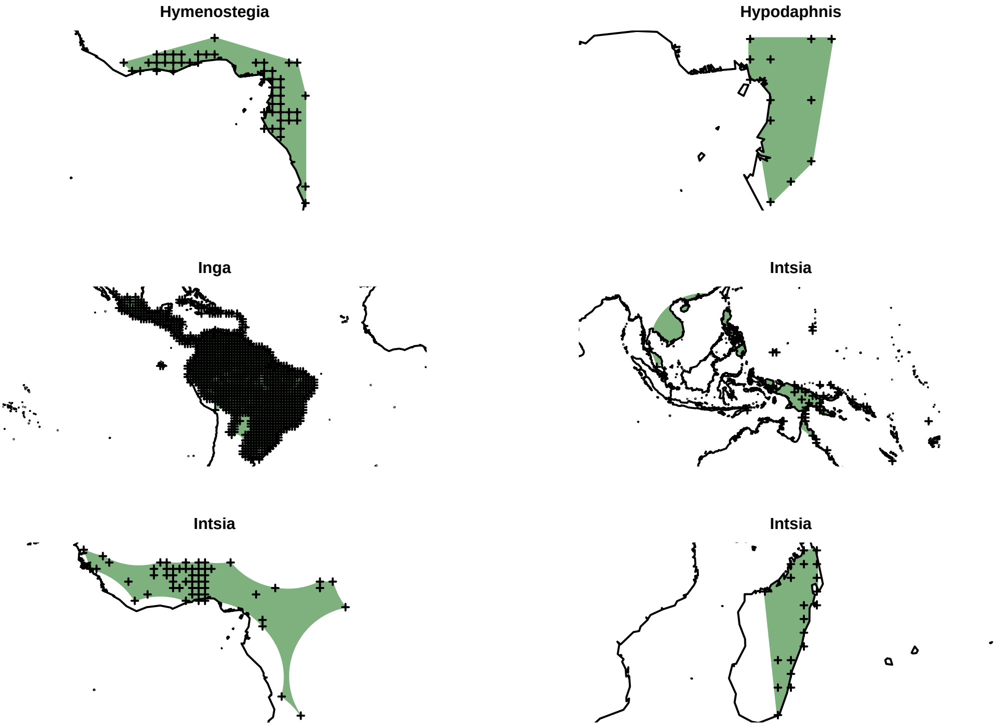

Hymenostegia
Hypodaphnis
Inga
Intsia
Intsia
Intsia

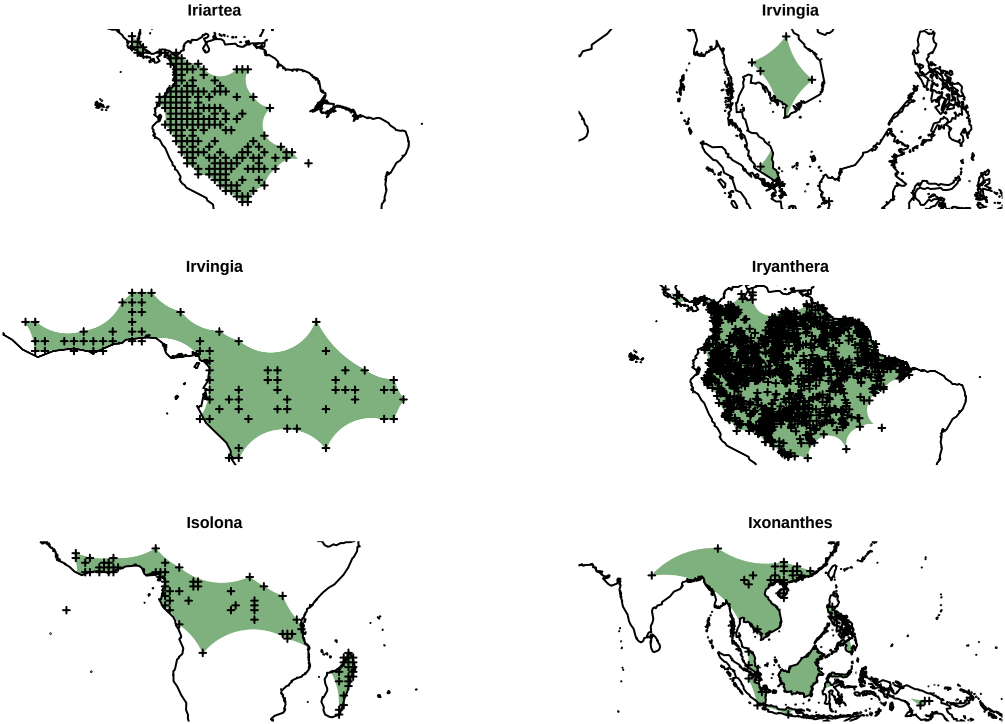
Iriartea
Irvingia
Irvingia
Iryanthera
Isolona
Ixonanthes
