# Supplementary material for: Large range sizes link fast life histories with high species richness across wet tropical tree floras
Source: Sci Rep. 2025 Feb 8;15:4695. doi: 10.1038/s41598-024-84367-3 (PMC11807110; doi:10.1038/s41598-024-84367-3)
Supplement: Supplementary file 6 — Supplementary Information 6. [file 41598_2024_84367_MOESM6_ESM.pdf]

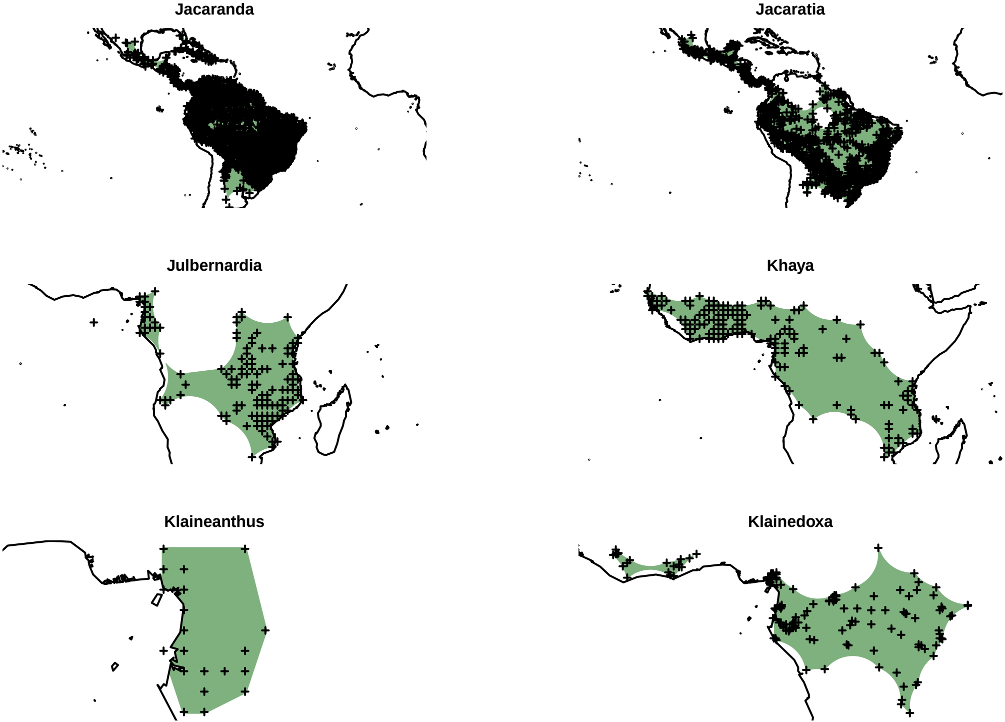
Jacaranda
Jacaratia
Julbernardia
Khaya
Klaineanthus
Klainedoxa

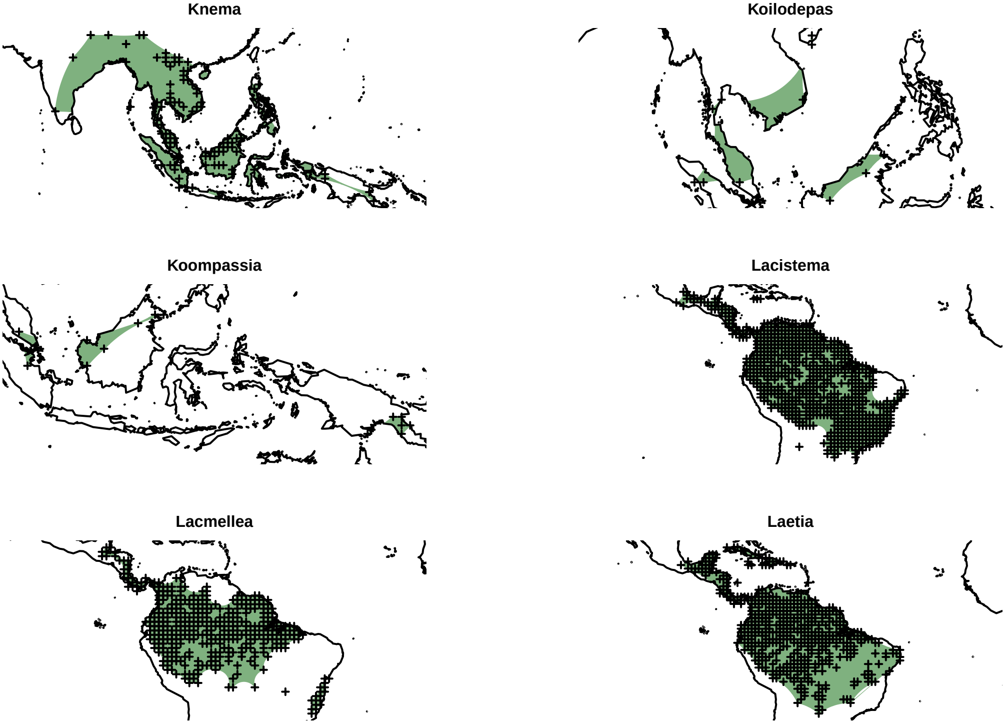
Knema
Koilodepas
Koompassia
Lacistema
Lacmellea
Laetia

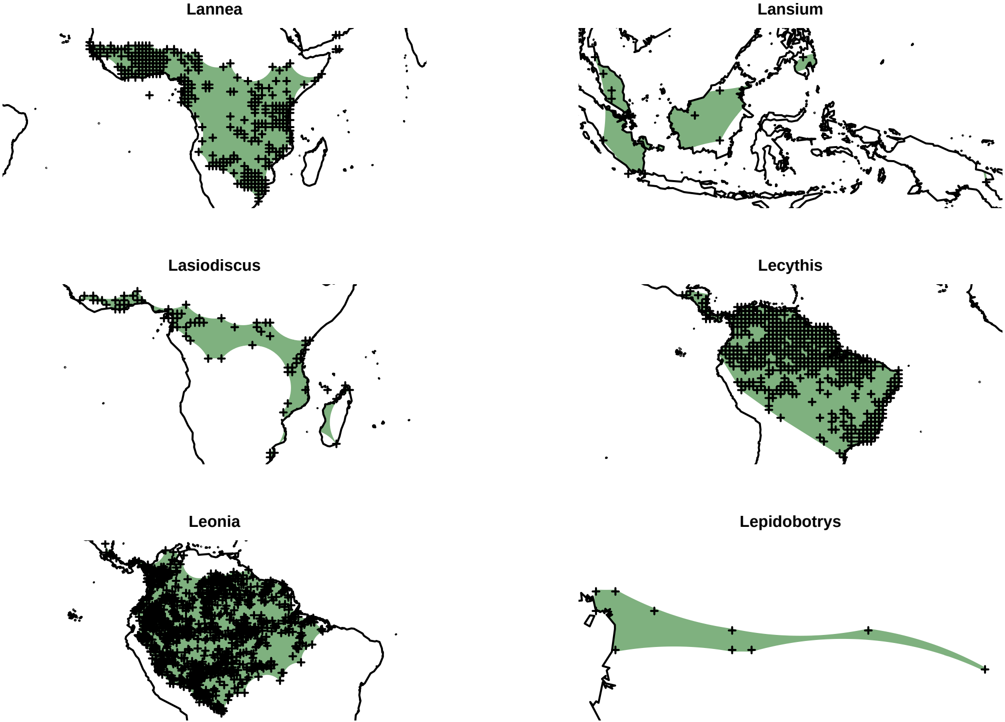
Lannea
Lansium
Lasiodiscus
Lecythis
Leonia
Lepidobotrys

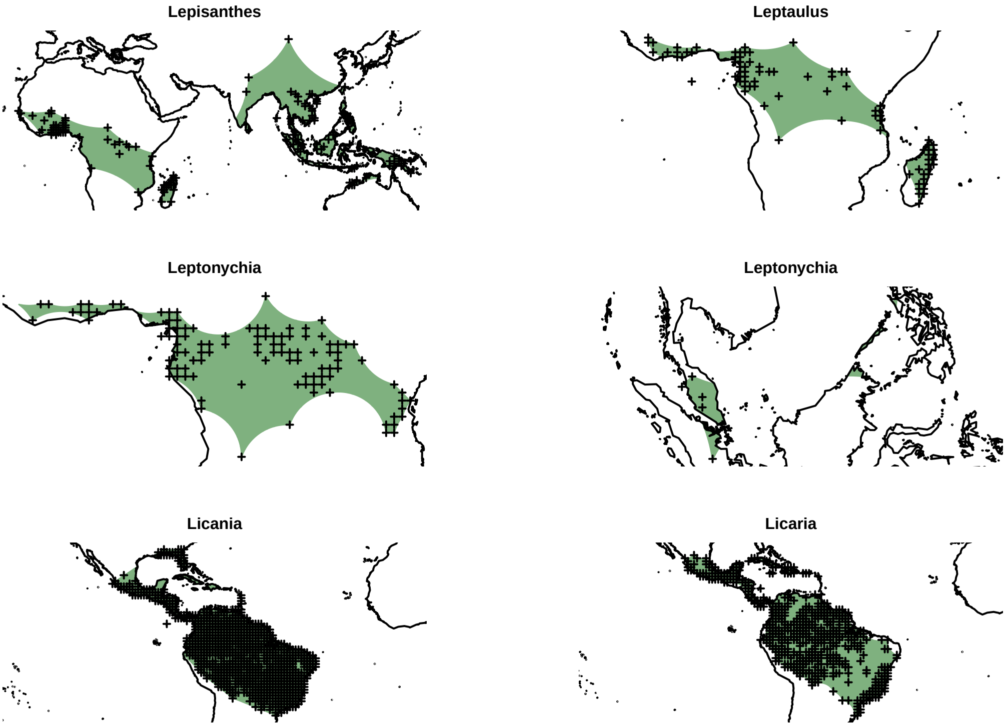
Lepisanthes
Leptaulus
Leptonychia
Leptonychia
Licania
Licaria

Lindackeria

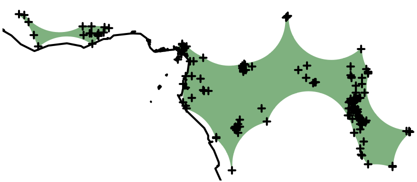

Lindackeria

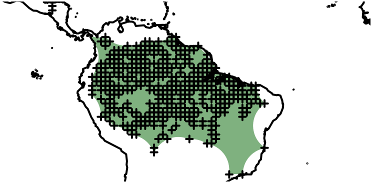

Lithocarpus

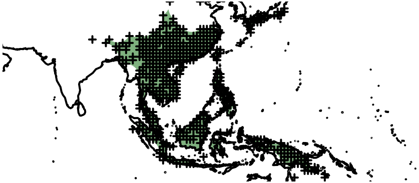

Litsea

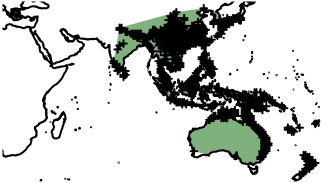

Litsea

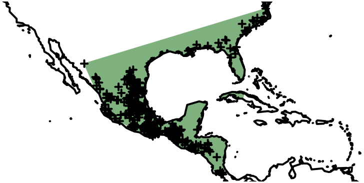

Lonchocarpus

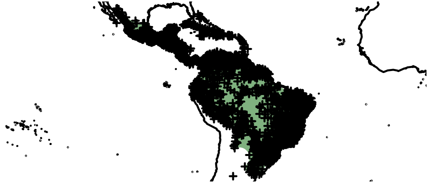

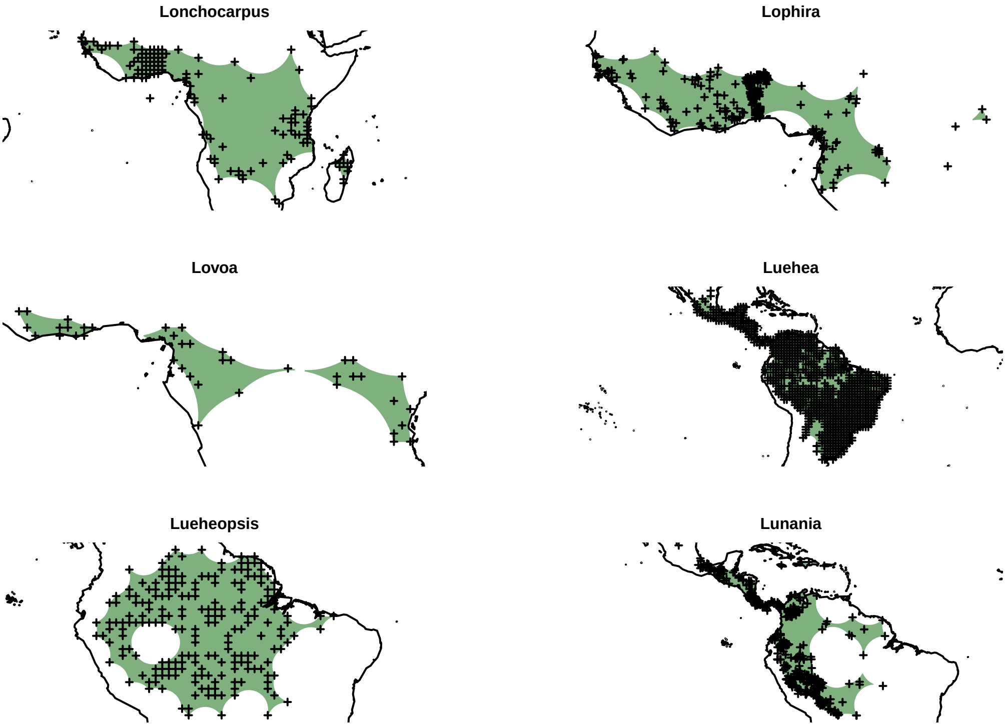
Lonchocarpus
Lophira
Lovoa
Luehea
Lueheopsis
Lunania

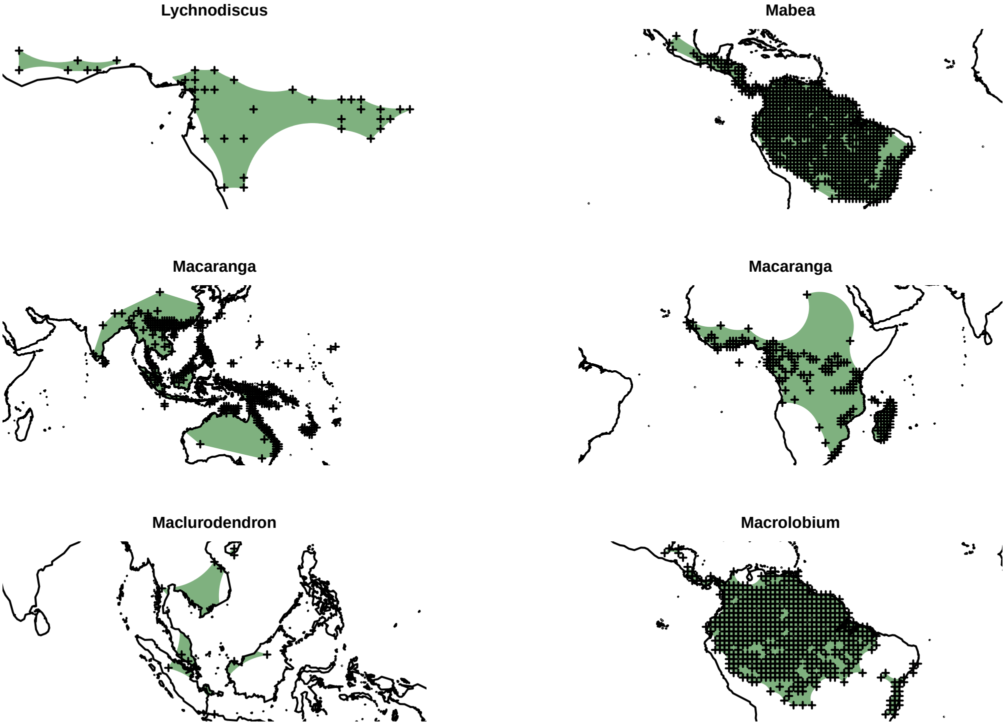
Lychnodiscus
Mabea
Macaranga
Macaranga
Maclurodendron
Macrolobium

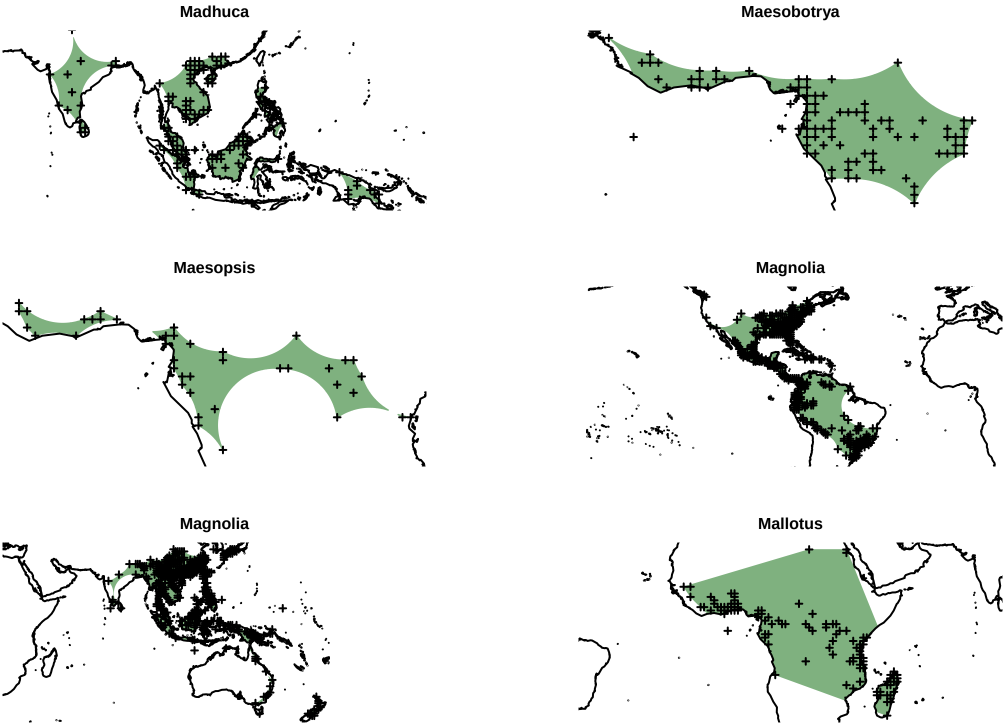

Madhuca
Maesobotrya
Maesopsis
Magnolia
Magnolia
Mallotus

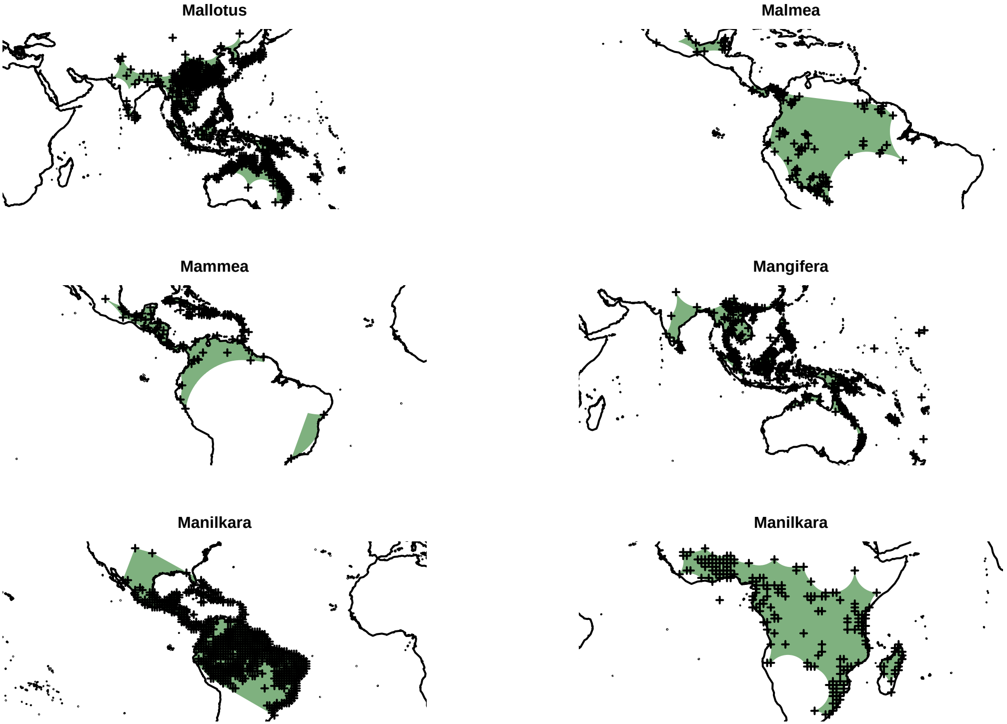
Mallotus
Malmea
Mammea
Mangifera
Manilkara
Manilkara

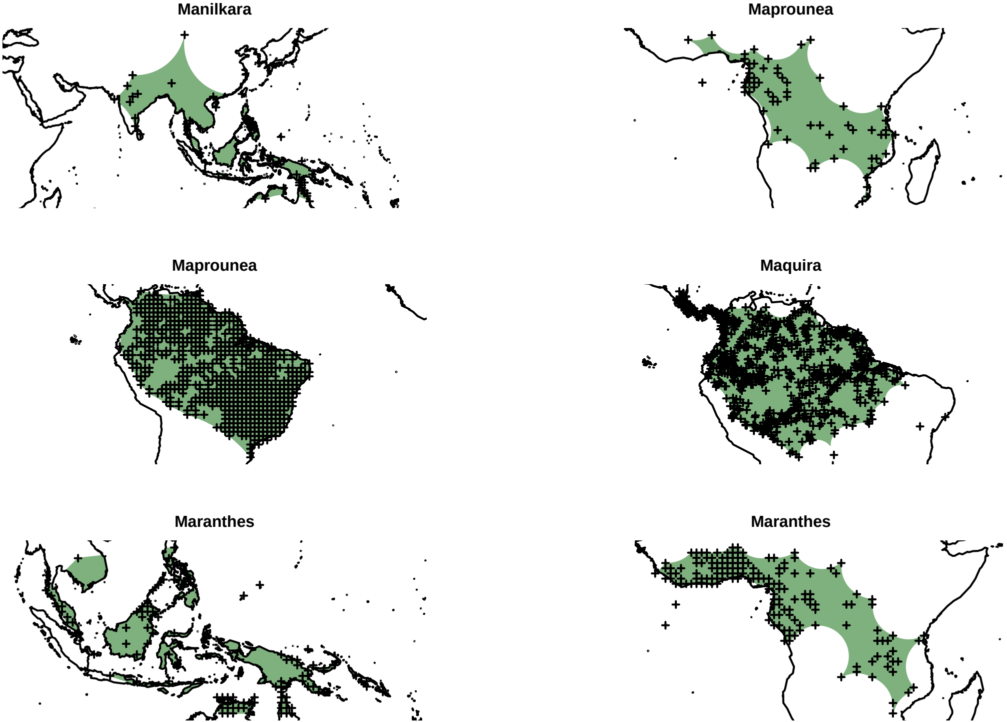
Manilkara
Maprounea
Maprounea
Maquira
Maranthes
Maranthes
