# Supplementary material for: Large range sizes link fast life histories with high species richness across wet tropical tree floras
Source: Sci Rep. 2025 Feb 8;15:4695. doi: 10.1038/s41598-024-84367-3 (PMC11807110; doi:10.1038/s41598-024-84367-3)
Supplement: Supplementary file 7 — Supplementary Information 7. [file 41598_2024_84367_MOESM7_ESM.pdf]

Mareya

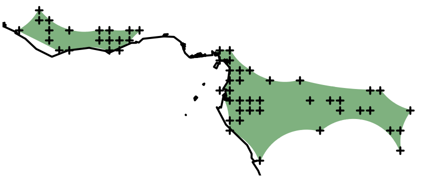

Mareyopsis

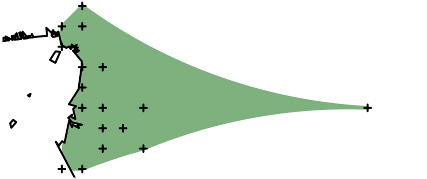

Margaritaria

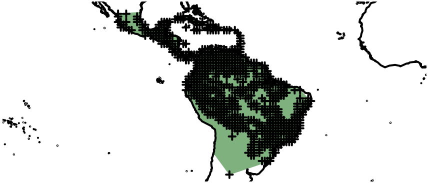

Margaritaria

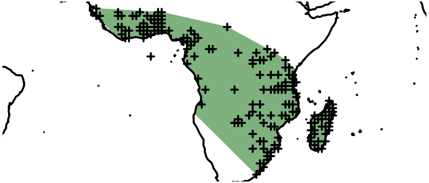

Margaritaria

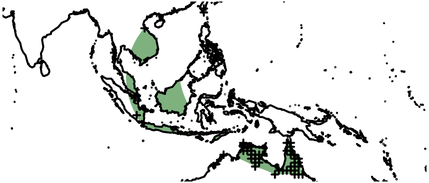

Markhamia

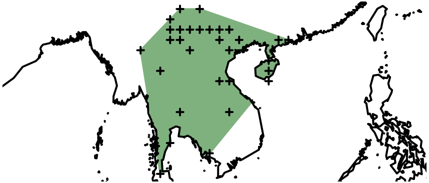

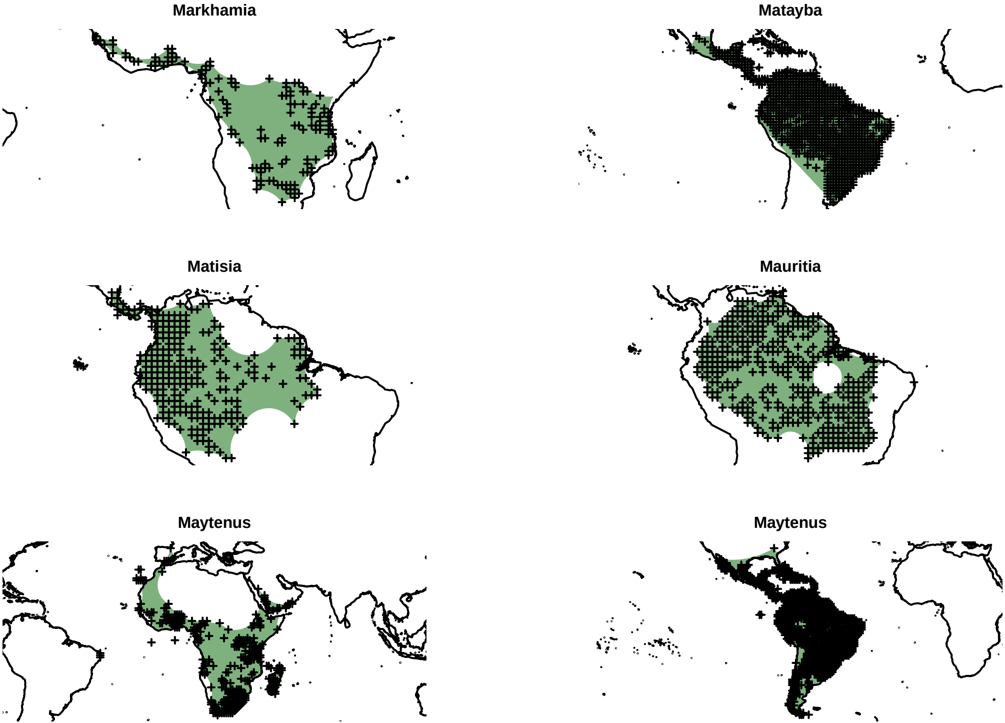
Markhamia
Matayba
Matisia
Mauritia
Maytenus
Maytenus

Memecylon

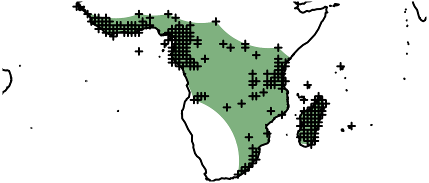

Memecylon

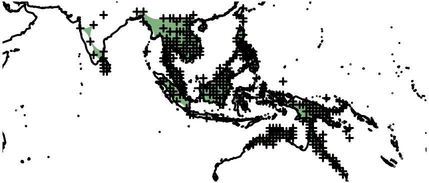

Mesua

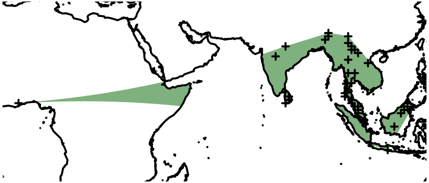

Metrodorea

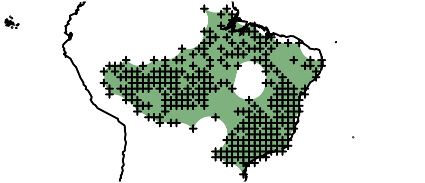

Mezilaurus

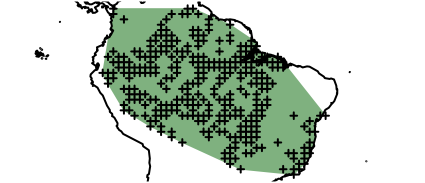

Mezzettia

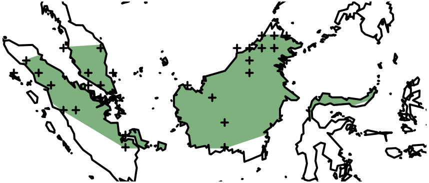

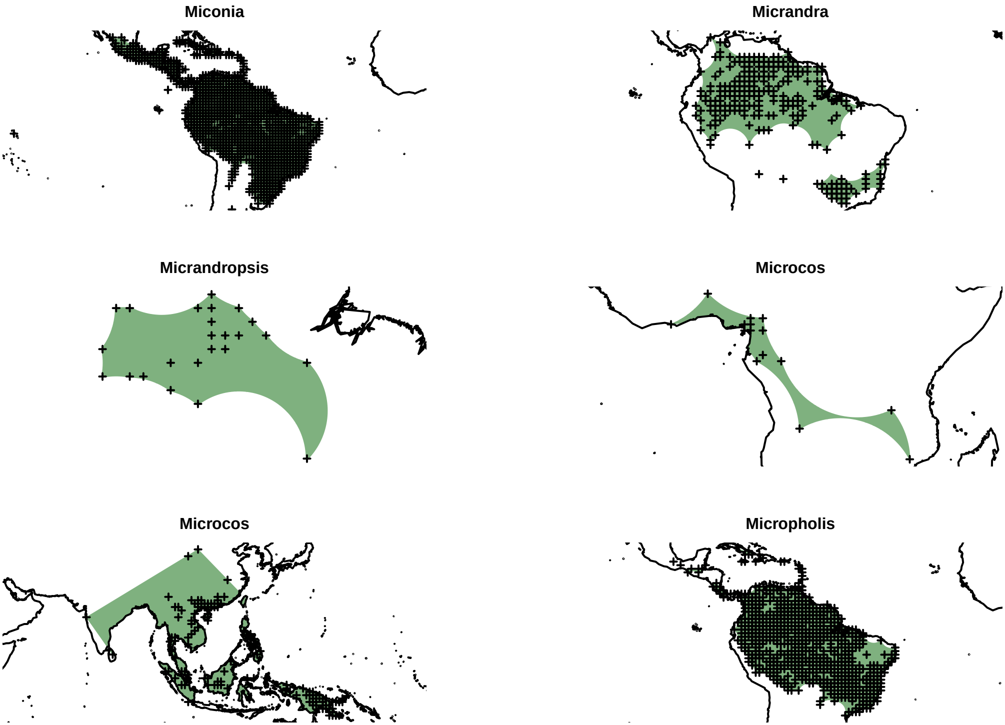
Miconia
Micrandra
Micrandropsis
Microcos
Microcos
Micropholis

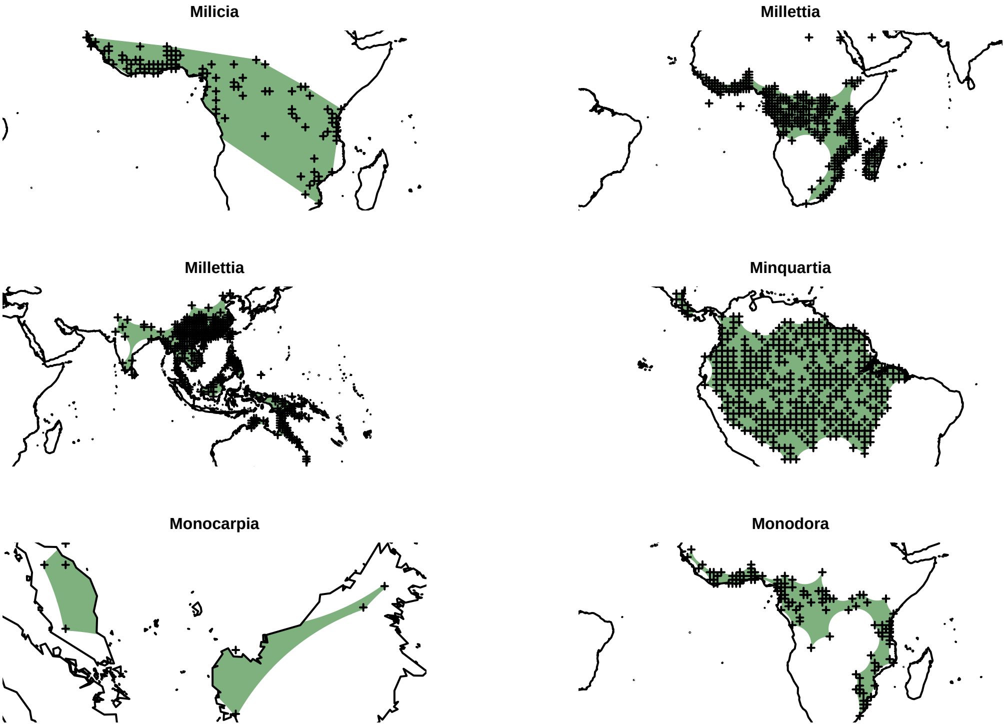
Milicia
Millettia
Millettia
Minquartia
Monocarpia
Monodora

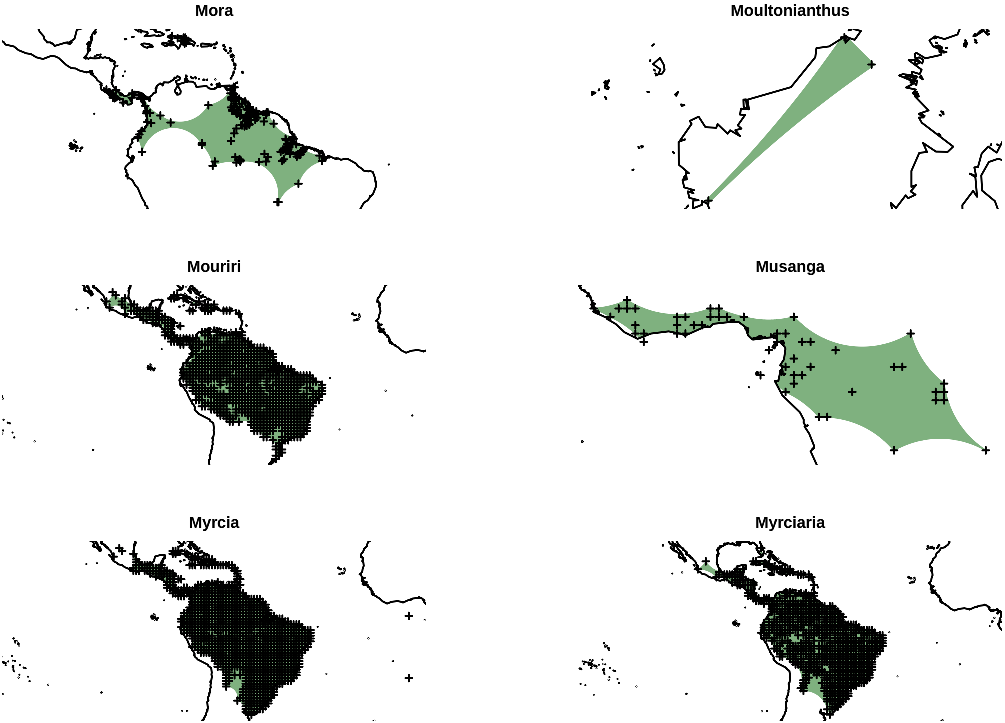
Mora
Moultonianthus
Mouriri
Musanga
Myrcia
Myrciaria

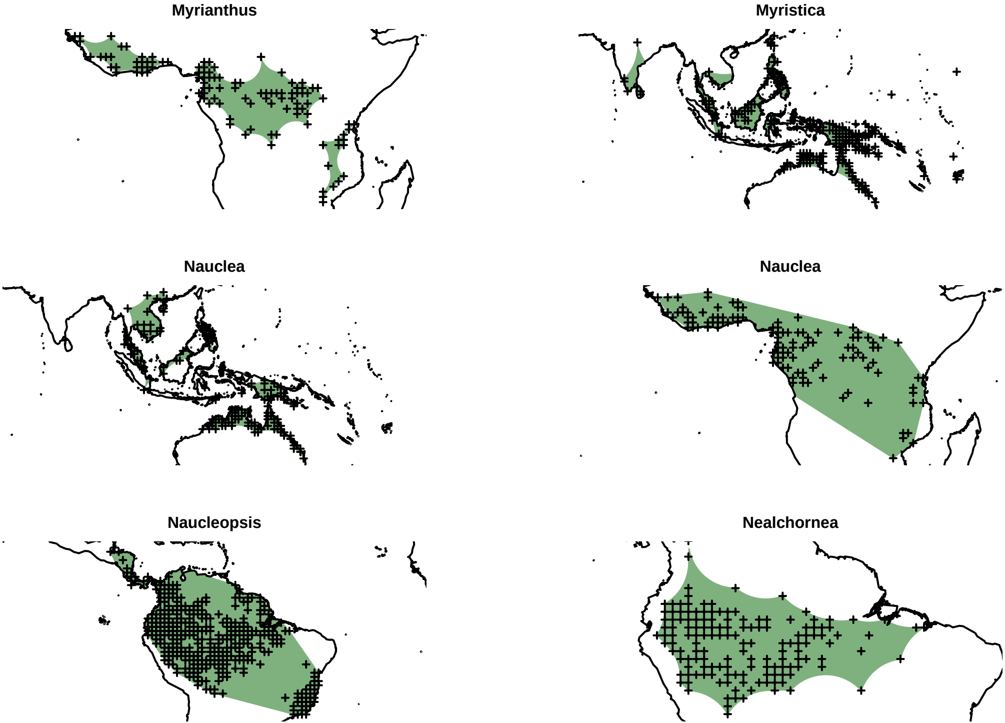
Myrianthus
Myristica
Nauclea
Nauclea
Naucleopsis
Nealchornea

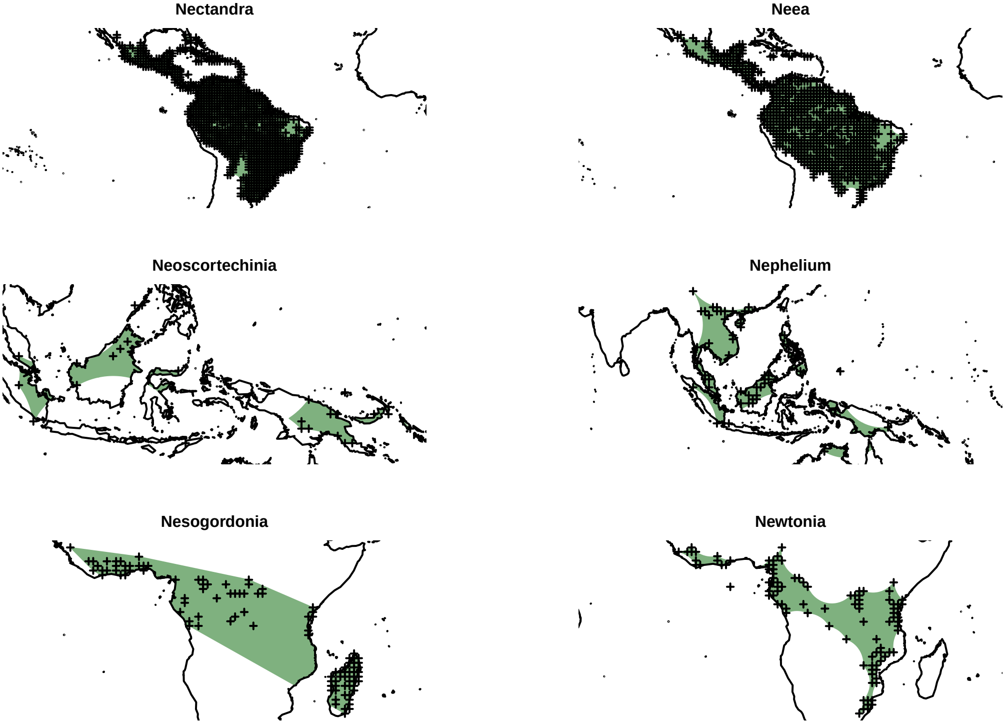
Nectandra
Neea
Neoscortechinia
Nephelium
Nesogordonia
Newtonia

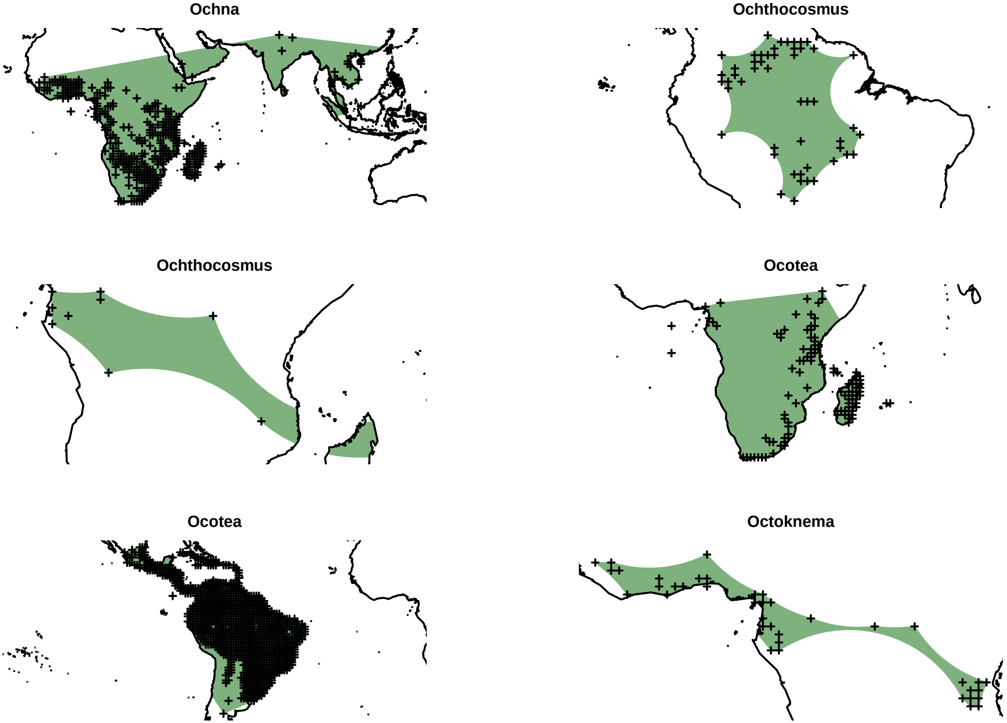
Ochna
Ochthocosmus
Ochthocosmus
Ocotea
Ocotea
Octoknema

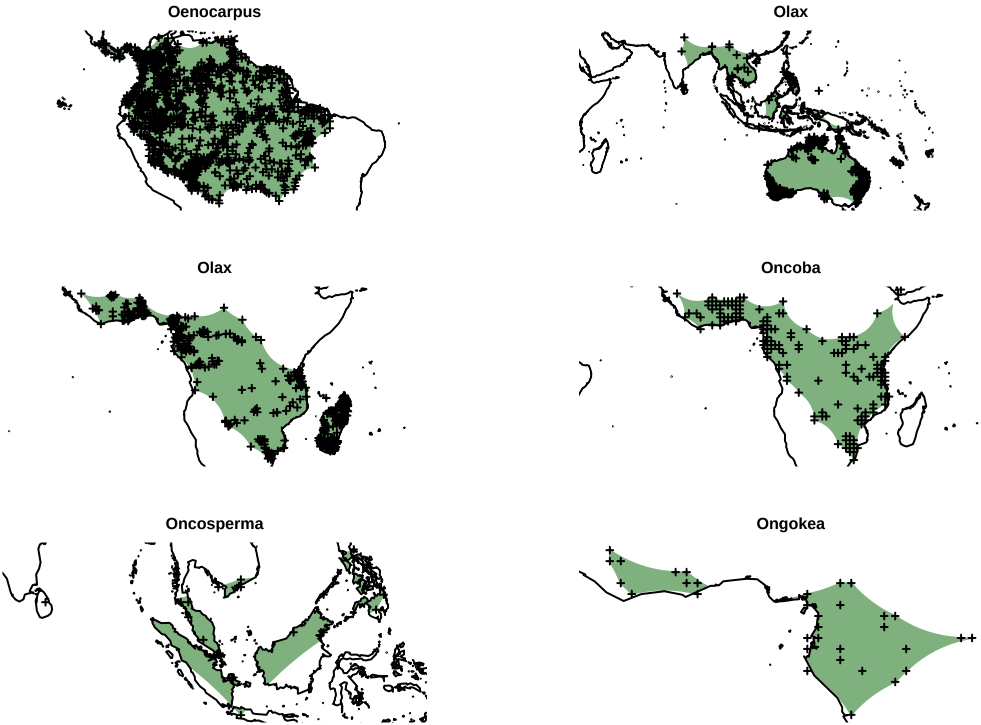
Oenocarpus
Olax
Olax
Oncoba
Oncosperma
Ongokea
