# Supplementary material for: Large range sizes link fast life histories with high species richness across wet tropical tree floras
Source: Sci Rep. 2025 Feb 8;15:4695. doi: 10.1038/s41598-024-84367-3 (PMC11807110; doi:10.1038/s41598-024-84367-3)
Supplement: Supplementary file 8 — Supplementary Information 8. [file 41598_2024_84367_MOESM8_ESM.pdf]

Ormosia

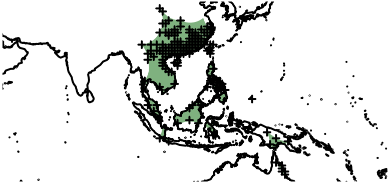

Ormosia

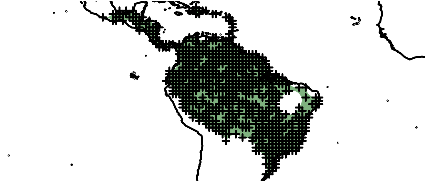

Osteophloeum

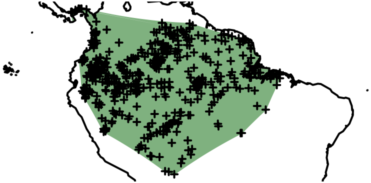

Oubanguia

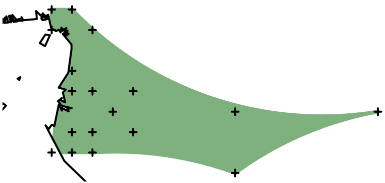

Ouratea

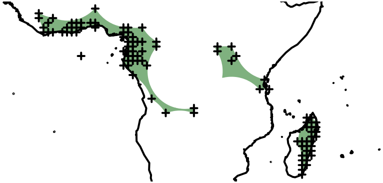

Ouratea

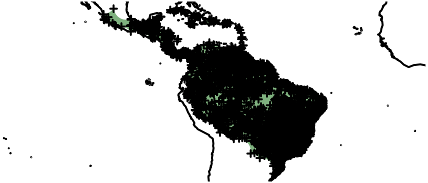

Oxandra

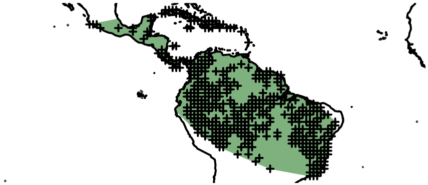

Pachira

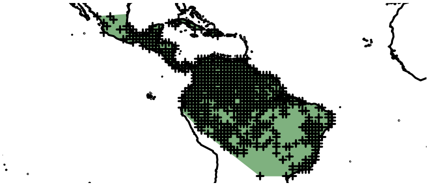

Palaquium

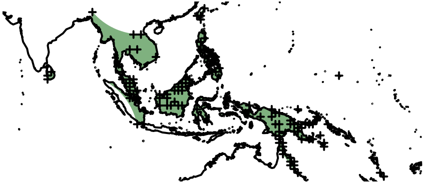

Pancovia

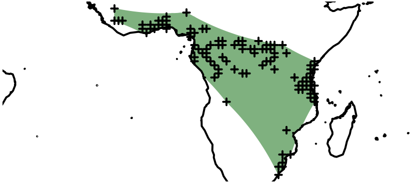

Panda

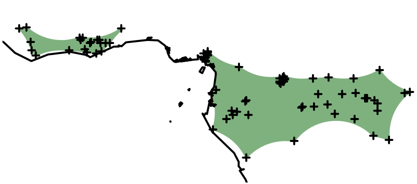

Paramachaerium

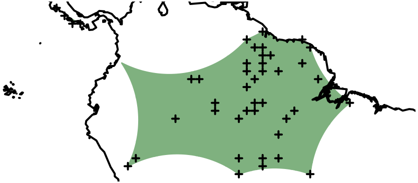

Paranephelium

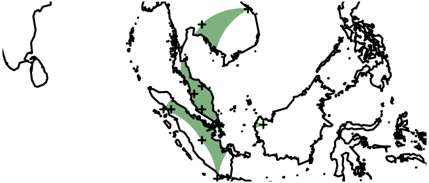

Parashorea

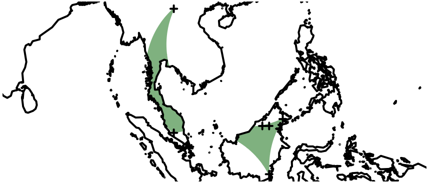

Parinari

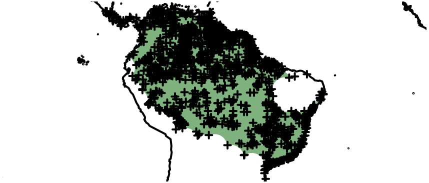

Parinari

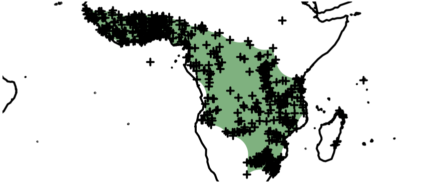

Parinari

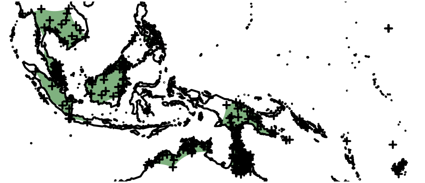

Parkia

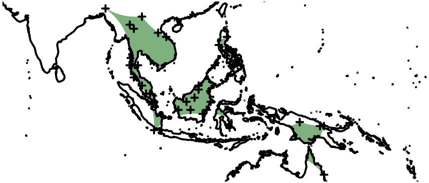

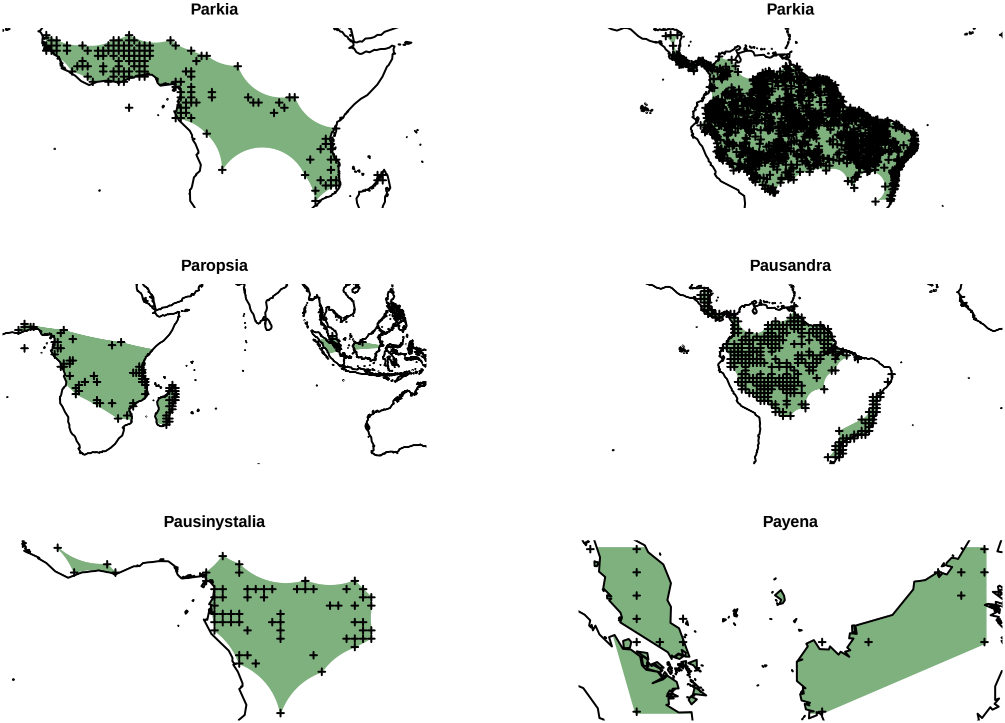
Parkia
Parkia
Paropsia
Pausandra
Pausinystalia
Payena

Peltogyne

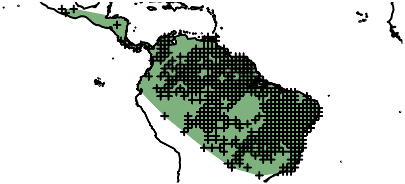

Pentaclethra

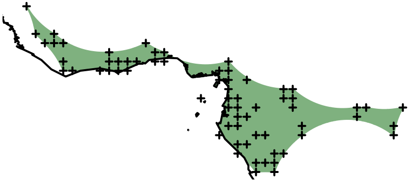

Pentaclethra

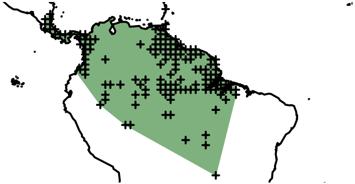

Pentadesma

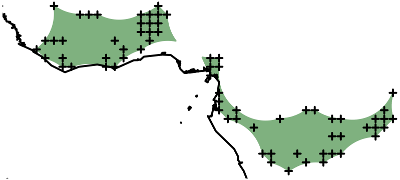

Perebea

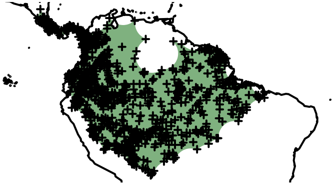

Petersianthus

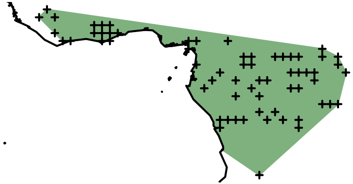

Phyllocosmus

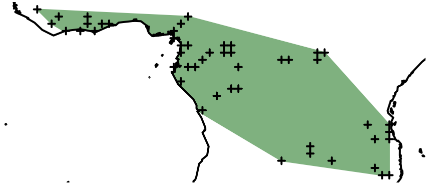

Picralima

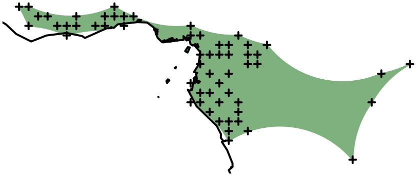

Pimelodendron

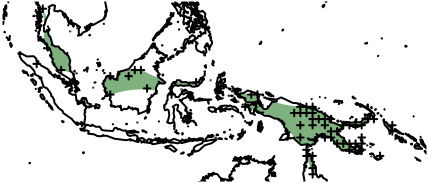

Piptadeniastrum

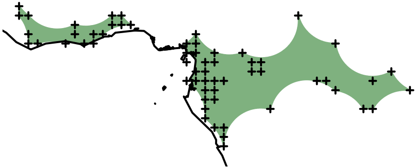

Pithecellobium

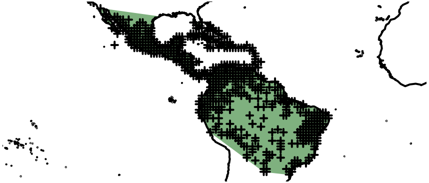

Pithecellobium

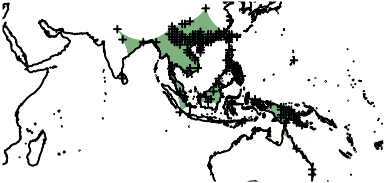

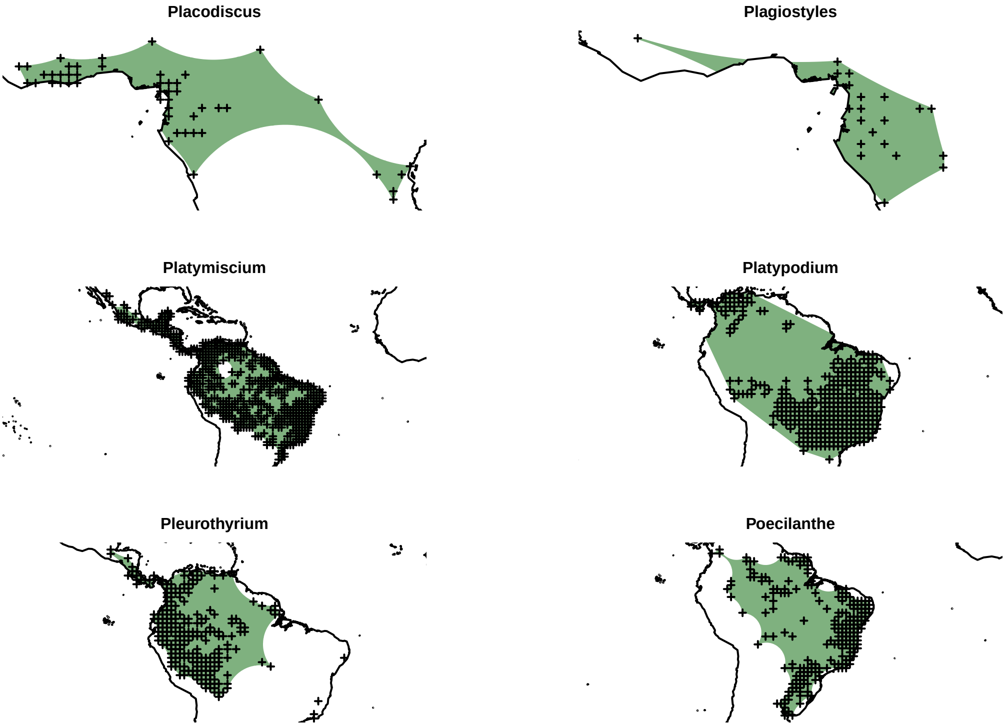
Placodiscus
Plagiostyles
Platymiscium
Platypodium
Pleurothyrium
Poecilanthe

Polyalthia

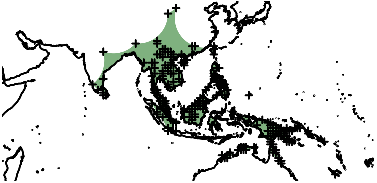

Polyalthia

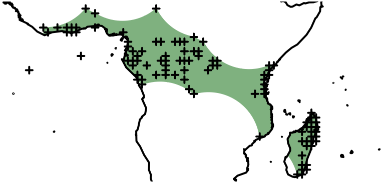

Poraqueiba

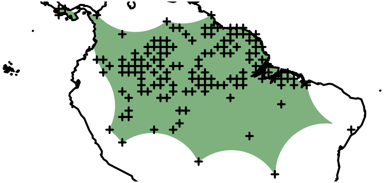

Porterandia

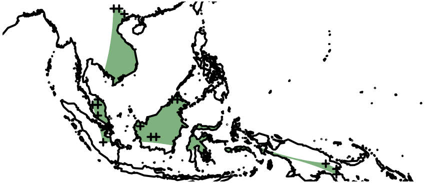

Poulsenia

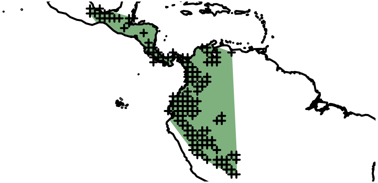

Pourouma

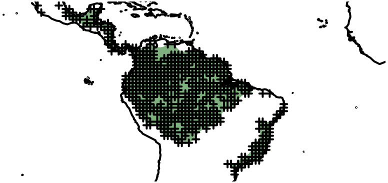

Pouteria

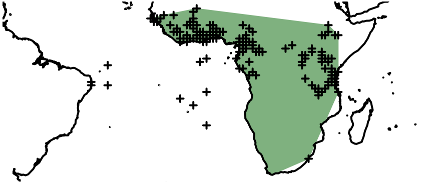

Pouteria

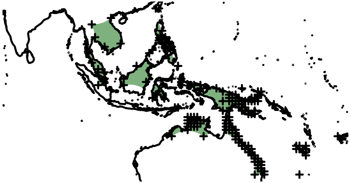

Pouteria

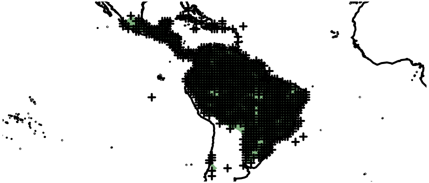

Pradosia

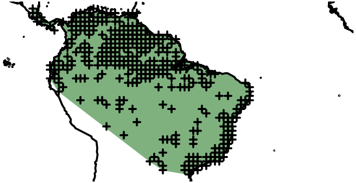

Prioria

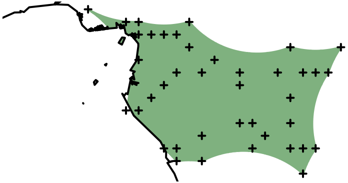

Prioria

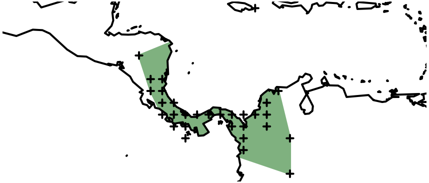

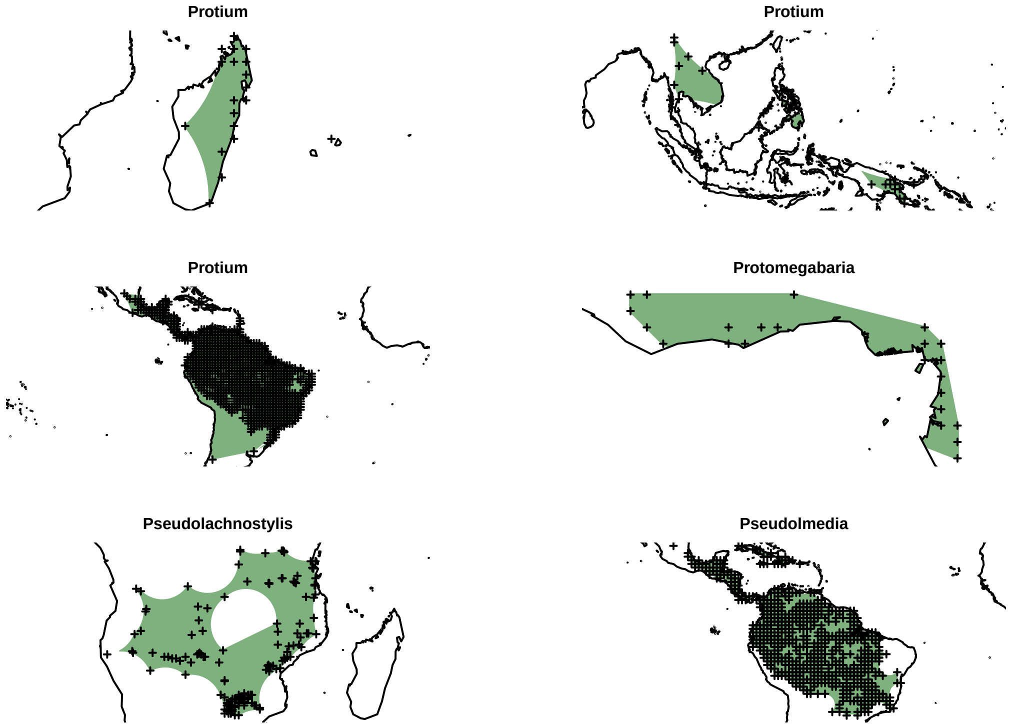
Protium
Protium
Protium
Protomegabaria
Pseudolachnostylis
Pseudolmedia
