# Supplementary material for: Large range sizes link fast life histories with high species richness across wet tropical tree floras
Source: Sci Rep. 2025 Feb 8;15:4695. doi: 10.1038/s41598-024-84367-3 (PMC11807110; doi:10.1038/s41598-024-84367-3)
Supplement: Supplementary file 9 — Supplementary Information 9. [file 41598_2024_84367_MOESM9_ESM.pdf]

Pseudopiptadenia

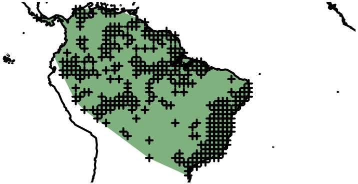

Pseudospondias

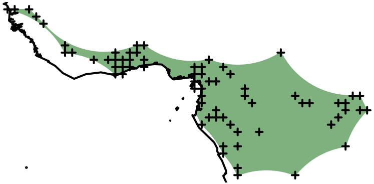

Pseudoxandra

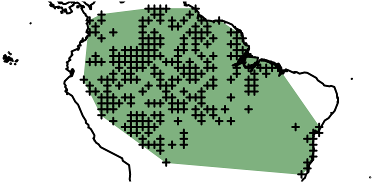

Psidium

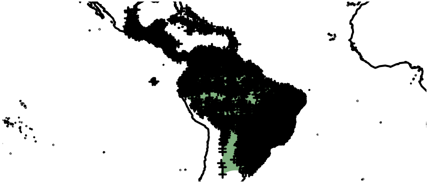

Psydrax

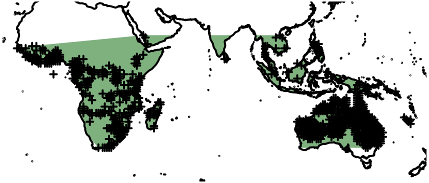

Pteleopsis

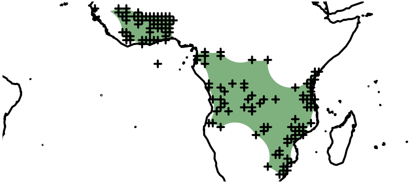

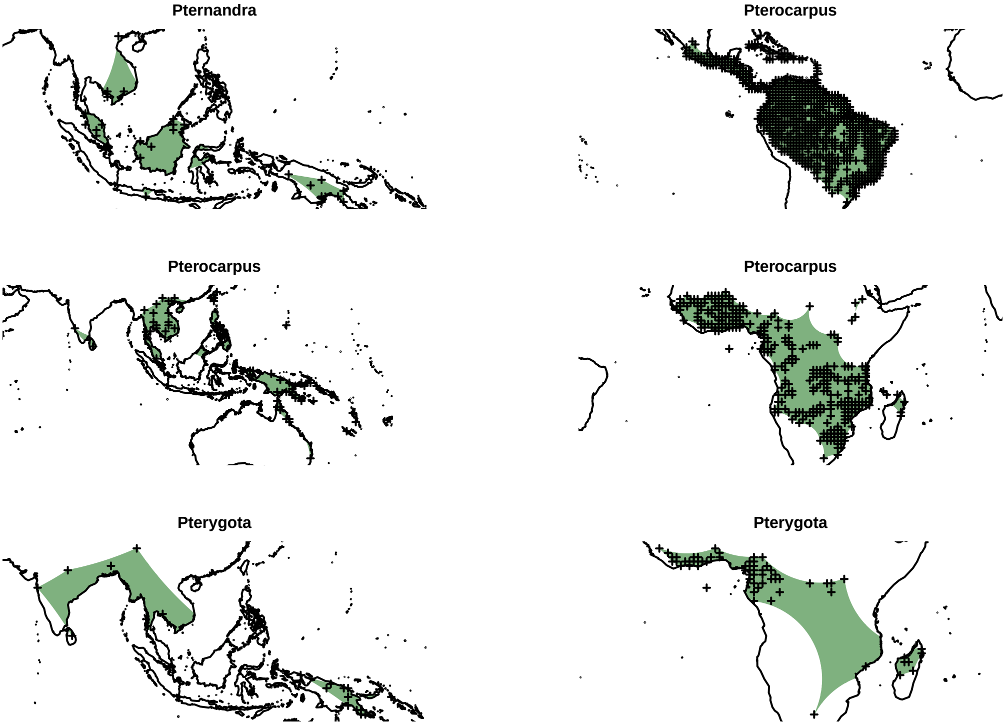

Pternandra
Pterocarpus
Pterocarpus
Pterocarpus
Pterygota
Pterygota

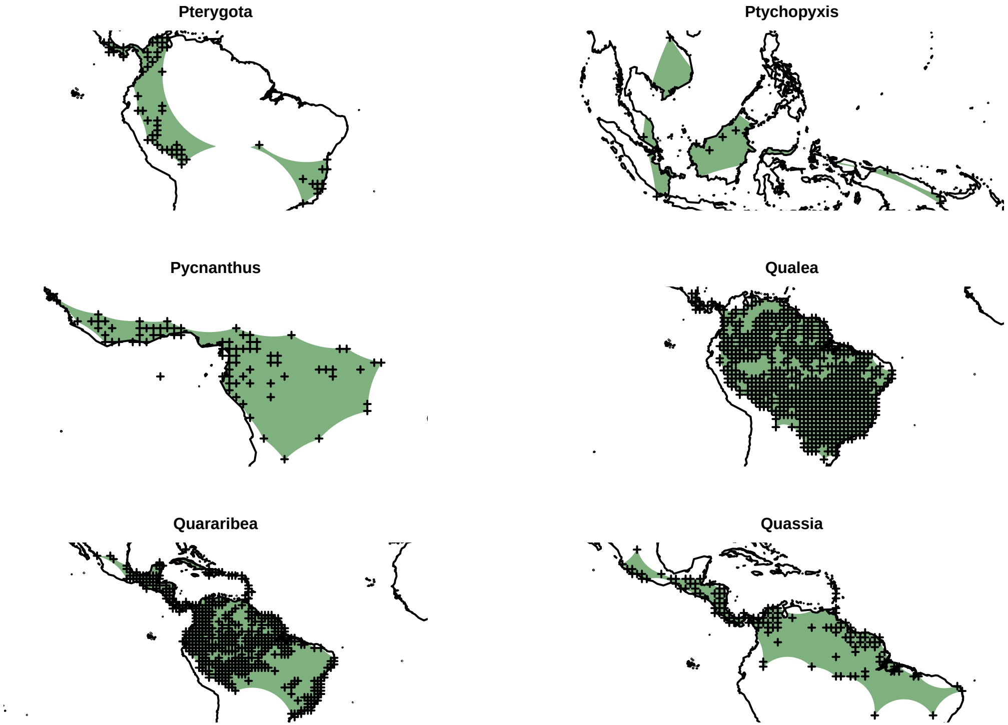
Pterygota
Ptychopyxis
Pycnanthus
Qualea
Quararibea
Quassia

Quassia

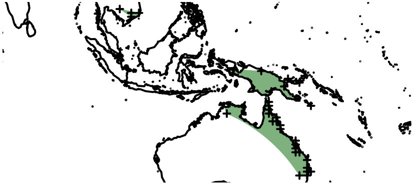

Quassia

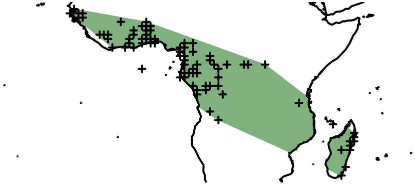

Quiina

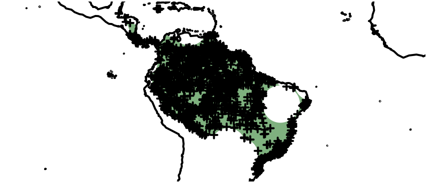

Rauvolfia

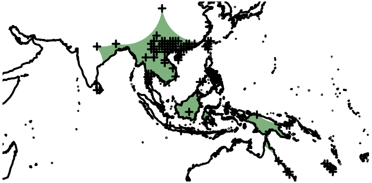

Rauvolfia

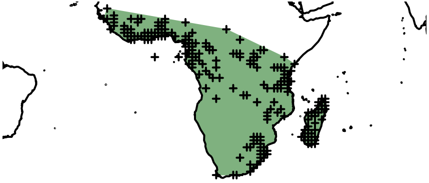

Rauvolfia

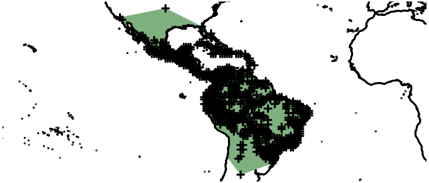

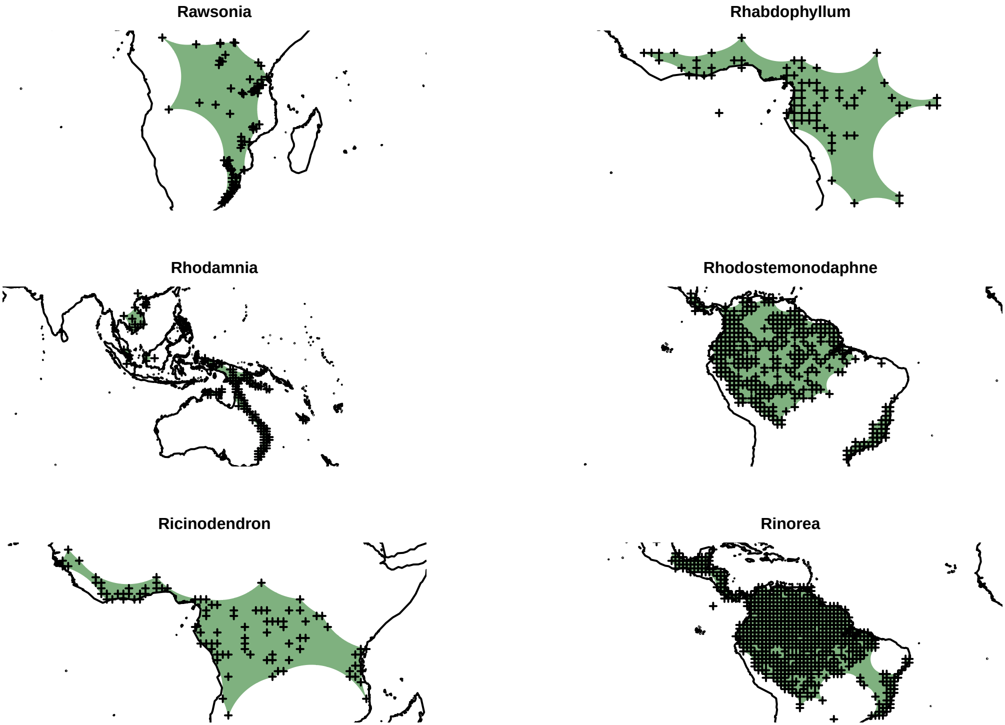
Rawsonia
Rhabdophyllum
Rhodamnia
Rhodostemonodaphne
Ricinodendron
Rinorea

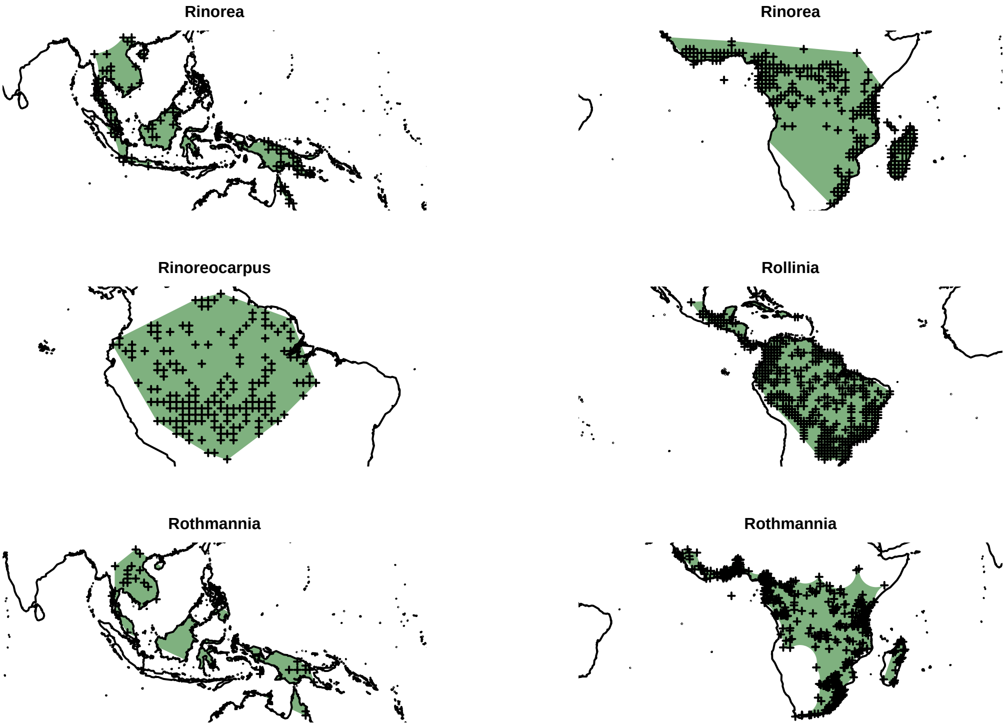
Rinorea
Rinorea
Rinoreocarpus
Rollinia
Rothmannia
Rothmannia

Roucheria

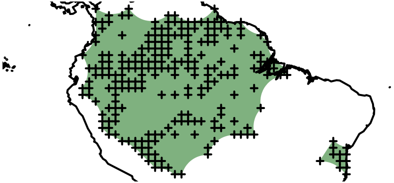

Ruizodendron

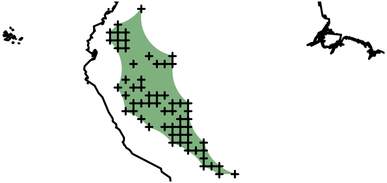

Sacoglottis

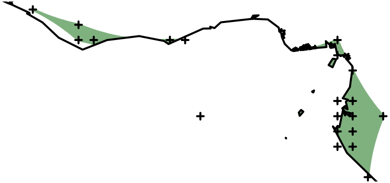

Sacoglottis

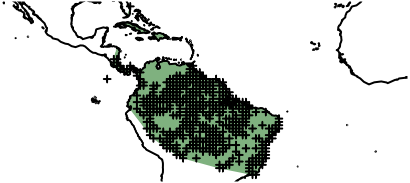

Sagotia

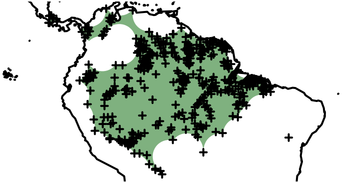

Santiria

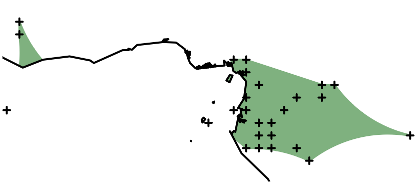

Santiria

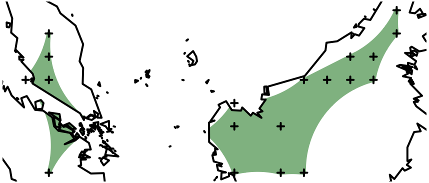

Sapium

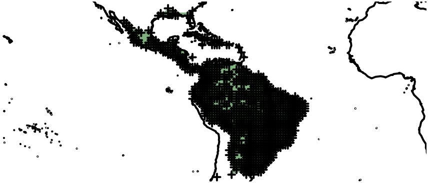

Sapium

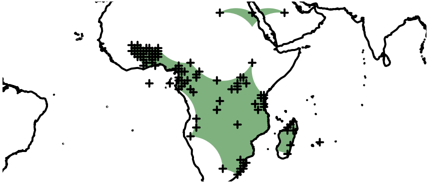

Saraca

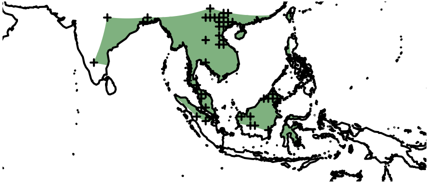

Sarcaulus

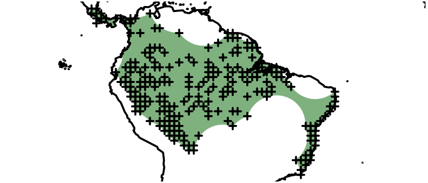

Sarcotheca

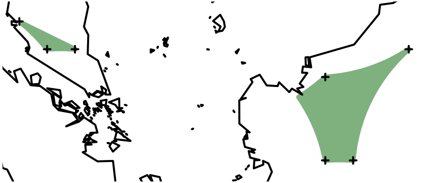

Scaphium

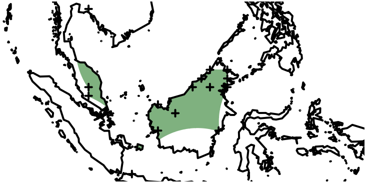

Scleronema

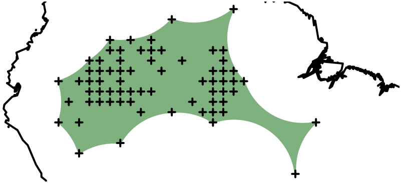

Scorodophloeus

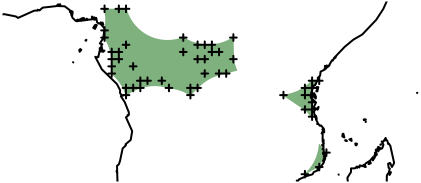

Scottellia

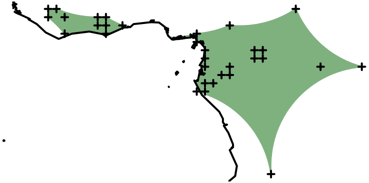

Scytopetalum

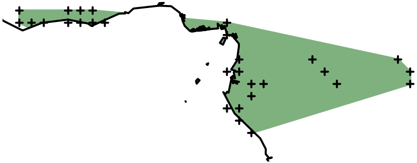

Shorea

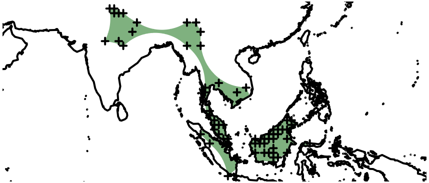

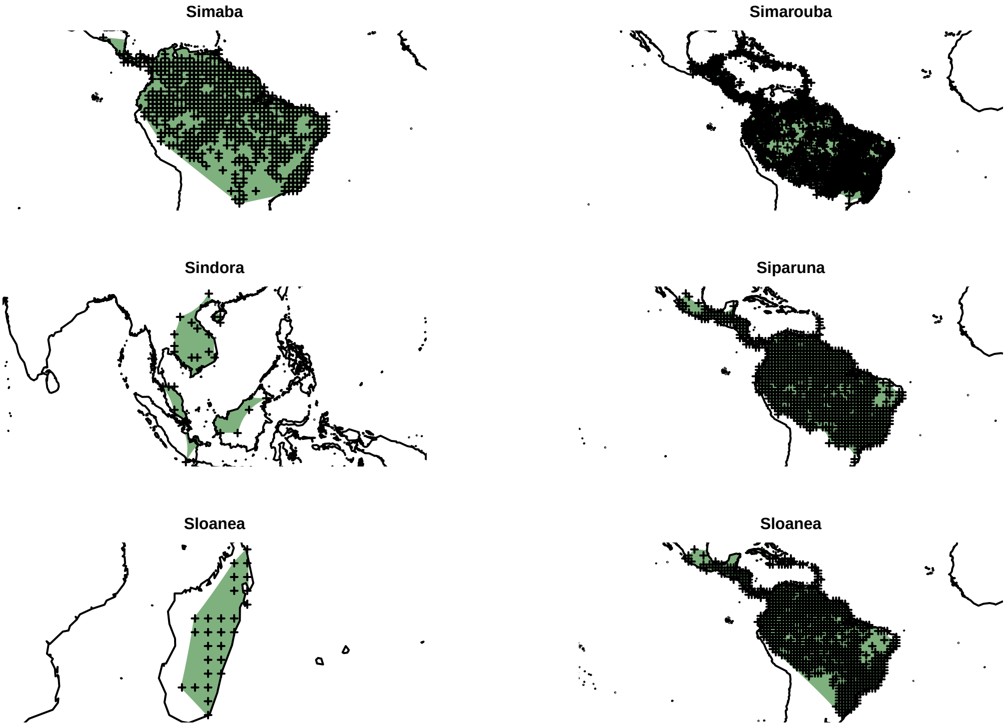
Simaba
Simarouba
Sindora
Siparuna
Sloanea
Sloanea
