# Supplementary material for: Large range sizes link fast life histories with high species richness across wet tropical tree floras
Source: Sci Rep. 2025 Feb 8;15:4695. doi: 10.1038/s41598-024-84367-3 (PMC11807110; doi:10.1038/s41598-024-84367-3)

Sloanea

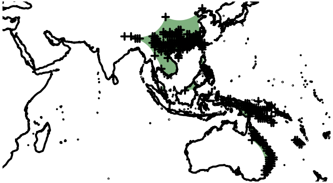

Socratea

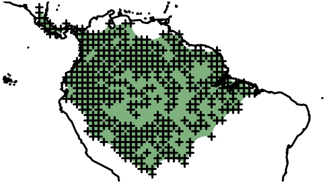

Sorindeia

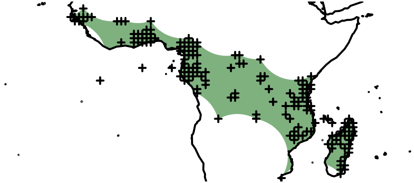

Sorocea

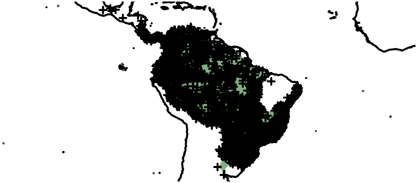

Spondias

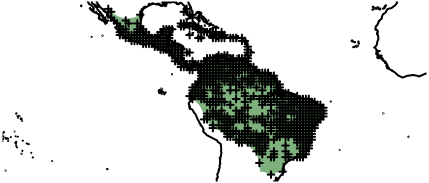

Spondias

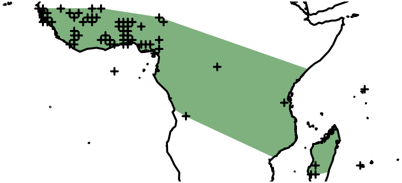

Spondias

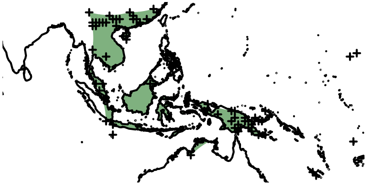

Stachyothyrsus

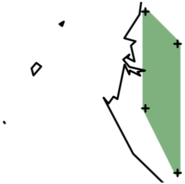

Staudtia

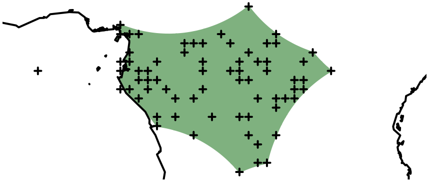

Stemonurus

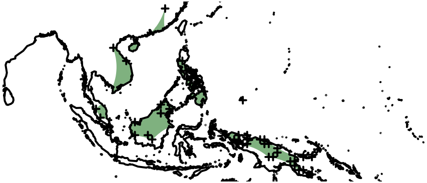

Sterculia

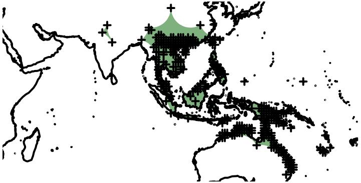

Sterculia

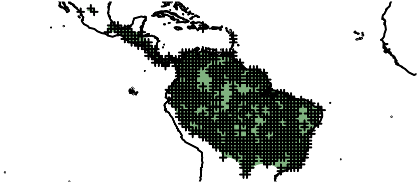

Sterculia

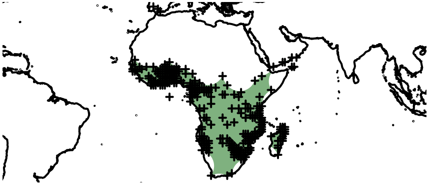

Strephonema

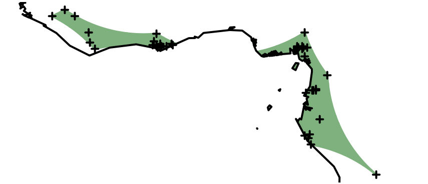

Strombosia

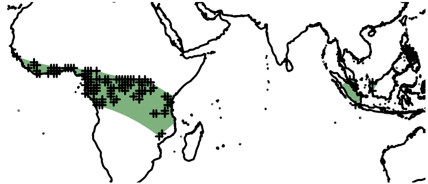

Strombosiopsis

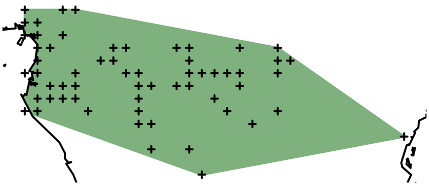

Stryphnodendron

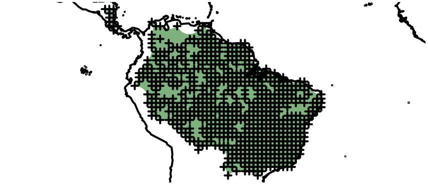

Swartzia

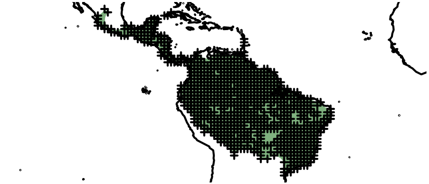

Symphonia

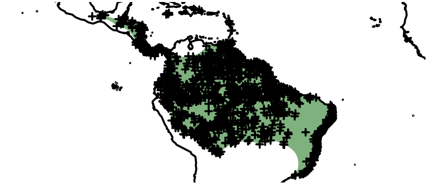

Symphonia

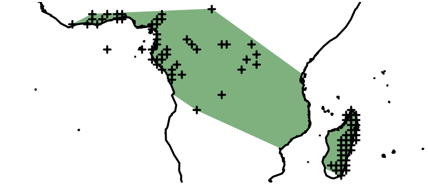

Symplocos

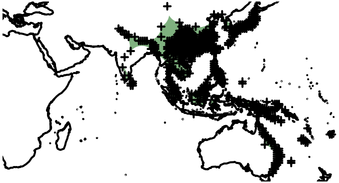

Symplocos

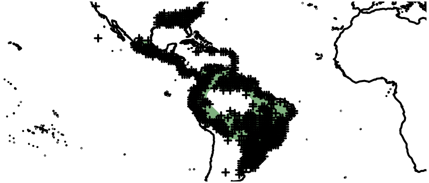

Synsepalum

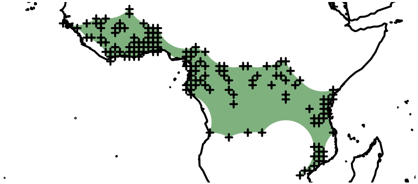

Syzygium

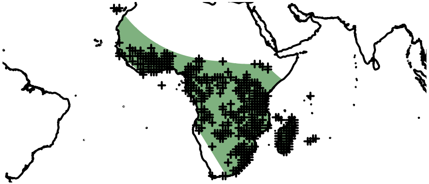

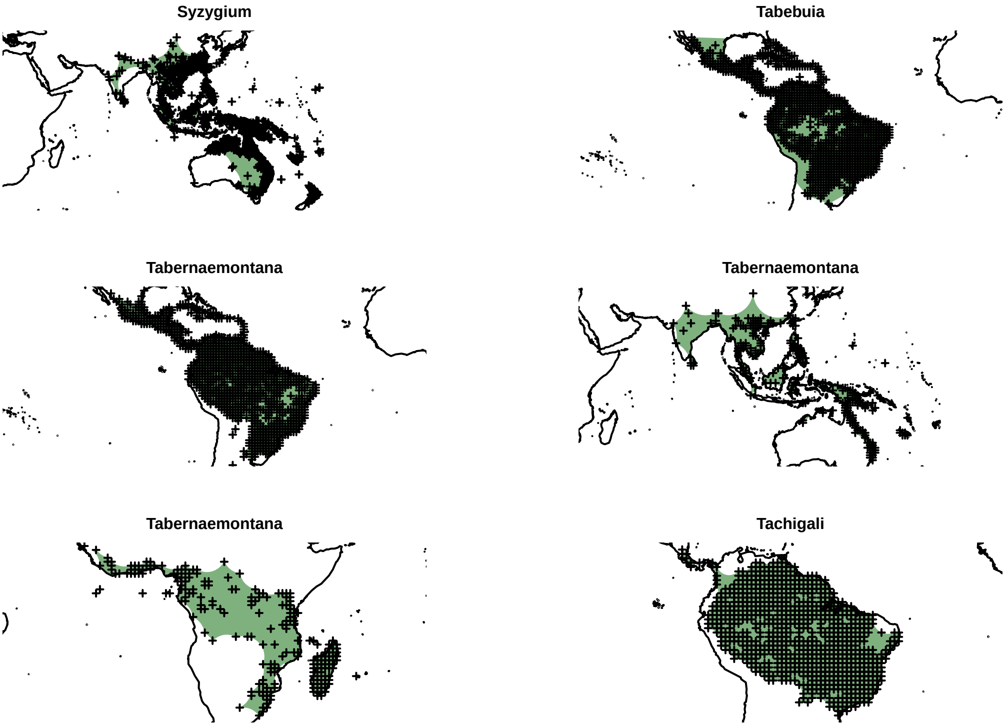
Syzygium
Tabebuia
Tabernaemontana
Tabernaemontana
Tabernaemontana
Tachigali

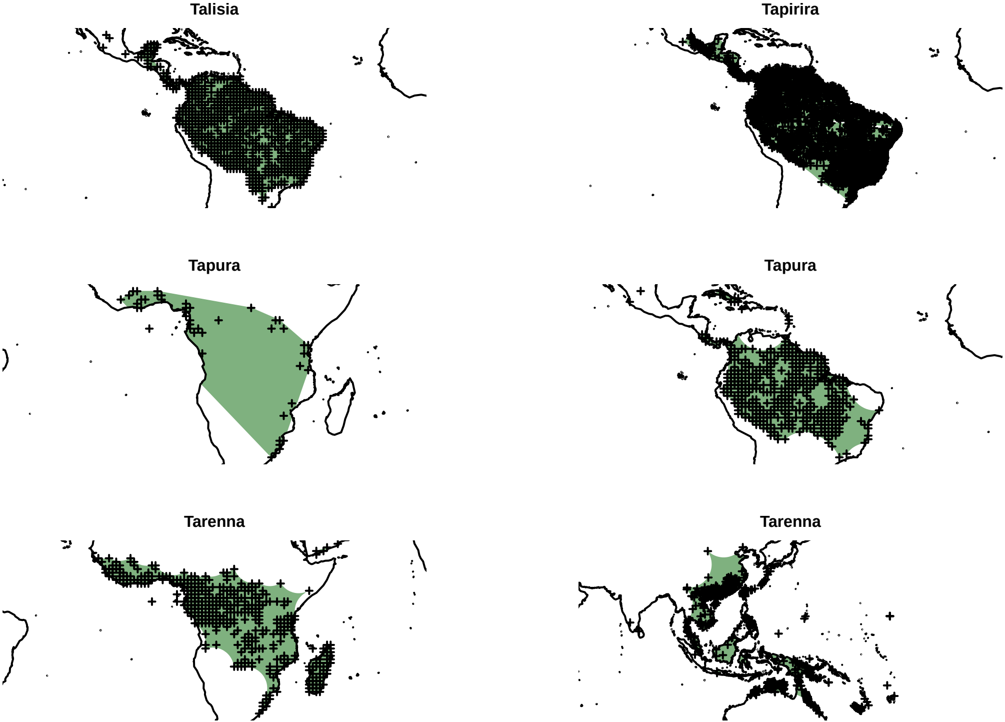
Talisia
Tapirira
Tapura
Tapura
Tarenna
Tarenna

Tarrietia

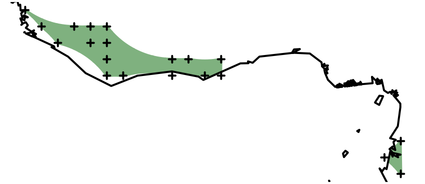

Teijsmanniodendron

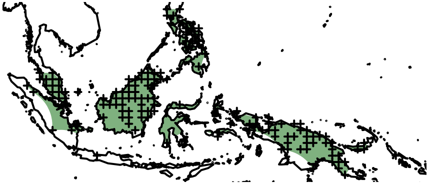

Terminalia

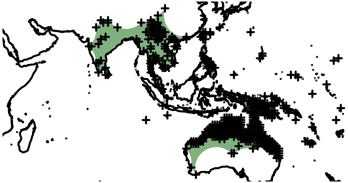

Terminalia

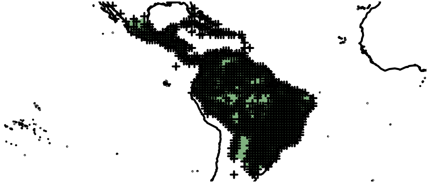

Terminalia

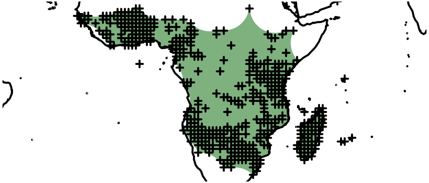

Ternstroemia

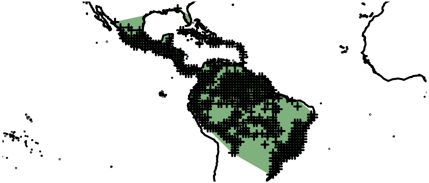

Ternstroemia

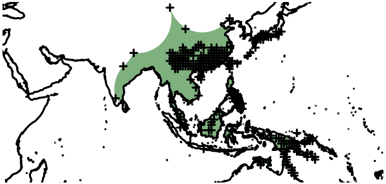

Tessmannia

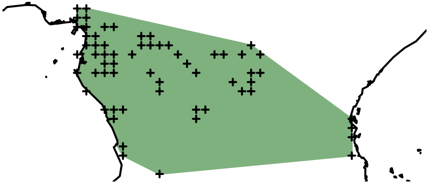

Tessmannia

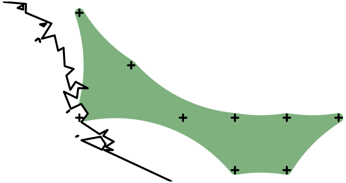

Tetraberlinia

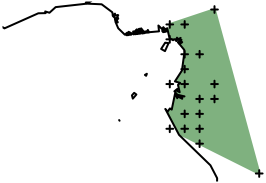

Tetragastris

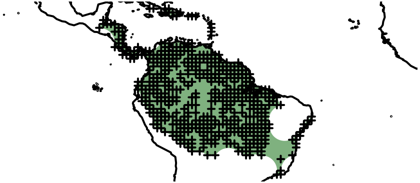

Tetrapleura

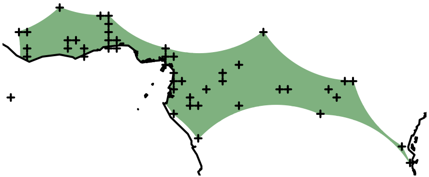

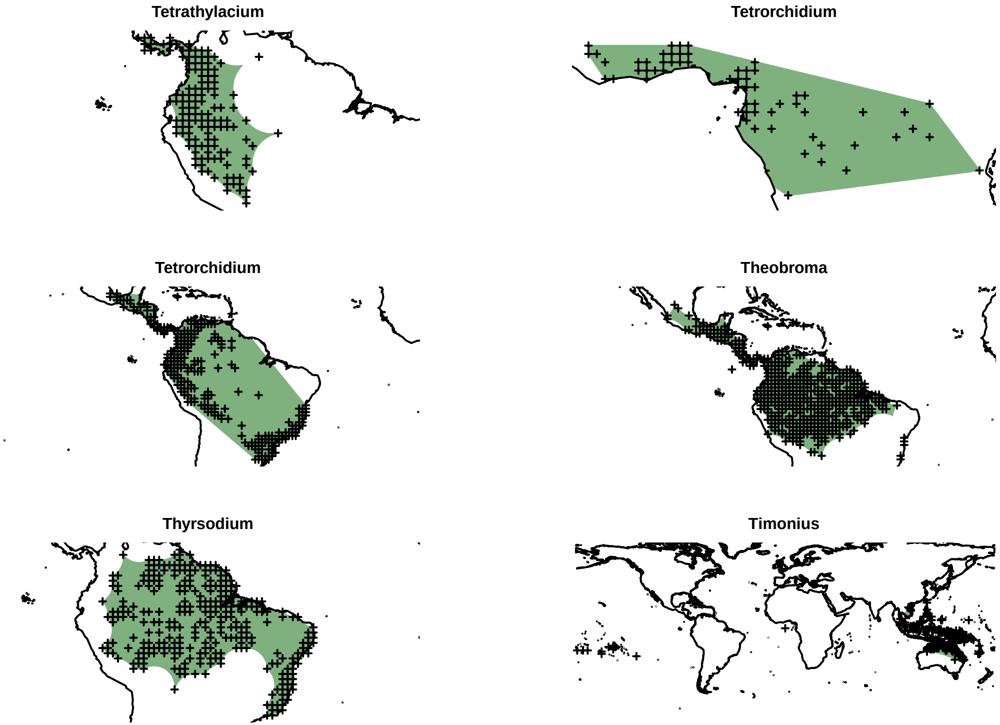
Tetrathylacium
Tetrorchidium
Tetrorchidium
Theobroma
Thyrsodium
Timonius

Tovomita

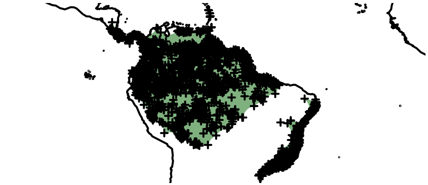

Trattinnickia

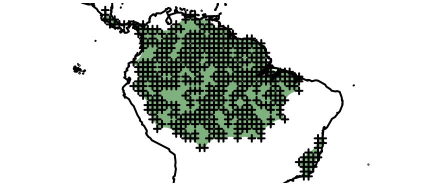

Treculia

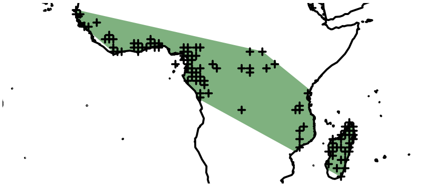

Trema

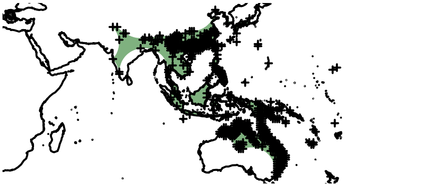

Trema

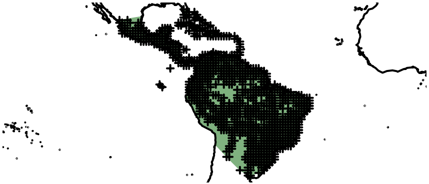

Trema

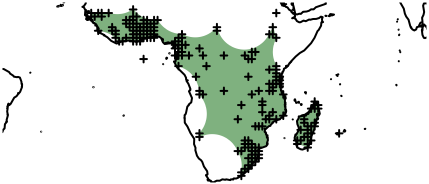

Supplement: Supplementary file 10 — Supplementary Information 10. [file 41598_2024_84367_MOESM10_ESM.pdf]
