# Supplementary material for: Large range sizes link fast life histories with high species richness across wet tropical tree floras
Source: Sci Rep. 2025 Feb 8;15:4695. doi: 10.1038/s41598-024-84367-3 (PMC11807110; doi:10.1038/s41598-024-84367-3)

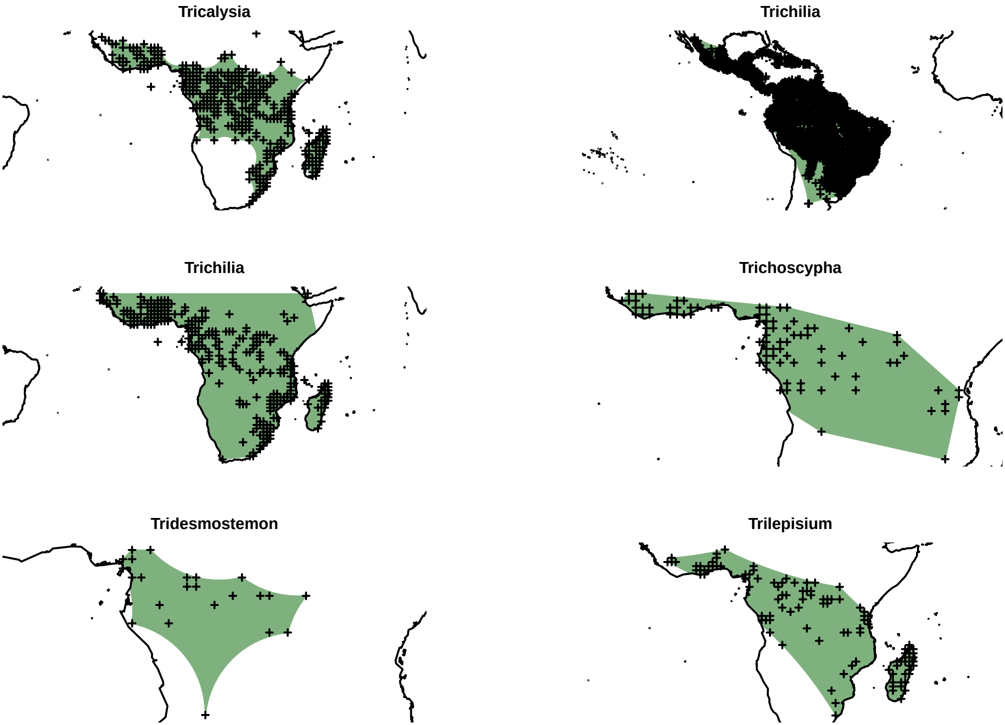
Tricalysia
Trichilia
Trichilia
Trichoscypha
Tridesmostemon
Trilepisium

Triplaris

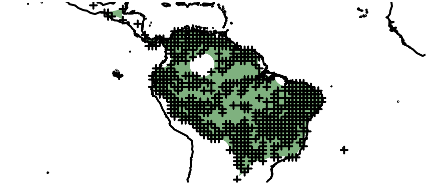

Triplochiton

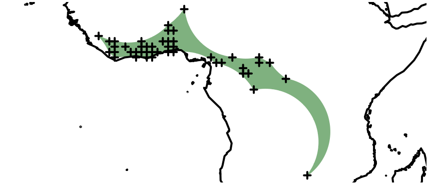

Tristania

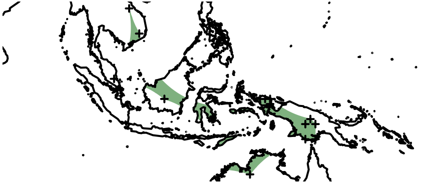

Tristania

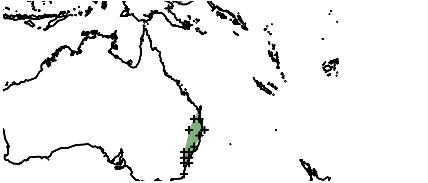

Tristaniopsis

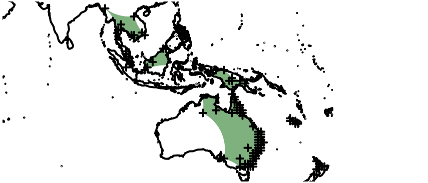

Trophis

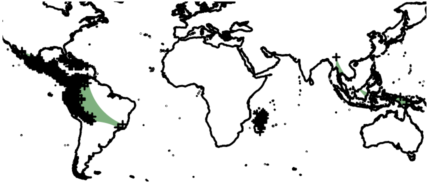

Turpinia

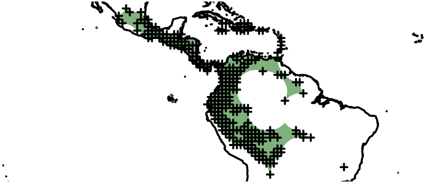

Turpinia

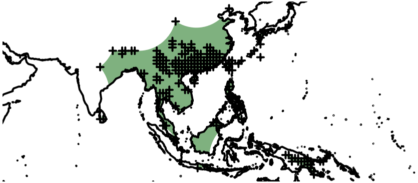

Turraeanthus

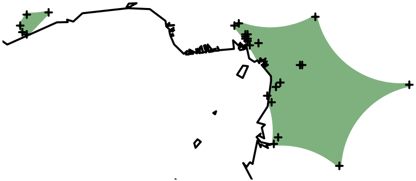

Uapaca

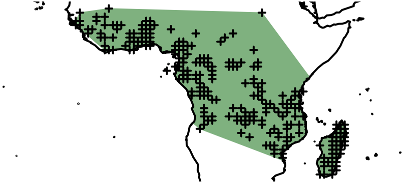

Unonopsis

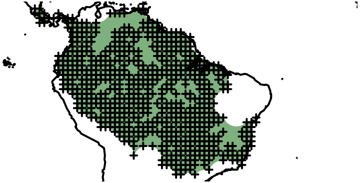

Uvariopsis

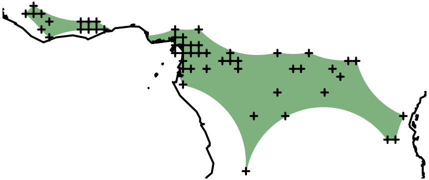

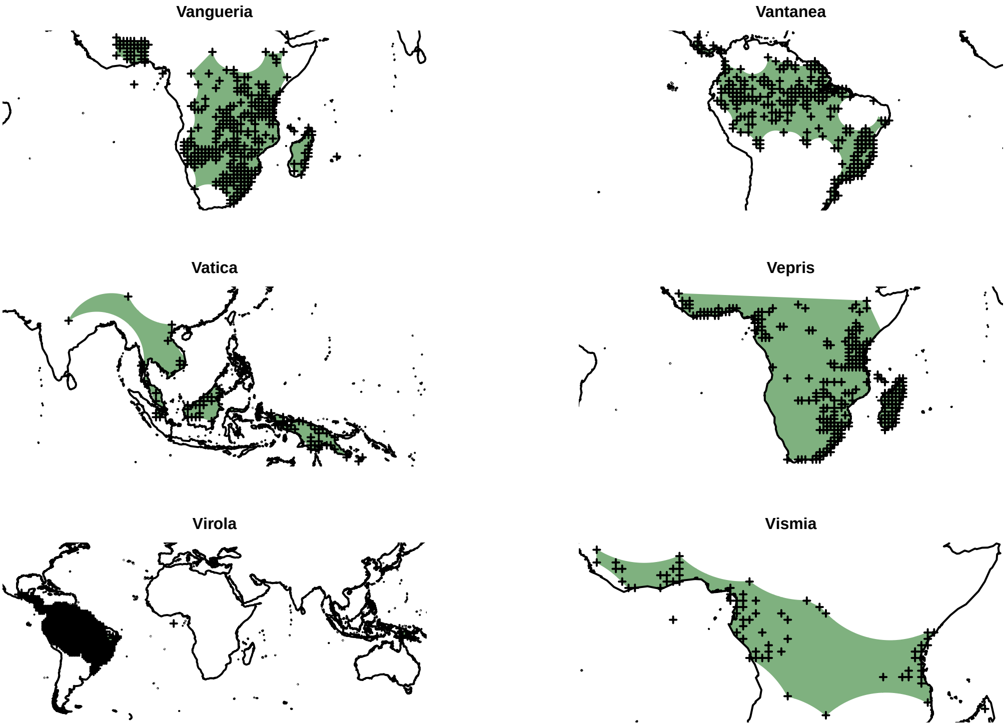
Vangueria
Vantanea
Vatica
Vepris
Virola
Vismia

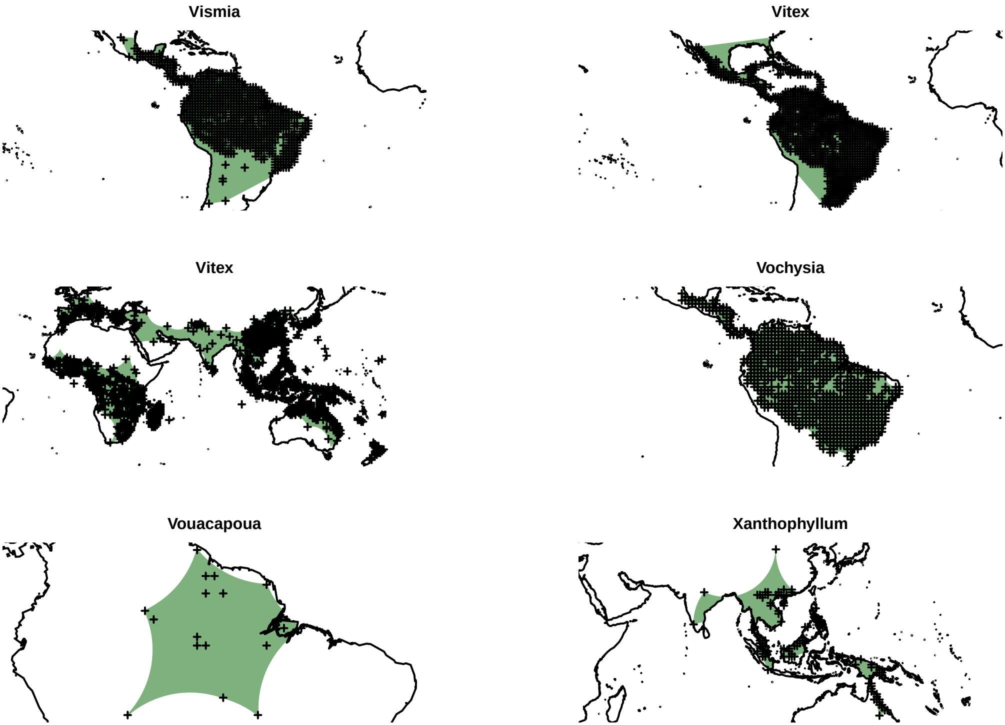
Vismia
Vitex
Vitex
Vochysia
Vouacapoua
Xanthophyllum

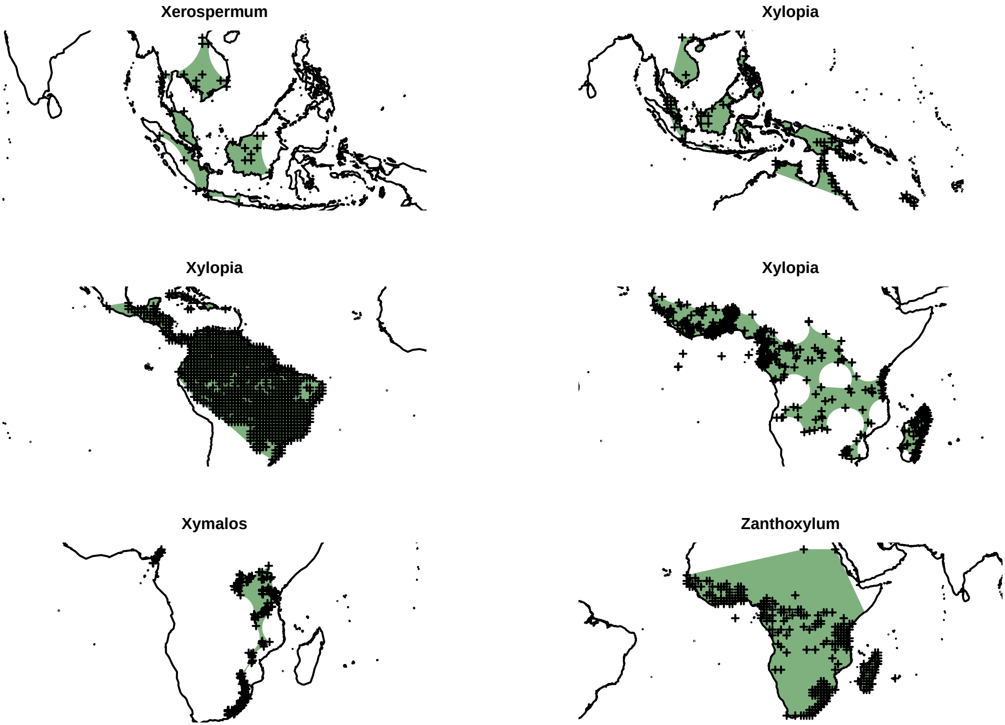
Xerospermum
Xylopia
Xylopia
Xylopia
Xymalos
Zanthoxylum

Zanthoxylum

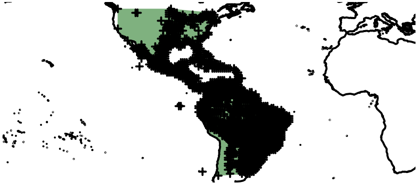

Zanthoxylum

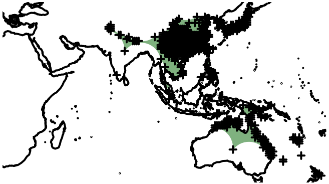

Zygia

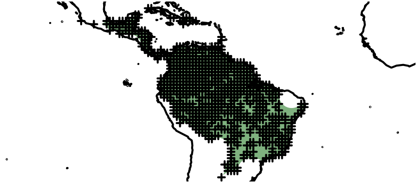

Supplement: Supplementary file 11 — Supplementary Information 11. [file 41598_2024_84367_MOESM11_ESM.pdf]
