# Supplementary material for: Large range sizes link fast life histories with high species richness across wet tropical tree floras
Source: Sci Rep. 2025 Feb 8;15:4695. doi: 10.1038/s41598-024-84367-3 (PMC11807110; doi:10.1038/s41598-024-84367-3)
Supplement: Supplementary file 12 — Supplementary Information 12. [file 41598_2024_84367_MOESM12_ESM.docx]

**Supplementary Information for ‘Large range sizes link fast demography with high species richness across wet tropical tree floras’**

by Timothy R. Baker^1*^, Stephen Adu-Bredu^2^, Kofi Affum-Baffoe^3^, Shin-ichiro Aiba^4^, Perpetra Akite^5^, Miguel Alexiades^6^, Everton Almeida^7^, Edmar Almeida de Oliveira^8^, Esteban Alvarez Davila^9^, Christian Amani^10,11^, Ana Andrade^12^, Luiz Aragao^13,14^, Alejandro Araujo-Murakami^15^, Eric Arets^16^, Luzmila Arroyo^15^, Peter Ashton^17^, Suspense A Averti Ifo^18^, Gerardo A. Aymard C.^19^, Michel Baisie^20^, William Balee^21^, Michael Balinga^22^, Lindsay F. Banin^23^, Olaf Banki^24,25^, Christopher Baraloto^26,27^, Jorcely Barroso^28^, Jean-Francois Bastin^29^, Hans Beeckman^30^, Serge Begne^31^, Natacha Nssi Bengone^32^, Nicholas Berry^33^, Wemo Betian^34^, Vincent Bezard^35^, Lilian Blanc^36^, Pascal Boeckx^37^, Damien Bonal^38^, Frans Bongers^39^, Francis Q Brearley^40^, Roel Brienen^1^, Foster Brown^41^, Musalmah Bt. Nasaradin^42^, Benoit Burban^43^, David F.R.P. Burslem^44^, Plinio Camargo^45^, Jose Luis Camargo^12^, Wendeson Castro^46^, Carlos Ceron^47^, Victor Chama Moscoso^48^, Colin Chapman^49^, Jerome Chave^50^, Eric Chezeaux^51^, Murray Collins^52,53^, James Comiskey^54,55^, David Coomes^56^, Fernando Cornejo Valverde^57^, Flavia R.C. Costa^58^, Aida Cuni-Sanchez^59,60^, Lola da Costa^61^, Douglas C Daly^6^, Martin Dančák^62^, Armandu Daniels^63^, Greta Dargie^1^, Stuart Davies^64^, Charles De Canniere^66^, Thales de Haulleville^30^, Jhon del Aguila Pasquel^65^, Geraldine Derroire^67^, Kyle G. Dexter^52,68^, Tony Di Fiore^69^, Marie-Noel Djuikouo K.^70^, Jean-Louis Doucet^71^, Vincent Droissart^72^, Gerald Eilu^73^, Thaise Emillio^74^, Julien Engel^72^, Bocko Yannick Enock^18^, Fidele Evouna Ondo ^75^, Corneille Ewango^76^, Sophie Fauset^77^, Ted R. Feldpausch^13^, Muhammad Fitriadi^78^, Gerardo Flores Llampazo^65^, Ernest G Foli^2^, Gabriella Fredriksson^79,80^, David R Galbraith^1^, Martin Gilpin^1^, Emanuel Gloor^1^, Christelle Gonmadje^81^, Rene Guillen Villaroel^15^, Jefferson Hall ^82^, Keith C Hamer^83^, Alan Hamilton^84^,^85^, Olivier Hardy^86^, Terese Hart^87,88^, Radim Hedl^89,90^, Rafael Herrera^91^, Niro Higuchi^92^, Claude Marcel Hladik^93^, Eurídice Honorio Coronado^94^, Isau Huamantupa-Chuquimaco^95^, Walter Huaraca Huasco^48^, Wannes Hubau^30,96^, Muhammad Idhamsyah^97^, Sascha A Ismail^98^, Kath Jeffery^99^, Eliana Jimenez^100^, Tommaso Jucker^101^, Elizabeth Kearsley^102^, Lip Khoon Kho^103^, Timothy Killeen^104^, Kanehiro Kitayama^105^, William Laurance^106^, Susan Laurance^106^, Miguel Leal^107^, Simon L. Lewis^1,108^, Stanislav Lhota^109^, Jeremy Lindsell^111,112^, Gabriela Lopez-Gonzalez^1^, Jon Lovett^1,113^, Richard Lowe, William E. Magnusson^58^, Jean-Remy Makana^76^, Yadvinder Malhi^114^, Beatriz Marimon^8^, Ben Hur Marimon Junior^8^, Andrew Marshall^60^, Colin Maycock^115^, Faustin Mbayu^116^, Casimiro Mendoza^117^, Irina Mendoza Polo^118^, Faizah Metali^119^, Vianet Mihindou^75^,^32^, Abel Monteagudo-Mendoza^48^, Sam Moore^114^, Patrick Mucunguzi^5^, Jacques Mukinzi^120,121^, Pantaleo Munishi^122^, Laszlo Nagy^123^, Petrus Naisso^36^, David Neill^124^, Adriano Nogueira Lima^58^, Percy Nunez Vargas^48^, Lucas Ojo^125^, Walter Palacios^126^, Nadir Pallqui Camacho^48^, Alexander Parada Gutierrez^15^, Julie Peacock^1^, Kelvin S.-H. Peh^127^, Antonio Pena Cruz^128^, Colin Pendry^68^, Toby Pennington^13,68^,Maria Cristina Penuela-Mora ^129^, Pascal Petronelli^130^, Oliver L. Phillips^1^, Georgia Pickavance^1^, G., John Pipoly^131^,^132^, Nigel Pitman^133^, Axel Dalberg Poulsen^134,68^, Ghillean T. Prance, Adriana Prieto^135^, Richard B. Primack^136^, Lan Qie^137^, Simon A. Queenborough^138,139^, Carlos Quesada^58^, Freddy Ramirez Arevalo^140^, Hirma Ramirez-Angulo^141^, Jan Reitsma^142^, Maxime Réjou-Méchain^72^, Anand Roopsind^143^, Francesco Rovero^144,145^, Ervan Rutishauser^146^, Kamariah Abu Salim^119^, Rafael Salomao^147^, Ismayadi Samsoedin^148^, Muhd Shahruney Saparudin^119^, Juliana Schietti^58^, Ricardo A. Segovia^149^, Julio Serrano^141^, Rafizah Serudia^119^, Douglas Sheil^39^, Natalino Silva^150^, Javier Silva Espejo^151^, Marcos Silveira^152^, Murielle Simo-Droissart^153^, James Singh^154^, Bonaventure Sonké^153^, Juliana Stropp^155^, Rahayu Sukri^119^, Terry Sunderland^156,157^, Martin Svátek^158^, Michael Swaine^44^, Hermann Taedoumg^159,160^, Joey Talbot^1^, Sylvester Tan^161,162^, James Taplin^163,164^, David Taylor^165^, Hans ter Steege^25^, John Terborgh^166^, Armando Torres-Lezama^141^, John Tshibamba Mukendi^116^, Darlington Tuagben ^167^, Peter van de Meer^168^, Geertje van der Heijden^169^, Peter van der Hout ^170^, Mark van Nieuwstadt^25^, Bert van Ulft^171^, Rodolfo Vasquez Martinez^172^, Ronald Vernimmen^173^, Barbara Vinceti^174^, Simone Vieira^175^, Ima Celia Guimaries Vieira^147^, Emilio Vilanova Torre^141,176^, Jason Vleminckx^177,178^, Lee White^179,32^, Simon Willcock^180,181^, Mathew Williams^52^, John T. Woods^182^, Tze Leong Yao^42^, Ishak Yassir^183^, Roderick Zagt^184^, Lise Zemagho^153^

**Figure S1**. Structural equation models from pSEM analysis showing the relationships between traits, range size and species richness for the core dataset of 288 genera that solely comprise tropical trees from the lowland wet tropics. Standardised effect sizes shown for significant relationships and arrow width is proportional to the standardised effect size. Non-significant relationships are shown with grey dotted lines.

**Figure S2.** Phylogenetic relationships of species richness, high mortality rates and small maximum size for 463 genera of tropical trees. Tip circle size is proportional to the species richness of each genus. Coloured bars indicate genera with high mortality rates and/or small maximum size. High mortality rates are classified as >2% a^-1^; small maximum size classified as <30 cm diameter. Bar colour indicates the biogeographic setting of each genus and named genera are those that share both high mortality rates and small maximum size; the distributed pattern of names illustrates that the association between high mortality rates and small maximum size is found across the phylogeny.

**Figure S3.** Relationship between (a) species richness and maximum size, (b) range size and the presence of dioecy, and (c) mortality rate and the presence of dioecy for 288 genera that solely comprise tropical trees. Regression lines show significant relationships, and asterisks indicate significant differences, based on univariate GLS models implemented within pSEM models and accounting for the phylogenetic covariance structure within the data.

**Figure S4.** Phylogenetic relationships of species richness, high mortality rates and presence of dioecy for 463 genera of tropical trees. Tip circle size is proportional to the species richness of each genus. Coloured bars indicate genera with high mortality rates and/or that contain dioecious species. High mortality rates are classified as >2% a^-1^. Bar colour indicates the biogeographic setting of each genus and named genera are those that share both high mortality rates and the presence of dioecy.

**Figure S5.** The distribution of the number of herbarium records used to create range maps for each of the 463 genera of tropical trees, classified by biogeographic setting.

**Figure S6** Variation in (A) maximum diameter, (B) annual mortality rate, (C) range size, (D) species richness and (E) the presence of dioecy among 463 genera containing tropical trees in wet, lowland forest in the Americass, Africa and Asia, and across multiple continents. Distributions of mortality rates, range size and species richness are shown as log transformed values.


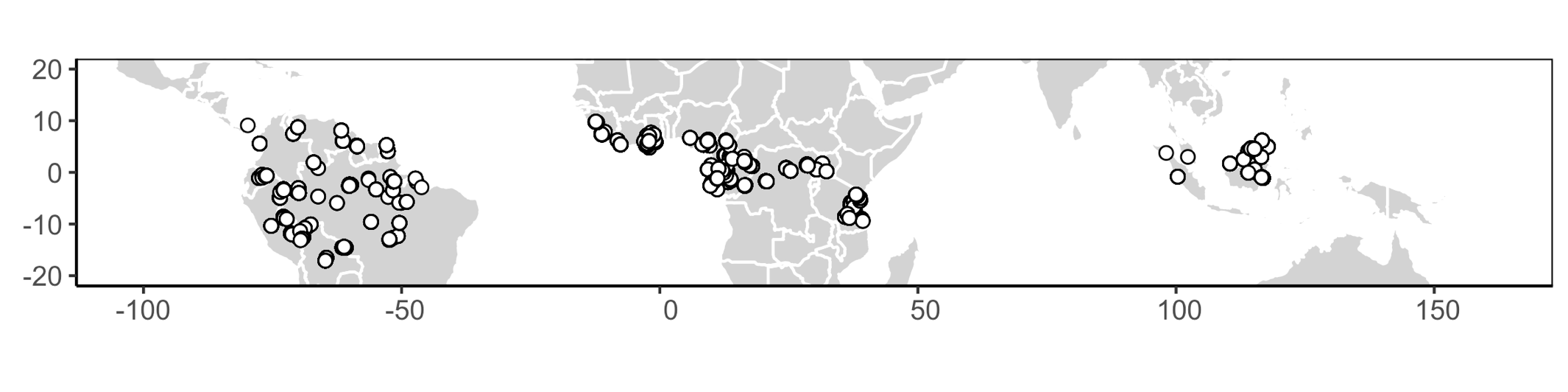


**Figure S7.** Location of 655 plots used to calculate variation in maximum size and mortality rates among genera.

**Figure S8.** Distribution of census intervals for all trees used to calculate mortality rates for 463 genera of tropical trees in this study.

**Figure S9.** Phylogenetic relationships for the 463 genera of tropical trees used on this study. The ten families with the greatest genus richness are labelled and shown in colour. Circle size of the tip labels is proportional to species richness of each genus.

**Figure S10 (see additional files).** Maps of extent of occupancy for 463 genera containing tropical trees based on herbarium records from GBIF. Crosses indicate underlying records and green shaded area indicate area of alpha hull.

**Figure S11.** Relationship between species richness and stem age for 463 genera of tropical trees. There are no significant relationships between these variables for any biogeographical settings. Note that the range of the y axis differs among plots.

**Table S1**. Generalised linear squares models, incorporating phylogenetic errors, for relationships between traits, range size and species richness and their interaction with biogeographical setting for 463 genera of tropical trees. Range size was analysed as a function of biogeographic setting, mortality rate, maximum size and presence of dioecy; species richness was analysed as a function of range size in addition to these terms. Biogeographical setting defined as genus being restricted to the Americass, Asia, Africa or occurring in multiple continents. All continuous variables were log transformed prior to analysis and the significance of terms was calculated using type 3 errors; see Materials and Methods for further details.

|  | Response variable | | | | | |
| --- | --- | --- | --- | --- | --- | --- |
|  | Range size | | | Species richness | | |
| Predictors | df | Chi sq | p | df | Chi sq | p |
| Range size |  |  |  | **1** | **62.0** | **<0.001** |
| Biogeo. setting | **3** | **28.9** | **<0.001** | **1** | **27.5** | **<0.001** |
| Mortality rate | **1** | **9.2** | **<0.005** | 3 | 1.33 | ns |
| Max. size | 1 | 0.0 | ns | **1** | **14.9** | **<0.001** |
| Dioecious? | 1 | 0.5 | ns | 1 | 0.16 | ns |
| Distribution: Range size |  |  |  | **3** | **27.6** | **<0.001** |
| Distribution: Mortality rate | 3 | 1.3 | ns | 3 | 2.6 | ns |
| Distribution: Max. size | **3** | **7.8** | **<0.05** | 3 | 4.5 | ns |
| Distribution: Dioecious? | 3 | 4.1 | ns | 3 | 4.5 | ns |

**Table S2**. Generalised linear squares models, incorporating phylogenetic errors, for relationships between traits, mean species range size and species richness for 105 genera of Amazonian trees. Mean species range size was analysed as a function of mortality rate, maximum size, seed mass, dispersal mode and the presence of dioecy; species richness was analysed as a quadratic function of mean species range size in addition to these terms. All continuous variables were log transformed prior to analysis and the significance of terms was calculated using type 3 errors.

|  | Response variable | | | | | |
| --- | --- | --- | --- | --- | --- | --- |
|  | Mean species range size | | | Species richness | | |
| Predictors | df | Chi sq | p | df | Chi sq | p |
| Mean species range size |  |  |  | **1** | **8.5** | **<0.005** |
| (Mean species range size)^2^ |  |  |  | **1** | **18.1** | **<0.001** |
| Population size |  |  |  | **1** | **36.8** | **<0.001** |
| Mortality rate | **1** | **8.6** | **<0.005** | 1 | 1.5 | ns |
| Max. size | 1 | 0.0 | ns | 1 | 1.7 | ns |
| Dioecious? | 1 | 2.6 | ns | 1 | 2.6 | ns |
| Seed mass | 1 | 0.5 | ns | 3 | 0.3 | ns |
| Dispersal mode | 1 | 0.0 | ns | 3 | 0.0 | ns |

**Table S3.** Plot codes, countries and leadership teams associated with each plot used to calculate maximum size and mortality rates of genera in this study.

| **Plot** | **Country** | **Leadership team** |
| --- | --- | --- |
| AGP-01 | COLOMBIA | Jimenez, Lloyd, Phillips, Prieto, Rudas, Vasquez Martinez |
| AGP-02 | COLOMBIA | Jimenez, Lloyd, Phillips, Prieto, Rudas |
| ALF-01 | BRAZIL | Almeida de Oliveira, Feldpausch, Lloyd, Marimon, Marimon Junior, Phillips, Quesada |
| ALF-02 | BRAZIL | Almeida de Oliveira, Feldpausch, Lloyd, Marimon, Marimon Junior, Phillips |
| ALM-01 | PERU | Brienen, Monteagudo-Mendoza, Phillips, Pitman, Terborgh |
| ALP-30 | PERU | Baker, Brienen, del Aguila Pasquel, Monteagudo-Mendoza, Phillips, Silva Espejo, Vasquez Martinez |
| ALP-40 | PERU | Brienen, del Aguila Pasquel, Monteagudo-Mendoza, Phillips, Ramirez Arevalo |
| AMA-02 | COLOMBIA | Alvarez Davila, Mendoza Polo, Phillips, Serna |
| AND-01 | BRUNEI DARUSSALAM | Davies, Lewis, Phillips, Qie, Salim, Saparudin |
| ANK-01 | GHANA | Adu-Bredu, Malhi, Moore |
| ANK-02 | GHANA | Adu-Bredu, Malhi, Moore |
| ANK-03 | GHANA | Adu-Bredu, Malhi, Moore |
| ASN-02 | GHANA | Affum-Baffoe, Lewis, Phillips |
| ASN-04 | GHANA | Affum-Baffoe, Hubau, Lewis, Phillips |
| ASR-01 | BRAZIL | Balee, Campbell |
| ASU-88 | GHANA | Affum-Baffoe, Baker, Fauset, Feldpausch, Lewis |
| ASU-99 | GHANA | Affum-Baffoe, Baker, Fauset, Feldpausch, Lewis, Phillips |
| BAC-01 | VENEZUELA | D'Jesus, Phillips, Ramirez-Angulo, Serrano, Torres-Lezama, van der Heijden, Vilanova Torre |
| BAC-02 | VENEZUELA | D'Jesus, Phillips, Ramirez-Angulo, Serrano, Torres-Lezama, van der Heijden, Vilanova Torre |
| BAC-03 | VENEZUELA | D'Jesus, Phillips, Ramirez-Angulo, Serrano, Torres-Lezama, van der Heijden, Vilanova Torre |
| BAC-04 | VENEZUELA | Chama Moscoso, D'Jesus, Phillips, Ramirez-Angulo, Serrano, Torres-Lezama, van der Heijden, Vilanova Torre |
| BAC-05 | VENEZUELA | Phillips, Ramirez-Angulo, Serrano, Torres-Lezama, van der Heijden, Vilanova Torre |
| BAC-06 | VENEZUELA | Phillips, Ramirez-Angulo, Serrano, Torres-Lezama, van der Heijden, Vilanova Torre |
| BAD-01 | BRUNEI | Banin, Davies, Lewis, Phillips, Qie, Salim, Serudia, |
| BBR-14 | GHANA | Affum-Baffoe, Baker, Fauset, Feldpausch, Lewis |
| BBR-16 | GHANA | Affum-Baffoe, Baker, Fauset, Feldpausch, Lewis |
| BBR-17 | GHANA | Affum-Baffoe, Baker, Fauset, Feldpausch, Lewis |
| BCI | PANAMA | CTFS-ForestGeo |
| BDF-01 | BRAZIL | Andrade, Camargo, Laurance, Lovejoy |
| BDF-03 | BRAZIL | Andrade, Camargo, Laurance, Lovejoy |
| **Plot** | **Country** | **Leadership team** |
| BDF-04 | BRAZIL | Andrade, Camargo, Laurance, Lovejoy |
| BDF-05 | BRAZIL | Andrade, Camargo, Laurance, Lovejoy |
| BDF-06 | BRAZIL | Andrade, Camargo, Laurance, Lovejoy |
| BDF-07 | BRAZIL | Andrade, Camargo, Laurance, Lovejoy |
| BDF-08 | BRAZIL | Andrade, Camargo, Laurance, Lovejoy |
| BDF-09 | BRAZIL | Andrade, Camargo, Laurance, Lovejoy |
| BDF-10 | BRAZIL | Andrade, Camargo, Laurance, Lovejoy |
| BDF-11 | BRAZIL | Andrade, Camargo, Laurance, Lovejoy |
| BDF-12 | BRAZIL | Andrade, Camargo, Laurance, Lovejoy |
| BDF-13 | BRAZIL | Andrade, Camargo, Laurance, Lovejoy |
| BDF-14 | BRAZIL | Andrade, Camargo, Laurance, Lovejoy |
| BEE-01 | BOLIVIA | Arroyo, Parada Gutierrez, Peacock, Phillips |
| BEE-05 | BOLIVIA | Arroyo, Parada Gutierrez, Peacock, Phillips |
| BEL-01 | BRUNEI DARUSSALAM | Poulsen |
| BEL-03 | BRUNEI DARUSSALAM | Dancak, Hedl, Lewis, Maycock, Metali , Phillips, Qie, Sukri, Sv_tek |
| BEL-04 | BRUNEI DARUSSALAM | Dancak, Hedl, Lewis, Maycock, Metali , Phillips, Qie, Sukri, Sv_tek |
| BEL-11 | BRUNEI DARUSSALAM | Lewis, Maycock, Pendry, Phillips, Qie |
| BEL-12 | BRUNEI DARUSSALAM | Lewis, Maycock, Pendry, Phillips, Qie |
| BEL-13 | BRUNEI DARUSSALAM | Lewis, Maycock, Pendry, Phillips, Qie |
| BEL-14 | BRUNEI DARUSSALAM | Lewis, Maycock, Pendry, Phillips, Qie |
| BEL-15 | BRUNEI DARUSSALAM | Lewis, Maycock, Pendry, Phillips, Qie |
| BEL-16 | BRUNEI DARUSSALAM | Lewis, Maycock, Pendry, Phillips, Qie |
| BEL-17 | BRUNEI DARUSSALAM | Maycock, Pendry |
| BEL-18 | BRUNEI DARUSSALAM | Maycock, Pendry |
| BEL-19 | BRUNEI DARUSSALAM | Lewis, Maycock, Pendry, Phillips, Qie |
| BFI-03 | GHANA | Feldpausch, Lloyd, Phillips |
| BFI-04 | GHANA | Feldpausch, Lloyd, Phillips |
| BIL-01 | GABON | Doucet, Paget |
| BIL-02 | GABON | Doucet, Paget |
| **Plot** | **Country** | **Leadership team** |
| BIL-03 | GABON | Doucet, Paget |
| BIL-04 | GABON | Doucet, Paget |
| BIS-01 | CAMEROON | Alaman, Begne, Kamdem, Lewis, Sonke |
| BIS-02 | CAMEROON | Begne, Kamdem, Lewis, Sonke |
| BIS-03 | CAMEROON | Begne, Kamdem, Lewis, Sonke |
| BIS-04 | CAMEROON | Begne, Kamdem, Lewis, Sonke |
| BIS-05 | CAMEROON | Begne, Kamdem, Lewis, Sonke |
| BIS-06 | CAMEROON | Begne, Kamdem, Lewis, Sonke |
| BKO-01 | MALAYSIA | Ashton, Khoon, Lewis, Phillips, Primack, Qie, Tan |
| BKO-02 | MALAYSIA | Ashton, Lewis, Phillips, Primack, Qie, Tan |
| BKO-03 | MALAYSIA | Ashton, Lewis, Phillips, Primack, Qie, Tan |
| BKO-04 | MALAYSIA | Ashton, Lewis, Phillips, Primack, Qie, Tan |
| BNT-05 | BRAZIL | Higuchi |
| BNT-06 | BRAZIL | Higuchi |
| BNT-07 | BRAZIL | Higuchi |
| BOB-01 | GHANA | Affum-Baffoe, Foli, Malhi, Moore |
| BOB-02 | GHANA | Affum-Baffoe, Foli, Malhi, Moore |
| BOB-03 | GHANA | Affum-Baffoe, Foli, Malhi, Moore |
| BOB-04 | GHANA | Affum-Baffoe, Foli, Malhi, Moore |
| BOB-05 | GHANA | Affum-Baffoe, Foli, Malhi, Moore |
| BOB-06 | GHANA | Affum-Baffoe, Foli, Malhi, Moore |
| BOG-01 | ECUADOR | Baker, Brienen, Di Fiore, Lloyd, Monteagudo-Mendoza, Neill, Pena Cruz, Phillips, Pitman |
| BOG-02 | ECUADOR | Baker, Brienen, Di Fiore, Lloyd, Monteagudo-Mendoza, Neill, Pena Cruz, Phillips, Pitman |
| BOK-01 | CONGO-BRAZZAVILLE | Begne, Lewis, Sonke |
| BOK-02 | CONGO-BRAZZAVILLE | Begne, Lewis, Sonke |
| BOK-03 | CONGO-BRAZZAVILLE | Begne, Lewis, Sonke |
| BOR-05 | GHANA | Affum-Baffoe, Baker, Fauset, Feldpausch, Lewis |
| BOR-06 | GHANA | Affum-Baffoe, Baker, Fauset, Feldpausch, Lewis |
| BRT-01 | INDONESIA | Lewis, Phillips, Qie, Rutishauser |
| BUD-07 | UGANDA | Eggeling, Sheil |
| BUL-01 | INDONESIA | Lewis, Nagy, Phillips, Proctor, Qie |
| BUL-02 | INDONESIA | Nagy, Proctor |
| BUL-03 | INDONESIA | Lewis, Nagy, Phillips, Proctor, Qie |
| BUL-04 | INDONESIA | Nagy, Proctor |
| BUL-05 | INDONESIA | Nagy, Proctor |
| BUL-06 | INDONESIA | Nagy, Proctor |
| **Plot** | **Country** | **Leadership team** |
| BUL-07 | INDONESIA | Lewis, Nagy, Phillips, Proctor, Qie |
| BUL-08 | INDONESIA | Lewis, Nagy, Phillips, Proctor, Qie |
| BUL-10 | INDONESIA | Brearley, Lewis, Nagy, Phillips, Proctor, Qie, Vernimmen |
| BUL-16 | INDONESIA | Brearley, Kidd, Proctor |
| BUL-17 | INDONESIA | Brearley, Kidd, Proctor |
| BUL-18 | INDONESIA | Brearley, Kidd, Proctor |
| BUL-19 | INDONESIA | Brearley, Mirmanto, Proctor |
| BUL-20 | INDONESIA | Brearley, Mirmanto, Proctor |
| BUL-21 | INDONESIA | Brearley, Mirmanto, Proctor |
| BUL-22 | INDONESIA | Brearley, Lewis, Mirmanto, Phillips, Proctor, Qie |
| BUL-25 | INDONESIA | Brearley, Lewis, Mirmanto, Phillips, Proctor, Qie |
| BUL-30 | INDONESIA | Brearley, Lewis, Mirmanto, Phillips, Proctor, Qie |
| BUL-31 | INDONESIA | Brearley, Mirmanto, Proctor |
| BUL-33 | INDONESIA | Brearley, Mirmanto, Proctor |
| BUL-38 | INDONESIA | Lewis, Phillips, Proctor, Qie |
| BUL-40 | INDONESIA | Lewis, Nagy, Phillips, Proctor, Qie |
| BUL-56 | INDONESIA | Lewis, Nagy, Phillips, Proctor, Qie |
| CAI-05 | VENEZUELA | D'Jesus, Phillips, Ramirez-Angulo, Torres-Lezama, van der Heijden, Veillon, Vilanova Torre |
| CAI-06 | VENEZUELA | D'Jesus, Phillips, Ramirez-Angulo, Torres-Lezama, van der Heijden, Veillon, Vilanova Torre |
| CAP-09 | GHANA | Affum-Baffoe, Hubau, Lewis, Phillips |
| CAP-10 | GHANA | Affum-Baffoe, Hubau, Lewis, Phillips |
| CAX-01 | BRAZIL | Almeida, Baker, Lima, Phillips |
| CAX-02 | BRAZIL | Almeida, Baker, Phillips |
| CAX-06 | BRAZIL | Almeida, Aragao, da Costa, Malhi, Phillips |
| CEB-01 | GABON | Doucet |
| CEB-02 | GABON | Doucet |
| CEB-03 | GABON | Doucet |
| CEB-04 | GABON | Doucet |
| CHO-01 | BOLIVIA | Arroyo, Killeen, Lloyd, Patino, Phillips |
| CNG-01 | VENEZUELA | Gentry, Phillips |
| CPP-01 | BRAZIL | Vieira |
| CPP-02 | BRAZIL | Vieira |
| CRG-01 | BRAZIL | Da Silva, Oliviera, Rosa |
| CRP-02 | BOLIVIA | Araujo-Murakami, Arroyo, Brienen, Guillen Villaroel, Killeen, Lloyd, Patino, Phillips |
| CUZ-01 | PERU | Baker, Gentry, Monteagudo-Mendoza, Nunez Vargas, Phillips, Talbot, Vasquez Martinez |
| CUZ-02 | PERU | Baker, Gentry, Monteagudo-Mendoza, Nunez Vargas, Phillips, Talbot, Vasquez Martinez |
| **Plot** | **Country** | **Leadership team** |
| CUZ-03 | PERU | Baker, Gentry, Monteagudo-Mendoza, Phillips, Talbot, Vasquez Martinez |
| CUZ-04 | PERU | Baker, Gentry, Monteagudo-Mendoza, Nunez Vargas, Phillips, Vasquez Martinez |
| CVL-01 | LIBERIA | Daniels, Hubau, Lewis, Phillips, Woell, Woods |
| CVL-08 | LIBERIA | Woell |
| CVL-10 | LIBERIA | Daniels, Lewis, Woell, Woods |
| CVL-11 | LIBERIA | Daniels, Hubau, Lewis, Phillips, Woell, Woods |
| DAD-03 | GHANA | Affum-Baffoe, Hubau, Lewis, Phillips |
| DAD-04 | GHANA | Affum-Baffoe, Hubau, Lewis, Phillips |
| DAN-01 | MALAYSIA | Berry, Hamer, Lewis, Phillips, Qie |
| DAN-02 | MALAYSIA | Berry, Hamer, Lewis, Phillips, Qie |
| DAN-03 | MALAYSIA | Berry, Hamer, Lewis, Phillips, Qie |
| DJA-01 | CAMEROON | Lewis, Sonke |
| DJA-02 | CAMEROON | Lewis, Sonke |
| DJA-03 | CAMEROON | Lewis, Sonke |
| DJA-04 | CAMEROON | Lewis, Sonke |
| DJA-05 | CAMEROON | Lewis, Sonke |
| DJA-07 | CAMEROON | Lewis, Sonke |
| DJA-09 | CAMEROON | Lewis, Sonke |
| DJA-17 | CAMEROON | Lewis, Sonke |
| DJK-01 | CAMEROON | Alaman, Banin, Begne, Lewis, Peh, Sonke, Taedoumg, Talbot, Zemagho |
| DJK-02 | CAMEROON | Alaman, Banin, Begne, Lewis, Peh, Sonke, Taedoumg, Talbot, Zemagho |
| DJK-03 | CAMEROON | Alaman, Banin, Begne, Lewis, Peh, Sonke, Taedoumg, Talbot, Zemagho |
| DJK-04 | CAMEROON | Alaman, Banin, Begne, Lewis, Peh, Sonke, Taedoumg, Talbot, Zemagho |
| DJK-05 | CAMEROON | Alaman, Banin, Begne, Lewis, Peh, Sonke, Taedoumg, Talbot, Zemagho |
| DJK-06 | CAMEROON | Alaman, Banin, Begne, Lewis, Peh, Sonke, Taedoumg, Talbot, Zemagho |
| DJL-01 | CAMEROON | Begne, Kamdem, Lewis, Sonke |
| DJL-02 | CAMEROON | Begne, Kamdem, Lewis, Sonke |
| DJL-03 | CAMEROON | Begne, Kamdem, Lewis, Sonke |
| DJL-04 | CAMEROON | Begne, Kamdem, Lewis, Sonke |
| DJL-05 | CAMEROON | Begne, Kamdem, Lewis, Sonke |
| DJL-06 | CAMEROON | Begne, Kamdem, Lewis, Sonke |
| DNG-01 | CAMEROON | Droissant, Sonke |
| DNG-02 | CAMEROON | Droissant, Sonke |
| **Plot** | **Country** | **Leadership team** |
| DNG-03 | CAMEROON | Sonke |
| DNG-04 | CAMEROON | Sonke |
| DNG-05 | CAMEROON | Sonke |
| DOI-01 | BRAZIL | Baker, Castro, Feldpausch, Lloyd, Phillips, Silveira |
| DOI-02 | BRAZIL | Baker, Castro, Feldpausch, Lloyd, Phillips, Silveira |
| DOU-01 | GABON | Reitsma |
| DRA-04 | GHANA | Affum-Baffoe, Baker, Fauset, Feldpausch, Lewis |
| DRA-05 | GHANA | Affum-Baffoe, Baker, Fauset, Feldpausch, Lewis |
| DZS-01 | CENTRAL AFRICAN REP | Sunderland |
| DZS-02 | CENTRAL AFRICAN REP | Sunderland |
| DZS-03 | CENTRAL AFRICAN REP | Sunderland |
| DZS-04 | CENTRAL AFRICAN REP | Sunderland |
| DZS-05 | CENTRAL AFRICAN REP | Sunderland |
| EJA-04 | CAMEROON | Comiskey, Lewis, Sonke, Sunderland, Talbot, Zemagho |
| EJA-05 | CAMEROON | Comiskey, Lewis, Sonke, Sunderland, Talbot, Zemagho |
| EKO-01 | GABON | Cuni Sanchez, Lewis, Reitsma |
| ELD-01 | VENEZUELA | D'Jesus, Phillips, Ramirez-Angulo, Serrano, Torres-Lezama, van der Heijden, Veillon, Vilanova Torre |
| ELD-02 | VENEZUELA | D'Jesus, Phillips, Ramirez-Angulo, Serrano, Torres-Lezama, van der Heijden, Veillon, Vilanova Torre |
| ELD-03 | VENEZUELA | D'Jesus, Phillips, Ramirez-Angulo, Serrano, Torres-Lezama, van der Heijden, Veillon, Vilanova Torre |
| ELD-04 | VENEZUELA | D'Jesus, Phillips, Ramirez-Angulo, Serrano, Torres-Lezama, van der Heijden, Veillon, Vilanova Torre |
| ESU-18 | GHANA | Affum-Baffoe, Baker, Fauset, Feldpausch, Lewis |
| FEC-01 | BRAZIL | Aparecida Vieira, Barbosa Camargo, Brown, Castro, Feldpausch, Phillips, Silveira |
| FLO-02 | BRAZIL | Feldpausch, Marimon, Marimon Junior, Phillips |
| FMH-01 | GUYANA | Arets, Banki, Brienen, Feldpausch, Phillips, Singh, ter Steege, van Ulft, Zagt |
| FMH-02 | GUYANA | Brienen, Feldpausch, Phillips, Singh, ter Steege, Zagt |
| FMH-03 | GUYANA | Banki, Brienen, Feldpausch, Phillips, Singh, ter Steege |
| FUR-07 | GHANA | Affum-Baffoe, Baker, Fauset, Feldpausch, Lewis |
| FUR-08 | GHANA | Affum-Baffoe, Baker, Fauset, Feldpausch, Lewis |
| GBO-01 | LIBERIA | Daniels, Hubau, Lewis, Woell, Woods |
| GBO-02 | LIBERIA | Daniels, Hubau, Lewis, Phillips, Tuagben , Woell, Woods |
| GBO-03 | LIBERIA | Daniels, Hubau, Lewis, Tuagben , Woell, Woods |
| **Plot** | **Country** | **Leadership team** |
| GBO-04 | LIBERIA | Daniels, Hubau, Lewis, Phillips, Tuagben , Woell, Woods |
| GBO-05 | LIBERIA | Daniels, Hubau, Lewis, Tuagben , Woell, Woods |
| GBO-06 | LIBERIA | Daniels, Hubau, Lewis, Tuagben , Woell, Woods |
| GBO-07 | LIBERIA | Daniels, Hubau, Lewis, Tuagben , Woell, Woods |
| GBO-08 | LIBERIA | Daniels, Hubau, Lewis, Phillips, Tuagben , Woell, Woods |
| GBO-09 | LIBERIA | Daniels, Hubau, Lewis, Tuagben , Woell, Woods |
| GBO-10 | LIBERIA | Daniels, Hubau, Lewis, Tuagben , Woell, Woods |
| GBO-11 | LIBERIA | Daniels, Hubau, Lewis, Phillips, Tuagben , Woell, Woods |
| GBO-12 | LIBERIA | Daniels, Hubau, Lewis, Tuagben , Woell, Woods |
| GBO-13 | LIBERIA | Daniels, Hubau, Lewis, Tuagben , Woell, Woods |
| GBO-14 | LIBERIA | Daniels, Hubau, Lewis, Tuagben , Woell, Woods |
| GBO-15 | LIBERIA | Daniels, Hubau, Lewis, Phillips, Tuagben , Woell, Woods |
| GBO-16 | LIBERIA | Daniels, Hubau, Lewis, Tuagben , Woell, Woods |
| GBO-17 | LIBERIA | Daniels, Hubau, Lewis, Tuagben , Woell, Woods |
| GBO-18 | LIBERIA | Daniels, Hubau, Lewis, Tuagben , Woell, Woods |
| GBO-19 | LIBERIA | Daniels, Hubau, Lewis, Phillips, Tuagben , Woell, Woods |
| GBO-20 | LIBERIA | Daniels, Hubau, Lewis, Tuagben , Woell, Woods |
| GJB-01 | INDONESIA | Phillips |
| GLS-01 | INDONESIA | Samsoedin |
| GMU-01 | MALAYSIA | Banin, Lewis, Phillips, Proctor, Qie, Tan |
| GMU-02 | MALAYSIA | Banin, Lewis, Phillips, Proctor, Qie, Tan |
| GMU-03 | MALAYSIA | Banin, Lewis, Phillips, Proctor, Qie, Tan |
| GMU-04 | MALAYSIA | Lewis, Phillips, Proctor, Qie, Tan |
| GOL-02 | SIERRA LEONE | Coomes, Cuni Sanchez, Fofanah , Jucker, Lindsell |
| GOL-03 | SIERRA LEONE | Coomes, Cuni Sanchez, Fofanah , Jucker, Lindsell |
| GOL-05 | SIERRA LEONE | Coomes, Cuni Sanchez, Fofanah , Jucker, Lindsell |
| GOL-07 | SIERRA LEONE | Coomes, Cuni Sanchez, Fofanah , Jucker, Lindsell |
| GOL-08 | SIERRA LEONE | Coomes, Cuni Sanchez, Fofanah , Jucker, Lindsell |
| GOL-10 | SIERRA LEONE | Coomes, Cuni Sanchez, Fofanah , Jucker, Lindsell |
| GOL-11 | SIERRA LEONE | Coomes, Cuni Sanchez, Fofanah , Jucker, Lindsell |
| GOL-12 | SIERRA LEONE | Coomes, Cuni Sanchez, Fofanah , Jucker, Lindsell |
| GOL-14 | SIERRA LEONE | Coomes, Cuni Sanchez, Fofanah , Jucker, Lindsell |
| GOL-17 | SIERRA LEONE | Coomes, Cuni Sanchez, Fofanah , Jucker, Lindsell |
| GOL-18 | SIERRA LEONE | Coomes, Cuni Sanchez, Fofanah , Jucker, Lindsell |
| GOL-19 | SIERRA LEONE | Coomes, Cuni Sanchez, Fofanah , Jucker, Lindsell |
| GOL-25 | SIERRA LEONE | Coomes, Cuni Sanchez, Fofanah , Jucker, Lindsell |
| GOL-26 | SIERRA LEONE | Coomes, Cuni Sanchez, Fofanah , Jucker, Lindsell |
| GOL-27 | SIERRA LEONE | Coomes, Cuni Sanchez, Fofanah , Jucker, Lindsell |
| GOL-31 | SIERRA LEONE | Coomes, Cuni Sanchez, Fofanah , Jucker, Lindsell |
| GOL-32 | SIERRA LEONE | Coomes, Cuni Sanchez, Fofanah , Jucker, Lindsell |
| **Plot** | **Country** | **Leadership team** |
| GOL-33 | SIERRA LEONE | Coomes, Cuni Sanchez, Fofanah , Jucker, Lindsell |
| GOL-34 | SIERRA LEONE | Coomes, Cuni Sanchez, Fofanah , Jucker, Lindsell |
| GOL-37 | SIERRA LEONE | Coomes, Cuni Sanchez, Fofanah , Jucker, Lindsell |
| GOL-39 | SIERRA LEONE | Coomes, Cuni Sanchez, Fofanah , Jucker, Lindsell |
| GOL-40 | SIERRA LEONE | Coomes, Cuni Sanchez, Fofanah , Jucker, Lindsell |
| GOL-41 | SIERRA LEONE | Coomes, Cuni Sanchez, Fofanah , Jucker, Lindsell |
| GOL-43 | SIERRA LEONE | Coomes, Cuni Sanchez, Fofanah , Jucker, Lindsell |
| GOL-44 | SIERRA LEONE | Coomes, Cuni Sanchez, Fofanah , Jucker, Lindsell |
| GOL-46 | SIERRA LEONE | Coomes, Cuni Sanchez, Fofanah , Jucker, Lindsell |
| GOL-47 | SIERRA LEONE | Coomes, Cuni Sanchez, Fofanah , Jucker, Lindsell |
| GOL-49 | SIERRA LEONE | Coomes, Cuni Sanchez, Fofanah , Jucker, Lindsell |
| GOL-50 | SIERRA LEONE | Coomes, Cuni Sanchez, Fofanah , Jucker, Lindsell |
| GOL-51 | SIERRA LEONE | Coomes, Cuni Sanchez, Fofanah , Jucker, Lindsell |
| GOL-52 | SIERRA LEONE | Coomes, Cuni Sanchez, Fofanah , Jucker, Lindsell |
| GOL-53 | SIERRA LEONE | Coomes, Cuni Sanchez, Fofanah , Jucker, Lindsell |
| GOL-54 | SIERRA LEONE | Coomes, Cuni Sanchez, Fofanah , Jucker, Lindsell |
| GOL-55 | SIERRA LEONE | Coomes, Cuni Sanchez, Fofanah , Jucker, Lindsell |
| GOL-56 | SIERRA LEONE | Coomes, Cuni Sanchez, Fofanah , Jucker, Lindsell |
| GOL-62 | SIERRA LEONE | Coomes, Cuni Sanchez, Fofanah , Jucker, Lindsell |
| GOL-63 | SIERRA LEONE | Coomes, Cuni Sanchez, Fofanah , Jucker, Lindsell |
| GOL-77 | SIERRA LEONE | Coomes, Cuni Sanchez, Fofanah , Jucker, Lindsell |
| GOL-88 | SIERRA LEONE | Coomes, Cuni Sanchez, Fofanah , Jucker, Lindsell |
| HAB-01 | GABON | Chezeaux |
| HAB-02 | GABON | Chezeaux |
| HAB-03 | GABON | Chezeaux, Cuni Sanchez, Lewis |
| HAB-04 | GABON | Chezeaux |
| HAB-05 | GABON | Chezeaux |
| HAB-06 | GABON | Chezeaux, Cuni Sanchez, Doucet, Lewis |
| HAB-07 | GABON | Chezeaux, Cuni Sanchez, Doucet, Lewis |
| INF-01 | PERU | Alexiades, Phillips |
| ITT-72 | INDONESIA | Yassir |
| ITU-01 | CONGO, DEMOCRATIC REPUBLIC | Hart |
| ITU-02 | CONGO, DEMOCRATIC REPUBLIC | Hart |
| ITU-03 | CONGO, DEMOCRATIC REPUBLIC | Hart |
| **Plot** | **Country** | **Leadership team** |
| ITU-04 | CONGO, DEMOCRATIC REPUBLIC | Hart |
| ITU-05 | CONGO, DEMOCRATIC REPUBLIC | Hart |
| ITU-06 | CONGO, DEMOCRATIC REPUBLIC | Hart |
| ITU-76 | INDONESIA | Idhamsyah, Lewis, Phillips, Qie, Yassir |
| ITV-76 | INDONESIA | Yassir |
| ITW-76 | INDONESIA | Yassir |
| ITX-71 | INDONESIA | Yassir |
| ITY-76 | INDONESIA | Yassir |
| ITZ-76 | INDONESIA | Yassir |
| IVI-01 | GABON | Bengone, Evouna Ondo, Jeffery, Malhi, Mihindou, Moore, White |
| IVI-02 | GABON | Bengone, Evouna Ondo, Jeffery, Malhi, Mihindou, Moore, White |
| JAC-01 | BRAZIL | Higuchi, Nogueira Lima |
| JAC-02 | BRAZIL | Higuchi, Nogueira Lima |
| JAM-01 | BRAZIL | Lima, Lisboa, Maciel |
| JAS-02 | ECUADOR | Baker, Brienen, Monteagudo-Mendoza, Neill, Phillips |
| JAS-03 | ECUADOR | Baker, Brienen, Lloyd, Monteagudo-Mendoza, Neill, Phillips |
| JEN-11 | PERU | Baker, Brienen, del Aguila Pasquel, Honorio Coronado, Monteagudo-Mendoza, Phillips |
| JEN-12 | PERU | Baker, Brienen, del Aguila Pasquel, Honorio Coronado, Monteagudo-Mendoza, Phillips |
| JEN-13 | PERU | Baker, Brienen, del Aguila Pasquel, Honorio Coronado, Monteagudo-Mendoza, Phillips |
| JRI-01 | BRAZIL | Silva |
| KDE-01 | GHANA | Swaine |
| KDE-02 | GHANA | Swaine |
| KIB-01 | UGANDA | Chapman |
| KIB-02 | UGANDA | Chapman |
| KIB-03 | UGANDA | Chapman |
| KIB-04 | UGANDA | Chapman |
| KIB-05 | UGANDA | Chapman |
| KIB-06 | UGANDA | Chapman |
| KIB-07 | UGANDA | Chapman |
| KIB-08 | UGANDA | Chapman |
| KIB-09 | UGANDA | Chapman |
| KIB-10 | UGANDA | Chapman |
| **Plot** | **Country** | **Leadership team** |
| KIB-11 | UGANDA | Chapman |
| KIF-05 | TANZANIA, | Williams |
| KIF-06 | TANZANIA, | Williams |
| KIF-07 | TANZANIA, | Williams |
| KIF-12 | TANZANIA, | Williams |
| KIF-13 | TANZANIA, | Williams |
| KIF-15 | TANZANIA, | Williams |
| KIQ-17 | MALAYSIA | Aiba, Kitayama |
| KIS-07 | MALAYSIA | Aiba, Kitayama |
| KIS-17 | MALAYSIA | Aiba, Kitayama |
| KIS-27 | MALAYSIA | Aiba, Kitayama |
| KIS-31 | MALAYSIA | Aiba, Kitayama |
| KIU-07 | MALAYSIA | Aiba, Kitayama |
| KIU-17 | MALAYSIA | Aiba, Kitayama |
| KIU-27 | MALAYSIA | Aiba, Kitayama |
| KOG-01 | GHANA | Feldpausch, Lloyd |
| KOG-02 | GHANA | Affum-Baffoe, Foli, Malhi, Moore |
| KOG-03 | GHANA | Affum-Baffoe, Foli, Malhi, Moore |
| KOG-04 | GHANA | Affum-Baffoe, Foli, Malhi, Moore |
| KOG-05 | GHANA | Affum-Baffoe, Foli, Malhi, Moore |
| KOG-06 | GHANA | Affum-Baffoe, Foli, Malhi, Moore |
| KOL-01 | CONGO-BRAZZAVILLE | Averti Ifo, Begne, Dargie, Enock, Lewis |
| KOL-02 | CONGO-BRAZZAVILLE | Averti Ifo, Begne, Dargie, Enock, Lewis |
| KOL-03 | CONGO-BRAZZAVILLE | Averti Ifo, Begne, Dargie, Enock, Lewis |
| KOL-04 | CONGO-BRAZZAVILLE | Averti Ifo, Begne, Dargie, Enock, Lewis |
| KSN-01 | DRC | Amani , Hardy, Hubau, Lewis, Phillips, Tshibamba Mukendi, Vleminckx |
| KSN-02 | DRC | Amani , Hardy, Hubau, Lewis, Phillips, Tshibamba Mukendi, Vleminckx |
| KSN-03 | DRC | Amani , Hardy, Hubau, Vleminckx |
| KSN-04 | DRC | Amani , Hardy, Hubau, Vleminckx |
| KSN-05 | DRC | Amani , Hardy, Hubau, Lewis, Phillips, Tshibamba Mukendi, Vleminckx |
| KSN-06 | DRC | Amani , Hardy, Hubau, Lewis, Phillips, Tshibamba Mukendi, Vleminckx |
| KSN-07 | DRC | Amani , Hardy, Hubau, Vleminckx |
| KSN-08 | DRC | Amani , Hardy, Hubau, Vleminckx |
| **Plot** | **Country** | **Leadership team** |
| KSN-09 | DRC | Amani , Hardy, Hubau, Vleminckx |
| KSN-10 | DRC | Amani , Hardy, Hubau, Vleminckx |
| KSN-11 | DRC | Amani , Hardy, Hubau, Vleminckx |
| KSN-12 | DRC | Amani , Hardy, Hubau, Vleminckx |
| KSN-13 | DRC | Amani , Hardy, Hubau, Vleminckx |
| KSN-14 | DRC | Amani , Hardy, Hubau, Vleminckx |
| KSN-15 | DRC | Amani , Hardy, Hubau, Vleminckx |
| KUB-01 | INDONESIA | Ambriansyah, Lewis, Phillips, Qie, Rutishauser |
| LAD-01 | BRUNEI DARUSSALAM | Davies |
| LAM-02 | MALAYSIA | Ashton, Lewis, Phillips, Qie, Tan |
| LAM-03 | MALAYSIA | Ashton, Lewis, Phillips, Qie, Tan |
| LAM-04 | MALAYSIA | Ashton, Lewis, Phillips, Qie, Tan |
| LAM-05 | MALAYSIA | Ashton, Lewis, Phillips, Qie, Tan |
| LAS-02 | PERU | Chama Moscoso, Cornejo Valverde, Phillips, Pitman |
| LCO-01 | GABON | Collins, Cuni Sanchez, Jeffery, Lewis, White |
| LCO-02 | GABON | Collins, Cuni Sanchez, Lewis, White |
| LCO-03 | GABON | Collins, Lewis, White |
| LCO-04 | GABON | Collins, Cuni Sanchez, Lewis, White |
| LCO-05 | GABON | Collins, Cuni Sanchez, Lewis, White |
| LFA-01 | BRAZIL | Da Silva, Rosa |
| LFB-01 | BOLIVIA | Araujo-Murakami, Arroyo, Brienen, Feldpausch, Killeen, Lloyd, Phillips |
| LFB-02 | BOLIVIA | Araujo-Murakami, Arroyo, Brienen, Feldpausch, Killeen, Lloyd, Phillips |
| LKM-01 | GABON | Chezeaux, Cuni Sanchez, Lewis |
| LKM-02 | GABON | Chezeaux, Cuni Sanchez, Lewis |
| LKM-03 | GABON | Chezeaux, Cuni Sanchez, Lewis |
| LKM-04 | GABON | Chezeaux, Cuni Sanchez |
| LKM-05 | GABON | Chezeaux |
| LKM-06 | GABON | Chezeaux |
| LKM-07 | GABON | Chezeaux, Cuni Sanchez, Lewis |
| LMA-01 | GABON | Collins, Cuni Sanchez, Jeffery, Lewis, White |
| LMA-02 | GABON | Collins, Cuni Sanchez, Jeffery, Lewis, White |
| LMA-03 | GABON | Collins, Cuni Sanchez, Jeffery, Lewis, White |
| LMA-04 | GABON | Collins, Cuni Sanchez, Jeffery, Lewis, White |
| LMA-05 | GABON | Collins, Cuni Sanchez, Jeffery, Lewis, White |
| LME-01 | GABON | Collins, Cuni Sanchez, Jeffery, Lewis, White |
| LME-02 | GABON | Collins, Cuni Sanchez, Jeffery, Lewis, White |
| LME-03 | GABON | Collins, Cuni Sanchez, Jeffery, Lewis, White |
| **Plot** | **Country** | **Leadership team** |
| LME-04 | GABON | Collins, Cuni Sanchez, Jeffery, Lewis, White |
| LME-05 | GABON | Collins, Cuni Sanchez, Jeffery, Lewis, White |
| LMO-01 | GABON | Collins, Cuni Sanchez, Dimoto, Jeffery, Lewis, White |
| LMO-02 | GABON | Collins, Cuni Sanchez, Jeffery, Lewis, White |
| LMO-03 | GABON | Collins, Cuni Sanchez, Jeffery, Lewis, White |
| LMO-04 | GABON | Collins, Cuni Sanchez, Jeffery, Lewis, White |
| LMO-05 | GABON | Collins, Cuni Sanchez, Jeffery, Lewis, White |
| LMP-01 | GABON | Collins, Jeffery, Lewis, White |
| LOP-01 | GABON | Collins, Jeffery, Lewis, Reitsma, White |
| LOP-25 | GABON | Collins, Jeffery, Lewis, White |
| LOR-01 | COLOMBIA | Alvarez Davila, Lloyd, Patino, Phillips, Prieto, Rudas |
| LOR-02 | COLOMBIA | Alvarez Davila, Lloyd, Patino, Phillips, Prieto, Rudas |
| LOR-03 | COLOMBIA | Lloyd, Phillips, Prieto, Rudas |
| LOT-01 | GABON | Doucet |
| LOT-02 | GABON | Doucet |
| LPG-01 | GABON | Bengone, Evouna Ondo , Jeffery, Malhi, Mihindou, Moore, White |
| LPG-02 | GABON | Bengone, Evouna Ondo , Jeffery, Malhi, Mihindou, Moore, White |
| LSL-01 | BOLIVIA | Araujo-Murakami, Arroyo, Brienen, Killeen, Lloyd, Phillips |
| LSL-02 | BOLIVIA | Araujo-Murakami, Arroyo, Brienen, Killeen, Lloyd, Phillips |
| LSO-01 | GABON | Collins, Jeffery, Lewis, White |
| LSO-02 | GABON | Collins, Jeffery, Lewis, White |
| LTL-01 | CONGO-BRAZZAVILLE | Dargie, Lewis |
| LWW-01 | GABON | Collins, Jeffery, Lewis, White |
| LWW-02 | GABON | Collins, Lewis, White |
| MAK-10 | GABON | Cuni Sanchez, Hladik, Lewis |
| MAL-01 | DRC | Bastin, Bogaert, De Canniere |
| MAL-02 | DRC | Bastin, Bogaert, De Canniere |
| MAL-03 | DRC | Bastin, Bogaert, De Canniere |
| MAL-04 | DRC | Bastin, Bogaert, De Canniere |
| MAL-05 | DRC | Bastin, Bogaert, De Canniere |
| MAL-06 | DRC | Bastin, Bogaert, De Canniere |
| MAL-07 | DRC | Bastin, Bogaert, De Canniere |
| MAL-08 | DRC | Bastin, Bogaert, De Canniere |
| MAL-09 | DRC | Bastin, Bogaert, De Canniere |
| MAL-10 | DRC | Bastin, Bogaert, De Canniere |
| MAL-11 | DRC | Bastin, Bogaert, De Canniere |
| MAL-12 | DRC | Bastin, Bogaert, De Canniere |
| MAL-13 | DRC | Bastin, Bogaert, De Canniere |
| **Plot** | **Country** | **Leadership team** |
| MAL-14 | DRC | Bastin, Bogaert, De Canniere |
| MAL-15 | DRC | Bastin, Bogaert, De Canniere |
| MAL-16 | DRC | Bastin, Bogaert, De Canniere |
| MAL-17 | DRC | Bastin, Bogaert, De Canniere |
| MAU-01 | INDONESIA | Phillips, Qie, Samsoedin, Sheil |
| MAU-02 | INDONESIA | Phillips, Qie, Samsoedin, Sheil |
| MAU-03 | INDONESIA | Phillips, Qie, Samsoedin, Sheil |
| MAU-04 | INDONESIA | Phillips, Qie, Samsoedin, Sheil |
| MBM-01 | GABON | Doucet, Paget |
| MBM-02 | GABON | Doucet |
| MBM-03 | GABON | Doucet, Paget |
| MBM-04 | GABON | Doucet, Paget |
| MCP-01 | BRAZIL | Da Silva, Rosa |
| MDC-01 | GABON | Cuni Sanchez, Leal, Lewis, Sunderland, White |
| MDC-02 | GABON | Cuni Sanchez, Leal, Lewis, Sunderland, White |
| MDC-03 | GABON | Cuni Sanchez, Leal, Lewis, Sunderland, White |
| MDC-04 | GABON | Cuni Sanchez, Leal, Lewis, Sunderland, White |
| MDC-05 | GABON | Cuni Sanchez, Leal, Lewis, Sunderland, White |
| MDJ-01 | CAMEROON | Begne, Feldpausch, Lewis, Lloyd, Sonke |
| MDJ-03 | CAMEROON | Begne, Feldpausch, Lewis, Lloyd, Sonke |
| MDJ-05 | CAMEROON | Begne, Feldpausch, Lewis, Lloyd, Sonke |
| MDJ-07 | CAMEROON | Begne, Feldpausch, Lewis, Lloyd, Sonke |
| MDJ-10 | CAMEROON | Begne, Feldpausch, Lewis, Lloyd, Sonke |
| MER-02 | MALAYSIA | Ashton, Tan |
| MER-05 | MALAYSIA | Ashton, Tan |
| MER-10 | MALAYSIA | Ashton, Tan |
| MER-11 | MALAYSIA | Ashton, Tan |
| MER-14 | MALAYSIA | Ashton, Tan |
| MIN-01 | BRAZIL | Barroso, Castro, Feldpausch, Phillips, Silveira |
| MIT-01 | CAMEROON | Droissant, Sonke |
| MKN-01 | GABON | Doucet |
| MKN-02 | GABON | Doucet |
| MKN-03 | GABON | Doucet |
| MKN-04 | GABON | Doucet |
| MKN-05 | GABON | Doucet |
| MKN-06 | GABON | Doucet |
| MKN-07 | GABON | Doucet |
| MKN-08 | GABON | Doucet |
| MKN-09 | GABON | Doucet |
| MKN-10 | GABON | Doucet |
| **Plot** | **Country** | **Leadership team** |
| MKN-11 | GABON | Doucet |
| MKN-12 | GABON | Doucet |
| MKN-13 | GABON | Doucet |
| MKN-14 | GABON | Doucet |
| MKN-15 | GABON | Doucet |
| MKN-16 | GABON | Doucet |
| MKN-17 | GABON | Doucet |
| MKN-18 | GABON | Doucet |
| MKN-19 | GABON | Doucet |
| MKN-20 | GABON | Doucet |
| MKN-21 | GABON | Doucet |
| MKN-22 | GABON | Doucet |
| MKN-23 | GABON | Doucet |
| MKN-24 | GABON | Doucet |
| MKN-25 | GABON | Doucet |
| MLL-01 | GABON | Doucet, Jeanmart |
| MLL-02 | GABON | Doucet, Jeanmart |
| MMI-01 | EQUATORIAL GUINEA | Gonmadje, Sunderland |
| MMI-02 | EQUATORIAL GUINEA | Gonmadje, Sunderland |
| MMI-03 | EQUATORIAL GUINEA | Gonmadje, Sunderland |
| MND-01 | GABON | Cuni Sanchez, Leal, Lewis, White |
| MND-02 | GABON | Cuni Sanchez, Leal, Lewis, White |
| MNG-03 | GABON | Bengone, Evouna, Jeffery, Malhi, Mihindou, Moore, White |
| MNG-04 | GABON | Bengone, Evouna Ondo , Jeffery, Malhi, Mihindou, Moore, White |
| MNK-01 | GABON | Leal, White |
| MNK-02 | GABON | Leal, White |
| MNK-03 | GABON | Cuni Sanchez, Lewis |
| MNU-03 | PERU | Brienen, Cornejo Valverde, Kapeshi, Nunez Vargas, Phillips, Terborgh |
| MNU-04 | PERU | Cornejo Valverde, Monteagudo-Mendoza, Nunez Vargas, Phillips, Terborgh |
| MNU-05 | PERU | Brienen, Cornejo Valverde, Nunez Vargas, Phillips, Terborgh |
| MNU-06 | PERU | Brienen, Cornejo Valverde, Nunez Vargas, Phillips, Terborgh |
| MNU-08 | PERU | Brienen, Cornejo Valverde, Monteagudo-Mendoza, Phillips, Pitman, Terborgh |
| MPG-01 | UGANDA | Akite, Eilu, Hamilton, Lewis, Mucunguzi, Taylor |
| MRB-01 | BRAZIL | Salomao |
| MRB-02 | BRAZIL | Salomao |
| **Plot** | **Country** | **Leadership team** |
| MRB-03 | BRAZIL | Salomao |
| MSH-01 | PERU | Gentry, Phillips |
| MTH-01 | BRAZIL | Baker, Barroso, Castro, Feldpausch, Lloyd, Phillips, Silveira |
| MYB-01 | GABON | Doucet, van de Pol |
| MYB-02 | GABON | Doucet, van de Pol |
| MYB-03 | GABON | Doucet, van de Pol |
| NGI-01 | CAMEROON | Lewis, Sonke, Talbot, Zemagho |
| NGI-02 | CAMEROON | Lewis, Sonke, Talbot, Zemagho |
| NGI-03 | CAMEROON | Lewis, Sonke, Talbot, Zemagho |
| NGI-04 | CAMEROON | Begne, Gilpin, Hubau, Lewis, Phillips, Qie, Sonke |
| NGI-05 | CAMEROON | Begne, Gilpin, Hubau, Lewis, Phillips, Qie, Sonke |
| NGI-06 | CAMEROON | Begne, Gilpin, Hubau, Lewis, Phillips, Sonke |
| NGI-07 | CAMEROON | Begne, Gilpin, Hubau, Lewis, Phillips, Sonke |
| NGI-08 | CAMEROON | Begne, Gilpin, Hubau, Lewis, Phillips, Sonke |
| NGI-09 | CAMEROON | Begne, Gilpin, Hubau, Lewis, Phillips, Sonke |
| NGI-10 | CAMEROON | Begne, Gilpin, Hubau, Lewis, Phillips, Sonke |
| NGI-11 | CAMEROON | Begne, Gilpin, Hubau, Lewis, Phillips, Sonke |
| NGI-12 | CAMEROON | Begne, Gilpin, Hubau, Lewis, Phillips, Sonke |
| NGO-01 | CAMEROON | Droissant, Sonke |
| NGO-02 | CAMEROON | Droissant, Sonke |
| NGO-03 | CAMEROON | Droissant, Sonke |
| NGO-04 | CAMEROON | Droissant, Simo, Sonke, Zemagho |
| NGO-05 | CAMEROON | Droissant, Sonke |
| NGO-06 | CAMEROON | Droissant, Simo, Sonke, Zemagho |
| NNP-01 | CONGO-BRAZZAVILLE | Gonmadje, Sunderland |
| NNP-02 | CONGO-BRAZZAVILLE | Gonmadje, Sunderland |
| NNP-03 | CONGO-BRAZZAVILLE | Gonmadje, Sunderland |
| NNP-04 | CONGO-BRAZZAVILLE | Sunderland |
| NNP-05 | CONGO-BRAZZAVILLE | Gonmadje, Sunderland |
| NOU-01 | FRENCH GUIANA | Baraloto, Betian, Blanc, Charles-Dominique, Chave, Engel, Fauset, Feldpausch, Monteagudo-Mendoza, Olivier, Petronelli, Phillips, Rejou-Mechain |
| NOU-02 | FRENCH GUIANA | Baraloto, Betian, Blanc, Charles-Dominique, Chave, Engel, Fauset, Feldpausch, Monteagudo-Mendoza, Olivier, Petronelli, Phillips, Rejou-Mechain |
| **Plot** | **Country** | **Leadership team** |
| NOU-03 | FRENCH GUIANA | Baraloto, Betian, Blanc, Charles-Dominique, Chave, Engel, Fauset, Feldpausch, Monteagudo-Mendoza, Olivier, Petronelli, Phillips, Rejou-Mechain |
| NOU-04 | FRENCH GUIANA | Baraloto, Betian, Blanc, Charles-Dominique, Chave, Engel, Fauset, Feldpausch, Monteagudo-Mendoza, Olivier, Petronelli, Phillips, Rejou-Mechain |
| NOU-05 | FRENCH GUIANA | Baraloto, Betian, Blanc, Charles-Dominique, Chave, Engel, Fauset, Feldpausch, Monteagudo-Mendoza, Olivier, Petronelli, Phillips, Rejou-Mechain |
| NOU-06 | FRENCH GUIANA | Baraloto, Betian, Blanc, Charles-Dominique, Chave, Engel, Fauset, Feldpausch, Monteagudo-Mendoza, Olivier, Petronelli, Phillips, Rejou-Mechain |
| NOU-07 | FRENCH GUIANA | Baraloto, Betian, Blanc, Charles-Dominique, Chave, Engel, Fauset, Feldpausch, Monteagudo-Mendoza, Olivier, Petronelli, Phillips, Rejou-Mechain |
| NOU-08 | FRENCH GUIANA | Baraloto, Betian, Blanc, Charles-Dominique, Chave, Engel, Fauset, Feldpausch, Monteagudo-Mendoza, Olivier, Petronelli, Phillips, Rejou-Mechain |
| NOU-09 | FRENCH GUIANA | Baraloto, Betian, Blanc, Charles-Dominique, Chave, Engel, Fauset, Feldpausch, Monteagudo-Mendoza, Olivier, Petronelli, Phillips, Rejou-Mechain |
| NOU-10 | FRENCH GUIANA | Baraloto, Betian, Blanc, Charles-Dominique, Chave, Engel, Fauset, Feldpausch, Monteagudo-Mendoza, Olivier, Petronelli, Phillips, Rejou-Mechain |
| NOU-11 | FRENCH GUIANA | Baisie, Baraloto, Betian, Bezard, Blanc, Bongers, Chave, Engel, Fauset, Feldpausch, Monteagudo-Mendoza, Naisso, Petronelli, Phillips, Rejou-Mechain, van de Meer |
| NOU-12 | FRENCH GUIANA | Baisie, Baraloto, Betian, Bezard, Blanc, Bongers, Chave, Engel, Fauset, Feldpausch, Monteagudo-Mendoza, Naisso, Petronelli, Phillips, Rejou-Mechain, van de Meer |
| NOU-13 | FRENCH GUIANA | Baisie, Baraloto, Betian, Bezard, Blanc, Bongers, Chave, Engel, Fauset, Feldpausch, Monteagudo-Mendoza, Naisso, Petronelli, Phillips, Rejou-Mechain, van de Meer |
| NOU-14 | FRENCH GUIANA | Baisie, Baraloto, Betian, Bezard, Blanc, Bongers, Chave, Engel, Fauset, Feldpausch, Monteagudo-Mendoza, Naisso, Petronelli, Phillips, Rejou-Mechain, van de Meer |
| NOU-15 | FRENCH GUIANA | Baisie, Baraloto, Betian, Bezard, Blanc, Bongers, Chave, Engel, Fauset, Feldpausch, Monteagudo-Mendoza, Naisso, Petronelli, Phillips, Rejou-Mechain, van de Meer |
| **Plot** | **Country** | **Leadership team** |
| NOU-16 | FRENCH GUIANA | Baisie, Baraloto, Betian, Bezard, Blanc, Bongers, Chave, Engel, Fauset, Feldpausch, Monteagudo-Mendoza, Naisso, Petronelli, Phillips, Rejou-Mechain, van de Meer |
| NOU-17 | FRENCH GUIANA | Baisie, Baraloto, Betian, Bezard, Blanc, Bongers, Chave, Engel, Fauset, Feldpausch, Monteagudo-Mendoza, Naisso, Petronelli, Phillips, Rejou-Mechain, van de Meer |
| NOU-18 | FRENCH GUIANA | Baisie, Baraloto, Betian, Bezard, Blanc, Bongers, Chave, Engel, Fauset, Feldpausch, Monteagudo-Mendoza, Naisso, Petronelli, Phillips, Rejou-Mechain, van de Meer |
| NOU-19 | FRENCH GUIANA | Baisie, Baraloto, Betian, Bezard, Blanc, Bongers, Chave, Engel, Fauset, Feldpausch, Monteagudo-Mendoza, Naisso, Petronelli, Phillips, Rejou-Mechain, van de Meer |
| NOU-20 | FRENCH GUIANA | Baisie, Baraloto, Betian, Bezard, Blanc, Bongers, Chave, Engel, Fauset, Feldpausch, Monteagudo-Mendoza, Naisso, Petronelli, Phillips, Rejou-Mechain, van de Meer |
| NOU-21 | FRENCH GUIANA | Baisie, Baraloto, Betian, Bezard, Blanc, Bongers, Chave, Engel, Fauset, Feldpausch, Monteagudo-Mendoza, Naisso, Petronelli, Phillips, Rejou-Mechain, van de Meer |
| NOU-22 | FRENCH GUIANA | Baisie, Baraloto, Betian, Bezard, Blanc, Bongers, Chave, Engel, Fauset, Feldpausch, Monteagudo-Mendoza, Naisso, Petronelli, Phillips, Rejou-Mechain, van de Meer |
| OBE-10 | NIGERIA | Lowe, Ojo |
| OBE-81 | NIGERIA | Lowe, Ojo |
| OBE-82 | NIGERIA | Lowe, Ojo |
| OBE-83 | NIGERIA | Lewis, Lowe, Ojo, Phillips |
| OBE-84 | NIGERIA | Lewis, Lowe, Ojo, Phillips |
| OBE-85 | NIGERIA | Lowe, Ojo |
| OBE-86 | NIGERIA | Lowe, Ojo |
| OBE-87 | NIGERIA | Lowe, Ojo |
| OBE-88 | NIGERIA | Lowe, Ojo |
| OBE-89 | NIGERIA | Lowe, Ojo |
| OBE-90 | NIGERIA | Lowe, Ojo |
| OBW-00 | NIGERIA | Lowe, Ojo |
| OBW-10 | NIGERIA | Lowe, Ojo |
| OBW-91 | NIGERIA | Lowe, Ojo |
| OBW-92 | NIGERIA | Lowe, Ojo |
| OBW-93 | NIGERIA | Lowe, Ojo |
| OBW-95 | NIGERIA | Lowe, Ojo |
| OBW-96 | NIGERIA | Lowe, Ojo |
| OBW-98 | NIGERIA | Lowe, Ojo |
| OBW-99 | NIGERIA | Lowe, Ojo |
| **Plot** | **Country** | **Leadership team** |
| ODE-01 | BRAZIL | Campbell, Daly, Maciel, Prance |
| ODE-02 | BRAZIL | Campbell, Daly, Maciel, Prance |
| OGI-01 | GABON | Chezeaux, Cuni Sanchez, Lewis |
| OGI-02 | GABON | Chezeaux |
| OGI-03 | GABON | Chezeaux |
| OGI-04 | GABON | Chezeaux |
| OGI-05 | GABON | Chezeaux |
| OGI-06 | GABON | Chezeaux |
| OGI-07 | GABON | Chezeaux, Cuni Sanchez, Lewis |
| OUT-01 | SIERRA LEONE | Balinga, Lewis, Sunderland |
| OUT-04 | SIERRA LEONE | Balinga, Lewis, Sunderland |
| OUT-05 | SIERRA LEONE | Balinga, Lewis, Sunderland |
| OVG-01 | GABON | Cuni Sanchez, Lewis, Reitsma |
| OWN-10 | NIGERIA | Lowe, Ojo |
| OWN-61 | NIGERIA | Lowe, Ojo |
| OWN-62 | NIGERIA | Lowe, Ojo |
| OWN-63 | NIGERIA | Lowe, Ojo |
| OWN-64 | NIGERIA | Lowe, Ojo |
| OWN-65 | NIGERIA | Lowe, Ojo |
| OWN-67 | NIGERIA | Lowe, Ojo |
| OWN-68 | NIGERIA | Lowe, Ojo |
| OWN-69 | NIGERIA | Lowe, Ojo |
| OWN-70 | NIGERIA | Lowe, Ojo |
| PAR-20 | FR. GUIANA | Bonal, Derroire, Stahl |
| PAR-21 | FR. GUIANA | Bonal, Burban, Derroire, Stahl |
| PAR-22 | FR. GUIANA | Bonal, Derroire, Stahl |
| PAR-23 | FR. GUIANA | Bonal, Derroire, Stahl |
| PAR-24 | FR. GUIANA | Bonal, Derroire, Stahl |
| PAR-25 | FR. GUIANA | Bonal, Derroire, Stahl |
| PAR-26 | FR. GUIANA | Bonal, Derroire, Stahl |
| PAR-27 | FR. GUIANA | Bonal, Derroire, Stahl |
| PAR-28 | FR. GUIANA | Bonal, Derroire, Stahl |
| PAR-29 | FR. GUIANA | Bonal, Derroire, Stahl |
| Pasoh | Malaysia | CTFS-ForestGeo |
| PAY-01 | ECUADOR | Ceron, Neill, Palacios, Pitman |
| PEA-02 | BRAZIL | Marimon, Marimon Junior |
| PIB-06 | GUYANA | Arets, Banki, Brienen, Phillips, Roopsind, Singh, ter Steege, van der Hout , van Ulft |
| PIB-12 | GUYANA | Arets, Banki, Brienen, Feldpausch, Phillips, Roopsind, Singh, ter Steege, van der Hout , van Ulft |
| **Plot** | **Country** | **Leadership team** |
| PIN-01 | INDONESIA | Phillips |
| PNY-04 | PERU | Monteagudo-Mendoza, Phillips, Vasquez Martinez |
| PNY-05 | PERU | Huamantupa-Chuquimaco, Monteagudo-Mendoza, Phillips, Vasquez Martinez |
| PNY-06 | PERU | Monteagudo, Pallqui Camacho, Phillips, Vasquez Martinez |
| PNY-07 | PERU | Monteagudo-Mendoza, Phillips, Vasquez Martinez |
| POR-01 | BRAZIL | Baker, Castro, Feldpausch, Lloyd, Phillips, Quesada, Silveira, Stropp |
| POR-02 | BRAZIL | Baker, Castro, Feldpausch, Lloyd, Phillips, Silveira |
| PPB-01 | BRAZIL | Salomao |
| PPB-02 | BRAZIL | Salomao |
| PPB-03 | BRAZIL | Salomao |
| PTB-01 | BRAZIL | Salomao |
| PTB-02 | BRAZIL | Salomao |
| RBM-01 | GABON | Doucet, van de Pol |
| RBM-02 | GABON | Doucet, van de Pol |
| RBM-03 | GABON | Doucet, van de Pol |
| RBM-04 | GABON | Doucet, van de Pol |
| RBR-01 | BRAZIL | Costa, Emilio, Magnusson, Schietti |
| RIA-01 | BRAZIL | Balee |
| RIO-01 | VENEZUELA | D'Jesus, Phillips, Ramirez-Angulo, Serrano, Torres-Lezama, van der Heijden, Veillon, Vilanova Torre |
| RIO-02 | VENEZUELA | Chama , D'Jesus, Phillips, Ramirez-Angulo, Serrano, Torres-Lezama, van der Heijden, Veillon, Vilanova Torre |
| RPI-01 | PERU | Nunez Vargas, Terborgh |
| RST-01 | BRAZIL | Baker, Barroso, Castro, Feldpausch, Phillips, Silveira |
| RTH-01 | PERU | Nunez Vargas, Terborgh |
| SAA-01 | BRAZIL | Marimon, Marimon Junior, Phillips |
| SCA-01 | GABON | Cuni Sanchez, Leal, Lewis, White |
| SCA-02 | GABON | Cuni Sanchez, Leal, Lewis, White |
| SCR-04 | VENEZUELA | Aymard C., Herrera Fernandez, Lloyd, Phillips |
| SCR-05 | VENEZUELA | Baker, Herrera Fernandez, Lloyd, Patino, Phillips |
| SCT-01 | BOLIVIA | Arroyo, Brienen, Mendoza, Parada Gutierrez, Phillips |
| SCT-06 | BOLIVIA | Arroyo, Brienen, Mendoza, Parada Gutierrez, Phillips |
| SGW-01 | INDONESIA | Lewis, Phillips, Qie, van Nieuwstadt |
| SGW-03 | INDONESIA | Lewis, Phillips, Qie, van Nieuwstadt |
| SGW-07 | INDONESIA | Lewis, Phillips, Qie, van Nieuwstadt |
| SGW-09 | INDONESIA | Lewis, Phillips, Qie, van Nieuwstadt |
| SGW-11 | INDONESIA | Lewis, Phillips, Qie, van Nieuwstadt |
| SGW-13 | INDONESIA | Lewis, Phillips, Qie, van Nieuwstadt |
| SGW-15 | INDONESIA | Lewis, Phillips, Qie, van Nieuwstadt |
| **Plot** | **Country** | **Leadership team** |
| SGW-17 | INDONESIA | Lewis, Phillips, Qie, van Nieuwstadt |
| SGW-21 | INDONESIA | Lhota, Fitriadi, Fredriksson, Lewis, Phillips |
| SGW-22 | INDONESIA | Lhota, Fitriadi, Fredriksson, Lewis, Phillips |
| SGW-23 | INDONESIA | Lhota, Fitriadi, Fredriksson, Lewis, Phillips |
| SGW-24 | INDONESIA | Lhota, Fitriadi, Fredriksson, Lewis, Phillips |
| SGW-25 | INDONESIA | Lhota, Fitriadi, Fredriksson, Lewis, Phillips |
| SGW-26 | INDONESIA | Lhota, Fitriadi, Fredriksson, Lewis, Phillips |
| SGW-27 | INDONESIA | Lhota, Fitriadi, Fredriksson, Lewis, Phillips |
| SGW-28 | INDONESIA | Lhota, Fitriadi, Fredriksson, Lewis, Phillips |
| SGW-29 | INDONESIA | Lhota, Fitriadi, Fredriksson, Lewis, Phillips |
| SGW-30 | INDONESIA | Lhota, Fitriadi, Fredriksson, Lewis, Phillips |
| SHI-01 | ECUADOR | Ceron, Pitman |
| SNG-01 | DRC | Ewango, Hall , Hubau, Lewis, Makana, Phillips, Umunay |
| SNG-02 | DRC | Ewango, Hall , Hubau, Lewis, Makana, Phillips, Umunay |
| SNG-03 | DRC | Ewango, Hall , Hubau, Lewis, Makana, Phillips, Umunay |
| SNG-04 | DRC | Ewango, Hall , Hubau, Lewis, Makana, Phillips, Umunay |
| SNG-05 | DRC | Ewango, Hall , Hubau, Lewis, Makana, Phillips, Umunay |
| SNG-06 | DRC | Ewango, Hall , Hubau, Lewis, Makana, Phillips, Umunay |
| SNG-07 | DRC | Ewango, Hall , Hubau, Lewis, Makana, Phillips, Umunay |
| SNG-08 | DRC | Ewango, Hall , Hubau, Lewis, Makana, Phillips, Umunay |
| SNG-09 | DRC | Ewango, Hall , Hubau, Lewis, Makana, Phillips, Umunay |
| SNG-10 | DRC | Ewango, Hall, Ilambu, Liengola, Makana, Mukinzi, Umunay |
| SNG-11 | DRC | Ewango, Hall, Ilambu, Liengola, Makana, Mukinzi, Umunay |
| SNG-12 | DRC | Ewango, Hall, Ilambu, Liengola, Makana, Mukinzi, Umunay |
| SNG-13 | DRC | Ewango, Hall, Ilambu, Liengola, Makana, Mukinzi, Umunay |
| SNG-14 | DRC | Ewango, Hall, Ilambu, Liengola, Makana, Mukinzi, Umunay |
| SNG-15 | DRC | Ewango, Hall, Ilambu, Liengola, Makana, Mukinzi, Umunay |
| SNG-16 | DRC | Ewango, Hall, Ilambu, Liengola, Makana, Mukinzi, Umunay |
| SNG-17 | DRC | Ewango, Hall, Ilambu, Liengola, Makana, Mukinzi, Umunay |
| SUC-01 | PERU | Baker, Brienen, Davila Cardozo, del Aguila Pasquel, Honorio Coronado, Monteagudo-Mendoza, Phillips, Pipoly, Ramirez Arevalo, Vasquez Martinez |
| SUC-02 | PERU | Baker, Brienen, Davila Cardozo, del Aguila Pasquel, Honorio Coronado, Monteagudo-Mendoza, Phillips, Pipoly, Ramirez Arevalo, Vasquez Martinez |
| SUC-03 | PERU | Baker, Brienen, Flores, Monteagudo-Mendoza, Phillips, Ramirez Arevalo, Vasquez Martinez |
| SUC-04 | PERU | Baker, Brienen, Davila, del Aguila, Honorio Coronado, Monteagudo-Mendoza, Phillips, Vasquez Martinez |
| **Plot** | **Country** | **Leadership team** |
| SUC-05 | PERU | Baker, Brienen, Davila, del Aguila, Honorio Coronado, Monteagudo-Mendoza, Phillips, Vasquez Martinez |
| SWT-01 | BRUNEI DARUSSALAM | Davies, Lewis, Phillips, Qie, Salim, Serudia, |
| TAM-01 | PERU | Baker, Erwin, Feldpausch, Gentry, Monteagudo-Mendoza, Phillips, Talbot, Vasquez Martinez, Vicenti |
| TAM-02 | PERU | Armas, Baker, Erwin, Feldpausch, Gentry, Hartshorn, Monteagudo-Mendoza, Phillips, Talbot, Vasquez Martinez |
| TAM-03 | PERU | Baker, Erwin, Feldpausch, Gentry, Monteagudo-Mendoza, Nunez Vargas, Phillips, Talbot, Vasquez Martinez |
| TAM-04 | PERU | Baker, Erwin, Feldpausch, Gentry, Lopez-Gonzalez, Monteagudo-Mendoza, Phillips, Vasquez Martinez |
| TAM-05 | PERU | Baker, Gentry, Monteagudo-Mendoza, Phillips, Talbot, Vasquez Martinez, Vicenti |
| TAM-07 | PERU | Baker, Erwin, Feldpausch, Gentry, Monteagudo-Mendoza, Phillips, Vasquez Martinez |
| TAM-08 | PERU | Baker, Monteagudo-Mendoza, Phillips, Talbot, Vasquez Martinez |
| TAM-09 | PERU | Huaraca Huasco, Malhi, Monteagudo-Mendoza, Phillips, Silva Espejo |
| TAN-02 | BRAZIL | Marimon, Marimon Junior, Phillips |
| TAN-04 | BRAZIL | Feldpausch, Lloyd, Marimon, Marimon Junior, Phillips |
| TAP-50 | BRAZIL | Silva |
| TAP-51 | BRAZIL | Silva |
| TAP-52 | BRAZIL | Silva |
| TAP-53 | BRAZIL | Silva |
| TAP-54 | BRAZIL | Silva |
| TAP-55 | BRAZIL | Silva |
| TAP-56 | BRAZIL | Silva |
| TAP-57 | BRAZIL | Silva |
| TAP-58 | BRAZIL | Silva |
| TAP-59 | BRAZIL | Silva |
| TAP-60 | BRAZIL | Silva |
| TAP-61 | BRAZIL | Silva |
| TBE-05 | GHANA | Affum-Baffoe, Baker, Fauset, Feldpausch, Lewis |
| TIP-01 | ECUADOR | Brienen, Monteagudo-Mendoza, Phillips, Pitman |
| TIP-02 | ECUADOR | Baker, Brienen, Erwin, Lloyd, Monteagudo-Mendoza, Neill, Phillips |
| TIP-03 | ECUADOR | Baker, Brienen, Erwin, Lloyd, Monteagudo-Mendoza, Neill, Phillips |
| TMP-01 | PERU | Nunez Vargas, Terborgh |
| TNP-06 | CAMEROON | Begne, Lewis, Sonke, Sunderland |
| **Plot** | **Country** | **Leadership team** |
| TNP-07 | CAMEROON | Begne, Lewis, Sonke, Sunderland |
| TNP-08 | CAMEROON | Begne, Lewis, Sonke, Sunderland |
| TNP-09 | CAMEROON | Begne, Lewis, Sonke, Sunderland |
| TNP-10 | CAMEROON | Begne, Lewis, Sonke, Sunderland |
| TNP-11 | CAMEROON | Begne, Lewis, Sonke, Sunderland |
| TNP-12 | CAMEROON | Begne, Lewis, Sonke, Sunderland |
| TNP-13 | CAMEROON | Begne, Lewis, Sonke, Sunderland |
| TNP-14 | CAMEROON | Begne, Lewis, Sonke, Sunderland |
| TNP-15 | CAMEROON | Begne, Lewis, Sonke, Sunderland |
| TON-01 | GHANA | Affum-Baffoe, Baker, Fauset, Feldpausch, Lewis |
| TON-08 | GHANA | Affum-Baffoe, Baker, Fauset, Feldpausch, Lewis |
| UDJ-01 | TANZANIA, | Lovett, Taplin, Willcock |
| UDJ-02 | TANZANIA, | Lovett, Taplin |
| VTA-02 | TANZANIA, | Lewis, Lovett, Marshall, Munishi, Willcock |
| VTA-03 | TANZANIA, | Lewis, Lovett, Marshall, Munishi, Rovero, Willcock |
| VTA-04 | TANZANIA, | Lewis, Lovett, Marshall, Munishi, Rovero, Willcock |
| VTA-05 | TANZANIA, | Lewis, Lovett, Marshall, Munishi, Rovero, Willcock |
| VTA-06 | TANZANIA, | Lewis, Lovett, Marshall, Munishi |
| VTA-07 | TANZANIA, | Lewis, Lovett, Marshall, Munishi, Willcock |
| VTA-08 | TANZANIA, | Lewis, Lovett, Marshall, Munishi, Willcock |
| VTA-09 | TANZANIA, | Lewis, Lovett, Marshall, Munishi, Willcock |
| VTA-10 | TANZANIA | Lewis, Lovett, Marshall, Munishi, Willcock |
| VTA-11 | TANZANIA, | Lewis, Lovett, Marshall, Munishi |
| VTA-12 | TANZANIA, | Lewis, Lovett, Marshall, Munishi, Willcock |
| VTA-13 | TANZANIA, | Lewis, Lovett, Marshall, Munishi, Willcock |
| VTA-14 | TANZANIA, | Lewis, Lovett, Marshall, Munishi, Rovero, Willcock |
| VTA-15 | TANZANIA, | Lewis, Lovett, Marshall, Munishi, Rovero, Willcock |
| VTA-16 | TANZANIA, | Lewis, Lovett, Marshall, Munishi, Willcock |
| VTA-17 | TANZANIA, | Lewis, Lovett, Marshall, Munishi, Willcock |
| VTA-18 | TANZANIA, | Lewis, Lovett, Marshall, Munishi, Willcock |
| VTA-19 | TANZANIA, | Lewis, Lovett, Marshall, Munishi, Willcock |
| VTA-20 | TANZANIA, | Lewis, Lovett, Marshall, Munishi, Willcock |
| VTA-21 | TANZANIA, | Lewis, Munishi, Willcock |
| VTA-22 | TANZANIA, | Lewis, Munishi, Willcock |
| VTA-23 | TANZANIA, | Lewis, Munishi, Willcock |
| VTA-24 | TANZANIA, | Lewis, Munishi, Willcock |
| VTA-25 | TANZANIA, | Lewis, Munishi, Willcock |
| **Plot** | **Country** | **Leadership team** |
| VTA-26 | TANZANIA, | Lewis, Munishi, Willcock |
| VTA-27 | TANZANIA, | Lewis, Munishi, Willcock |
| VTA-28 | TANZANIA, | Lewis, Munishi, Willcock |
| VTA-29 | TANZANIA, | Lewis, Munishi, Willcock |
| VTA-30 | TANZANIA, | Lewis, Munishi, Willcock |
| VTA-31 | TANZANIA, | Lewis, Munishi, Willcock |
| VTA-32 | TANZANIA, | Lewis, Munishi, Willcock |
| VTA-33 | TANZANIA, | Lewis, Munishi, Willcock |
| VTA-34 | TANZANIA, | Lewis, Munishi, Willcock |
| VTA-35 | TANZANIA, | Lewis, Munishi, Willcock |
| VTA-36 | TANZANIA, | Lewis, Munishi, Willcock |
| VTA-37 | TANZANIA, | Lewis, Munishi, Willcock |
| VTA-38 | TANZANIA, | Lewis, Munishi, Willcock |
| VTA-39 | TANZANIA, | Lewis, Munishi, Willcock |
| VTA-40 | TANZANIA, | Lewis, Munishi, Willcock |
| VTA-41 | TANZANIA, | Lewis, Munishi, Willcock |
| VTA-42 | TANZANIA, | Lewis, Munishi, Willcock |
| WKA-06 | GABON | Gonmadje, Sunderland |
| WKA-07 | GABON | Gonmadje, Sunderland |
| WKA-08 | GABON | Gonmadje, Sunderland |
| WKA-09 | GABON | Gonmadje, Sunderland |
| WKA-10 | GABON | Gonmadje, Sunderland |
| YAN-01 | PERU | Baker, Brienen, del Aguila Pasquel, Gentry, Honorio Coronado, Monteagudo-Mendoza, Phillips, Vasquez Martinez |
| YAN-02 | PERU | Baker, Brienen, del Aguila Pasquel, Honorio Coronado, Monteagudo-Mendoza, Phillips, Vasquez Martinez |
| YGB-08 | DRC | Beeckman, Boeckx, Bogaert, de Haulleville, Hubau, Kearsley |
| YGB-09 | DRC | Beeckman, Boeckx, Bogaert, de Haulleville, Delvaux, Kearsley |
| YGB-10 | DRC | Beeckman, Boeckx, Bogaert, de Haulleville, Delvaux, Kearsley |
| YGB-11 | DRC | Beeckman, Boeckx, Bogaert, de Haulleville, Delvaux, Kearsley |
| YGB-12 | DRC | Beeckman, Boeckx, Bogaert, de Haulleville, Delvaux, Kearsley |
| YGB-13 | DRC | Beeckman, Boeckx, Bogaert, de Haulleville, Delvaux, Kearsley |
| YGB-14 | DRC | Beeckman, Boeckx, Bogaert, de Haulleville, Hubau, Kearsley |
| YGB-15 | DRC | Beeckman, Boeckx, Bogaert, de Haulleville, Hubau, Kearsley |
| YGB-16 | DRC | Beeckman, Boeckx, Bogaert, de Haulleville, Hubau, Kearsley |
| YGB-17 | DRC | Beeckman, Boeckx, Bogaert, de Haulleville, Hubau, Kearsley |
| YGB-18 | DRC | Beeckman, Boeckx, Bogaert, de Haulleville, Hubau, Kearsley |
| YGB-24 | DRC | Beeckman, Boeckx, Bogaert, de Haulleville, Hubau, Kearsley |
| **Plot** | **Country** | **Leadership team** |
| YGB-25 | DRC | Beeckman, Boeckx, Bogaert, de Haulleville, Hubau, Kearsley |
| YGB-26 | DRC | Beeckman, Boeckx, Bogaert, de Haulleville, Hubau, Kearsley |
| YGB-27 | DRC | Beeckman, Boeckx, Bogaert, de Haulleville, Hubau, Kearsley |
| YGB-28 | DRC | Beeckman, Boeckx, Bogaert, de Haulleville, Hubau, Kearsley |
| YOK-01 | DRC | Hubau, Mbayu |
| YOK-02 | DRC | Hubau, Mbayu |
| YOK-03 | DRC | Hubau, Mbayu |
| YOK-04 | DRC | Hubau, Mbayu |
| YOK-05 | DRC | Hubau, Mbayu |
| YOK-06 | DRC | Hubau, Lewis, Mbayu, Phillips, Tshibamba Mukendi |
| YOK-07 | DRC | Hubau, Lewis, Mbayu, Phillips, Tshibamba Mukendi |
| YOK-08 | DRC | Hubau, Lewis, Mbayu, Phillips, Tshibamba Mukendi |
| YOK-09 | DRC | Hubau, Lewis, Mbayu, Phillips, Tshibamba Mukendi |
| YOK-10 | DRC | Hubau, Lewis, Mbayu, Phillips, Tshibamba Mukendi |
| YOK-11 | DRC | Hubau, Mbayu |
| YOK-12 | DRC | Hubau, Mbayu |
| YOK-13 | DRC | Hubau, Mbayu |
| YOK-14 | DRC | Hubau, Mbayu |
| YOK-15 | DRC | Hubau, Mbayu |
| YOK-16 | DRC | Hubau, Lewis, Mbayu, Phillips, Tshibamba Mukendi |
| YOK-17 | DRC | Hubau, Lewis, Mbayu, Phillips, Tshibamba Mukendi |
| YOK-18 | DRC | Hubau, Lewis, Mbayu, Phillips, Tshibamba Mukendi |
| YOK-19 | DRC | Hubau, Lewis, Mbayu, Phillips, Tshibamba Mukendi |
| YOK-20 | DRC | Hubau, Lewis, Mbayu, Phillips, Tshibamba Mukendi |
| ZAR-02 | COLOMBIA | Alvarez Davila, Jimenez, Lloyd, Penuela-Mora , Phillips |
| ZAR-03 | COLOMBIA | Alvarez Davila, Jimenez, Lloyd, Penuela-Mora , Phillips |
| ZAR-04 | COLOMBIA | Alvarez Davila, Jimenez, Lloyd, Penuela-Mora , Phillips |

**Table S4**

The latitudinal and longitudinal limits applied to the genera in this study to restrict calculations of range size to native occurrences of species within each genus. Some genera have multiple sets of range limits that reflect the disjunct nature of their distributions.

|  | **Genus** | **Max long** | **Min long** | | **Max lat** | **Min lat** |
| --- | --- | --- | --- | --- | --- | --- |
| 1 | *Abarema* | -35 |  |  | | -20 |
| 2 | *Adelia* |  |  |  | |  |
| 3 | *Adinandra* |  | 65 |  | |  |
| 4 | *Afrostyrax* |  |  |  | |  |
| 5 | *Afzelia* |  |  | 20 | |  |
| 6 | *Aglaia* |  | 76.4 |  | |  |
| 7 | *Aidia* |  | 60 |  | |  |
| 8 | *Aidia* | 60 | -50 |  | |  |
| 9 | *Alangium* | 70 | -50 |  | |  |
| 10 | *Alangium* |  | 60 |  | |  |
| 11 | *Albizia* | -35 | -120 |  | |  |
| 12 | *Albizia* |  | -30 |  | |  |
| 13 | *Alchornea* |  |  | 35 | |  |
| 14 | *Alchornea* | 60 | -35 |  | |  |
| 15 | *Alchornea* | -20 |  |  | |  |
| 16 | *Allanblackia* |  |  |  | |  |
| 17 | *Allophylus* | -30 | -130 |  | |  |
| 18 | *Allophylus* | 70 | 40 |  | |  |
| 19 | *Allophylus* | 70 | -30 | 40 | |  |
| 20 | *Alphonsea* |  | 70 |  | |  |
| 21 | *Alseis* | -30 |  |  | |  |
| 22 | *Alseodaphne* | 50 |  |  | |  |
| 23 | *Alstonia* | 50 | -45 | 45 | |  |
| 24 | *Alstonia* |  | 60 |  | | -35 |
| 25 | *Alstonia* | -50 | -120 |  | |  |
| 26 | *Amaioua* | -30 |  |  | |  |
| 27 | *Amanoa* | -20 | -92 |  | |  |
| 28 | *Amanoa* |  | -20 |  | |  |
| 29 | *Ambelania* | -35 |  |  | |  |
| 30 | *Ampelocera* |  |  |  | |  |
| 31 | *Amphimas* |  |  |  | |  |
| 32 | *Amphirrhox* |  |  |  | |  |
| 33 | *Anacardium* | -35 | -110 |  | |  |
| 34 | *Anaxagorea* | -35 |  |  | |  |
| 35 | *Anaxagorea* |  | 82 |  | |  |
| 36 | *Andira* | 28 | -30 |  | |  |
| 37 | *Andira* | -40 | -110 |  | |  |
| 38 | *Angylocalyx* |  |  |  | |  |
| 39 | *Aniba* | -20 |  |  | |  |
| 40 | *Anisophyllea* | -40 |  |  | |  |
| 41 | *Anisophyllea* | 60 | -40 |  | |  |
| 42 | *Anisophyllea* | 50 |  |  | |  |
| 43 | *Anisoptera* |  |  |  | |  |
| 44 | *Annickia* |  |  |  | |  |
| 45 | *Annona* | 80 | -28 | 58 | |  |
| 46 | *Annona* | -30 | -110 | 38 | |  |
| 47 | *Anonidium* | 35 | -10 |  | |  |
| 48 | *Anopyxis* |  |  |  | |  |
| 49 | *Anthonotha* |  | -10 |  | |  |
| 50 | *Antiaris* | 100 | -50 |  | |  |
| 51 | *Antidesma* | 70 | -45 |  | |  |
| 52 | *Antidesma* | 178 | 70 |  | |  |
|  | **Genus** | **Max long** | **Min long** | | **Max lat** | **Min lat** |
| 53 | *Aoranthe* |  |  |  | |  |
| 54 | *Aparisthmium* | -35 | -85 |  | |  |
| 55 | *Apeiba* | -35 |  |  | | -30 |
| 56 | *Aporosa* |  |  |  | | -15 |
| 57 | *Archidendron* |  |  |  | |  |
| 58 | *Artocarpus* |  | 60 |  | | -30 |
| 59 | *Aspidosperma* | -30 |  |  | |  |
| 60 | *Astrocaryum* | -35 |  |  | |  |
| 61 | *Astronium* | -10 |  |  | | -35 |
| 62 | *Attalea* | -10 |  | 35 | |  |
| 63 | *Atuna* |  |  |  | |  |
| 64 | *Aucoumea* | 46 |  |  | |  |
| 65 | *Aulacocalyx* |  |  |  | |  |
| 66 | *Baccaurea* |  | 50 | 35 | |  |
| 67 | *Baphia* | 100 |  |  | |  |
| 68 | *Barringtonia* |  | 70 |  | | -40 |
| 69 | *Barringtonia* | 70 | -30 |  | |  |
| 70 | *Barteria* |  |  |  | |  |
| 71 | *Batocarpus* |  |  |  | |  |
| 72 | *Bauhinia* |  | -30 | 56 | |  |
| 73 | *Bauhinia* | -35 | -120 | 55 | |  |
| 74 | *Beilschmiedia* | -40 |  | 30 | |  |
| 75 | *Beilschmiedia* | 60 | -38 |  | |  |
| 76 | *Beilschmiedia* | 60 |  |  | |  |
| 77 | *Berlinia* |  |  |  | |  |
| 78 | *Bhesa* |  |  |  | |  |
| 79 | *Bixa* | -35 | -110 |  | |  |
| 80 | *Blighia* | 120 | -20 |  | |  |
| 81 | *Blumeodendron* |  |  |  | |  |
| 82 | *Bocageopsis* |  |  | 8 | | -24.5 |
| 83 | *Bocoa* |  |  |  | |  |
| 84 | *Brachystegia* |  |  |  | |  |
| 85 | *Bridelia* |  | 60 |  | |  |
| 86 | *Bridelia* | 60 |  |  | |  |
| 87 | *Brosimum* | -35 |  |  | | -35 |
| 88 | *Buchanania* |  | 60 |  | |  |
| 89 | *Buchenavia* | -35 |  |  | |  |
| 90 | *Burkea* |  |  |  | |  |
| 91 | *Bussea* |  | 20 |  | |  |
| 92 | *Bussea* | 10 |  | 10 | |  |
| 93 | *Byrsonima* | -20 |  |  | |  |
| 94 | *Calatola* |  |  |  | |  |
| 95 | *Calophyllum* | -30 | -120 |  | |  |
| 96 | *Calophyllum* | 70 | 40 |  | |  |
| 97 | *Calophyllum* |  | 60 |  | |  |
| 98 | *Calpocalyx* |  |  |  | |  |
| 99 | *Calyptranthes* | -35 |  |  | | -35.2 |
| 100 | *Canarium* | 178 | 60 |  | |  |
| 101 | *Canarium* | 60 | -35 |  | |  |
| 102 | *Canthium* | 170 | 60 |  | |  |
| 103 | *Canthium* | 60 | -50 |  | |  |
|  | **Genus** | **Max long** | **Min long** | | **Max lat** | **Min lat** |
| 104 | *Capirona* | -35 |  |  | |  |
| 105 | *Caraipa* | -25 |  |  | |  |
| 106 | *Carallia* |  |  |  | |  |
| 107 | *Carapa* | -30 |  |  | |  |
| 108 | *Carapa* |  | -30 |  | |  |
| 109 | *Caryocar* | -30 |  |  | |  |
| 110 | *Casearia* | 60 | -140 | 35 | | -35 |
| 111 | *Casearia* |  | 60 | 40 | | -30 |
| 112 | *Cassipourea* | 70 | -35 |  | |  |
| 113 | *Cassipourea* | -35 |  |  | |  |
| 114 | *Castanopsis* |  | 83 | 30 | | -20 |
| 115 | *Castilla* | -30 | -115 | 35 | |  |
| 116 | *Catostemma* |  |  |  | |  |
| 117 | *Cecropia* | -35 | -140 | 35 | | -35 |
| 118 | *Ceiba* | -35 | -120 | 35 | |  |
| 119 | *Celtis* | -35 | -120 |  | |  |
| 120 | *Celtis* | 170 | -30 |  | |  |
| 121 | *Centroplacus* |  |  |  | |  |
| 122 | *Cephalomappa* |  |  |  | |  |
| 123 | *Chaetocarpus* | -35 |  |  | |  |
| 124 | *Chaetocarpus* | 60 | -20 | 45 | |  |
| 125 | *Chaetocarpus* | 85 |  |  | |  |
| 126 | *Cheiloclinium* | -30 |  |  | | -25.2 |
| 127 | *Chimarrhis* | -30 |  |  | |  |
| 128 | *Chionanthus* | 70 |  |  | |  |
| 129 | *Chionanthus* | 70 | -30 | 35 | |  |
| 130 | *Chionanthus* | -35 | -115 |  | |  |
| 131 | *Chisocheton* |  |  |  | |  |
| 132 | *Chlorocardium* |  |  |  | |  |
| 133 | *Chrysochlamys* |  |  |  | |  |
| 134 | *Chrysophyllum* | -35 | -120 | 38 | |  |
| 135 | *Chrysophyllum* | 70 | -30 | 40 | |  |
| 136 | *Chrysophyllum* | 65 |  |  | |  |
| 137 | *Cinnamomum* | 65 |  |  | |  |
| 138 | *Clarisia* | -25 |  | 23 | |  |
| 139 | *Cleistanthus* | 60 |  | 40 | |  |
| 140 | *Cleistanthus* |  | 60 | 40 | |  |
| 141 | *Cleistopholis* |  |  |  | |  |
| 142 | *Coccoloba* | -35 | -120 | 35 | | -35.2 |
| 143 | *Coelocaryon* |  |  | 11 | |  |
| 144 | *Coffea* | 65 | -35 | 20 | |  |
| 145 | *Coffea* |  | 65 |  | |  |
| 146 | *Cola* | 60 | -35 |  | |  |
| 147 | *Colubrina* |  |  |  | |  |
| 148 | *Combretum* | -35 | -115 |  | |  |
| 149 | *Combretum* | 60 | -30 | 35 | |  |
| 150 | *Combretum* | 160 | 60 |  | |  |
| 151 | *Conceveiba* | -20 |  |  | |  |
| 152 | *Copaifera* |  |  |  | |  |
| 153 | *Cordia* |  | -30 |  | |  |
| 154 | *Cordia* | -30 | -120 |  | |  |
| 155 | *Corynanthe* |  |  |  | |  |
| 156 | *Corythophora* |  |  |  | |  |
| 157 | *Couepia* | -20 |  |  | |  |
| 158 | *Coula* |  |  |  | |  |
| 159 | *Couratari* | -30 |  |  | |  |
| 160 | *Cratoxylum* |  | 0 |  | |  |
| 161 | *Crossopteryx* |  |  |  | |  |
| 162 | *Croton* | -30 | -140 |  | |  |
| 163 | *Croton* |  | 60 |  | |  |
|  | **Genus** | **Max long** | **Min long** | | **Max lat** | **Min lat** |
| 164 | *Croton* | 60 | -30 |  | |  |
| 165 | *Crudia* |  | 80 |  | |  |
| 166 | *Crudia* | -35 |  |  | |  |
| 167 | *Crudia* | 50 | -35 |  | |  |
| 168 | *Cryptocarya* |  |  | 40 | |  |
| 169 | *Ctenolophon* | 80 |  |  | |  |
| 170 | *Ctenolophon* | 80 |  |  | |  |
| 171 | *Cupania* | -30 |  |  | | -35.2 |
| 172 | *Cyathocalyx* |  |  |  | |  |
| 173 | *Cylicodiscus* |  |  |  | |  |
| 174 | *Cynometra* | -35 | -120 |  | |  |
| 175 | *Cynometra* |  | 60 |  | |  |
| 176 | *Cynometra* | 60 | -35 |  | |  |
| 177 | *Dacryodes* |  | 50 | 15 | |  |
| 178 | *Dacryodes* | -35 |  |  | |  |
| 179 | *Dactyladenia* | 120 |  |  | |  |
| 180 | *Dalbergia* | 178 | 60 |  | |  |
| 181 | *Dalbergia* | -35 | -110 | 35 | | -35 |
| 182 | *Dalbergia* | 50 | -30 |  | |  |
| 183 | *Daniellia* |  |  |  | |  |
| 184 | *Dasylepis* |  |  |  | |  |
| 185 | *Dehaasia* | 152 |  |  | |  |
| 186 | *Dendrobangia* |  |  |  | |  |
| 187 | *Dendropanax* | -35 |  |  | |  |
| 188 | *Dendropanax* | 95 |  |  | |  |
| 189 | *Desbordesia* | 25 |  |  | |  |
| 190 | *Desplatsia* |  |  |  | |  |
| 191 | *Dialium* | -35 |  |  | |  |
| 192 | *Dialium* | 60 | -30 |  | |  |
| 193 | *Dialium* |  | 90 |  | |  |
| 194 | *Dichostemma* |  |  |  | |  |
| 195 | *Dicorynia* |  |  |  | |  |
| 196 | *Dicymbe* |  |  |  | |  |
| 197 | *Dicypellium* |  |  |  | |  |
| 198 | *Dillenia* |  | 80 |  | | -35 |
| 199 | *Diogoa* |  |  |  | |  |
| 200 | *Diospyros* | -35 | -140 | 70 | |  |
| 201 | *Diospyros* |  | -28 |  | |  |
| 202 | *Diplorhynchus* | -20 |  |  | |  |
| 203 | *Diplospora* |  |  |  | |  |
| 204 | *Diplotropis* | -20 |  |  | |  |
| 205 | *Dipterocarpus* |  |  |  | |  |
| 206 | *Dipteryx* | -20 |  | 40 | |  |
| 207 | *Discoglypremna* |  |  |  | |  |
| 208 | *Distemonanthus* |  |  |  | |  |
| 209 | *Dryobalanops* | 103 |  |  | |  |
| 210 | *Drypetes* |  | 60 |  | |  |
| 211 | *Drypetes* | -35 |  |  | |  |
| 212 | *Drypetes* | 60 | -30 |  | |  |
| 213 | *Duguetia* | -20 |  |  | |  |
| 214 | *Duguetia* |  | -20 |  | |  |
| 215 | *Durio* | 146 | 50 |  | |  |
| 216 | *Duroia* | -20 |  |  | |  |
| 217 | *Dyera* |  |  |  | |  |
| 218 | *Dysoxylum* | 176 | 50 |  | |  |
| 219 | *Ecclinusa* | 0 |  |  | | -25 |
| 220 | *Elaeocarpus* | 140 | 10 |  | | -20 |
| 221 | *Elaeocarpus* | 178 | 140 | 0 | | -20 |
| 222 | *Elateriospermum* | 121 |  |  | |  |
| 223 | *Endlicheria* | -20 |  | 15 | |  |
|  | **Genus** | **Max long** | **Min long** | | **Max lat** | **Min lat** |
| 224 | *Endopleura* |  | -70 | 21 | | -15 |
| 225 | *Englerophytum* |  |  |  | |  |
| 226 | *Enicosanthum* |  |  |  | |  |
| 227 | *Entandrophragma* |  | 23 | -25.2 | |  |
| 228 | *Eperua* | -20 |  |  | |  |
| 229 | *Eriocoelum* |  |  |  | |  |
| 230 | *Eriotheca* | -20 |  |  | |  |
| 231 | *Erisma* | -20 |  |  | |  |
| 232 | *Erythrina* |  | -35 | 38 | |  |
| 233 | *Erythrina* | -35 | -120 |  | |  |
| 234 | *Erythrophleum* | 120 |  |  | |  |
| 235 | *Erythrophleum* | 60 |  | 38 | |  |
| 236 | *Eschweilera* | -35 | -90 |  | |  |
| 237 | *Eugenia* |  | 83 |  | |  |
| 238 | *Eugenia* | 60 | -130 | 55 | |  |
| 239 | *Eusideroxylon* |  |  |  | |  |
| 240 | *Euterpe* | -2 |  | 21 | |  |
| 241 | *Ficus* |  | -30 |  | |  |
| 242 | *Ficus* | -35 | -130 | 35 | |  |
| 243 | *Funtumia* |  | -30 |  | |  |
| 244 | *Fusaea* |  |  |  | |  |
| 245 | *Garcinia* | 65 | -28 | 20 | |  |
| 246 | *Garcinia* |  | 65 |  | |  |
| 247 | *Garcinia* | -35 | -120 | 35 | |  |
| 248 | *Geissospermum* |  |  |  | |  |
| 249 | *Gilbertiodendron* | -30 |  |  | |  |
| 250 | *Gironniera* | 170 | 50 |  | |  |
| 251 | *Gluta* | 80 | 47.5 |  | |  |
| 252 | *Gluta* | 120 | 80 |  | |  |
| 253 | *Gonocaryum* | 50 | 39 |  | |  |
| 254 | *Gonystylus* | 170 | 50 |  | |  |
| 255 | *Goupia* |  |  |  | |  |
| 256 | *Greenwayodendron* |  |  |  | |  |
| 257 | *Grewia* | 170 | -35 |  | |  |
| 258 | *Grossera* | 40 |  |  | |  |
| 259 | *Grossera* |  | 40 |  | |  |
| 260 | *Guapira* |  |  |  | |  |
| 261 | *Guarea* | -35 |  | 40 | |  |
| 262 | *Guarea* | 60 | -30 |  | |  |
| 263 | *Guatteria* | -30 |  |  | | -35 |
| 264 | *Guazuma* | -30 |  |  | | -35.2 |
| 265 | *Guettarda* | -35 | -120 |  | | -35.2 |
| 266 | *Gustavia* | -20 |  |  | |  |
| 267 | *Gymnacranthera* |  |  |  | |  |
| 268 | *Hasseltia* |  |  |  | |  |
| 269 | *Heisteria* |  | -20 |  | |  |
| 270 | *Heisteria* | -20 |  |  | |  |
| 271 | *Helicostylis* | -25 |  |  | |  |
| 272 | *Heritiera* |  | 60 |  | |  |
| 273 | *Heritiera* | 60 | 30 |  | |  |
| 274 | *Hevea* | -20 | -150 |  | |  |
| 275 | *Hexalobus* |  |  |  | |  |
| 276 | *Hirtella* | -35 |  |  | |  |
| 277 | *Hirtella* |  | 35 |  | |  |
| 278 | *Homalium* | 70 | -30 | 38 | |  |
| 279 | *Homalium* |  | 70 |  | |  |
| 280 | *Homalium* | -35 | -120 |  | |  |
| 281 | *Hopea* |  | 83 |  | |  |
| 282 | *Horsfieldia* |  | 40 |  | |  |
|  | **Genus** | **Max long** | **Min long** | | **Max lat** | **Min lat** |
| 283 | *Hura* | -45 |  | 25 | |  |
| 284 | *Hydnocarpus* | 50 | 30 | -10 | |  |
| 285 | *Hylodendron* |  |  |  | |  |
| 286 | *Hymenaea* | -35 | -120 |  | |  |
| 287 | *Hymenaea* | 60 | -15 |  | |  |
| 288 | *Hymenocardia* | 60 |  |  | |  |
| 289 | *Hymenostegia* |  |  |  | |  |
| 290 | *Hypodaphnis* |  |  |  | |  |
| 291 | *Inga* | -30 | -120 |  | | -35.2 |
| 292 | *Intsia* |  | 90 |  | |  |
| 293 | *Intsia* | 30 | -30 | 30 | |  |
| 294 | *Intsia* | 60 | 43 |  | |  |
| 295 | *Iriartea* |  |  |  | |  |
| 296 | *Irvingia* |  | 50 |  | |  |
| 297 | *Irvingia* | 50 | -100 |  | |  |
| 298 | *Iryanthera* |  |  |  | |  |
| 299 | *Isolona* |  |  |  | |  |
| 300 | *Ixonanthes* |  |  |  | |  |
| 301 | *Jacaranda* | -35 | -115 |  | |  |
| 302 | *Jacaratia* | -20 |  |  | |  |
| 303 | *Julbernardia* |  |  |  | |  |
| 304 | *Khaya* | 60 | -30 |  | |  |
| 305 | *Klaineanthus* |  |  |  | |  |
| 306 | *Klainedoxa* |  |  | 10 | |  |
| 307 | *Knema* |  |  |  | |  |
| 308 | *Koilodepas* | 140 |  |  | |  |
| 309 | *Koompassia* |  |  |  | |  |
| 310 | *Lacistema* | -20 |  |  | |  |
| 311 | *Lacmellea* |  |  |  | |  |
| 312 | *Laetia* | -20 |  |  | |  |
| 313 | *Lannea* | 65 | -70 |  | |  |
| 314 | *Lansium* |  | 0 |  | |  |
| 315 | *Lasiodiscus* |  |  |  | |  |
| 316 | *Lecythis* | -35 |  |  | |  |
| 317 | *Leonia* | -45 |  | 20 | |  |
| 318 | *Lepidobotrys* |  |  |  | |  |
| 319 | *Lepisanthes* |  |  |  | |  |
| 320 | *Leptaulus* | 60 |  |  | |  |
| 321 | *Leptonychia* | 50 |  |  | |  |
| 322 | *Leptonychia* | 130 | 80 |  | |  |
| 323 | *Licania* | -35 |  |  | |  |
| 324 | *Licaria* | -20 |  |  | |  |
| 325 | *Lindackeria* |  | -20 |  | |  |
| 326 | *Lindackeria* | -35 | -90 | 16 | |  |
| 327 | *Lithocarpus* | 160 | 60 |  | |  |
| 328 | *Litsea* |  | 60 |  | |  |
| 329 | *Litsea* | -50 | -160 | 37 | | -15 |
| 330 | *Lonchocarpus* | -35 |  |  | |  |
| 331 | *Lonchocarpus* | 60 | -30 |  | |  |
| 332 | *Lophira* |  |  |  | |  |
| 333 | *Lovoa* |  |  |  | |  |
| 334 | *Luehea* | -20 | -120 |  | | -35.2 |
| 335 | *Lueheopsis* |  |  |  | |  |
| 336 | *Lunania* |  |  | 28 | |  |
| 337 | *Lychnodiscus* |  |  |  | |  |
| 338 | *Mabea* | -20 |  |  | | -30 |
| 339 | *Macaranga* |  | 65 |  | |  |
| 340 | *Macaranga* | 60 | -50 |  | |  |
| 341 | *Maclurodendron* |  |  |  | |  |
| 342 | *Macrolobium* | -20 |  |  | |  |
|  | **Genus** | **Max long** | **Min long** | | **Max lat** | **Min lat** |
| 343 | *Madhuca* |  | 65 |  | |  |
| 344 | *Maesobotrya* |  |  |  | |  |
| 345 | *Maesopsis* |  |  |  | |  |
| 346 | *Magnolia* | -35 | -140 |  | |  |
| 347 | *Magnolia* |  | 60 | 35 | |  |
| 348 | *Mallotus* | 60 | -30 | 40 | |  |
| 349 | *Mallotus* |  | 60 |  | |  |
| 350 | *Malmea* | -45 |  |  | |  |
| 351 | *Mammea* | -35 | -110 |  | |  |
| 352 | *Mangifera* |  | 60 |  | |  |
| 353 | *Manilkara* | -35 | -120 |  | |  |
| 354 | *Manilkara* | 60 | -30 | 38 | |  |
| 355 | *Manilkara* | 160 | 60 |  | |  |
| 356 | *Maprounea* |  | -20 |  | |  |
| 357 | *Maprounea* | -20 |  |  | |  |
| 358 | *Maquira* |  |  |  | |  |
| 359 | *Maranthes* |  | 50 |  | |  |
| 360 | *Maranthes* | 50 | -40 |  | |  |
| 361 | *Mareya* |  |  |  | |  |
| 362 | *Mareyopsis* |  |  |  | |  |
| 363 | *Margaritaria* | -35 |  |  | |  |
| 364 | *Margaritaria* | 60 | -30 | 38 | |  |
| 365 | *Margaritaria* |  | 100 |  | |  |
| 366 | *Markhamia* | 135 | 85 | 38 | |  |
| 367 | *Markhamia* | 50 | -40 |  | |  |
| 368 | *Matayba* | -20 |  |  | | -35.2 |
| 369 | *Matisia* |  |  | 20 | |  |
| 370 | *Mauritia* |  |  |  | |  |
| 371 | *Maytenus* | 65 | -35 |  | |  |
| 372 | *Maytenus* | -35 | -115 |  | |  |
| 373 | *Memecylon* | 60 | -100 |  | |  |
| 374 | *Memecylon* |  | 60 |  | |  |
| 375 | *Mesua* | 130 | -20 |  | |  |
| 376 | *Metrodorea* | -20 |  | 3 | |  |
| 377 | *Mezilaurus* | -20 |  |  | |  |
| 378 | *Mezzettia* |  |  |  | |  |
| 379 | *Miconia* | -30 |  |  | |  |
| 380 | *Micrandra* |  |  |  | | -29.5 |
| 381 | *Micrandropsis* |  |  |  | |  |
| 382 | *Microcos* | 50 | -150 |  | |  |
| 383 | *Microcos* |  | 50 |  | |  |
| 384 | *Micropholis* | -30 |  |  | |  |
| 385 | *Milicia* |  |  |  | |  |
| 386 | *Millettia* | 60 | -40 |  | |  |
| 387 | *Millettia* | 170 | 60 |  | |  |
| 388 | *Minquartia* | -20 |  |  | | -25 |
| 389 | *Monocarpia* |  |  |  | |  |
| 390 | *Monodora* |  | -40 |  | |  |
| 391 | *Mora* | -20 |  |  | |  |
| 392 | *Moultonianthus* |  |  |  | |  |
| 393 | *Mouriri* | -20 |  |  | |  |
| 394 | *Musanga* |  | -40 |  | |  |
| 395 | *Myrcia* | 20 |  |  | | -35 |
| 396 | *Myrciaria* | -10 | -150 | 39 | | -35.2 |
| 397 | *Myrianthus* |  | -60 |  | |  |
| 398 | *Myristica* |  | 60 |  | |  |
| 399 | *Nauclea* |  | 60 |  | |  |
| 400 | *Nauclea* | 50 | -40 |  | |  |
| 401 | *Naucleopsis* | -20 |  | 30 | |  |
|  | **Genus** | **Max long** | **Min long** | | **Max lat** | **Min lat** |
| 402 | *Nealchornea* |  |  |  | |  |
| 403 | *Nectandra* | -30 |  |  | | -35.2 |
| 404 | *Neea* | -30 |  |  | |  |
| 405 | *Neoscortechinia* | 90 |  |  | |  |
| 406 | *Nephelium* |  | 85 | 35 | | -15 |
| 407 | *Nesogordonia* |  |  |  | |  |
| 408 | *Newtonia* |  | -20 |  | |  |
| 409 | *Ochna* | 130 | -35 |  | |  |
| 410 | *Ochthocosmus* | -40 |  |  | |  |
| 411 | *Ochthocosmus* | 8 |  |  | |  |
| 412 | *Ocotea* | 80 | -28 | 18 | |  |
| 413 | *Ocotea* | -35 |  | 30 | |  |
| 414 | *Octoknema* |  |  |  | |  |
| 415 | *Oenocarpus* |  |  |  | |  |
| 416 | *Olax* |  | 60 |  | |  |
| 417 | *Olax* | 60 |  |  | |  |
| 418 | *Oncoba* | 145 | -45 |  | |  |
| 419 | *Oncosperma* |  |  |  | |  |
| 420 | *Ongokea* |  |  |  | |  |
| 421 | *Ormosia* |  | 80 |  | |  |
| 422 | *Ormosia* | -35 |  |  | |  |
| 423 | *Osteophloeum* |  |  |  | |  |
| 424 | *Oubanguia* |  |  |  | |  |
| 425 | *Ouratea* | 60 | -30 |  | | -29.9 |
| 426 | *Ouratea* | -35 |  | 23.5 | | -29.9 |
| 427 | *Oxandra* |  |  |  | |  |
| 428 | *Pachira* | -35 | -125 | 30 | |  |
| 429 | *Palaquium* |  | -145 |  | | -20.5 |
| 430 | *Pancovia* |  | -60 |  | |  |
| 431 | *Panda* |  |  |  | |  |
| 432 | *Paramachaerium* |  |  |  | |  |
| 433 | *Paranephelium* |  |  |  | |  |
| 434 | *Parashorea* |  |  |  | |  |
| 435 | *Parinari* | -35 | -140 | 15 | |  |
| 436 | *Parinari* | 70 | -30 |  | |  |
| 437 | *Parinari* |  | 70 |  | |  |
| 438 | *Parkia* |  | 80 |  | |  |
| 439 | *Parkia* | 60 | -30 |  | |  |
| 440 | *Parkia* | -35 |  |  | |  |
| 441 | *Paropsia* |  |  |  | |  |
| 442 | *Pausandra* | -20 |  |  | |  |
| 443 | *Pausinystalia* | -100 |  |  | |  |
| 444 | *Payena* |  | 80 |  | |  |
| 445 | *Peltogyne* | -20 |  |  | |  |
| 446 | *Pentaclethra* | -40 |  |  | |  |
| 447 | *Pentaclethra* | -40 |  |  | |  |
| 448 | *Pentadesma* |  |  |  | |  |
| 449 | *Perebea* |  |  |  | | -20.5 |
| 450 | *Petersianthus* | 40 |  |  | |  |
| 451 | *Phyllocosmus* | -14 |  |  | |  |
| 452 | *Picralima* |  | -145 |  | |  |
| 453 | *Pimelodendron* |  |  |  | |  |
| 454 | *Piptadeniastrum* |  |  |  | |  |
| 455 | *Pithecellobium* | -35 | -140 | 38 | |  |
| 456 | *Pithecellobium* | 65 |  |  | |  |
| 457 | *Placodiscus* |  |  | 12 | |  |
| 458 | *Plagiostyles* |  |  |  | |  |
| 459 | *Platymiscium* | -25 | -140 |  | | -35.2 |
| 460 | *Platypodium* | -20 |  |  | |  |
| 461 | *Pleurothyrium* |  |  | -35 | |  |
| 462 | *Poecilanthe* | -20 |  |  | | -35.2 |
| 463 | *Polyalthia* | 178 | 60 | 42 | |  |
|  | **Genus** | **Max long** | **Min long** | | **Max lat** | **Min lat** |
| 464 | *Polyalthia* | 60 | -45 |  | |  |
| 465 | *Poraqueiba* | -30 |  |  | |  |
| 466 | *Porterandia* |  | 90 |  | |  |
| 467 | *Poulsenia* | -67 |  |  | | -15 |
| 468 | *Pourouma* | -10 |  |  | |  |
| 469 | *Pouteria* | 59.9 | -34.9 | 20 | |  |
| 470 | *Pouteria* |  | 60 |  | |  |
| 471 | *Pouteria* | -35 | -140 | 40 | |  |
| 472 | *Pradosia* | -20 |  |  | | -30.5 |
| 473 | *Prioria* | 38 | -50 |  | |  |
| 474 | *Prioria* | -50 |  |  | |  |
| 475 | *Protium* | 60 | 40 |  | |  |
| 476 | *Protium* |  | 80 |  | |  |
| 477 | *Protium* | -35 |  |  | |  |
| 478 | *Protomegabaria* | 25 |  |  | |  |
| 479 | *Pseudolachnostylis* |  | -3 | -25.2 | |  |
| 480 | *Pseudolmedia* | -30 |  |  | |  |
| 481 | *Pseudopiptadenia* | -20 | -90 |  | |  |
| 482 | *Pseudospondias* | 40 |  |  | |  |
| 483 | *Pseudoxandra* |  |  |  | |  |
| 484 | *Psidium* | -35 | -101 | 35 | |  |
| 485 | *Psydrax* |  | -30 |  | |  |
| 486 | *Pteleopsis* |  | -40 |  | |  |
| 487 | *Pternandra* |  |  |  | | -10 |
| 488 | *Pterocarpus* | -35 | -120 |  | |  |
| 489 | *Pterocarpus* |  | 60 |  | |  |
| 490 | *Pterocarpus* | 60 | -30 |  | |  |
| 491 | *Pterygota* |  | 60 |  | |  |
| 492 | *Pterygota* | 60 | -30 |  | |  |
| 493 | *Pterygota* | -35 |  |  | |  |
| 494 | *Ptychopyxis* |  |  |  | |  |
| 495 | *Pycnanthus* |  |  |  | |  |
| 496 | *Qualea* | -20 |  |  | | -30.5 |
| 497 | *Quararibea* | -20 |  |  | |  |
| 498 | *Quassia* | -35 | -115 |  | |  |
| 499 | *Quassia* |  | 80 |  | |  |
| 500 | *Quassia* | 60 | -35 |  | |  |
| 501 | *Quiina* | -30 |  |  | | -30.5 |
| 502 | *Rauvolfia* |  | 60 |  | |  |
| 503 | *Rauvolfia* | 60 | -30 | 35 | |  |
| 504 | *Rauvolfia* | -35 | -120 |  | |  |
| 505 | *Rawsonia* |  |  |  | |  |
| 506 | *Rhabdophyllum* | -30 |  |  | |  |
| 507 | *Rhodamnia* |  |  |  | |  |
| 508 | *Rhodostemonodaphne* |  |  |  | |  |
| 509 | *Ricinodendron* |  |  |  | |  |
| 510 | *Rinorea* | -35 |  |  | |  |
| 511 | *Rinorea* |  | 80 |  | |  |
| 512 | *Rinorea* | 60 | -30 |  | |  |
| 513 | *Rinoreocarpus* |  |  |  | |  |
| 514 | *Rollinia* | -20 | -158 |  | | -32 |
| 515 | *Rothmannia* |  | 60 |  | | -20 |
| 516 | *Rothmannia* | 60 | -40 |  | |  |
| 517 | *Roucheria* |  |  |  | |  |
| 518 | *Ruizodendron* | -83 |  |  | |  |
| 519 | *Sacoglottis* |  | -30 |  | |  |
| 520 | *Sacoglottis* | -35 |  |  | |  |
| 521 | *Sagotia* |  |  |  | |  |
| 522 | *Santiria* | 50 |  |  | |  |
|  | **Genus** | **Max long** | **Min long** | | **Max lat** | **Min lat** |
| 523 | *Santiria* | 130 | 50 |  | |  |
| 524 | *Sapium* | -35 |  |  | |  |
| 525 | *Sapium* | 60 | -30 |  | |  |
| 526 | *Saraca* |  | 70 |  | |  |
| 527 | *Sarcaulus* | -20 |  |  | |  |
| 528 | *Sarcotheca* |  |  |  | |  |
| 529 | *Scaphium* | 140 |  | 20 | |  |
| 530 | *Scleronema* |  |  |  | |  |
| 531 | *Scorodophloeus* |  |  |  | |  |
| 532 | *Scottellia* |  |  |  | |  |
| 533 | *Scytopetalum* |  |  |  | |  |
| 534 | *Shorea* | 135 |  |  | |  |
| 535 | *Simaba* | -20 | -98 |  | |  |
| 536 | *Simarouba* | -20 |  |  | | -30.5 |
| 537 | *Sindora* |  | 50 |  | |  |
| 538 | *Siparuna* |  |  |  | |  |
| 539 | *Sloanea* | 60 | 20 |  | |  |
| 540 | *Sloanea* | -35 |  |  | |  |
| 541 | *Sloanea* |  | 60 |  | |  |
| 542 | *Socratea* |  |  |  | |  |
| 543 | *Sorindeia* |  |  |  | |  |
| 544 | *Sorocea* | -20 |  |  | | -35.2 |
| 545 | *Spondias* | -35 | -120 |  | |  |
| 546 | *Spondias* | 80 | -30 | 20 | |  |
| 547 | *Spondias* |  | 80 |  | |  |
| 548 | *Stachyothyrsus* | 20 | -2 |  | |  |
| 549 | *Staudtia* |  |  |  | |  |
| 550 | *Stemonurus* | 160 | 90 |  | |  |
| 551 | *Sterculia* | 175 | 65 |  | |  |
| 552 | *Sterculia* | -35 | -110 |  | |  |
| 553 | *Sterculia* | 70 | -30 |  | |  |
| 554 | *Strephonema* |  |  |  | |  |
| 555 | *Strombosia* |  |  |  | |  |
| 556 | *Strombosiopsis* |  |  |  | |  |
| 557 | *Stryphnodendron* | -20 |  |  | |  |
| 558 | *Swartzia* | -20 |  | 38 | |  |
| 559 | *Symphonia* | -35 |  |  | |  |
| 560 | *Symphonia* |  | -30 |  | |  |
| 561 | *Symplocos* |  | 60 |  | |  |
| 562 | *Symplocos* | -35 |  |  | |  |
| 563 | *Synsepalum* | 60 | -50 |  | |  |
| 564 | *Syzygium* | 60 | -30 | 40 | |  |
| 565 | *Syzygium* |  | 60 | 55 | |  |
| 566 | *Tabebuia* | -25 | -118 | 30 | | -35.2 |
| 567 | *Tabernaemontana* | -35 | -120 | 35 | |  |
| 568 | *Tabernaemontana* | 60 |  | -35 | |  |
| 569 | *Tabernaemontana* | 60 | -30 | 40 | |  |
| 570 | *Tachigali* | -20 |  |  | |  |
| 571 | *Talisia* |  |  |  | |  |
| 572 | *Tapirira* | -20 |  |  | |  |
| 573 | *Tapura* |  | -35 |  | |  |
| 574 | *Tapura* | -35 |  |  | |  |
| 575 | *Tarenna* | 60 | -50 |  | |  |
| 576 | *Tarenna* |  | 60 |  | |  |
| 577 | *Tarrietia* | 20 |  |  | |  |
| 578 | *Teijsmanniodendron* |  |  |  | |  |
| 579 | *Terminalia* |  | 60 |  | |  |
| 580 | *Terminalia* | -35 | -118 |  | | -38 |
| 581 | *Terminalia* | 60 | -20 |  | |  |
| 582 | *Ternstroemia* | -35 |  |  | |  |
|  | **Genus** | **Max long** | **Min long** | | **Max lat** | **Min lat** |
| 583 | *Ternstroemia* | 160 | 60 |  | |  |
| 584 | *Tessmannia* |  | 5 |  | |  |
| 585 | *Tessmannia* | -8 |  |  | |  |
| 586 | *Tetraberlinia* |  |  |  | |  |
| 587 | *Tetragastris* | -20 | -98 |  | |  |
| 588 | *Tetrapleura* |  | -40 |  | |  |
| 589 | *Tetrathylacium* | -95 |  |  | |  |
| 590 | *Tetrorchidium* | -12 |  |  | |  |
| 591 | *Tetrorchidium* | -35 |  |  | |  |
| 592 | *Theobroma* | -35 | -110 | 30 | |  |
| 593 | *Thyrsodium* | -20 |  |  | |  |
| 594 | *Timonius* |  |  |  | |  |
| 595 | *Tovomita* | -20 |  |  | |  |
| 596 | *Trattinnickia* | -20 |  |  | |  |
| 597 | *Treculia* |  |  |  | |  |
| 598 | *Trema* |  | 70 |  | |  |
| 599 | *Trema* | -35 | -120 | 30 | |  |
| 600 | *Trema* | 70 | -30 | 40 | |  |
| 601 | *Tricalysia* | 60 | -30 |  | |  |
| 602 | *Trichilia* | -35 |  |  | |  |
| 603 | *Trichilia* | 60 | -28 |  | |  |
| 604 | *Trichoscypha* |  |  |  | |  |
| 605 | *Tridesmostemon* |  |  |  | |  |
| 606 | *Trilepisium* | 80 | -14 |  | |  |
| 607 | *Triplaris* | -20 | -100 |  | | -35.2 |
| 608 | *Triplochiton* |  |  |  | |  |
| 609 | *Tristania* | 147 |  |  | | -20 |
| 610 | *Tristania* |  | 147 |  | |  |
| 611 | *Tristaniopsis* |  |  |  | |  |
| 612 | *Trophis* |  |  |  | |  |
| 613 | *Turpinia* | -35 |  |  | |  |
| 614 | *Turpinia* |  | 50 | 45 | |  |
| 615 | *Turraeanthus* |  |  |  | |  |
| 616 | *Uapaca* |  | -25 |  | |  |
| 617 | *Unonopsis* | -20 | -95 |  | |  |
| 618 | *Uvariopsis* |  |  |  | |  |
| 619 | *Vangueria* | 80 | -20 |  | |  |
| 620 | *Vantanea* | -20 |  |  | |  |
| 621 | *Vatica* |  |  |  | |  |
| 622 | *Vepris* | 50 | -50 | 15 | |  |
| 623 | *Virola* |  |  |  | |  |
| 624 | *Vismia* |  | -30 |  | |  |
| 625 | *Vismia* | -35 |  |  | |  |
| 626 | *Vitex* | -35 | -115 |  | |  |
| 627 | *Vitex* |  | -30 |  | |  |
| 628 | *Vochysia* | -20 |  |  | |  |
| 629 | *Vouacapoua* |  |  |  | |  |
| 630 | *Xanthophyllum* | -50 |  |  | |  |
| 631 | *Xerospermum* | 90 | 30 |  | |  |
| 632 | *Xylopia* |  | 85 |  | |  |
| 633 | *Xylopia* | -35 |  |  | |  |
| 634 | *Xylopia* | 55 | -30 |  | |  |
| 635 | *Xymalos* |  | 5 |  | |  |
| 636 | *Zanthoxylum* | 60 | -30 | 40 | |  |
| 637 | *Zanthoxylum* | -35 | -140 |  | |  |
| 638 | *Zanthoxylum* | 60 |  |  | |  |
| 639 | *Zygia* | -20 |  |  | |  |

**Table S5**

Summary information about the genera included in this study. Mort., average annual mortality rate for all individual trees in the genus calculated from plot data; Spp., species richness; Distrib., biogeographical setting (Americas, Africa, Asia or multiple continents); Range, extent of occupancy calculated from GBIF records; Dio., genus contains no dioecious species (0) or at least some dioecious species (1); Lifeform, Tree, Shrub or Liana; Biome: MTF, moist tropical forest; SDTF, seasonally dry tropical forest; SUB, sub-tropical forest; SAV, savanna; Elevation, Lowland or Montane (>1500 m). Mean thousand seed mass per genus from (1). Where no specific reference is given, information sourced via (2).

|  | **Genus** | **Mort.** | **Spp.** | **Distrib.** | **Range** | **Dio.** | **Lifeform** | **Biome** | **Elevation** | **Seed mass** | **Source: Breeding system** | | **Source: Biome, Lifeform, Elevation** |
| --- | --- | --- | --- | --- | --- | --- | --- | --- | --- | --- | --- | --- | --- |
|  |  | % a^-1^ |  |  | M km^2^ |  |  |  |  | g |  |  | |
| 1 | *Abarema* | 0.80 | 4 | Americas | 12.50 | 0 | Tree | MTF | Lowland | 122.6 | (3) |  | |
| 2 | *Adelia* | 2.47 | 10 | Americas | 4.93 | 1 | Tree, Shrub | MTF, SUB | Lowland |  | (4) | (4) | |
| 3 | *Adinandra* | 2.51 | 106 | Asia | 10.11 | 0 | Tree, Shrub, Liana | MTF | Lowland |  | (5) | (6) | |
| 4 | *Afrostyrax* | 0.71 | 3 | Africa | 0.91 | 0 | Shrub, Tree | MTF | Lowland |  | (7) | (8) | |
| 5 | *Afzelia* | 1.02 | 12 | Africa | 12.82 | 0 | Tree | MTF | Lowland |  | (7) | (8) | |
| 6 | *Aglaia* | 1.73 | 119 | Asia | 6.58 | 0 | Tree | MTF, SUB | Lowland |  | (9) | (9) | |
| 7 | *Aidia* | 1.15 | 56 | Africa | 16.62 | 0 | Shrub, Tree | MTF | Lowland |  | (7) | (8) | |
| 8 | *Alangium* | 1.14 | 57 | Multiple | 12.41 | 0 | Tree | MTF | Lowland, Montane |  | (10) | (8) | |
| 9 | *Albizia* | 2.01 | 99 | Multiple | 57.57 | 0 | Tree | MTF | Lowland |  | (3) | (3) | |
| 10 | *Alchornea* | 1.92 | 51 | Multiple | 53.66 | 1 | Tree | MTF | Lowland |  | (11) | (3) | |
| 11 | *Allanblackia* | 0.46 | 9 | Africa | 2.24 | 1 | Tree | MTF | Lowland, Montane |  | (11) | (8), | |
| 12 | *Allophylus* | 2.79 | 21 | Multiple | 39.01 | 1 | Tree | MTF | Lowland |  | (3) | (3) | |
| 13 | *Alphonsea* | 1.22 | 38 | Asia | 3.00 | 0 | Tree, Shrub | MTF, SUB | Lowland |  | (12) | (12) | |
| 14 | *Alseis* | 0.36 | 18 | Americas | 7.71 | 0 | Tree | SDTF, MTF | Lowland |  | (3) | (3) | |
| 15 | *Alseodaphne* | 1.82 | 53 | Asia | 4.62 | 0 | Tree | MTF | Lowland |  | (13) | (13) | |
| 16 | *Alstonia* | 1.33 | 44 | Multiple | 11.15 | 0 | Tree, Shrub | MTF | Lowland |  | (14), (15) | (14) | |
| 17 | *Amaioua* | 2.17 | 11 | Multiple | 11.07 | 1 | Tree | MTF | Lowland |  | (11) | (3) | |
| 18 | *Amanoa* | 0.81 | 16 | Multiple | 11.18 | 0 | Tree | MTF | Lowland |  | (3) | (3) | |
| 19 | *Ambelania* | 0.86 | 3 | Americas | 4.15 | 0 | Tree | MTF | Lowland |  | (3) | (3) | |
| 20 | *Ampelocera* | 1.93 | 10 | Americas | 12.11 | 0 | Tree | MTF | Lowland | 485.0 | (3) | (3) | |
| 21 | *Amphimas* | 0.40 | 3 | Africa | 1.99 | 0 | Tree | MTF, SDTF | Lowland |  |  |  | |
| 22 | *Amphirrhox* | 1.10 | 2 | Americas | 8.94 | 0 | Tree | MTF | Lowland |  | (3) | (3) | |
| 23 | *Anacardium* | 0.71 | 13 | Americas | 15.00 | 1 | Tree | MTF | Lowland | 3149.0 | (3) | (3), (16) | |
| 24 | *Anaxagorea* | 2.77 | 25 | Multiple | 10.52 | 0 | Tree | MTF | Lowland |  | (3) | (3) | |
| 25 | *Andira* | 0.97 | 30 | Americas | 17.43 | 0 | Tree | MTF | Lowland | 4365.4 | (3) |  | |
| 26 | *Angylocalyx* | 0.97 | 7 | Africa | 2.89 | 0 | Shrub, Tree | MTF, SDTF | Lowland |  | (15) | (8), (15) | |
| 27 | *Aniba* | 1.81 | 49 | Americas | 10.63 | 0 | Tree | MTF | Lowland | 505.0 | (3) | (3) | |
|  | **Genus** | **Mort.** | **Spp.** | **Distrib.** | **Range** | **Dio.** | **Lifeform** | **Biome** | **Elevation** | **Seed mass** | **Source: Breeding system** | **Source: Biome, Lifeform, Elevation** | |
|  |  | % a^-1^ |  |  | M km^2^ |  |  |  |  | g |  |  | |
| 28 | *Anisophyllea* | 0.80 | 66 | Multiple | 6.58 | 0 | Tree | MTF | Lowland |  | (3) | (3) | |
| 29 | *Anisoptera* | 0.61 | 10 | Asia | 10.65 | 0 | Tree | MTF, SDTF | Lowland |  | (10) | (10) | |
| 30 | *Annickia* | 1.33 | 11 | Africa | 1.55 | 0 | Tree | MTF | Lowland |  | (15) | (8), | |
| 31 | *Annona* | 2.58 | 170 | Multiple | 33.19 | 0 | Tree | MTF | Lowland |  | (3) | (3) | |
| 32 | *Anonidium* | 0.71 | 5 | Africa | 1.12 | 0 | Tree | MTF, SAV | Lowland |  | (15) | (8), (15) | |
| 33 | *Anopyxis* | 1.32 | 1 | Africa | 0.89 | 0 | Tree | MTF | Lowland |  | (17) | (7), (17) | |
| 34 | *Anthonotha* | 1.26 | 17 | Africa | 3.21 | 0 | Shrub, Tree | SAV, MTF, SDTF | Lowland |  | (15) | (8), (15) | |
| 35 | *Antiaris* | 1.05 | 1 | Africa | 9.48 | 1 | Tree | SAV, SDTF, MTF | Lowland, Montane |  | (18) | (8) | |
| 36 | *Antidesma* | 1.50 | 103 | Multiple | 20.42 | 1 | Tree | MTF, SAV | Lowland |  | (11) | (7) | |
| 37 | *Aparisthmium* | 7.47 | 1 | Americas | 8.68 | 1 | Tree | MTF | Lowland |  | (11) | (3) | |
| 38 | *Apeiba* | 1.70 | 10 | Americas | 11.65 | 0 | Tree | MTF | Lowland | 41.8 | (3) | (3) | |
| 39 | *Aporosa* | 1.65 | 85 | Asia | 4.56 | 1 | Tree | MTF | Lowland |  | (11) | (13) | |
| 40 | *Archidendron* | 1.95 | 98 | Asia | 6.26 | 0 | Tree, Shrub | MTF | Lowland |  | (19) | (19) | |
| 41 | *Artocarpus* | 0.85 | 73 | Asia | 7.35 | 0 | Tree | MTF | Lowland |  | (13) | (13) | |
| 42 | *Aspidosperma* | 0.96 | 79 | Americas | 14.72 | 0 | Tree | MTF | Lowland | 523.5 | (3) | (3) | |
| 43 | *Astrocaryum* | 2.20 | 39 | Multiple | 10.73 | 0 | Tree | MTF | Lowland |  | (3) |  | |
| 44 | *Astronium* | 0.51 | 11 | Americas | 14.57 | 1 | Tree | MTF | Lowland | 27.0 | (11) | (16) | |
| 45 | *Attalea* | 1.93 | 40 | Americas | 12.18 | 1 | Tree | MTF | Lowland | 4992.0 | (3), (11) |  | |
| 46 | *Atuna* | 1.41 | 8 | Asia | 3.48 | 0 | Tree | MTF | Lowland |  | (10) | (10) | |
| 47 | *Aucoumea* | 1.29 | 1 | Africa | 0.23 | 1 | Tree | SAV, MTF | Lowland |  | (20) | (20) | |
| 48 | *Aulacocalyx* | 1.52 | 11 | Africa | 3.57 | 0 | Tree, Shrub | MTF | Lowland |  | (7) | (7) | |
| 49 | *Baccaurea* | 1.33 | 51 | Asia | 4.32 | 1 | Tree | MTF | Lowland |  | (11) |  | |
| 50 | *Baphia* | 1.41 | 50 | Africa | 8.03 | 0 | Shrub, Tree | SDTF, MTF | Lowland |  |  |  | |
| 51 | *Barringtonia* | 0.73 | 72 | Multiple | 9.10 | 0 | Tree | MTF | Lowland |  | (21) | (8), (21) | |
| 52 | *Barteria* | 1.64 | 6 | Africa | 1.33 | 0 | Shrub, Tree | MTF | Lowland |  | (15) | (8), (15) | |
| 53 | *Batocarpus* | 1.33 | 3 | Americas | 7.73 | 1 | Tree | MTF | Lowland | 600 | (11) | (3) | |
| 54 | *Bauhinia* | 3.87 | 193 | Multiple | 50.23 | 1 | Tree, Liana | SDTF, MTF | Lowland |  | (11) |  | |
| 55 | *Beilschmiedia* | 1.54 | 268 | Multiple | 31.90 | 0 | Tree | MTF | Lowland, Montane |  | (3) | (3) | |
| 56 | *Berlinia* | 0.99 | 21 | Africa | 4.35 | 0 | Tree | MTF | Lowland |  |  | (8), | |
| 57 | *Bhesa* | 0.88 | 8 | Asia | 2.36 | 0 | Tree | MTF | Lowland |  | (10) | (10) | |
| 58 | *Bixa* | 3.35 | 6 | Americas | 14.13 | 0 | Tree | MTF | Lowland | 25.1 | (3) | (3) | |
| 59 | *Blighia* | 1.10 | 3 | Africa | 4.64 | 1 | Shrub, Tree | MTF, SDTF, SAV | Lowland |  | (22) | (8), (22) | |
| 60 | *Blumeodendron* | 1.29 | 9 | Asia | 1.28 | 1 | Tree | MTF | Lowland |  | (11) | (23) | |
| 61 | *Bocageopsis* | 0.92 | 4 | Americas | 6.70 | 0 | Tree | MTF | Lowland | 200.0 | (3) | (3) | |
| 62 | *Bocoa* | 0.85 | 3 | Multiple | 6.58 | 0 | Tree | MTF | Lowland |  | (3) |  | |
|  | **Genus** | **Mort.** | **Spp.** | **Distrib.** | **Range** | **Dio.** | **Lifeform** | **Biome** | **Elevation** | **Seed mass** | **Source: Breeding system** | **Source: Biome, Lifeform, Elevation** | |
|  |  | % a^-1^ |  |  | M km^2^ |  |  |  |  | g |  |  | |
| 63 | *Brachystegia* | 1.01 | 33 | Africa | 6.14 | 0 | Tree | MTF, SDTF, SAV | Lowland |  | (15) | (8) | |
| 64 | *Bridelia* | 2.36 | 50 | Multiple | 23.80 | 1 | Tree, Shrub | MTF, SUB | Lowland |  | (11), (24) | (24), (25) | |
| 65 | *Brosimum* | 0.75 | 19 | Americas | 13.57 | 1 | Tree | MTF | Lowland | 1333.2 | (11) | (3) | |
| 66 | *Buchanania* | 3.70 | 26 | Asia | 4.97 | 0 | Tree | MTF | Lowland |  | (10) | (10) | |
| 67 | *Buchenavia* | 0.65 | 30 | Americas | 13.66 | 0 | Tree | MTF | Lowland | 1329.4 | (3) | (3) | |
| 68 | *Bussea* | 0.84 | 7 | Africa | 1.38 | 0 | Shrub, Tree | SDTF, MTF | Lowland |  | (15) | (8) | |
| 69 | *Byrsonima* | 2.45 | 164 | Americas | 13.99 | 0 | Tree | MTF, SDTF | Lowland |  | (3) | (3) | |
| 70 | *Calatola* | 1.65 | 6 | Americas | 6.77 | 1 | Tree | MTF | Lowland | 9700.0 | (3) | (3) | |
| 71 | *Calophyllum* | 2.04 | 185 | Multiple | 26.23 | 1 | Tree | MTF | Lowland |  | (3) | (3) | |
| 72 | *Calpocalyx* | 1.58 | 11 | Africa | 0.58 | 0 | Tree | MTF | Lowland |  | (7) | (8) (7) | |
| 73 | *Calyptranthes* | 1.42 | 274 | Americas | 15.18 | 0 | Tree | MTF | Lowland | 442.3 | (3) | (3) | |
| 74 | *Canarium* | 1.82 | 121 | Multiple | 17.44 | 1 | Tree | MTF | Lowland |  | (11) |  | |
| 75 | *Canthium* | 2.23 | 80 | Multiple | 20.40 | 1 | Shrub, Tree | MTF | Lowland, Montane |  | (26) | (26) | |
| 76 | *Capirona* | 2.11 | 1 | Americas | 6.56 | 0 | Tree | MTF | Lowland |  | (3) | (3) | |
| 77 | *Caraipa* | 0.93 | 42 | Americas | 10.73 | 0 | Tree | MTF | Lowland | 120.0 | (3) | (3) | |
| 78 | *Carallia* | 3.14 | 15 | Asia | 6.31 | 0 | Tree | MTF | Lowland |  | (10) | (10) | |
| 79 | *Carapa* | 1.02 | 25 | Multiple | 10.29 | 0 | Tree | MTF | Lowland |  | (3) | (3) | |
| 80 | *Caryocar* | 0.86 | 16 | Americas | 10.75 | 0 | Tree | MTF | Lowland | 46640 | (3) | (3) | |
| 81 | *Casearia* | 2.33 | 245 | Multiple | 26.92 | 0 | Tree | MTF, SDTF | Lowland |  | (3) |  | |
| 82 | *Cassipourea* | 1.04 | 72 | Multiple | 16.16 | 0 | Tree | MTF | Lowland |  | (3) | (3) | |
| 83 | *Castanopsis* | 2.45 | 143 | Asia | 4.71 | 0 | Tree | MTF, TEMP, SUB | Lowland, Montane |  | (10) | (10) | |
| 84 | *Castilla* | 1.23 | 3 | Multiple | 8.00 | 1 | Tree | MTF | Lowland |  | (11) | (3) | |
| 85 | *Catostemma* | 0.95 | 15 | Americas | 4.51 | 0 | Tree | MTF | Lowland, Montane |  | (3) |  | |
| 86 | *Cecropia* | 3.35 | 63 | Americas | 16.31 | 1 | Tree | MTF | Lowland | 0.8 | (11) | (3) | |
| 87 | *Ceiba* | 1.20 | 19 | Multiple | 20.10 | 0 | Tree | MTF, SDTF | Lowland |  | (3) | (3) | |
| 88 | *Celtis* | 0.96 | 69 | Multiple | 58.58 | 0 | Shrub, Tree, Liana | MTF, MED | Lowland, Montane |  | (3) | (3) | |
| 89 | *Centroplacus* | 2.99 | 1 | Africa | 0.25 | 1 | Tree | MTF | Lowland |  | (11) | (8) | |
| 90 | *Cephalomappa* | 2.30 | 6 | Asia | 0.14 | 0 | Tree | MTF | Lowland |  | (27) | (27) | |
| 91 | *Chaetocarpus* | 0.78 | 16 | Multiple | 13.66 | 1 | Tree, Shrub | MTF, SUB | Lowland |  | (11) | (3) | |
| 92 | *Cheiloclinium* | 1.59 | 13 | Americas | 12.58 | 0 | Liana, Tree | MTF | Lowland, Montane |  | (3) | (3) | |
| 93 | *Chimarrhis* | 0.99 | 15 | Americas | 6.94 | 0 | Tree | MTF | Lowland |  | (3) | (3) | |
| 94 | *Chionanthus* | 2.87 | 141 | Multiple | 40.98 | 0 | Tree | MTF, SDTF | Lowland, Montane |  | (3) | (3) | |
| 95 | *Chisocheton* | 1.25 | 50 | Asia | 4.04 | 1 | Tree | MTF | Lowland |  | (10) | (10) | |
| 96 | *Chlorocardium* | 0.20 | 3 | Americas | 1.68 | 0 | Tree | MTF | Lowland | 35480 | (3) | (3) | |
| 97 | *Chrysochlamys* | 1.90 | 36 | Americas | 5.85 | 1 | Tree | MTF | Lowland | 101.1 | (3) | (3) | |
| 98 | *Chrysophyllum* | 0.95 | 41 | Multiple | 32.80 | 1 | Tree | MTF | Lowland |  | (3) | (28) | |
| 99 | *Cinnamomum* | 1.67 | 252 | Multiple | 11.26 | 0 | Tree | MTF | Lowland |  | (3) | (3) | |
| 100 | *Clarisia* | 1.25 | 4 | Americas | 12.46 | 1 | Tree | MTF | Lowland | 1782.9 | (11) | (3) | |
| 101 | *Cleistanthus* | 1.65 | 134 | Multiple | 13.13 | 1 | Shrub, Tree | MTF | Lowland |  | (11) | (8) | |
| 102 | *Cleistopholis* | 2.85 | 4 | Africa | 2.55 | 0 | Shrub, Tree | MTF | Lowland |  | (15) | (8) | |
|  | **Genus** | **Mort.** | **Spp.** | **Distrib.** | **Range** | **Dio.** | **Lifeform** | **Biome** | **Elevation** | **Seed mass** | **Source: Breeding system** | **Source: Biome, Lifeform, Elevation** | |
|  |  | % a^-1^ |  |  | M km^2^ |  |  |  |  | g |  |  | |
| 103 | *Coccoloba* | 1.76 | 178 | Americas | 15.82 | 1 | Tree | MTF | Lowland | 144.8 | (11) | (3) | |
| 104 | *Coelocaryon* | 1.15 | 4 | Africa | 3.06 | 1 | Tree | MTF | Lowland |  | (11) | (8) | |
| 105 | *Coffea* | 2.01 | 132 | Africa | 17.69 | 0 | Shrub, Tree | SDTF, MTF | Lowland |  | (3) | (8) | |
| 106 | *Cola* | 1.01 | 134 | Africa | 12.93 | 1 | Shrub, Tree | MTF | Lowland |  | (29) | (8) | |
| 107 | *Colubrina* | 2.26 | 38 | Multiple | 22.68 | 0 | Tree | MTF | Lowland |  | (3) | (3) | |
| 108 | *Combretum* | 1.70 | 283 | Multiple | 35.35 | 0 | Shrub, Tree, Liana | MTF, SAV | Lowland |  |  | (8) | |
| 109 | *Conceveiba* | 1.18 | 15 | Multiple | 7.14 | 1 | Tree | MTF | Lowland |  | (11) | (3) | |
| 110 | *Copaifera* | 1.81 | 40 | Multiple | 13.69 | 0 | Tree | MTF | Lowland |  | (3) | (3) | |
| 111 | *Cordia* | 2.15 | 225 | Multiple | 48.60 | 1 | Tree | MTF | Lowland |  | (11) | (3) | |
| 112 | *Corynanthe* | 1.81 | 7 | Africa | 2.01 | 0 | Tree | MTF | Lowland |  | (20), (30) | (20), (30) | |
| 113 | *Corythophora* | 0.56 | 4 | Americas | 2.51 | 0 | Tree | MTF | Lowland |  | (31) | (31) | |
| 114 | *Couepia* | 0.98 | 63 | Americas | 12.27 | 0 | Tree | MTF | Lowland | 5020.0 | (3) | (32) | |
| 115 | *Coula* | 0.60 | 1 | Africa | 0.66 | 0 | Tree | MTF | Lowland |  |  | (8) | |
| 116 | *Couratari* | 0.30 | 19 | Americas | 7.02 | 0 | Tree | MTF | Lowland | 147.2 | (3) | (3) | |
| 117 | *Cratoxylum* | 2.00 | 7 | Asia | 3.93 | 0 | Tree, Shrub | MTF | Lowland |  | (10) | (10) | |
| 118 | *Croton* | 3.64 | 1150 | Multiple | 56.51 | 1 | Shrub, Tree, Herb | MTF | Lowland |  | (11) | (3) | |
| 119 | *Crudia* | 0.61 | 37 | Multiple | 8.13 | 0 | Tree | MTF | Lowland |  | (3) | (3) | |
| 120 | *Cryptocarya* | 1.55 | 362 | Multiple | 77.68 | 0 | Tree | MTF | Lowland, Montane |  | (3), (33) | (3), (34) | |
| 121 | *Ctenolophon* | 1.35 | 2 | Multiple | 1.23 | 0 | Tree | MTF | Lowland |  | (10) | (10) | |
| 122 | *Cupania* | 1.34 | 58 | Americas | 15.61 | 1 | Tree | MTF, SUB | Lowland, Montane |  | (3) | (3) | |
| 123 | *Cyathocalyx* | 2.53 | 7 | Asia | 4.03 | 0 | Tree | MTF | Lowland |  | (35) |  | |
| 124 | *Cylicodiscus* | 0.27 | 1 | Africa | 0.30 | 0 | Tree | MTF | Lowland |  | (7) | (7), (8) | |
| 125 | *Cynometra* | 0.75 | 112 | Multiple | 22.91 | 0 | Tree | MTF | Lowland |  | (3) | (3) | |
| 126 | *Dacryodes* | 1.07 | 71 | Multiple | 8.55 | 1 | Tree | MTF | Lowland |  | (3) | (3), (16) | |
| 127 | *Dactyladenia* | 1.16 | 31 | Africa | 5.91 | 0 | Shrub, Tree | MTF, SAV | Lowland |  | (36) | (8); (36) | |
| 128 | *Dehaasia* | 0.69 | 43 | Asia | 1.98 | 0 | Tree, Shrub | MTF, SUB | Lowland, Montane |  | (33) |  | |
| 129 | *Dendrobangia* | 0.85 | 2 | Americas | 10.09 | 0 | Tree | MTF | Lowland, Montane |  | (3) | (3) | |
| 130 | *Dendropanax* | 1.87 | 95 | Americas | 16.61 | 1 | Tree | MTF | Lowland | 8.2 | (11) | (3) | |
| 131 | *Desbordesia* | 0.67 | 1 | Africa | 0.28 | 0 | Tree | MTF | Lowland |  |  | (20) | |
| 132 | *Desplatsia* | 1.54 | 4 | Africa | 2.61 | 0 | Shrub, Tree | MTF | Lowland |  | (15) | (8), (15), | |
| 133 | *Dialium* | 1.09 | 37 | Multiple | 21.72 | 0 | Tree | MTF | Lowland |  | (3) |  | |
| 134 | *Dichostemma* | 1.26 | 2 | Africa | 1.09 | 0 | Tree | MTF | Lowland |  |  | (8), | |
| 135 | *Dicorynia* | 0.55 | 2 | Americas | 2.90 | 0 | Tree | MTF | Lowland |  |  |  | |
| 136 | *Dicymbe* | 1.07 | 20 | Americas | 3.45 | 0 | Tree | MTF | Lowland | 6632.8 |  |  | |
| 137 | *Dicypellium* | 1.83 | 2 | Americas | 0.95 | 0 | Tree | MTF | Lowland |  |  |  | |
| 138 | *Dillenia* | 1.09 | 60 | Asia | 4.71 | 0 | Tree, Shrub | MTF, SUB | Lowland |  | (37) | (37) | |
| 139 | *Diogoa* | 0.71 | 2 | Africa | 0.41 | 0 | Tree | MTF | Lowland |  |  | (8), (20) | |
| 140 | *Diospyros* | 1.13 | 779 | Multiple | 54.10 | 1 | Tree | MTF | Lowland |  | (11) | (3) | |
| 141 | *Diplorhynchus* | 3.43 | 1 | Africa | 4.81 | 0 | Shrub, Tree | MTF, SDTF | Lowland |  | (38) | (38) | |
| 142 | *Diplospora* | 0.92 | 23 | Asia | 5.00 | 0 | Shrub, Tree | MTF | Lowland |  |  |  | |
|  | **Genus** | **Mort.** | **Spp.** | **Distrib.** | **Range** | **Dio.** | **Lifeform** | **Biome** | **Elevation** | **Seed mass** | **Source: Breeding system** | **Source: Biome, Lifeform, Elevation** | |
|  |  | % a^-1^ |  |  | M km^2^ |  |  |  |  | g |  |  | |
| 143 | *Diplotropis* | 0.74 | 10 | Americas | 7.34 | 0 | Tree | MTF | Lowland | 160.0 | (3) |  | |
| 144 | *Dipterocarpus* | 0.86 | 65 | Asia | 1.78 | 0 | Tree | MTF | Lowland |  | (13) | (13) | |
| 145 | *Dipteryx* | 0.46 | 11 | Americas | 10.04 | 0 | Tree | MTF | Lowland | 5949.2 | (3) |  | |
| 146 | *Discoglypremna* | 3.08 | 1 | Africa | 1.33 | 1 | Tree | MTF | Lowland |  | (11) | (8) | |
| 147 | *Distemonanthus* | 0.84 | 1 | Africa | 1.08 | 0 | Tree | MTF | Lowland |  |  | (8) | |
| 148 | *Dryobalanops* | 0.86 | 7 | Asia | 0.07 | 0 | Tree | MTF | Lowland |  | (10), (39) | (40) | |
| 149 | *Drypetes* | 1.12 | 213 | Multiple | 32.85 | 1 | Tree | MTF | Lowland |  | (11) | (3) | |
| 150 | *Duguetia* | 1.44 | 95 | Multiple | 13.52 | 0 | Tree | MTF | Lowland |  | (3) | (3) | |
| 151 | *Durio* | 0.81 | 28 | Asia | 2.09 | 0 | Tree | MTF | Lowland |  | (41) | (41) | |
| 152 | *Duroia* | 1.47 | 37 | Americas | 7.86 | 1 | Tree, Shrub | MTF | Lowland, Montane |  | (11) | (3) | |
| 153 | *Dyera* | 1.37 | 2 | Asia | 0.05 | 0 | Tree | MTF | Lowland |  | (10) | (10) | |
| 154 | *Dysoxylum* | 0.97 | 35 | Asia | 4.25 | 1 | Tree, Shrub | MTF | Lowland, Montane |  | (10) | (10) | |
| 155 | *Ecclinusa* | 0.70 | 12 | Americas | 7.93 | 1 | Tree | MTF | Lowland | 575 | (3) | (28) | |
| 156 | *Elaeocarpus* | 2.48 | 488 | Asia | 7.24 | 1 | Tree | MTF | Lowland |  | (11) | (11), (13) | |
| 157 | *Elateriospermum* | 0.69 | 1 | Asia | 0.34 | 0 | Tree | MTF | Lowland |  | (42) | (42) | |
| 158 | *Endlicheria* | 2.99 | 60 | Americas | 11.09 | 1 | Tree | MTF | Lowland | 943.3 | (11) | (3) | |
| 159 | *Englerophytum* | 1.09 | 19 | Africa | 4.75 | 0 | Shrub, Tree | MTF | Lowland, Montane |  | (43) | (20); (8) | |
| 160 | *Entandrophragma* | 0.85 | 11 | Africa | 6.79 | 0 | Tree | MTF, SDTF | Lowland, Montane |  | (44) | (44) | |
| 161 | *Eperua* | 0.71 | 16 | Americas | 3.26 | 0 | Tree | MTF | Lowland | 70.0 |  |  | |
| 162 | *Eriocoelum* | 0.96 | 11 | Africa | 4.59 | 1 | Shrub, Tree | MTF, SAV | Lowland |  | (15) | (8), (15) | |
| 163 | *Erisma* | 0.79 | 21 | Americas | 9.79 | 0 | Tree | MTF | Lowland |  | (3) | (3) | |
| 164 | *Erythrina* | 3.09 | 128 | Multiple | 48.88 | 0 | Tree | SDTF, MTF | Lowland |  | (3) |  | |
| 165 | *Erythrophleum* | 1.27 | 10 | Africa | 10.04 | 0 | Tree | MTF, SDTF, SAV | Lowland |  | (15) | (8), (15), | |
| 166 | *Eschweilera* | 0.69 | 97 | Americas | 12.88 | 0 | Tree | MTF | Lowland | 2926.1 | (3) | (3) | |
| 167 | *Eugenia* | 1.59 | 1219 | Multiple | 28.08 | 1 | Tree, Shrub | MTF, SUB | Lowland, Montane |  | (11) | (3) | |
| 168 | *Eusideroxylon* | 0.42 | 1 | Asia | 0.33 | 0 | Tree | MTF | Lowland, Montane |  | (45) | (45) | |
| 169 | *Euterpe* | 3.39 | 7 | Americas | 10.31 | 0 | Tree | MTF | Lowland | 70.0 | (3) |  | |
| 170 | *Ficus* | 3.42 | 884 | Multiple | 80.80 | 1 | Tree, Epiphyte | MTF | Lowland, Montane |  | (11) | (3) | |
| 171 | *Funtumia* | 1.74 | 2 | Africa | 4.61 | 0 | Shrub, Tree | MTF | Lowland |  | (7), (46) | (15) | |
| 172 | *Fusaea* | 1.76 | 2 | Americas | 7.42 | 0 | Tree | MTF | Lowland |  | (3) | (3) | |
| 173 | *Garcinia* | 1.56 | 406 | Multiple | 33.54 | 1 | Tree | MTF | Lowland | 2173.0 | (11) | (3) | |
| 174 | *Geissospermum* | 0.24 | 5 | Americas | 3.74 | 0 | Tree | MTF | Lowland | 483.3 | (3) | (3) | |
| 175 | *Gilbertiodendron* | 0.63 | 38 | Africa | 2.56 | 0 | Tree | MTF | Lowland |  | (47) | (8), | |
| 176 | *Gironniera* | 1.38 | 6 | Asia | 4.49 | 0 | Shrub, Tree | MTF | Lowland, Montane |  | (10) | (10) | |
| 177 | *Gluta* | 0.80 | 35 | Asia | 1.00 | 0 | Tree | MTF | Lowland |  | (10) | (10) | |
| 178 | *Gonocaryum* | 0.83 | 11 | Asia | 1.30 | 0 | Shrub, Tree | MTF, SUB | Lowland |  | (48) | (19) | |
|  | **Genus** | **Mort.** | **Spp.** | **Distrib.** | **Range** | **Dio.** | **Lifeform** | **Biome** | **Elevation** | **Seed mass** | **Source: Breeding system** | **Source: Biome, Lifeform, Elevation** | |
|  |  | % a^-1^ |  |  | M km^2^ |  |  |  |  | g |  |  | |
| 179 | *Gonystylus* | 1.62 | 32 | Asia | 2.27 | 0 | Tree | MTF | Lowland | 9091 | (10) | (10) | |
| 180 | *Goupia* | 0.76 | 2 | Americas | 6.59 | 0 | Tree | MTF | Lowland |  | (3) | (3) | |
| 181 | *Greenwayodendron* | 2.23 | 6 | Africa | 4.08 | 0 | Tree | MTF | Lowland |  | (15), (49) | (15); (49) | |
| 182 | *Grewia* | 1.71 | 277 | Multiple | 49.61 | 0 | Shrub, Tree | SAV, MTF | Lowland |  | (7), (15) | (7), (15) | |
| 183 | *Grossera* | 1.52 | 9 | Africa | 1.70 | 1 | Shrub, Tree | MTF | Lowland |  | (11) | (7) | |
| 184 | *Guapira* | 0.79 | 74 | Americas | 15.11 | 1 | Tree | MTF | Lowland | 40.7 | (11) | (3) | |
| 185 | *Guarea* | 1.56 | 70 | Multiple | 17.21 | 1 | Tree | MTF | Lowland | 1156.2 | (11) | (3) | |
| 186 | *Guatteria* | 2.56 | 184 | Americas | 12.98 | 0 | Tree | MTF | Lowland | 150 | (3) | (3) | |
| 187 | *Guazuma* | 2.06 | 4 | Americas | 15.53 | 0 | Tree | MTF, SDTF | Lowland |  | (3) | (3) | |
| 188 | *Guettarda* | 1.24 | 145 | Americas | 14.89 | 1 | Tree, Shrub | SDTF, MTF | Lowland |  | (3) | (3), (50) | |
| 189 | *Gustavia* | 0.57 | 46 | Americas | 8.94 | 0 | Tree | MTF | Lowland | 5150.0 | (3) | (3) | |
| 190 | *Gymnacranthera* | 1.48 | 7 | Asia | 1.35 | 1 | Shrub, Tree | SDTF, MTF | Lowland |  | (11) | (19) | |
| 191 | *Hasseltia* | 2.35 | 6 | Americas | 6.07 | 0 | Tree | MTF | Lowland |  | (3) |  | |
| 192 | *Heisteria* | 0.85 | 38 | Multiple | 15.05 | 0 | Tree | MTF | Lowland |  | (3) | (3) | |
| 193 | *Helicostylis* | 1.13 | 8 | Americas | 8.45 | 1 | Tree | MTF | Lowland | 230.0 | (11) | (3) | |
| 194 | *Heritiera* | 1.41 | 39 | Asia | 4.20 | 0 | Tree | MTF, SDTF | Lowland, Montane |  |  | (8), (15) | |
| 195 | *Hevea* | 0.64 | 10 | Americas | 10.40 | 0 | Tree | MTF | Lowland | 3179.4 | (3) | (3) | |
| 196 | *Hexalobus* | 2.39 | 5 | Africa | 9.55 | 0 | Shrub, Tree | MTF | Lowland |  | (15) | (8), (15) | |
| 197 | *Hirtella* | 0.78 | 110 | Multiple | 15.99 | 0 | Tree | MTF | Lowland |  | (3) | (32) | |
| 198 | *Homalium* | 0.87 | 176 | Multiple | 26.15 | 0 | Tree | MTF | Lowland, Montane |  | (3) | (7) | |
| 199 | *Hopea* | 1.44 | 114 | Asia | 4.46 | 0 | Tree | MTF | Lowland |  | (13) | (13) | |
| 200 | *Horsfieldia* | 1.57 | 106 | Asia | 6.59 | 1 | Shrub, Tree | MTF | Lowland |  | (11) | (19) | |
| 201 | *Hura* | 0.41 | 2 | Americas | 10.26 | 0 | Tree | MTF | Lowland, Montane |  | (3) | (3) | |
| 202 | *Hydnocarpus* | 1.15 | 44 | Asia | 2.52 | 1 | Tree | MTF | Lowland |  | (51) | (51) | |
| 203 | *Hylodendron* | 1.74 | 1 | Africa | 0.34 | 0 | Tree | MTF | Lowland |  |  | (20) | |
| 204 | *Hymenaea* | 0.55 | 22 | Multiple | 14.25 | 0 | Tree | MTF | Lowland |  | (3) |  | |
| 205 | *Hymenocardia* | 2.34 | 6 | Africa | 7.50 | 1 | Shrub, Tree | MTF, Savanna | Lowland |  | (11) | (7) | |
| 206 | *Hymenostegia* | 1.03 | 15 | Africa | 1.19 | 0 | Shrub, Tree | MTF, SDTF | Lowland |  | (7) | (8) | |
| 207 | *Hypodaphnis* | 1.79 | 1 | Africa | 0.27 | 1 | Tree | MTF | Lowland |  | (7) | (7), (8) | |
| 208 | *Inga* | 2.78 | 281 | Americas | 15.52 | 0 | Tree | MTF | Lowland | 776.4 | (3) |  | |
| 209 | *Intsia* | 0.66 | 2 | Asia | 5.48 | 0 | Tree | MTF | Lowland |  | (10) | (10) | |
| 210 | *Iriartea* | 2.11 | 1 | Americas | 4.34 | 0 | Tree | MTF | Lowland | 3233.3 | (3) |  | |
| 211 | *Irvingia* | 1.07 | 7 | Multiple | 3.10 | 0 | Tree | MTF | Lowland |  |  | (8) | |
|  | **Genus** | **Mort.** | **Spp.** | **Distrib.** | **Range** | **Dio.** | **Lifeform** | **Biome** | **Elevation** | **Seed mass** | **Source: Breeding system** | **Source: Biome, Lifeform, Elevation** | |
|  |  | % a^-1^ |  |  | M km^2^ |  |  |  |  | g |  |  | |
| 212 | *Iryanthera* | 1.56 | 22 | Americas | 7.31 | 1 | Tree | MTF | Lowland | 2033.3 | (11) | (3) | |
| 213 | *Isolona* | 1.47 | 20 | Africa | 4.87 | 0 | Shrub, Tree | MTF | Lowland |  | (15) | (7), (8), (15) | |
| 214 | *Ixonanthes* | 1.33 | 3 | Asia | 4.59 | 0 | Tree | MTF | Lowland |  | (10) | (10) | |
| 215 | *Jacaranda* | 1.27 | 49 | Americas | 15.20 | 0 | Tree | MTF | Lowland | 7.5 | (3) | (3) | |
| 216 | *Jacaratia* | 4.72 | 7 | Americas | 12.62 | 1 | Tree | MTF | Lowland, Montane |  | (11) | (3) | |
| 217 | *Julbernardia* | 1.59 | 10 | Africa | 4.56 | 0 | Shrub, Tree | MTF, SDTF, Savanna | Lowland, Montane |  | (15) | (8), (15) | |
| 218 | *Khaya* | 0.99 | 8 | Africa | 8.59 | 0 | Tree | MTF, Savanna | Lowland |  |  | (8) | |
| 219 | *Klaineanthus* | 1.00 | 1 | Africa | 0.39 | 1 | Tree | MTF | Lowland |  | (11) | (8) | |
| 220 | *Klainedoxa* | 0.62 | 2 | Africa | 2.39 | 0 | Tree | MTF, Savanna | Lowland |  |  | (8) | |
| 221 | *Knema* | 1.29 | 96 | Asia | 5.62 | 1 | Tree | MTF, SDTF | Lowland, Montane |  | (11) | (19) | |
| 222 | *Koilodepas* | 0.88 | 12 | Asia | 0.50 | 0 | Tree | MTF | Lowland |  | (52) | (52) | |
| 223 | *Koompassia* | 1.19 | 3 | Asia | 0.22 | 1 | Tree | MTF | Lowland |  |  |  | |
| 224 | *Lacistema* | 3.20 | 12 | Americas | 12.01 | 0 | Shrub, Tree | MTF | Lowland |  | (3) | (3) | |
| 225 | *Lacmellea* | 0.82 | 24 | Americas | 6.78 | 0 | Tree | MTF | Lowland | 341.8 | (3) | (3) | |
| 226 | *Laetia* | 1.61 | 9 | Americas | 12.49 | 0 | Tree | MTF | Lowland | 5.4 | (3) |  | |
| 227 | *Lannea* | 1.17 | 36 | Africa | 12.24 | 1 | Shrub, Tree | MTF, SDTF, Savanna | Lowland, Montane |  | (11) | (8), (22) | |
| 228 | *Lansium* | 1.03 | 3 | Asia | 0.97 | 1 | Tree | MTF | Lowland |  | (10) | (10) | |
| 229 | *Lasiodiscus* | 1.03 | 12 | Africa | 3.37 | 0 | Shrub, Tree | MTF, SDTF | Lowland |  | (15) | (8), (15) | |
| 230 | *Lecythis* | 0.47 | 32 | Americas | 12.33 | 0 | Tree | MTF | Lowland | 1607.5 | (3) | (3) | |
| 231 | *Leonia* | 1.52 | 23 | Americas | 7.09 | 0 | Tree | MTF | Lowland | 717.0 | (3) | (3) | |
| 232 | *Lepidobotrys* | 0.88 | 1 | Africa | 0.23 | 1 | Tree | MTF | Lowland |  | (11) | (20) | |
| 233 | *Lepisanthes* | 0.67 | 31 | Multiple | 19.05 | 1 | Tree, Shrub | MTF | Lowland |  | (10) | (10) | |
| 234 | *Leptaulus* | 2.49 | 6 | Africa | 4.90 | 0 | Shrub, Tree | MTF | Lowland |  | (44) | (8), (44) | |
| 235 | *Leptonychia* | 1.55 | 38 | Africa | 3.87 | 0 | Shrub, Tree | MTF | Lowland |  | (15) | (8) | |
| 236 | *Licania* | 0.96 | 103 | Multiple | 14.49 | 0 | Tree | MTF | Lowland |  | (3) | (32) | |
| 237 | *Licaria* | 1.23 | 85 | Americas | 13.30 | 0 | Tree | MTF, SDTF | Lowland |  | (3) | (3) | |
| 238 | *Lindackeria* | 1.81 | 14 | Multiple | 11.16 | 0 | Tree | MTF | Lowland |  | (3) | (3) | |
| 239 | *Lithocarpus* | 2.04 | 344 | Asia | 6.48 | 0 | Shrub, Tree | MTF, SUB | Lowland, Montane |  | (10) | (10) | |
| 240 | *Litsea* | 1.97 | 392 | Asia | 23.85 | 1 | Shrub, Tree | MTF, SUB | Lowland |  | (11) |  | |
| 241 | *Lonchocarpus* | 1.85 | 166 | Multiple | 25.88 | 0 | Tree | MTF | Lowland |  | (3) |  | |
| 242 | *Lophira* | 0.76 | 2 | Africa | 2.23 | 0 | Tree | MTF, SAV | Lowland |  |  | (8) | |
| 243 | *Lovoa* | 1.51 | 2 | Africa | 1.57 | 0 | Tree | MTF | Lowland |  | (44) | (8) | |
| 244 | *Luehea* | 0.95 | 19 | Americas | 14.66 | 0 | Tree | MTF, SDTF | Lowland, Montane |  | (3) | (3) | |
| 245 | *Lueheopsis* | 1.05 | 6 | Americas | 6.70 | 0 | Tree | MTF | Lowland |  | (3) | (3) | |
| 246 | *Lunania* | 3.23 | 15 | Americas | 4.44 | 0 | Tree | MTF | Lowland | 0.6 | (3) |  | |
| 247 | *Mabea* | 1.83 | 39 | Americas | 13.21 | 0 | Shrub, Tree | MTF | Lowland |  | (3) | (3) | |
| 248 | *Macaranga* | 3.35 | 307 | Multiple | 29.94 | 1 | Shrub, Tree | MTF, SDTF | Lowland, Montane |  | (11) | (8), (53) | |
|  | **Genus** | **Mort.** | **Spp.** | **Distrib.** | **Range** | **Dio.** | **Lifeform** | **Biome** | **Elevation** | **Seed mass** | **Source: Breeding_system** | **Source: Biome, Lifeform, Elevation** | |
|  |  | % a^-1^ |  |  | M km^2^ |  |  |  |  | g |  |  | |
| 249 | *Maclurodendron* | 1.64 | 6 | Asia | 0.59 | 1 | Tree | MTF | Lowland |  | (10) | (10) | |
| 250 | *Macrolobium* | 1.12 | 75 | Americas | 8.35 | 0 | Tree | MTF | Lowland | 3382.7 | (3) |  | |
| 251 | *Madhuca* | 1.02 | 118 | Asia | 4.02 | 0 | Tree | MTF | Lowland |  | (54) | (54) | |
| 252 | *Maesobotrya* | 1.99 | 19 | Africa | 3.21 | 1 | Shrub, Tree | MTF | Lowland |  | (11) | (8) | |
| 253 | *Maesopsis* | 2.13 | 1 | Africa | 1.72 | 0 | Tree | MTF, SAV | Lowland, Montane |  | (15) | (8) | |
| 254 | *Magnolia* | 0.37 | 365 | Multiple | 24.28 | 0 | Shrub, Tree | MTF, TEMP | Lowland, Montane |  | (3) | (3) | |
| 255 | *Mallotus* | 1.38 | 118 | Multiple | 29.43 | 1 | Tree, Shrub | MTF, SDTF | Lowland |  | (11) | (7) | |
| 256 | *Malmea* | 1.37 | 6 | Americas | 4.67 | 0 | Tree | MTF | Lowland | 1184 | (3) | (3) | |
| 257 | *Mammea* | 1.31 | 45 | Multiple | 3.81 | 0 | Tree | MTF | Lowland, Montane |  | (7) | (3), (8) | |
| 258 | *Mangifera* | 1.04 | 64 | Asia | 6.84 | 0 | Tree | MTF | Lowland, Montane |  | (10) | (10) | |
| 259 | *Manilkara* | 0.52 | 77 | Multiple | 36.38 | 0 | Tree | MTF | Lowland |  | (3) | (55) | |
| 260 | *Maprounea* | 2.43 | 5 | Multiple | 17.59 | 1 | Tree | MTF | Lowland |  | (11) | (3) | |
| 261 | *Maquira* | 1.91 | 4 | Americas | 7.60 | 1 | Tree | MTF | Lowland |  | (11) | (3) | |
| 262 | *Maranthes* | 0.89 | 12 | Multiple | 8.67 | 0 | Tree | MTF | Lowland |  | (21) | (8) | |
| 263 | *Mareya* | 1.10 | 4 | Africa | 1.36 | 1 | Shrub, Tree | MTF | Lowland |  |  | (8) | |
| 264 | *Mareyopsis* | 0.57 | 2 | Africa | 0.56 | 1 | Shrub, Tree | MTF | Lowland |  | (8), | (8) | |
| 265 | *Margaritaria* | 2.39 | 13 | Multiple | 31.85 | 1 | Tree | MTF | Lowland |  | (11) | (3) | |
| 266 | *Markhamia* | 2.50 | 5 | Africa | 8.72 | 0 | Shrub, Tree | MTF, SDTF | Lowland |  | (15) | (8), (15) | |
| 267 | *Matayba* | 2.14 | 49 | Americas | 15.61 | 1 | Tree | MTF, SUB | Lowland, Montane |  | (3) | (3) | |
| 268 | *Matisia* | 1.27 | 60 | Americas | 6.01 | 0 | Tree | MTF | Lowland |  | (3) | (3) | |
| 269 | *Mauritia* | 1.99 | 2 | Americas | 9.35 | 1 | Tree | MTF | Lowland | 19000 | (11) | (3) | |
| 270 | *Maytenus* | 1.07 | 176 | Multiple | 38.10 | 1 | Tree | MTF, SUB, TEMP | Lowland, Montane |  | (11) | (3) | |
| 271 | *Memecylon* | 1.96 | 370 | Multiple | 15.10 | 0 | Shrub, Tree | MTF | Lowland |  | (15) | (8), (15), | |
| 272 | *Mesua* | 1.29 | 10 | Asia | 6.60 | 0 | Shrub, Tree | MTF, SDTF | Lowland |  |  |  | |
| 273 | *Metrodorea* | 3.09 | 6 | Americas | 6.88 | 0 | Tree | MTF | Lowland |  | (3) | (3) | |
| 274 | *Mezilaurus* | 0.48 | 24 | Americas | 10.46 | 0 | Tree | MTF | Lowland |  | (3) | (3) | |
| 275 | *Mezzettia* | 1.07 | 5 | Asia | 1.07 | 0 | Tree | MTF | Lowland |  | (56) | (56) | |
| 276 | *Miconia* | 4.74 | 1896 | Americas | 14.82 | 0 | Shrub, Tree | MTF | Lowland, Montane |  | (3) | (3) | |
| 277 | *Micrandra* | 0.71 | 9 | Americas | 5.85 | 1 | Tree | MTF | Lowland | 340 | (11) | (3) | |
| 278 | *Micrandropsis* | 0.33 | 1 | Americas | 1.10 | 0 | Tree | MTF | Lowland |  | (57) | (57) | |
| 279 | *Microcos* | 2.59 | 78 | Asia | 11.90 | 0 | Tree, Shrub | MTF | Lowland, Montane |  | (58) |  | |
| 280 | *Micropholis* | 1.42 | 38 | Americas | 11.49 | 1 | Tree | MTF | Lowland | 465 | (3) | (28) | |
| 281 | *Milicia* | 1.25 | 2 | Africa | 9.21 | 1 | Tree | MTF, SDTF, SAV | Lowland |  | (11) | (8) | |
| 282 | *Millettia* | 0.73 | 169 | Multiple | 17.07 | 0 | Shrub, Tree, Liana | MTF, SAV | Lowland |  |  | (8) | |
| 283 | *Minquartia* | 0.72 | 1 | Americas | 7.11 | 0 | Tree | MTF | Lowland |  | (3) | (3) | |
| 284 | *Monocarpia* | 1.16 | 4 | Asia | 0.19 | 0 | Tree | MTF | Lowland |  | (59) | (59); | |
| 285 | *Monodora* | 1.89 | 14 | Africa | 4.55 | 0 | Shrub, Tree | MTF | Lowland |  |  | (8) | |
| 286 | *Mora* | 0.39 | 6 | Americas | 3.01 | 0 | Tree | MTF | Lowland | 81163 |  |  | |
| 287 | *Moultonianthus* | 1.17 | 1 | Asia | 0.06 | 0 | Tree | MTF | Lowland |  | (60) | (60) | |
| 288 | *Mouriri* | 1.27 | 89 | Americas | 12.95 | 0 | Tree | MTF | Lowland | 75.1 | (3) | (3) | |
|  | **Genus** | **Mort.** | **Spp.** | **Distrib.** | **Range** | **Dio.** | **Lifeform** | **Biome** | **Elevation** | **Seed mass** | **Source: Breeding system** | **Source: Biome, Lifeform, Elevation** | |
|  |  | % a^-1^ |  |  | M km^2^ |  |  |  |  | g |  |  | |
| 289 | *Musanga* | 4.98 | 2 | Africa | 3.11 | 1 | Tree | MTF | Lowland |  | (11) | (8) | |
| 290 | *Myrcia* | 2.09 | 782 | Americas | 14.87 | 0 | Tree | MTF | Lowland, Montane |  | (3) | (3) | |
| 291 | *Myrciaria* | 1.06 | 30 | Americas | 14.95 | 0 | Tree | MTF | Lowland | 425.0 | (3) | (3) | |
| 292 | *Myrianthus* | 1.84 | 8 | Africa | 3.42 | 1 | Shrub, Tree | MTF | Lowland, Montane |  | (11) | (8), | |
| 293 | *Myristica* | 0.98 | 172 | Asia | 3.93 | 1 | Tree | MTF | Lowland |  | (11) | (19) | |
| 294 | *Nauclea* | 0.97 | 12 | Multiple | 11.63 | 0 | Tree | MTF | Lowland |  | (61) | (7) | |
| 295 | *Naucleopsis* | 1.40 | 25 | Americas | 11.32 | 1 | Tree | MTF | Lowland |  | (11) | (3) | |
| 296 | *Nectandra* | 2.65 | 99 | Americas | 15.40 | 0 | Tree | MTF | Lowland | 6628.0 | (3) | (3) | |
| 297 | *Neea* | 1.42 | 85 | Americas | 13.42 | 1 | Tree | MTF | Lowland | 117.0 | (11) | (3) | |
| 298 | *Neoscortechinia* | 1.43 | 6 | Asia | 1.06 | 1 | Tree | MTF | Lowland |  | (62) | (62) | |
| 299 | *Nephelium* | 1.97 | 23 | Asia | 2.96 | 1 | Tree, Tree | MTF, SAV | Lowland |  | (11) | (10) | |
| 300 | *Nesogordonia* | 0.66 | 22 | Africa | 7.48 | 0 | Tree | MTF, SDTF | Lowland |  | (29) | (8), (29) | |
| 301 | *Newtonia* | 1.72 | 16 | Multiple | 4.20 | 0 | Tree | MTF | Lowland |  | (3) |  | |
| 302 | *Ochna* | 0.95 | 79 | Africa | 28.99 | 0 | Shrub, Tree | MTF, SDTF, SAV | Lowland, Montane |  |  | (8) | |
| 303 | *Ochthocosmus* | 2.70 | 8 | Multiple | 6.25 | 0 | Shrub, Tree | MTF | Lowland |  | (44) | (44);(8) | |
| 304 | *Ocotea* | 1.81 | 539 | Multiple | 31.02 | 1 | Tree | MTF | Lowland |  | (11) | (3) | |
| 305 | *Octoknema* | 2.00 | 14 | Africa | 1.50 | 1 | Shrub, Tree | MTF | Lowland |  | (11) |  | |
| 306 | *Oenocarpus* | 1.75 | 10 | Americas | 8.30 | 0 | Tree | MTF | Lowland |  | (3) |  | |
| 307 | *Oncoba* | 2.95 | 4 | Africa | 9.71 | 1 | Shrub, Tree | MTF. SDTF | Lowland, Montane |  | (11) | (8), | |
| 308 | *Oncosperma* | 1.40 | 5 | Asia | 1.22 | 0 | Tree | MTF | Lowland |  | (63) | (63) | |
| 309 | *Ongokea* | 0.49 | 1 | Africa | 1.27 | 0 | Tree | MTF | Lowland |  | (7) | (7), (8) | |
| 310 | *Ormosia* | 1.46 | 131 | Multiple | 17.46 | 0 | Tree | MTF | Lowland |  | (3) |  | |
| 311 | *Osteophloeum* | 0.83 | 1 | Americas | 7.09 | 1 | Tree | MTF | Lowland |  | (11) | (3) | |
| 312 | *Oubanguia* | 0.41 | 3 | Africa | 0.61 | 0 | Tree | MTF | Lowland |  | (7) | (7), (8) | |
| 313 | *Ouratea* | 2.02 | 293 | Multiple | 15.51 | 0 | Tree | MTF | Lowland |  | (3) | (3) | |
| 314 | *Oxandra* | 1.86 | 28 | Americas | 12.87 | 0 | Tree | MTF | Lowland | 375.0 | (3) | (64) | |
| 315 | *Pachira* | 1.91 | 52 | Americas | 14.31 | 0 | Tree | MTF | Lowland | 2218.6 | (3) | (3) | |
| 316 | *Palaquium* | 1.44 | 120 | Asia | 3.87 | 0 | Tree | MTF | Lowland |  | (13) | (13) | |
| 317 | *Pancovia* | 1.26 | 13 | Africa | 8.45 | 1 | Shrub, Tree | MTF | Lowland |  | (22) | (22); (8) | |
| 318 | *Panda* | 0.51 | 1 | Africa | 1.44 | 1 | Tree | MTF | Lowland |  | (11) | (8) | |
| 319 | *Paramachaerium* | 0.79 | 5 | Americas | 4.09 | 0 | Tree | MTF | Lowland |  | (3) | (3) | |
| 320 | *Paranephelium* | 1.84 | 6 | Asia | 0.57 | 1 | Tree, Shrub | MTF | Lowland |  | (10) | (10) | |
| 321 | *Parashorea* | 1.95 | 13 | Asia | 0.65 | 0 | Tree | MTF | Lowland |  | (10) | (10) | |
| 322 | *Parinari* | 1.25 | 39 | Multiple | 23.57 | 0 | Tree | MTF | Lowland |  | (3) | (32) | |
| 323 | *Parkia* | 1.60 | 38 | Multiple | 18.89 | 0 | Tree | MTF | Lowland |  | (3) |  | |
| 324 | *Paropsia* | 0.94 | 12 | Africa | 8.82 | 0 | Tree, Shrub | MTF | Lowland |  | (7) | (7) | |
| 325 | *Pausandra* | 1.79 | 8 | Americas | 7.29 | 1 | Tree | MTF | Lowland |  | (11) | (3) | |
| 326 | *Pausinystalia* | 1.09 | 6 | Africa | 2.65 | 0 | Tree | MTF | Lowland |  |  | (20) | |
|  | **Genus** | **Mort.** | **Spp.** | **Distrib.** | **Range** | **Dio.** | **Lifeform** | **Biome** | **Elevation** | **Seed mass** | **Source: Breeding system** | **Source: Biome, Lifeform, Elevation** | |
|  |  | % a^-1^ |  |  | M km^2^ |  |  |  |  | g |  |  | |
| 327 | *Payena* | 1.61 | 20 | Asia | 0.52 | 0 | Tree | MTF | Lowland, Montane |  | (10) | (10), (65) | |
| 328 | *Peltogyne* | 0.92 | 24 | Americas | 12.29 | 0 | Tree | MTF | Lowland | 220.1 | (3) |  | |
| 329 | *Pentaclethra* | 1.06 | 3 | Multiple | 8.86 | 1 | Tree | MTF | Lowland |  | (11) | (3) | |
| 330 | *Pentadesma* | 1.12 | 5 | Africa | 1.86 | 0 | Tree | MTF | Lowland, Montane |  |  | (8), | |
| 331 | *Perebea* | 1.11 | 10 | Americas | 6.74 | 1 | Tree | MTF | Lowland | 629.0 | (11) | (3) | |
| 332 | *Petersianthus* | 0.83 | 2 | Africa | 3.53 | 0 | Tree | MTF | Lowland |  |  | (8) | |
| 333 | *Phyllocosmus* | 1.17 | 5 | Africa | 5.50 | 0 | Tree | MTF | Lowland |  |  | (8) | |
| 334 | *Picralima* | 1.08 | 1 | Africa | 2.15 | 0 | Shrub, Tree | MTF | Lowland |  | (7), (15) | (7), (15) | |
| 335 | *Pimelodendron* | 0.93 | 4 | Asia | 1.30 | 1 | Tree | MTF | Lowland |  | (66) | (66) | |
| 336 | *Piptadeniastrum* | 1.56 | 2 | Africa | 1.65 | 0 | Tree | MTF | Lowland |  | (15) | (8), (15) | |
| 337 | *Pithecellobium* | 1.39 | 24 | Multiple | 22.59 | 0 | Tree | SDTF, MTF | Lowland |  | (3) |  | |
| 338 | *Placodiscus* | 0.76 | 20 | Africa | 2.91 | 1 | Tree | MTF | Lowland |  | (7) | (7); (8) | |
| 339 | *Plagiostyles* | 1.16 | 1 | Africa | 0.96 | 1 | Shrub, Tree | MTF | Lowland |  | (11) | (8) | |
| 340 | *Platymiscium* | 1.63 | 19 | Americas | 13.29 | 0 | Tree | MTF | Lowland | 236.6 | (3) | (3) | |
| 341 | *Pleurothyrium* | 2.93 | 48 | Americas | 6.80 | 0 | Tree | MTF | Lowland, Montane |  | (3) | (3) | |
| 342 | *Poecilanthe* | 1.97 | 9 | Americas | 8.91 | 0 | Tree | MTF | Lowland | 1093.8 | (3) |  | |
| 343 | *Polyalthia* | 1.46 | 98 | Multiple | 12.26 | 0 | Tree | MTF | Lowland |  | (7) | (8) | |
| 344 | *Poraqueiba* | 0.62 | 3 | Americas | 6.79 | 0 | Tree | MTF | Lowland |  | (3) | (3) | |
| 345 | *Porterandia* | 2.36 | 22 | Multiple | 1.63 | 0 | Tree | MTF | Lowland |  | (67) | (67) | |
| 346 | *Poulsenia* | 2.61 | 1 | Americas | 3.97 | 0 | Tree | MTF | Lowland | 75.0 | (3) | (3) | |
| 347 | *Pourouma* | 2.69 | 31 | Americas | 8.85 | 1 | Tree | MTF | Lowland | 500.0 | (11) | (3) | |
| 348 | *Pouteria* | 0.87 | 201 | Multiple | 33.96 | 1 | Tree | MTF | Lowland |  | (11) | (28) | |
| 349 | *Pradosia* | 0.30 | 26 | Multiple | 12.40 | 0 | Tree | MTF | Lowland |  | (3) | (28) | |
| 350 | *Prioria* | 0.39 | 14 | Africa | 2.17 | 0 | Tree | MTF | Lowland |  |  | (8) | |
| 351 | *Protium* | 1.66 | 152 | Americas | 18.77 | 1 | Tree | MTF | Lowland | 1173.4 | (11) | (3), (16) | |
| 352 | *Protomegabaria* | 1.03 | 3 | Africa | 0.62 | 1 | Tree | MTF | Lowland |  | (11) | (8), | |
| 353 | *Pseudolmedia* | 1.21 | 11 | Americas | 11.13 | 1 | Tree | MTF | Lowland | 878.6 | (11) | (3) | |
| 354 | *Pseudospondias* | 1.19 | 2 | Africa | 3.83 | 1 | Shrub, Tree | MTF | Lowland, Montane |  | (22) | (8) | |
| 355 | *Pseudoxandra* | 2.11 | 24 | Americas | 9.70 | 0 | Tree | MTF | Lowland | 397.0 | (3) |  | |
| 356 | *Psidium* | 1.51 | 95 | Multiple | 15.63 | 0 | Tree | MTF | Lowland, Montane |  | (3) | (3) | |
| 357 | *Psydrax* | 2.84 | 96 | Multiple | 31.91 | 0 | Tree, Shrub | MTF, SDTF, SUB | Lowland |  |  |  | |
| 358 | *Pteleopsis* | 3.63 | 10 | Africa | 6.78 | 0 | Tree | MTF, SDTF, SAV | Lowland |  | (15) | (8), (15) | |
| 359 | *Pternandra* | 1.74 | 17 | Asia | 1.57 | 0 | Tree | MTF | Lowland |  |  |  | |
| 360 | *Pterocarpus* | 2.20 | 37 | Multiple | 28.18 | 0 | Tree | MTF | Lowland |  | (3) |  | |
| 361 | *Pterygota* | 1.63 | 21 | Multiple | 12.02 | 1 | Tree | MTF | Lowland |  | (3) | (3) | |
| 362 | *Ptychopyxis* | 1.30 | 11 | Asia | 1.35 | 1 | Tree | MTF | Lowland |  | (68) | (68) | |
| 363 | *Pycnanthus* | 1.31 | 4 | Africa | 3.95 | 1 | Tree, Liane | MTF | Lowland |  | (11) | (8), (69) | |
| 364 | *Qualea* | 0.77 | 53 | Americas | 12.03 | 0 | Tree | MTF | Lowland | 44.0 | (3) | (3) | |
| 365 | *Quararibea* | 0.81 | 55 | Americas | 12.46 | 0 | Tree | MTF | Lowland | 1827.1 | (3) | (3) | |
| 366 | *Quassia* | 1.36 | 1 | Multiple | 15.17 | 0 | Shrub, Tree | MTF, SAV | Lowland |  | (15) | (8) | |
|  | **Genus** | **Mort.** | **Spp.** | **Distrib.** | **Range** | **Dio.** | **Lifeform** | **Biome** | **Elevation** | **Seed mass** | **Source: Breeding_system** | **Source: Biome, Lifeform, Elevation** | |
|  |  | % a^-1^ |  |  | M km^2^ |  |  |  |  | g |  |  | |
| 367 | *Quiina* | 1.88 | 40 | Americas | 9.87 | 1 | Tree | MTF | Lowland | 505.0 | (3) | (3) | |
| 368 | *Rawsonia* | 1.65 | 2 | Africa | 4.15 | 0 | Shrub, Tree | MTF, SDTF | Lowland |  | (15) | (15); (8) | |
| 369 | *Rhodamnia* | 1.32 | 41 | Asia | 1.87 | 0 | Tree, Shrub | MTF | Lowland |  | (70) | (70) | |
| 370 | *Rhodostemonodaphne* | 1.26 | 41 | Americas | 7.43 | 1 | Tree | MTF | Lowland |  | (11) | (3) | |
| 371 | *Ricinodendron* | 2.57 | 1 | Africa | 5.16 | 1 | Tree | MTF, SDTF | Lowland |  | (11) | (8), (53) | |
| 372 | *Rinorea* | 1.44 | 213 | Multiple | 26.41 | 0 | Tree | MTF | Lowland, Montane |  | (3) | (3) | |
| 373 | *Rinoreocarpus* | 1.24 | 1 | Americas | 6.44 | 0 | Tree | MTF | Lowland |  | (3) | (3) | |
| 374 | *Rollinia* | 2.82 | 38 | Americas | 14.75 | 0 | Tree | MTF | Lowland | 151.1 | (3) | (3) | |
| 375 | *Rothmannia* | 1.69 | 23 | Multiple | 12.66 | 0 | Shrub, Tree | MTF | Lowland |  | (7) | (7), (8) | |
| 376 | *Roucheria* | 2.84 | 7 | Americas | 6.41 | 0 | Tree | MTF | Lowland |  | (3) | (3) | |
| 377 | *Ruizodendron* | 1.88 | 1 | Americas | 1.27 | 0 | Tree | MTF | Lowland |  | (3) | (3) | |
| 378 | *Sacoglottis* | 1.24 | 11 | Multiple | 12.24 | 0 | Tree | MTF | Lowland |  | (3) | (3) | |
| 379 | *Sagotia* | 1.29 | 3 | Americas | 5.74 | 0 | Tree | MTF | Lowland |  | (3) | (3) | |
| 380 | *Santiria* | 1.06 | 21 | Multiple | 1.04 | 0 | Tree | MTF | Lowland |  | (7) | (8) | |
| 381 | *Sapium* | 2.12 | 27 | Multiple | 26.26 | 1 | Tree | MTF | Lowland, Montane |  | (11) | (3) | |
| 382 | *Saraca* | 1.18 | 12 | Asia | 3.89 | 0 | Shrub, Tree | MTF | Lowland |  | (10) | (10) | |
| 383 | *Sarcaulus* | 1.15 | 5 | Americas | 7.51 | 1 | Tree | MTF | Lowland |  | (3) | (3) | |
| 384 | *Sarcotheca* | 1.04 | 12 | Asia | 0.10 | 0 | Shrub, Tree | MTF | Lowland |  | (10) | (10) | |
| 385 | *Scaphium* | 1.12 | 8 | Asia | 0.59 | 0 | Tree | MTF | Lowland |  | (71) | (71) | |
| 386 | *Scleronema* | 0.83 | 5 | Americas | 2.05 | 0 | Tree | MTF | Lowland |  | (3) | (3) | |
| 387 | *Scorodophloeus* | 0.66 | 3 | Africa | 1.73 | 0 | Tree | MTF | Lowland |  | (15) | (8), (15) | |
| 388 | *Scottellia* | 0.77 | 3 | Africa | 2.26 | 0 | Tree | MTF | Lowland |  | (72) | (8) | |
| 389 | *Scytopetalum* | 0.78 | 4 | Africa | 1.14 | 0 | Tree | MTF | Lowland |  |  | (8) | |
| 390 | *Shorea* | 1.50 | 50 | Asia | 2.44 | 0 | Tree | MTF | Lowland |  | (13) | (13) | |
| 391 | *Simaba* | 1.01 | 7 | Americas | 12.74 | 0 | Tree | MTF | Lowland |  | (3) | (3) | |
| 392 | *Simarouba* | 2.37 | 6 | Americas | 12.03 | 1 | Tree | MTF | Lowland | 521.5 | (11) | (3) | |
| 393 | *Sindora* | 0.78 | 21 | Multiple | 1.13 | 0 | Tree | MTF, SDTF | Lowland |  |  | (8) | |
| 394 | *Siparuna* | 1.98 | 53 | Americas | 13.32 | 1 | Shrub, Tree | MTF | Lowland, Montane |  | (11) | (3) | |
| 395 | *Sloanea* | 1.25 | 187 | Multiple | 21.15 | 0 | Tree | MTF | Lowland |  | (3) | (3) | |
| 396 | *Socratea* | 2.56 | 5 | Americas | 7.23 | 0 | Tree | MTF | Lowland | 3400.0 | (3) |  | |
| 397 | *Sorindeia* | 1.05 | 10 | Africa | 6.92 | 1 | Shrub, Tree | MTF, SAV | Lowland |  | (11) | (7) | |
| 398 | *Sorocea* | 1.91 | 22 | Americas | 11.66 | 1 | Tree | MTF | Lowland | 325.0 | (11) | (3) | |
| 399 | *Spondias* | 1.09 | 18 | Multiple | 29.16 | 1 | Tree | MTF | Lowland |  | (11) | (16) | |
| 400 | *Stachyothyrsus* | 0.99 | 2 | Africa | 0.06 | 0 | Tree | SDTF, SAV  MTF | Lowland |  |  | (8) | |
| 401 | *Staudtia* | 0.69 | 2 | Africa | 2.92 | 1 | Tree | MTF | Lowland |  | (69) | (8) | |
| 402 | *Stemonurus* | 1.86 | 13 | Asia | 1.65 | 0 | Shrub, Tree | MTF | Lowland |  | (73) | (73) | |
| 403 | *Sterculia* | 1.14 | 182 | Multiple | 33.41 | 1 | Tree | MTF | Lowland |  | (3) | (3) | |
|  | **Genus** | **Mort.** | **Spp.** | **Distrib.** | **Range** | **Dio.** | **Lifeform** | **Biome** | **Elevation** | **Seed mass** | **Source: Breeding_system** | **Source: Biome, Lifeform, Elevation** | |
|  |  | % a^-1^ |  |  | M km^2^ |  |  |  |  | g |  |  | |
| 404 | *Strombosia* | 1.12 | 11 | Multiple | 6.19 | 0 | Tree | MTF | Lowland |  | (15) | (15); (8) | |
| 405 | *Strombosiopsis* | 0.80 | 3 | Africa | 3.00 | 0 | Tree | MTF | Lowland |  | (15) | (8) | |
| 406 | *Stryphnodendron* | 2.25 | 28 | Americas | 11.15 | 0 | Tree | MTF | Lowland | 88.1 | (3) |  | |
| 407 | *Swartzia* | 0.69 | 193 | Multiple | 12.53 | 0 | Tree | MTF | Lowland |  | (3) |  | |
| 408 | *Symphonia* | 2.00 | 16 | Multiple | 17.99 | 0 | Tree | MTF | Lowland |  | (3) | (3) | |
| 409 | *Synsepalum* | 0.60 | 37 | Africa | 6.11 | 0 | Shrub, Tree | MTF | Lowland |  | (7) | (20) | |
| 410 | *Syzygium* | 1.34 | 1232 | Multiple | 28.96 | 0 | Shrub, Tree | MTF | Lowland, Montane |  |  | (8) | |
| 411 | *Tabebuia* | 1.18 | 76 | Americas | 17.73 | 0 | Tree | MTF, SDTF | Lowland |  | (3) | (3) | |
| 412 | *Tabernaemontana* | 2.16 | 126 | Multiple | 30.08 | 0 | Tree | MTF | Lowland |  | (3) | (3) | |
| 413 | *Tachigali* | 2.39 | 74 | Americas | 11.12 | 0 | Tree | MTF | Lowland | 866.1 | (3) | (3) | |
| 414 | *Talisia* | 1.01 | 53 | Americas | 12.49 | 1 | Tree | MTF | Lowland | 1075.0 | (3) | (3) | |
| 415 | *Tapirira* | 2.39 | 9 | Americas | 13.25 | 1 | Tree | MTF | Lowland | 283.2 | (11) | (16) | |
| 416 | *Tapura* | 1.13 | 36 | Multiple | 19.25 | 0 | Tree | MTF | Lowland |  | (3) | (3) | |
| 417 | *Tarenna* | 3.11 | 192 | Multiple | 16.11 | 0 | Shrub, Tree | SDTF, MTF | Lowland, Montane |  |  | (8) | |
| 418 | *Teijsmanniodendron* | 1.10 | 23 | Asia | 2.05 | 0 | Tree | MTF | Lowland |  | (74) | (74) | |
| 419 | *Terminalia* | 1.32 | 281 | Multiple | 40.55 | 0 | Tree | MTF | Lowland |  | (3) | (3) | |
| 420 | *Ternstroemia* | 2.13 | 160 | Multiple | 25.66 | 1 | Shrub, Tree | MTF, SUB | Lowland, Montane |  | (11) | (3) | |
| 421 | *Tessmannia* | 1.06 | 13 | Africa | 4.44 | 0 | Tree | MTF, SDTF | Lowland |  | (15) | (8) | |
| 422 | *Tetraberlinia* | 1.16 | 7 | Africa | 0.46 | 0 | Tree | MTF | Lowland |  |  | (8) | |
| 423 | *Tetragastris* | 0.82 | 8 | Americas | 10.02 | 1 | Tree | MTF | Lowland | 464.4 | (11) | (3), (16) | |
| 424 | *Tetrapleura* | 0.78 | 2 | Africa | 2.92 | 0 | Tree | MTF | Lowland |  |  | (8) | |
| 425 | *Tetrathylacium* | 1.63 | 5 | Americas | 2.52 | 0 | Tree | MTF | Lowland | 1.0 | (3) |  | |
| 426 | *Tetrorchidium* | 3.18 | 23 | Multiple | 17.40 | 1 | Tree | MTF | Lowland |  | (11) | (3) | |
| 427 | *Theobroma* | 1.42 | 20 | Americas | 9.19 | 0 | Tree | MTF | Lowland | 1648.0 | (3) | (3) | |
| 428 | *Thyrsodium* | 1.99 | 6 | Americas | 8.10 | 1 | Tree | MTF | Lowland |  | (3) | (16) | |
| 429 | *Timonius* | 1.34 | 194 | Asia | 5.98 | 1 | Shrub, Tree | MTF | Lowland |  | (75) | (75) | |
| 430 | *Tovomita* | 0.97 | 77 | Americas | 8.58 | 1 | Tree | MTF | Lowland | 1354.0 | (11) | (3) | |
| 431 | *Trattinnickia* | 1.65 | 20 | Americas | 8.07 | 1 | Tree | MTF | Lowland | 285.0 | (11) | (3), (16) | |
| 432 | *Treculia* | 0.82 | 5 | Africa | 8.77 | 1 | Shrub, Tree | MTF | Lowland |  | (11) | (8), (11) | |
| 433 | *Trema* | 9.47 | 18 | Multiple | 39.40 | 1 | Tree | MTF | Lowland |  | (3) | (3) | |
| 434 | *Tricalysia* | 2.21 | 81 | Multiple | 10.09 | 0 | Shrub, Tree | MTF, Savanna | Lowland |  | (7) | (7) | |
| 435 | *Trichilia* | 1.71 | 109 | Multiple | 35.59 | 1 | Tree | MTF | Lowland |  | (11) | (3) | |
| 436 | *Trichoscypha* | 0.92 | 32 | Multiple | 5.60 | 1 | Tree | MTF | Lowland |  | (11) | (8) | |
| 437 | *Tridesmostemon* | 0.28 | 2 | Africa | 1.83 | 0 | Tree | MTF | Lowland |  |  | (20) | |
| 438 | *Trilepisium* | 0.93 | 2 | Africa | 9.00 | 0 | Tree | MTF, SDTF | Lowland, Montane |  | (18) | (8) | |
| 439 | *Triplaris* | 2.67 | 19 | Americas | 12.37 | 1 | Tree | MTF | Lowland | 58.9 | (11) | (3) | |
| 440 | *Triplochiton* | 1.10 | 2 | Africa | 2.06 | 0 | Tree | MTF | Lowland |  | (29) | (8), (29) | |
| 441 | *Tristania* | 2.22 | 1 | Asia | 1.20 | 0 | Shrub, Tree | MTF | Lowland |  |  |  | |
| 442 | *Tristaniopsis* | 2.15 | 42 | Asia | 5.51 | 0 | Tree | MTF | Lowland |  | (76) | (76) | |
| 443 | *Trophis* | 2.42 | 5 | Americas | 9.56 | 1 | Shrub, Tree | MTF | Lowland |  | (11) | (3) | |
|  | **Genus** | **Mort.** | **Spp.** | **Distrib.** | **Range** | **Dio.** | **Lifeform** | **Biome** | **Elevation** | **Seed mass** | **Source: Breeding_system** | **Source: Biome, Lifeform, Elevation** | |
|  |  | % a^-1^ |  |  | M km^2^ |  |  |  |  | g |  |  | |
| 444 | *Turpinia* | 3.23 | 11 | Americas | 12.38 | 0 | Tree | MTF | Lowland | 50.0 | (3) | (3) | |
| 445 | *Turraeanthus* | 1.19 | 3 | Africa | 0.31 | 0 | Shrub, Tree | MTF | Lowland, Montane |  |  | (8) | |
| 446 | *Uapaca* | 1.64 | 25 | Africa | 13.63 | 1 | Tree | MTF | Lowland |  | (11) | (13) | |
| 447 | *Unonopsis* | 1.28 | 48 | Americas | 10.44 | 0 | Tree | MTF | Lowland | 326.7 | (3) | (3) | |
| 448 | *Uvariopsis* | 1.16 | 19 | Africa | 2.77 | 1 | Shrub, Tree, treelet | MTF | Lowland |  | (11) | (8) | |
| 449 | *Vangueria* | 4.63 | 58 | Africa | 10.47 | 0 | Shrub, Tree | MTF, Semiarid | Lowland, Montane |  | (26) | (26) | |
| 450 | *Vantanea* | 0.60 | 23 | Americas | 7.74 | 0 | Tree | MTF | Lowland |  | (3) | (3) | |
| 451 | *Vatica* | 1.05 | 77 | Asia | 3.71 | 0 | Shrub, Tree | MTF | Lowland |  |  | (13) | |
| 452 | *Vepris* | 1.94 | 93 | Africa | 15.15 | 1 | Shrub, Tree | MTF, SDTF | Lowland, Montane |  |  | (8) | |
| 453 | *Virola* | 1.71 | 71 | Americas | 11.88 | 1 | Tree | MTF | Lowland | 1666.2 | (11) | (3) | |
| 454 | *Vismia* | 4.72 | 47 | Multiple | 22.08 | 0 | Tree | MTF | Lowland |  | (3) | (3) | |
| 455 | *Vitex* | 1.02 | 209 | Multiple | 54.15 | 0 | Tree | MTF | Lowland |  | (3) | (3) | |
| 456 | *Vochysia* | 2.27 | 147 | Americas | 12.55 | 0 | Tree | MTF | Lowland | 92.9 | (3) | (3) | |
| 457 | *Vouacapoua* | 0.62 | 3 | Americas | 2.47 | 0 | Tree | MTF | Lowland | 15040 |  |  | |
| 458 | *Xanthophyllum* | 1.50 | 108 | Multiple | 5.54 | 0 | Shrub, Tree | MTF | Lowland |  |  | (13) | |
| 459 | *Xerospermum* | 2.09 | 2 | Asia | 1.25 | 0 | Tree, Shrub | MTF, SUB | Lowland, Montane |  | (10) | (10) | |
| 460 | *Xylopia* | 1.81 | 185 | Multiple | 26.45 | 0 | Tree | MTF | Lowland |  | (3) | (3) | |
| 461 | *Xymalos* | 0.92 | 1 | Africa | 1.30 | 1 | Shrub, Tree | MTF, SUB | Lowland, Montane |  | (11) | (7) | |
| 462 | *Zanthoxylum* | 2.63 | 232 | Multiple | 57.88 | 1 | Tree | MTF, SDTF | Lowland |  | (11) | (3) | |
| 463 | *Zygia* | 1.26 | 60 | Americas | 12.60 | 0 | Tree | MTF | Lowland |  | (3) |  | |

**Supplementary Table S5 References**

1. Seed Information Database (SID) Version 7.1 (2008) Seed Information Database (SID) Version 7.1. (Royal Botanic Gardens Kew).

2. POWO (2022) Plants of the World Online. Facilitated by the Royal Botanic Gardens, Kew. Published on the Internet <http://www.plantsoftheworldonline.org/>.

3. Pennington TD, Reynel C, & Daza A (2004) *Illustrated guide to the trees of Peru* (DH books).

4. De-Nova JA, Sosa V, & Steinmann VW (2007) A synopsis of Adelia (Euphorbiaceae ss). *Systematic Botany* 32(3):583-595.

5. Pathak M, Bhaumik M, & Krishna G (2014) A new species of *Adinandra* (Pentaphylaceae) from India. *Edinburgh Journal of Botany* 71(3):379-383.

6. Tsou C-H (1995) Embryology of Theaceae - Anther and ovule development of Adinandra, Cleyera and Eurya. *Journal of Plant Research* 108(1):77-86.

7. Hutchinson J, Dalziel J, Keay R, Hepper F, & Alston A (1954-1972) *Flora of West Tropical Africa* ( Crown Agents for Oversea Governments and Administrations, London, UK).

8. African Plant Database (version 3.4.0) (Conservatoire et Jardin botaniques de la Ville de Genève and South African National Biodiversity Institute, Pretoria).

9. Grudinski M, Wanntorp L, Pannell CM, & Muellner‐Riehl AN (2014) West to east dispersal in a widespread animal‐dispersed woody angiosperm genus (Aglaia, Meliaceae) across the Indo‐Australian Archipelago. *Journal of Biogeography* 41(6):1149-1159.

10. Soepadmo E & Wong KM (1995) *Tree flora of Sabah and Sarawak* (Forest Research Institute Malaysia).

11. Renner SS (2014) The relative and absolute frequencies of angiosperm sexual systems: dioecy, monoecy, gynodioecy, and an updated online database. *Americasn Journal of Botany* 101(10):1588-1596.

12. Leeratiwong C, Chalermglin P, & Johnson D (2020) Taxonomic notes on the genus Alphonsea (Annonaceae) in Thailand. *Thai Forest Bulletin (Botany)* 48(1):24-33.

13. Sasidharan N (2006) Illustrated manual on tree flora of Kerala supplemented with computer-aided identification. *Kerala Forest Research Institute, Peechi, Kerala. 698p*.

14. Sidiyasa K (1998) Taxonomy, phylogeny, and wood anatomy of Alstonia (Apocynaceae). *Blumea. Supplement* 11(1):1-230.

15. Flora of Tropical East Africa (1948-2012) (Royal Botanic Gardens Kew).

16. Weeks A*, et al.* (2014) To move or to evolve: contrasting patterns of intercontinental connectivity and climatic niche evolution in “Terebinthaceae” (Anacardiaceae and Burseraceae). *Frontiers in Genetics* 5.

17. Juncosa AM & Tomlinson PB (1988) A historical and taxonomic synopsis of Rhizophoraceae and Anisophylleaceae. *Annals of the Missouri Botanical Garden*:1278-1295.

18. Flora Zambesiaca (1991) vol. 9 Ulmaceae to Ceratophylaceae.).

19. Flora Malesiana (Various dates) Series I: Volume 11-1 (1992), 11-2 (1993), 11-3 (1994), 12-1 (1995), 12-2 (1996), 13 (1997), 14 (2000), 15 (2001); Series II: Volume 2-1 (1991), 3 (1998).

20. Burkill HM (1995) *The useful plants of west tropical Africa, Vols. 1-3* Second Ed.

21. Flora Zambesiaca (1978) vol. 4 Rosaceae to Cornaceae.

22. Flora Zambesiaca (1966) vol. 2 Aquifoliaceae to Connaraceae.

23. Ottens-Treurniet M & van Welzen P (2016) A revision of the Malesian genus Blumeodendron (Euphorbiaceae). *Blumea-Biodiversity, Evolution and Biogeography of Plants* 61(1):64-82.

24. Dressler S (1996) The genus Bridelia (Euphorbiaceae) in Malesia. *Blumea* 41:263-331.

25. Li Y, Dressler S, Zhang D, & Renner SS (2009) More Miocene dispersal between Africa and Asia—the case of Bridelia (Phyllanthaceae). *Systematic Botany* 34(3):521-529.

26. Flora Zambesiaca (1998) vol. 5 Rubiaceae: tribe Vanguerieae.

27. Widuri R & Van Welzen P (1998) A revision of the genus Cephalomappa (Euphorbiaceae) in Malesia. *Reinwardtia* 11(3):153-184.

28. Swenson U & Anderberg AA (2005) Phylogeny, character evolution, and classification of Sapotaceae (Ericales). *Cladistics* 21(2):101-130.

29. Flora Zambesiaca (1961) vol 1 Caryophyllaceae to Sterculiaceae.

30. Stoffelen P, Robbrecht E, & Smets E (1996) A revision of Corynanthe and Pausinystalia (African Rubiaceae-Coptosapelteae). *Botanical Journal of the Linnean Society* 120(4):287-326.

31. Huang Y-Y, Mori SA, & Kelly LM (2015) Toward a phylogenetic-based generic classification of neotropical lecythidaceae—I. Status of Bertholletia, Corythophora, Eschweilera and Lecythis. *Phytotaxa* 203(2):85–121-185–121.

32. Bardon L*, et al.* (2013) Origin and evolution of Chrysobalanaceae: insights into the evolution of plants in the Neotropics. *Botanical Journal of the Linnean Society* 171(1):19-37.

33. van der Werff H (2001) An annotated key to the genera of Lauraceae in the Flora Malesiana region. *Blumea: Biodiversity, Evolution and Biogeography of Plants* 46(1):125-140.

34. De Kok R (2015) A revision of Cryptocarya (Lauraceae) from Thailand and Indochina. *Gard. Bull. Singapore* 67(2):309-350.

35. Wang RJ & Saunders RM (2006) The genus Cyathocalyx (Annonaceae) in the Philippines. *Systematic Botany* 31(2):285-297.

36. Prance GT & White F (1979) Resurrection of the genus Dactyladenia (Chrysobalanaceae). *Brittonia* 31(4):483-487.

37. Saiful Yazan L & Armania N (2014) Dillenia species: A review of the traditional uses, active constituents and pharmacological properties from pre-clinical studies. *Pharmaceutical Biology* 52(7):890-897.

38. Ali S & Robbrecht E (1991) Remarks on the tropical Asian and Australian taxa included in Diplospora or Tricalysia (Rubiaceae—Ixoroideae—Gardenieae). *Blumea: Biodiversity, Evolution and Biogeography of Plants* 35(2):279-305.

39. Ghazoul J (2016) *Dipterocarp biology, ecology, and conservation* (Oxford University Press).

40. Chua L & Saw L (2003) A new record of Dryobalanops beccarii (Dipterocarpaceae) from Peninsular Malaysia. *The Gardens' Bulletin Singapore* 55:1-6.

41. Kostermans AJG (1958) The genus Durio adans.(Bombac.). *Reinwardtia* 4(3):357-460.

42. Shaw HA (1972) The Euphorbiaceae of Siam. *Kew Bulletin*:191-363.

43. Flora Zambesiaca (1983) vol. 7 Escalloniaceae to Salvadoraceae.

44. Flora Zambesiaca (1963) vol. 2 Tiliaceae to Icacinaceae.

45. Kurokawa H, Yoshida T, Nakamura T, Lai J, & Nakashizuka T (2003) The age of tropical rain-forest canopy species, Borneo ironwood (Eusideroxylon zwageri), determined by 14C dating. *Journal of Tropical Ecology* 19(1):1-7.

46. Flora Zambesiaca (1985) vol. 7 Apocynaceae.

47. De La Estrella M & Devesa JA (2014) Gilbertiodendron grandistipulatum (Leguminosae-Caesalpinioideae), a singular species from West Central Africa and new record for Congo (Brazzaville). *Boletín de la Sociedad Argentina de Botánica* 49(1):137-144.

48. Utteridge T & Schori M (2011) Updating Malesian Icacinaceae. *Gardens’ Bulletin Singapore* 63(1-2):105-118.

49. Lissambou B-J*, et al.* (2018) Taxonomic revision of the African genus Greenwayodendron (Annonaceae). *PhytoKeys* (114):55.

50. Hilje B, Calvo-Alvarado J, Jiménez-Rodríguez C, & Sánchez-Azofeifa A (2015) Tree species composition, breeding systems, and pollination and dispersal syndromes in three forest successional stages in a tropical dry forest in MesoAmericas. *Tropical Conservation Science* 8(1):76-94.

51. Jose Robi A, Sasidharan N, & Antony Jose P (2014) Hydnocarpus longipedunculatus (Achariaceae: Flacourtiaceae, sl): a new species from the Western Ghats, India. *Webbia* 69(2):243-247.

52. van Welzen P (2010) Revision of the Asian Genus Koilodepas (Euphorbiaceae) 1. *Annals of the Missouri Botanical Garden* 97(2):218-234.

53. Flora Zambesiaca (1996) vol. 9 Euphorbiaceae: Heywoodia to Maprounea.

54. Yii P & Chai P (2001) New combinations, new names and new species of Madhuca (Sapotaceae) from Sabah and Sarawak, Borneo. *Gard Bull Singap* 53:342-356.

55. Armstrong K*, et al.* (2014) Patterns of diversification amongst tropical regions compared: a case study in Sapotaceae. *Evolutionary and Population Genetics* 5:362.

56. Van der Heijden E & Keßler P (1990) Studies on the tribe Saccopetaleae (Annonaceae)—III Revision of the genus Mezzettia Beccari. *Blumea: Biodiversity, Evolution and Biogeography of Plants* 35(1):217-228.

57. Rodrigues WA (1973) Micrandropsis, novo gênero de Euphorbiaceae da Amazônia. *Acta Amazonica* 3:5-6.

58. Chung R & Soepadmo E (2011) Taxonomic revision of the genus Microcos (Malvaceae-Grewioideae) in Peninsular Malaysia and Singapore. *Blumea-Biodiversity, Evolution and Biogeography of Plants* 56(3):273-299.

59. Chaowasku T*, et al.* (2018) Enlarging the monotypic Monocarpieae (Annonaceae, Malmeoideae): recognition of a second genus from Vietnam informed by morphology and molecular phylogenetics. *Candollea* 73(2):261-275.

60. Welzen PC (1995) Taxonomy and phylogeny of the Euphorbiaceae tribe Erismantheae GL Webster (Erismanthus, Moultonianthus, and Syndyophyllum). *Blumea: Biodiversity, Evolution and Biogeography of Plants* 40(2):375-396.

61. Haviland GD (1897) A revision of the tribe Naucleae (Nat. Ord Rubiaceae). *Botanical Journal of the Linnean Society* 33:1-94.

62. van Welzen PC (1994) Taxonomy, phylogeny, and geography of Neoscortechinia Hook. f. ex Pax (Euphorbiaceae). *Blumea: Biodiversity, Evolution and Biogeography of Plants* 39(1/2):301-320.

63. Loo AH, Foong AW, Baker WJ, & Tan HT (2015) An Introduction to the Native Palms of Singapore. *Palms* 59(3).

64. Couvreur TL*, et al.* (2011) Early evolutionary history of the flowering plant family Annonaceae: steady diversification and boreotropical geodispersal. *Journal of Biogeography* 38(4):664-680.

65. Tam S (1999) Floristic Diversity of Bukit Bauk (Terengganu), Peninsular Malaysia. *Gardens’ Bulletin Singapore* 51:257-308.

66. Djarwaningsih T (2004) Revision of Pimelodendron (Euphorbiaceae) in Malesia. *Blumea-Biodiversity, Evolution and Biogeography of Plants* 49(2-3):407-423.

67. Zahid M & Wong K (2010) The circumscription, taxonomy and biogeography of Porterandia (Rubiaceae–Gardenieae). *Edinburgh Journal of Botany* 67(2):265-342.

68. Stoops E & van Welzen PC (2013) A revision of Ptychopyxis (Euphorbiaceae) in southeast Asia. *Nordic Journal of Botany* 31(1):094-112.

69. Flora Zambesiaca (1997) vol. 9 Podostemaceae to Hernandiaceae.

70. Snow N (2007) Systematics of the Australian species of Rhodamnia (Myrtaceae). *Systematic Botany Monographs*:1-69.

71. Wilkie P (2009) A revision of Scaphium (Sterculioideae, Malvaceae/Sterculiaceae). *Edinburgh Journal of Botany* 66(2):283-328.

72. Sleumer H (1972) A taxonomic revision of the genus Scottellia Oliv.(Flacourtiaceae). *Blumea: Biodiversity, Evolution and Biogeography of Plants* 20(2):275-281.

73. Ridley H (1922) The flora of the Malay Peninsula, vol. 1. London: Reeve.

74. De Kok R, Rusea G, & Latiff A (2009) The genus Teijsmanniodendron Koord.(Lamiaceae). *Kew Bulletin* 64(4):587-625.

75. Darwin SP (2010) Six new species of Timonius (Rubiaceae: Guettardeae) from Papuasia. *Brittonia* 62(2):126-136.

76. Ahmad B & Wilson PG (2015) Two new species of Tristaniopsis (Myrtaceae) from Sabah. *Telopea* 18:475-479.
